# A method for the efficient evaluation of substrate-based cholinesterase imaging probes for Alzheimer’s disease

**DOI:** 10.1080/14756366.2023.2225797

**Published:** 2023-06-26

**Authors:** Sultan Darvesh, Scott Banfield, Maeve Dufour, Katrina L. Forrestall, Hillary Maillet, G. Andrew Reid, Dane Sands, Ian R. Pottie

**Affiliations:** a Department of Medical Neuroscience, Dalhousie University, Halifax, Nova Scotia, Canada; b Department of Medicine (Geriatric Medicine & Neurology), Halifax, Nova Scotia, Canada; c Department of Chemistry and Physics, Mount St. Vincent University, Halifax, Nova Scotia, Canada; d Department of Chemistry, Saint Mary’s University, Halifax, Nova Scotia, Canada

**Keywords:** Butyrylcholinesterase, acetylcholinesterase, Alzheimer’s disease, neuroimaging, radioligands

## Abstract

Cholinesterase (ChE) enzymes have been identified as diagnostic markers for Alzheimer disease (AD). Substrate-based probes have been synthesised to detect ChEs but they have not detected changes in ChE distribution associated with AD pathology. Probes are typically screened using spectrophotometric methods with pure enzyme for specificity and kinetics. However, the biochemical properties of ChEs associated with AD pathology are altered. The present work was undertaken to determine whether the Karnovsky-Roots (KR) histochemical method could be used to evaluate probes at the site of pathology. Thirty thioesters and esters were synthesised and evaluated using enzyme kinetic and KR methods. Spectrophotometric methods demonstrated all thioesters were ChE substrates, yet only a few provided staining in the brain with the KR method. Esters were ChE substrates with interactions with brain ChEs. These results suggest that the KR method may provide an efficient means to screen compounds as probes for imaging AD-associated ChEs.

## Introduction

Alzheimer’s Disease (AD) is the leading cause of dementia worldwide, accounting for nearly 70% of dementing illness^1^^,^^2^. The prevalence of AD continues to grow, increasing demand on caregivers, care facilities, and families^2^. Tools for the early diagnosis and treatment monitoring of AD have become increasingly important.

Nuclear imaging probes have been explored as a diagnostic tool to detect brain AD pathology, including amyloid-β (Aβ) plaques^3^^,^^4^ and tau neurofibrillary tangles (NFTs)^5^^,^^6^. Although the presence of Aβ plaques and tau NFTs in AD is well recognised, their implication in neurodegeneration is not fully understood^7^^,^^8^. This is highlighted by the observation of Aβ plaques and tau NFTs in post-mortem brain tissues of cognitively normal older adults^9–13^. In fact, it has been found that up to 30% of elderly cognitively normal individuals have brain Aβ plaques and tau NFTs^9^^,^^11^^,^^12^^,^^14^. This suggests that other more specific diagnostic targets would be valuable.

In addition to NFTs and Aβ plaque deposition, AD is characterised by changes to the cholinergic system, including the loss of cholinergic neurons and the resulting decrease in the neurotransmitter acetylcholine (ACh)^15^. The metabolising enzymes of ACh, acetylcholinesterase (AChE; EC 3.1.1.7) and butyrylcholinesterase (BChE; EC 3.1.1.8), are also altered in AD. In the normal brain, histochemical staining for these enzymes has shown distinct distributions associated with neural elements^16–20^. However, in AD, AChE and BChE become associated with Aβ plaques and NFTs^21–26^. In addition, AChE levels have been shown to decrease^27^ while BChE has been shown to increase in abundance^27^ or remain the same^26^. Although Aβ plaques and NFTs have been observed in cognitively normal brains^12^^,^^14^^,^^28^, the association of BChE with these pathological structures is more specific to AD^28^. Furthermore, BChE-associated pathology is not present in any other dementias, such as frontotemporal dementia with tau or dementia with Lewy bodies^28^. The changes to the levels of cholinesterases (ChEs) in AD and the specific association of BChE with AD pathology suggest these enzymes are promising targets to facilitate diagnosis and treatment monitoring of AD.

Substrate-based imaging probes have been developed for the quantification of ChE activity in the brain. Positron emission tomography (PET) imaging of AChE using the substrate probes of *N*-methylpiperidinyl esters, notably 4-(*N*-[^11^C]methyl) piperidinyl acetate ([^11^C]AMP) and 4-(*N*-[^11^C]methyl) piperidinyl propionate ([^11^C]PMP), have been evaluated previously^29–39^. These compounds have generally recapitulated known brain distributions and activity levels of AChE, with uptake in the striatum, cortex, and cerebellum^29–31^^,^^36^, regions known to have high levels of AChE^40^. In addition, these probes have demonstrated *in vivo* reduction of AChE levels in AD brains^31–33^^,^^36–38^, recapitulating protein levels found post-mortem^26^^,^^27^. Similarly, BChE-specific probes such as 1-^11^C-methyl-4-piperidinyl n-butyrate (^11^C-MP4B)^41–43^ and *N*-methyl piperidinyl *p*-fluoro benzoate^44^ and *N*-methyl piperidinyl *p*-iodo benzoate^45^ have demonstrated uptake in the cerebellum and midbrain, regions known to contain high levels of BChE activity associated with neural elements^46^^,^^47^. However, neither AChE nor BChE imaging probes have shown increases in ChE activity associated with Aβ plaques in the AD brain. These observations necessitate further development of ChE probes for AD diagnosis.

Prior to radiolabelling, methods to efficiently screen novel AD imaging probes are required. For ChE substrate-based probes, enzyme kinetic methods are typically used to determine their specificity for AChE or BChE as well as their kinetic parameters using spectrophotometric methods. These methods use either pure enzymes or those isolated from brain tissues. However, it has been shown that the properties of AChE and BChE are altered when associated with Aβ plaques and tau NFTs in AD^22^^,^^26^^,^^48^. Consequently, it will be useful to evaluate novel probes with ChEs when associated with this AD pathology.

In a previous study, Macdonald et al.^49^ evaluated a series of *N*-methylpiperidinyl thioesters for staining of AChE or BChE in human brain tissue using the Karnovsky-Roots (KR) histochemical method^50^. It was found that thioesters could be employed for the histochemical detection of the enzyme activity associated with normal neural structures in brain tissues and that this staining could be used for comparison with *in vivo* images generated by analogous radiolabelled molecules. Herein we expand on this work to develop a means to rapidly evaluate the association of ChE substrates with normal neural and AD pathological structures for eventual use as AD diagnostic molecular imaging probes. Five groups of known and new esters and corresponding thioesters were synthesised and evaluated, including analogues of choline, alkyl *N*-methylpiperidinol, aryl *N*-methylpiperidinol, alkyl *N*-methylpyrrolidinol, and aryl *N*-methylpyrrolidinol. Thioesters allowed for the direct evaluation of specificity and determination of enzyme kinetic parameters with AChE or BChE. They also allowed for the evaluation of the interaction of these compounds with ChEs in human and mouse AD brain tissues using the KR method, as done previously^49^. Esters were evaluated for specificity and determination of enzyme kinetic parameters with AChE or BChE using competition studies with a spectrophotometric method, as described previously^51–53^. For their interaction with ChEs associated with AD pathology, esters were used to determine whether they prevented the staining of AChE or BChE with their respective substrates in KR histochemical methods. These experiments showed that the histochemical method may prove to be more reliable than enzyme kinetic methods when evaluating potential ChE probes for AD pathology.

## Materials and methods

### Materials

Solvents used were purchased from Fisher Scientific (https://www.fishersci.ca) or VWR International (https://ca.vwr.com). Acetylcholine iodide (AChI), acetylthiocholine iodide (ATChI), butyrylcholine iodide (BChI), butyrylthiocholine iodide (BTChI), purified recombinant human AChE, 3,3′- diaminobenzidine tetrahydrochloride (DAB), 1-methylpiperidin-4-ol, (*S*)-1-methylpyrrolidin-3-ol, (*R*)-1-methylpyrrolidin-3-ol, 4-iodobenzoyl chloride, 4-fluorobenzoyl chloride, acetyl chloride, butyryl chloride, iodomethane, 2-(dimethylamino)ethanethiol hydrochloride, potassium thioacetate, oxalic acid, gelatine, sodium azide, 1,5-Bis(4-allyldimethylammoniumphenyl)pentan-3-one-dibromide (BW 284c51), and deuterated solvents were purchased from Sigma-Aldrich (https://www.sigmaaldrich.com) or Oakwood Chemical (https://oakwoodchemical.com). BChE purified from human plasma was a gift from Dr. Oksana Lockridge (Eppley Institute, University of Nebraska Medical Centre, Omaha, Nebraska, United States). Gaseous argon (99.999% purity) was purchased from Air Liquide (https://www.airliquide.ca). *Ortho*-nitrofluoroacetanilide (*o-*NTFNAC) was synthesised as previously described^54^.

### Characterisation of synthetic compounds

Nuclear magnetic resonance (NMR) spectra were obtained using a Bruker Avance Neo 400 NMR, equipped with a 5 mm X-nuclei-optimized double resonance broad band smart probe (PA BBO BBF-H-D-05 OZ SP IP) and two TRX1200 transceivers, operated at 400.3 MHz for ^1^H or 100.7 MHz for ^13^C, and referenced internally to the NMR solvent or tetramethyl silane. For broadband decoupled ^19^F experiments, the NMR operated at 376.6 MHz and the field was referenced externally to α,α,α-trifluorotoluene (−63.72 ppm) in CDCl_3_ immediately before running the ^19^F experiment. Chemical shifts were reported in parts per million (ppm), the coupling constants were reported in hertz (Hz) and the multiplicities were apparent. NMR experiments performed on all synthetic compounds were: ^1^H-^13^C Uniform Driven Equilibrium Fourier Transform (UDEFT), ^1^H-^13^C heteronuclear single quantum coherence (HSQC), ^1^H-^13^C heteronuclear multiple bond correlation (HMBC), ^1^H-^15^N HMBC, and ^1^H-^1^H correlated spectroscopy (COSY). All samples were run at 298.0 K in Wilmad high throughput NMR sample tubes (WG-1000–7). Spectral data was analysed using Topspin 4.1.1 software (https://www.bruker.com/). For ^13^C NMR data, the number of attached protons for each signal, obtained from a default phase-sensitive HSQC experiment, are given in parentheses. Melting points were determined using a Fisher-Johns melting point apparatus and all values are uncorrected. Infra-red (IR) spectra were collected using an Alpha Bruker attenuated total reflection (ATR) Fourier transform infra-red (FTIR) spectrometer and analysed using OPUS 7.5 software (Bruker, Massachusetts, United States). Low- and high-resolution mass spectral data was performed by Mass Spec Services (Department of Chemistry, Dalhousie University, Halifax, Nova Scotia, Canada) using a Compact QTOF mass spectrometer from Bruker Daltonics. The ionisation method used was electrospray ionisation (ESI) and samples were introduced by syringe pump at 3 µL/min. The spray voltage applied to the ESI needle was 3.5 kV. Dry gas flow rate was 4 L/min and nebuliser gas was 0.5 Bar with a source temperature of 180 °C. Ultraviolet (UV) absorbance for each compound was obtained using a VWR UV-1600PC spectrophotometer and analysed using M.Wave Professional software 1.0.20 (Azzota Scientific, Delaware, United States). The purity of all compounds was determined using an Agilent Technologies 1200 series high-performance liquid chromatography (HPLC) system equipped with a photodiode UV-visible detector, quaternary pump, column heater set to 25 °C, and Eclipse XDB-C18 (4.6 × 150 mm, 5 µm) column. Spectroscopic data for all compounds is included in the Supplementary Material.

### Chemical synthesis

**Scheme 1.**
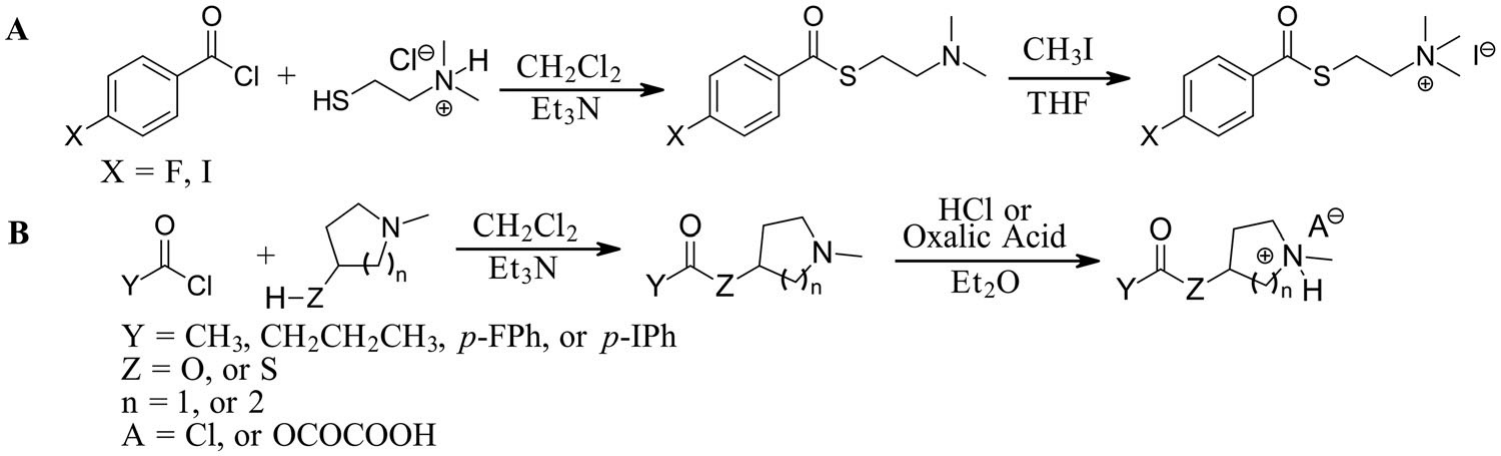
Synthetic schemes. A. An acyl substitution strategy was employed to generate thiocholine derivatives. The acyl chloride was mixed with 2-(dimethylamino)ethanethiol hydrochloride, triethylamine, and dichloromethane to afford the corresponding thioester. The quaternary amine was produced from reaction with methyl iodide in THF. B. An acyl substitution strategy was used to generate the piperidinyl and pyrrolidinyl derivatives. The appropriate acid chloride was mixed with the *N*-methylpiperidinol, *R* or *S N*-methylpyrrolidinol in dichloromethane and triethylamine to produce the ester/thioester. The ammonium salt was formed by reaction with HCl or oxalic acid. Legend: *p*-FPh = *para*-fluorophenyl; *p*-IPh = *para*-iodophenyl.

Compounds **1**–**4** (Table 1) were purchased commercially. Compounds **5–30** (Table 1) were chemically synthesised using an acyl substitution strategy that started from the appropriate acid chloride (Scheme 1). The series of compounds assessed herein were divided into 5 categories based on their chemical structures: choline esters and thioesters (**1**–**6**); alkyl *N*-methyl piperidinyl thioesters and esters (**7**–**10**); aryl *N*-methyl piperidinyl thioesters and esters (**11**–**14**), alkyl *N*-methyl pyrrolidinyl thioesters and esters (**15**–**22**), and aryl *N*-methyl pyrrolidinyl thioesters and esters (**23**–**30**). The synthetic procedures and spectroscopic data for all compounds is included in the Supplementary Material. Compounds were synthesised in moderate to high yields ranging from (16–99%) with ≥97% purity by HPLC for the majority of the compounds except compound **8** (91.5%), compound **16** (95%), and compound **19** (89%). Spectroscopic analysis confirmed the identity of all chemical structures. Chemical structures were drawn using ChemDoodle (Version 11.13.0, https://www.chemdoodle.com/).

**Table 1.** Catalytic parameters (*k*_cat_, *K_m_,* and *k*_cat_/*K_m_*) of substrate hydrolysis by AChE and BChE.

		AchE			BChE		
	Structure	*k*_cat_ x 10^3^ (min^-1^)	*K_m_* x 10^-6^ (M)	*k*_cat_/*K_m_* x 10^7^ (min^-1^ M^-1^)	*k*_cat_ x 10^3^ (min^-1^)	*K_m_* x 10^-6^ (M)	*k*_cat_/*K_m_* x 10^7^ (min^-1^ M^-1^)
Choline Thioesters & Esters							
**1**	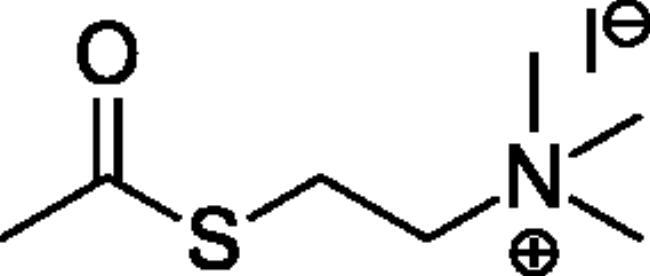	700^52^	150^52^	460^52^	50^55^	40^55^	130^55^
**2**	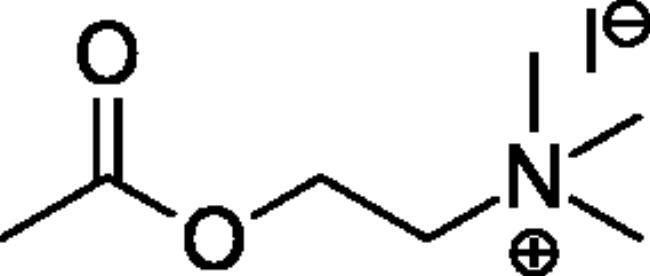	260^52^	175^52^	150^52^	13^52^	1400^52^	12^52^
**3**	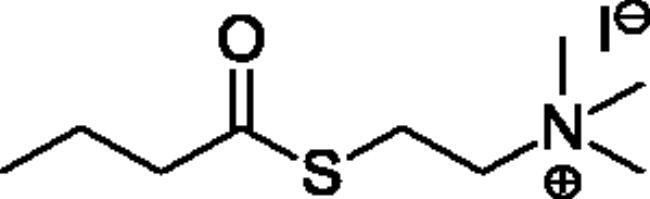	Not a substrate	Not a substrate	Not a substrate	24^52^	18^52^	130^52^
**4**	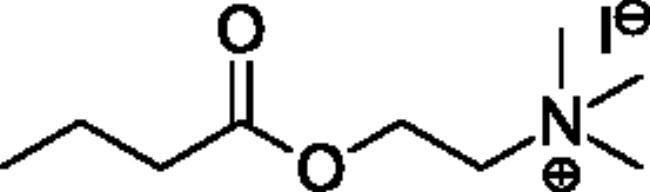	Not a substrate*	Not a substrate*	Not a substrate*	80^56^	910^56^	8.8^56^
**5**	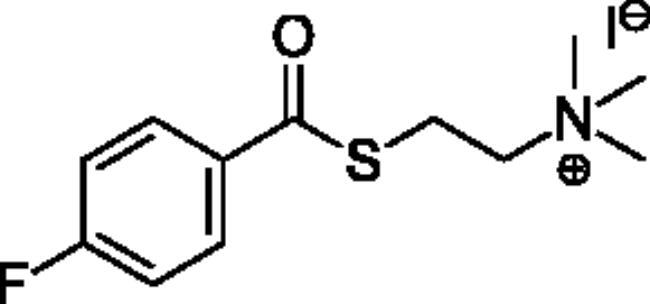	Not a substrate	Not a substrate	Not a substrate	5.81 ± 0.27	59.2 ± 7.4	9.88 ± 0.79
**6**	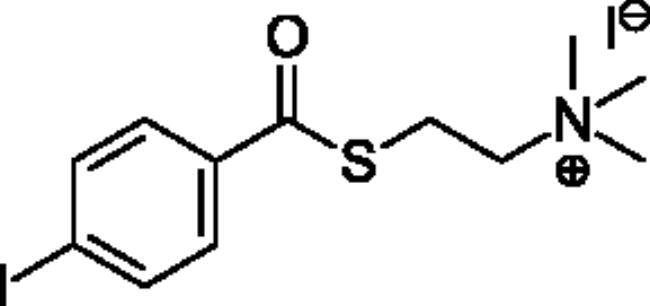	Not a substrate	Not a substrate	Not a substrate	2.60 ± 0.14	8.93 ± 0.55	29.2 ± 2.79
Alkyl* N*-Methylpiperidinyl Thioesters & Esters							
**7**	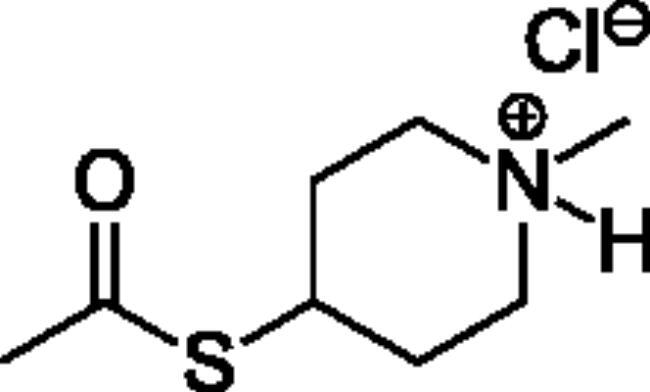	95.6 ± 17.1	2440 ± 492	3.93 ± 0.09	2.00 ± 1.08	1160 ± 740	0.179 ± 0.02
**8**	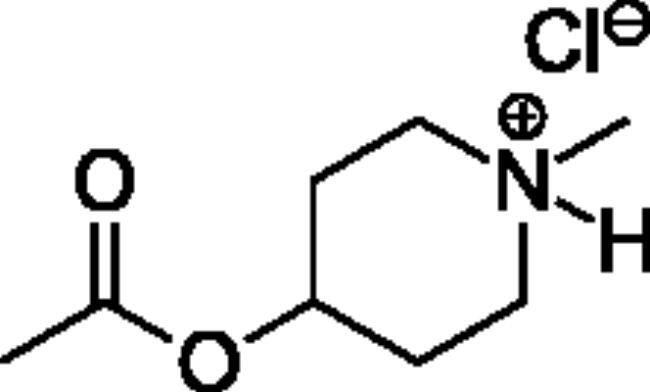	19.6 ± 14.4	3730 ± 2570	0.52 ± 0.05	65.4 ± 19.2	13000 ± 6700	0.545 ± 0.119
**9**	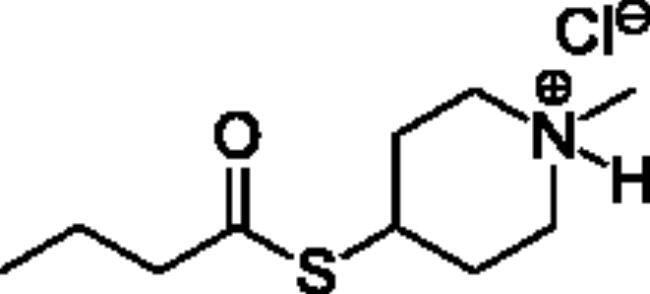	Not a substrate	Not a substrate	Not a substrate	10.4 ± 1.6	534 ± 85	1.95 ± 0.01
**10**	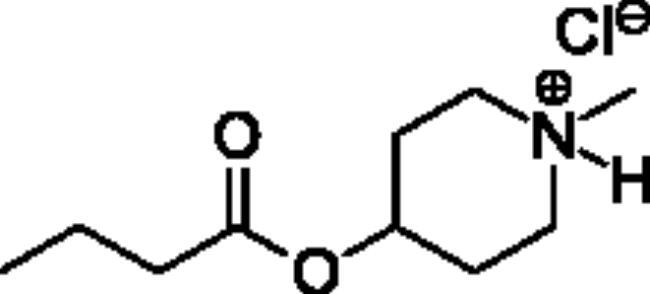	Not a substrate*	Not a substrate*	Not a substrate*	45.5 ± 23.4	11100 ± 4230	0.398 ± 0.083
Aryl *N*-Methylpiperidinyl Thioesters & Esters							
**11**	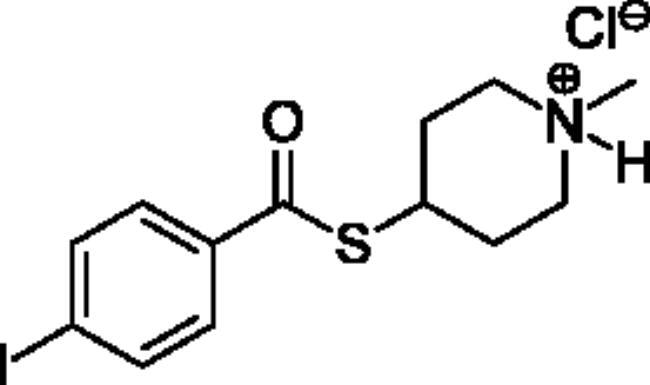	Not a substrate	Not a substrate	Not a substrate	0.212 ± 0.010	15.8 ± 1.3	1.34 ± 0.04
**12**	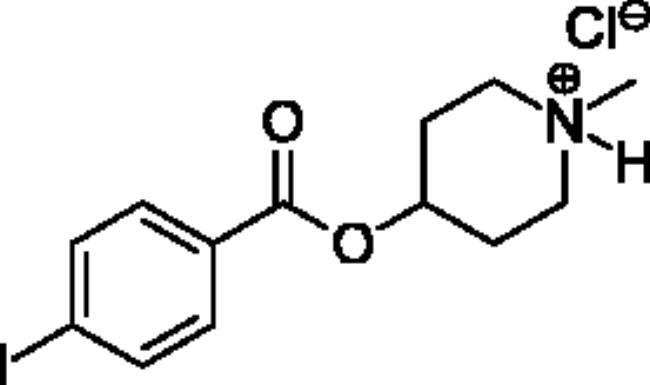	Not a substrate	Not a substrate	Not a substrate	0.0621 ± 0.0044	24.5 ± 3.3	0.256 ± 0.034
**13**	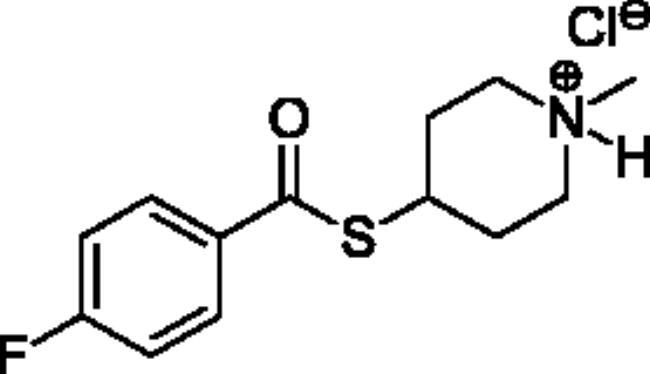	Not a substrate	Not a substrate	Not a substrate	0.629 ± 0.079	85.7 ± 18.5	0.745 ± 0.077
**14**	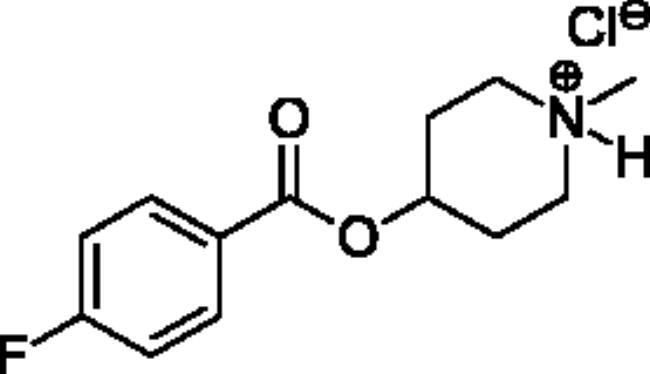	Not a substrate	Not a substrate	Not a substrate	19.0 ± 22.0	1120 ± 1360	1.81 ± 0.13
Alkyl* N*-Methylpyrrolidinyl Thioesters & Esters							
**15**	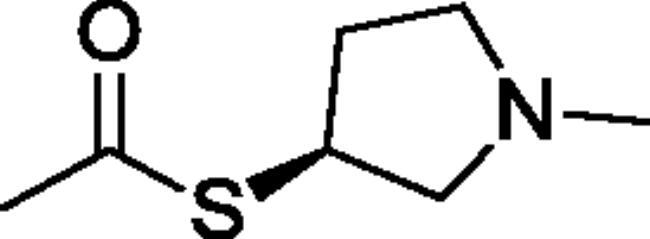	48.6 ± 2.8	218 ± 16	22.3 ± 0.4	0.432 ± 0.020	103 ± 10	0.421 ± 0.024
**16**	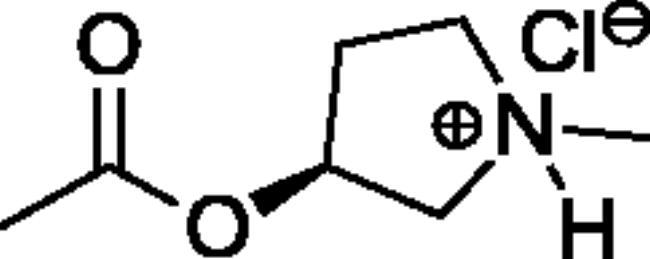	82.8 ± 51.6	4580 ± 1560	1.68 ± 0.61	24.9 ± 7.4	8020 ± 3900	0.327 ± 0.055
**17**	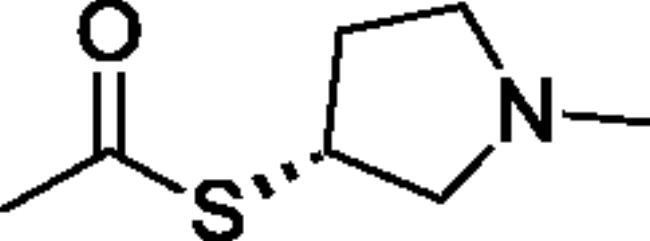	80.8 ± 11.4	947 ± 134	8.53 ± 0.23	1.23 ± 0.03	174 ± 4	0.704 ± 0.019
**18**	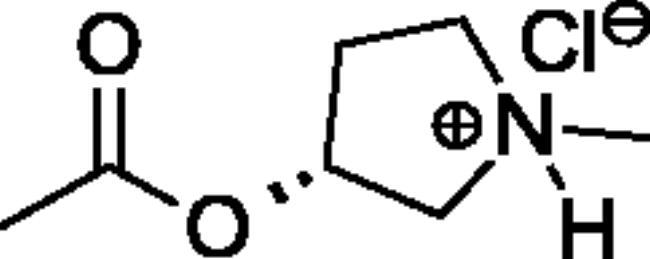	112 ± 52	4680 ± 2200	2.43 ± 0.79	31.0 ± 6.3	3770 ± 1540	0.877 ± 0.243
**19**	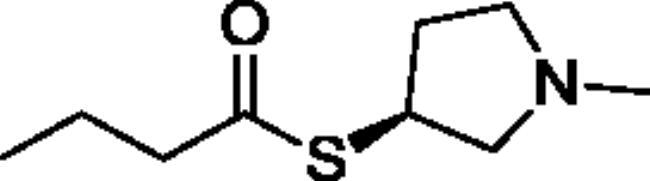	Not a substrate	Not a substrate	Not a substrate	6.63 ± 0.61	177 ± 27	3.78 ± 0.26
**20**	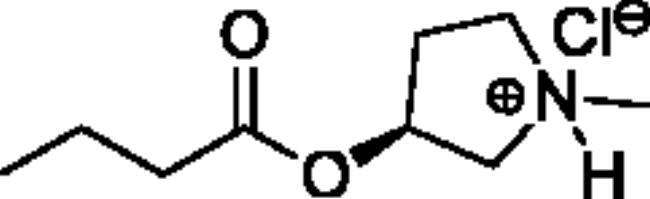	Not a substrate*	Not a substrate*	Not a substrate*	6.88 ± 0.52	912 ± 85	0.758 ± 0.088
**21**	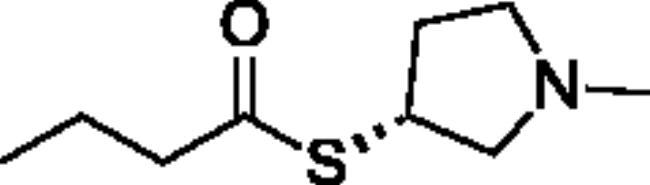	Not a substrate	Not a substrate	Not a substrate	6.63 ± 0.10	52.7 ± 1.7	12.6 ± 0.2
**22**	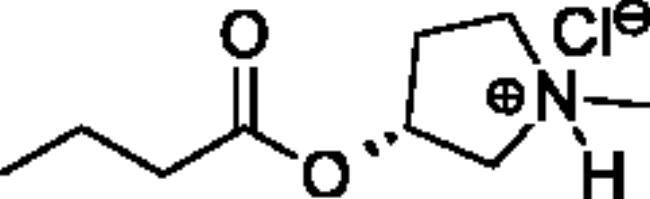	Not a substrate*	Not a substrate*	Not a substrate*	22.8 ± 13.0	1750 ± 774	1.24 ± 0.22
Aryl* N*-Methylpyrrolidinyl Thioesters & Esters							
**23**	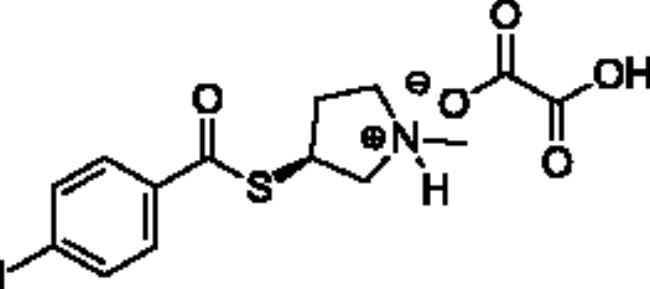	Not a substrate	Not a substrate	Not a substrate	7.91 ± 0.16	21.7 ± 1.0	36.5 ± 1.9
**24**	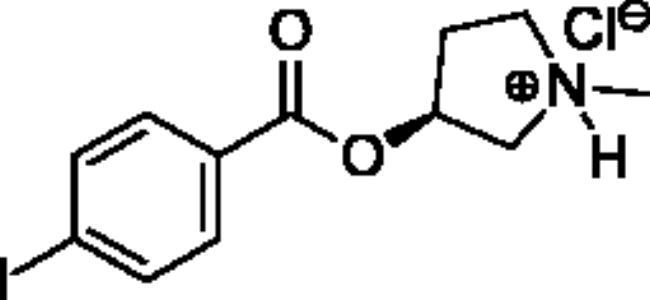	Not a substrate	Not a substrate	Not a substrate	0.706 ± 0.067	28.6 ± 5.1	2.49 ± 0.19
**25**	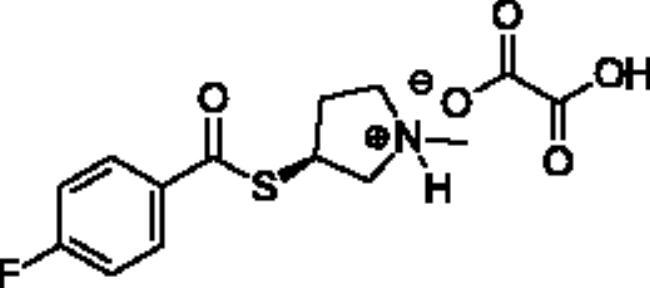	Not a substrate	Not a substrate	Not a substrate	8.96 ± 0.48	84.6 ± 7.3	10.6 ± 0.4
**26**	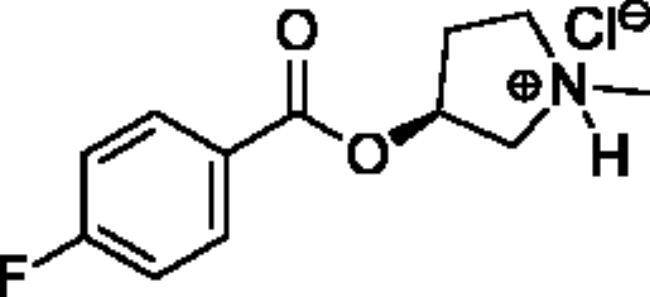	Not a substrate	Not a substrate	Not a substrate	13.2 ± 2.4	188 ± 47	7.12 ± 0.62
**27**	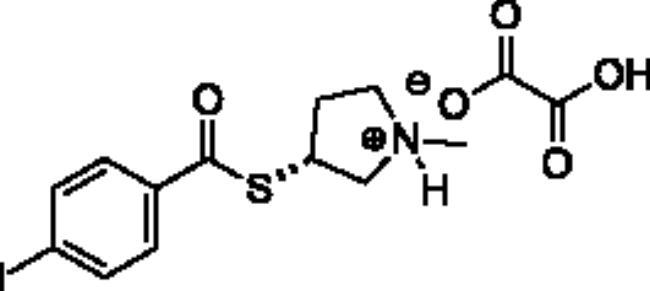	Not a substrate	Not a substrate	Not a substrate	6.42 ± 0.09	22.9 ± 1.8	28.1 ± 1.8
**28**	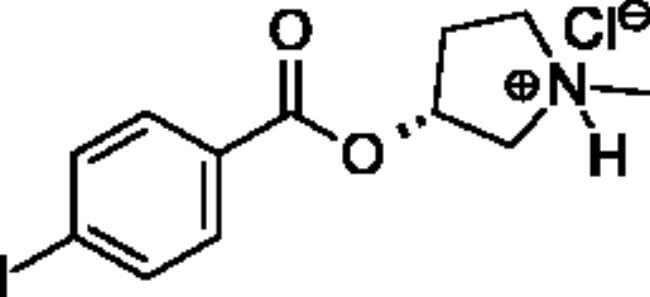	Not a substrate	Not a substrate	Not a substrate	8.50 ± 0.33	125 ± 6	6.79 ± 0.07
**29**	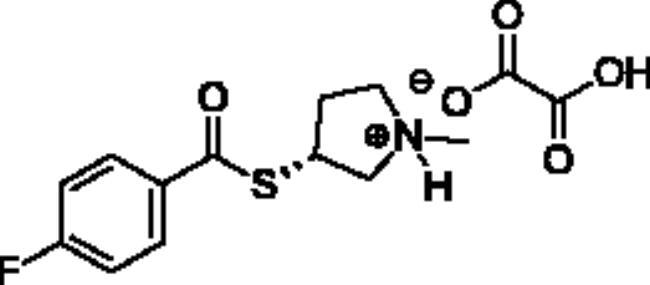	Not a substrate	Not a substrate	Not a substrate	4.43 ± 0.71	123 ± 28	3.63 ± 0.29
**30**	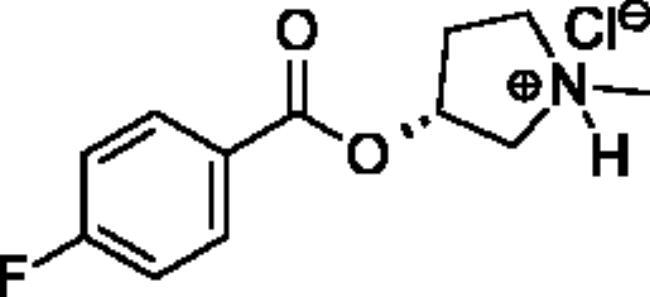	Not a substrate	Not a substrate	Not a substrate	133 ± 18	956 ± 138	14.0 ± 0.7

*To determine if a compound is a substrate, the *k*_cat_/*K_m_* value greater than or equal to that of the reporter molecule (2.62 × 10^6^ (min^−1^ M^−1^) is required, which these compounds did not exhibit. Since the corresponding thioesters are not substrates, we conclude that these compounds are also not substrates.

### Enzyme kinetics

To determine ChE specificity and whether compounds undergo hydrolysis with AChE or BChE, maximal absorbance changes were followed spectrophotometrically through repetitive wave scans and compared to the UV spectra of each compound and their hydrolysis products. To determine AChE and BChE specificity of compounds with a chromophore that absorbs strongly above 220 nm, the following was placed in a quartz cuvette (1 cm path length): potassium phosphate buffer (1.65 mL; 0.1 M; pH 7.0), 50 µL of either a stock BChE solution (1.5 pM in 0.01% gelatine_(aq)_) or stock AChE solution (3.7 pM in 0.01% gelatine_(aq)_), and 50 µL of 50% CH_3_CN_(aq)_. The cuvette was placed in the spectrophotometer and a background reading was collected. Compounds to be evaluated were dissolved in 50% CH_3_CN_(aq)_ at various concentrations (0.5–1 mM). In a clean quartz cuvette (1 cm path length) the following were added: potassium phosphate buffer (1.65 mL; 0.1 M; pH 7.0), 50 µL of either a stock BChE solution (1.5 pM in 0.01% gelatine_(aq)_) or stock AChE solution (3.7 pM in 0.01% gelatine_(aq)_), and 50 µL of 50% CH_3_CN_(aq)_ containing the compound to be evaluated. UV absorbance was scanned at room-temperature from 210–850 nm every 12 min for a total of 120 min using a VWR UV-1600PC spectrophotometer as previously described, with minor modifications^49^. Hydrolysis of the compounds was displayed as a spectral shift of starting materials to products. The wavelength corresponding to the maximal absorbance change during hydrolysis of each compound was used for the subsequent determination of BChE affinity and the kinetic parameters of *K*_m_ (substrate concentration at ½ maximum reaction rate, V_max_) and *k*_cat_ (turnover number; rate constant of substrate converted to products).

For determination of kinetic parameters of compounds containing a chromophore that absorbed strongly above 220 nm, the following was added to a quartz cuvette (1 cm path length): potassium phosphate buffer (1.72 mL; 0.1 M; pH 7.0), 30 µL of either a stock BChE solution (81 nM in 0.01% gelatine_(aq)_) or stock AChE solution (44.3 nM in 0.01% gelatine_(aq)_) and, 50 µL of 50% CH_3_CN_(aq)_. Cholinesterase stock solutions were more concentrated in this assay compared to the above to reduce the scan times needed in each experiment. The cuvette was placed in the spectrophotometer and a background reading was collected. Assessed compounds were dissolved in 50% CH_3_CN_(aq)_ at various concentrations (0.5–10 mM). In a clean quartz cuvette, the same additions were made as above but with 50 µL of compound solution in 50% CH_3_CN_(aq)_. When the compound was introduced to the enzyme, the reaction began. The cuvette was placed in the spectrophotometer and the absorbance was monitored over various timeframes (30 s to 5 min) depending on the rate of hydrolysis, at the wavelength determined above. Double-reciprocal Lineweaver-Burke plots were generated to give kinetic parameters, *K*_m_, V_max_, and *k*_cat,_ as described previously^54^. All assays were completed in triplicate to generate average kinetic parameters as shown in Table 1.

For esters that do not have a chromophore that absorbed strongly above 220 nm, competition studies were carried out to determine catalytic parameters as described in the literature^51–53^. In this method, a weak substrate in the visible range (Substrate A) with a known *k*_cat_/*K*_m_ value was used to determine the same parameters of an ‘invisible’ substrate (Substrate B) using the equation: R = (*k*_cat_/*K*_m_)_B_/(*k*_cat_/*K*_m_)_A_^51–53^. For these competition experiments, *ortho*-nitrofluoroacetanilide (*o-*NTFNAC; 3 mM in CH_3_CN; ε_430_ = 3954 M^−1^cm^−1^), a weak ChE substrate (BChE *k*_cat_/*K*_m_ = 2.5 x 10^6^ min^−1^M^−1^; AChE *k*_cat_/*K*_m_ = 2.6 x 10^6^ min^−1^M^−1^)^57^ was used. Kinetic analysis of BChE-catalyzed hydrolysis of *o-*NTFNAC was used as Substrate A. Briefly, 1.65 mL of 0.1 M potassium phosphate buffer (pH 7.0) was combined with 50 µL BChE (14 nM) or AChE (18 nM) in 0.1% gelatine_(aq)_ with 0.01% sodium azide, and 25 µL distilled water (dH_2_O) in a glass cuvette of 1 cm path length. To initiate the reaction, 25 µL of *o-*NTFNAC was added along with 50 µL of ester compound in 50% CH_3_CN_(aq)_ at various concentrations (0–500 mM) for a final cuvette volume of 1.80 mL. Absorbance was measured at 430 nm every 30 s for a total of 60 min with BChE, and every 60 s for a total of 180 min with AChE. Data were processed using PRISM 9.3.1 (471) software (GraphPad Dotmatics, Massachusetts, United States). A control experiment, in the absence of Substrate B, was used to determine the time needed for Substrate A to reach 90% hydrolysis (t90; 0.90 x plateau absorbance). This value was used to generate Δ_t90_ values that were plotted versus [B] to give V_maxB_. The *k*_catB_ value was calculated from maximal reaction rate as above, and *K*_mB_ was calculated using initial hydrolysis rates of the competition experiment in the presence (v^i^_0_) and absence (v_0_) of Substrate B. Ratios of v^i^_0_/v_0_ were plotted against [B] in PRISM and fit non-linearly to the regression: v^i^_0_/v_0_ = (1 + [A]/*K*_mA_)/(1 + [A]/*K*_mA_ + [B]/*K*_mB_)^52^^,^^53^. All experiments were performed in triplicate to generate average catalytic parameters summarised in Table 1.

### Brain tissues

Thioester and ester probes were evaluated in human and mouse brain tissues. Although the end goal is to develop diagnostic probes for AD, compounds were tested in mouse tissue as further evaluation of selected compounds will require pre-clinical SPECT or PET imaging in this species.

Post-mortem tissues from 6 AD brains (3 ♀ and 3 ♂; age range 68–98) and corresponding anonymized clinical and neuropathological information were provided by the Maritime Brain Tissue Bank (Halifax, Nova Scotia, Canada) following approval from the Nova Scotia Health Research Ethics Board. All cases fulfilled the neuropathological criteria for AD^58^. The brains were removed between 10–37.5 h after death and bisected at the midline. Half of the brain was used by a neuropathologist for diagnosis and half for histochemical studies. The latter halves were cut in 1 cm thick slabs, immersion fixed in 4% formalin in 0.1 M phosphate buffer pH 7.4 (PB) between 48 and 96 h, cryoprotected in a graded series of sucrose (10–40%) in 0.1 M PB and stored in PB with 40% sucrose and 0.6% sodium azide until use.

Formal approval to conduct these experiments with mouse tissue was obtained from the Dalhousie University Committee on Laboratory Animals (21–046). The 5XFAD (B6SJL-Tg (APPSwFlLon, PSEN1*M146L*L286V) 6799Vas/Mmjax, 034840-JAX) mice, described previously^59^, used in this study were produced from strains obtained from The Jackson Laboratory and the Mutant Mouse Resource & Research Centres. There were 26 mice (16 ♀ and 10 ♂) used in this study with an average age of 11.5 months. Mice were housed in same-sex groups of 1–5, within polyethylene cages, containing a wood-chip bedding and covered by a metal cage top and micro-isolator filter. Food (Purina rodent chow, #5001) and tap water were available *ad libitum*. Animals were kept in a normal light/dark cycle. An ear punch was used to identify mice and collect a skin sample for genotyping. Genotyping of mice was accomplished as described previously^60^. Animals were euthanized using a lethal intraperitoneal injection of 0.06 mL sodium phenobarbital (340 mg/mL) and 0.06 mL lidocaine (20 mg/mL), followed by transcardial perfusion with 0.9% saline solution and 0.1% sodium nitrite. Tissues were fixed by perfusion with 4% paraformaldehyde in 0.1 M PB (pH 7.4). Brains were removed and post-fixed for 2 h, then stored at 4 °C in a 30% sucrose solution with 0.05% sodium azide until sectioned.

Brain regions with either significant Aβ plaque load or specific distributions of AChE and BChE associated with neural elements (i.e. neurons, neuropil, axons) were chosen to examine the interaction of thioesters and esters with brain tissue ChEs. From human brains, blocks of tissue containing the orbitofrontal cortex and the thalamus were chosen to examine the interaction of esters and thioesters with ChEs associated with Aβ plaques^28^ and neural elements^19^, respectively. In mouse brain tissues, the cerebral cortex and midbrain were used to evaluate the interaction of probes with ChEs associated with Aβ plaques^60^ and neural elements^47^, respectively.

Mouse brains and blocks of human brain tissues were frozen with dry ice and cut on a Leica SM2000R microtome with a Physitemp freezing stage in 40 or 50 μm thick sections, respectively. Until used for histochemical staining experiments, sections of mouse brain tissue were stored in PB with 0.05% sodium azide at 4 °C while those from human brain were stored in PB with 40% sucrose and 0.6% sodium azide at −20 °C.

### Amyloid-β immunohistochemistry

To confirm the presence of Aβ plaques in the 5XFAD mouse and human AD brains, tissue sections underwent immunohistochemical staining for the Aβ protein. Standard immunohistochemical techniques were performed using a primary antibody for polyclonal rabbit anti-amyloid (1:400; 71–5800, Invitrogen, Rockford, Illinois, United States), specific for the 4- to 5-kDa amyloid peptide, as previously described^28^^,^^60^. In control experiments, no staining was observed when the primary antibody was omitted from the reaction.

### Histochemical studies

A modified^60^ Karnovsky-Roots^50^ method was used for histochemical staining of AChE and BChE activity in 5XFAD mouse and human AD brain tissues, as previously described. Unless otherwise specified, KR methodologies remained the same for both human and mouse brain tissues. Tissue sections were rinsed in 0.1 M PB (pH 7.4) for 30 min, incubated for 30 min in 0.1 M PB (pH 7.4) containing 0.15% or 0.3% hydrogen peroxide (H_2_O_2_) for human or mouse tissue, respectively, and then rinsed with 0.1 M maleate buffer (MB; pH 6.8 or 7.4) for 30 min. When staining for AChE, mouse and human sections were incubated for 1 and 1.75 h, respectively, in the KR staining solution. When staining for BChE and thioesters, mouse and human sections were incubated in the KR staining solution for 2 and 2.5 h, respectively. Incubation times for each ChE and tissue type were chosen based on known properties of AChE and BChE^26^ and the types of tissue stained. In the brain, there is more AChE than BChE activity^26^. To accommodate for this increased activity and to prevent overstaining, a reduced incubation time is then required for optimal visualisation of AChE distribution in brain tissues. Alternatively, the longer incubation time for BChE reactions allows for better visualisation of BChE staining distributions. The KR staining solution contained 0.5 mM sodium citrate, 0.47 mM cupric sulphate, and 0.05 mM of potassium ferricyanide, a thioester ChE substrate and inhibitor in MB at pH 6.8 or 8.0. The ChE substrate and inhibitor added to specifically stain for AChE activity was ATChI (**1**) and 0.06 mM ethopropazine, respectively. For BChE activity, 0.8 mM BTChI (**3**) and 0.01 mM BW 284c51 were added. Staining at pH 6.8 allowed for the visualisation of ChE activity associated with Aβ plaques, while pH 8.0 allowed for the optimal visualisation of ChEs associated with normal neural elements^24^^,^^26^. This is based on previous studies that have shown that the normal neural distribution of ChE activity in brain tissues is optimally visualised using the KR histochemical reaction at slightly alkaline conditions (pH 8.0), while ChE activity associated with AD plaques is optimally visualised using slightly acidic conditions (pH 6.8)^24^^,^^26^. Following incubation, sections were rinsed in dH_2_O for 30 min, incubated in 0.1% cobalt chloride in dH_2_O for 10 min, followed by another 30 min rinse in dH_2_O. Tissue sections were then placed in 1.39 mM DAB in PB for 5 min. The chromogenic effect of DAB was developed by the addition of 50 µL of 0.3% H_2_O_2_ in dH_2_O per mL of DAB solution. The reaction was stopped by rinsing the sections in 0.01 M acetate buffer (pH 3.3) for 30 min. Sections were mounted on glass slides, cover-slipped, and examined with brightfield microscopy. All staining experiments were duplicated to confirm staining patterns.

**Figure 1.**
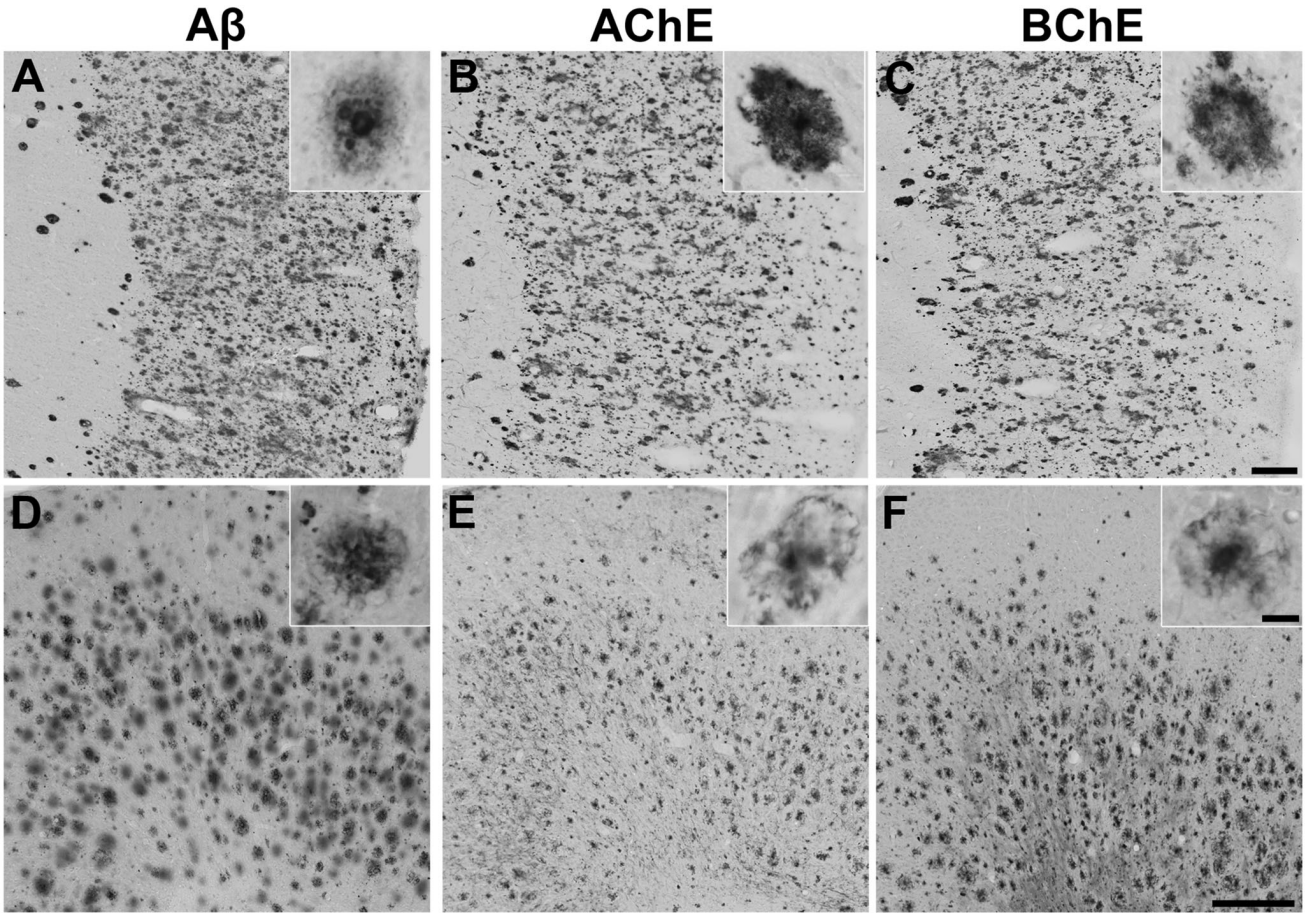
Representative photomicrographs showing staining of plaque pathology for Aβ immunohistochemistry (A, D) and AChE (B, E) and BChE (C, F) histochemistry at pH 6.8 in the cerebral cortex of human AD (A-C) and 5XFAD mouse (D-F) brain tissues. Insets are higher magnification photomicrographs showing examples of plaque pathology. Note the similarities in the cortical plaques stained for Aβ, AChE, and BChE. Scale bars = 200 µm, insets = 10 µm.

Post-mortem interval and the duration of fixation can affect KR histochemical staining of AChE and BChE activity in brain tissues. Prior to carrying out histochemical analysis of the thioesters and esters, brain tissues from each human AD and mouse case were evaluated for the quality of ChE staining using traditional ChE substrates. Mouse brain tissues typically demonstrated better ChE staining presumably due to the rapid and relatively short fixation obtained via perfusion rather than the longer immersion fixation that is used for human tissue. This resulted in shorter staining periods for mouse brain tissues compared to that in humans. Regardless, mouse and human cases selected for this study showed similar ChE staining patterns and characteristics^26^^,^^60^ as demonstrated in Figure 1.

The procedure for evaluating the ability of synthesised thioesters to interact with ChEs in brain tissues was the same as above with the omission of the traditional ChE substrates, ATChI (**1**) or BTChI (**3**), and inhibitors, ethopropazine and BW 284c51, from the KR staining solution. Thioesters **1**, **3**, **5**, **7**, **9**, **13**, **15**, **17**, **19**, **21**, **25**, and **29** were individually added at a concentration of 1 mM. Due to lower solubility, thioesters **6**, **23**, and **27** were used at 0.4 mM and **11** at 0.2 mM. Following tissue incubation in the KR solution containing thioesters, staining was visualised with cobalt chloride and DAB, as described above.

Ester compounds could not be evaluated directly with the KR histochemical method as thioesters were. Therefore, esters were employed as competitors of the traditionally used substrates of AChE and BChE, ATChI (**1**), and BTChI (**3**), respectively. Esters that interacted with and were hydrolysed by AChE or BChE would proportionally decrease the hydrolysis of ATChI (**1**) and BTChI (**3**), thereby decreasing staining intensity. Staining for AChE or BChE activity was done as described above. All esters were initially added to the KR solution at 1 mM. If an ester failed to reduce the staining intensity of AChE or BChE, the concentration was increased to 5 mM. Esters **12**, **24**, **26**, **28**, and **30** were tested at 1 mM while **2**, **4**, **8**, **10**, **14**, **16**, **18**, **20**, and **22** were evaluated at 5 mM. Following incubation with an ester in the KR staining solution, tissue staining was completed as described above. For comparative purposes, positive control sections were stained for AChE and BChE activity at pH 6.8 and 8.0 without any additions. To ensure specificity of AChE and BChE staining, control experiments whereby the ATChI and BTChI substrates were omitted were performed at pH 6.8 and 8.0 in human and mouse brain tissues. No staining was observed when the substrate was omitted.

### Analysis of histochemistry

**Figure 2.**
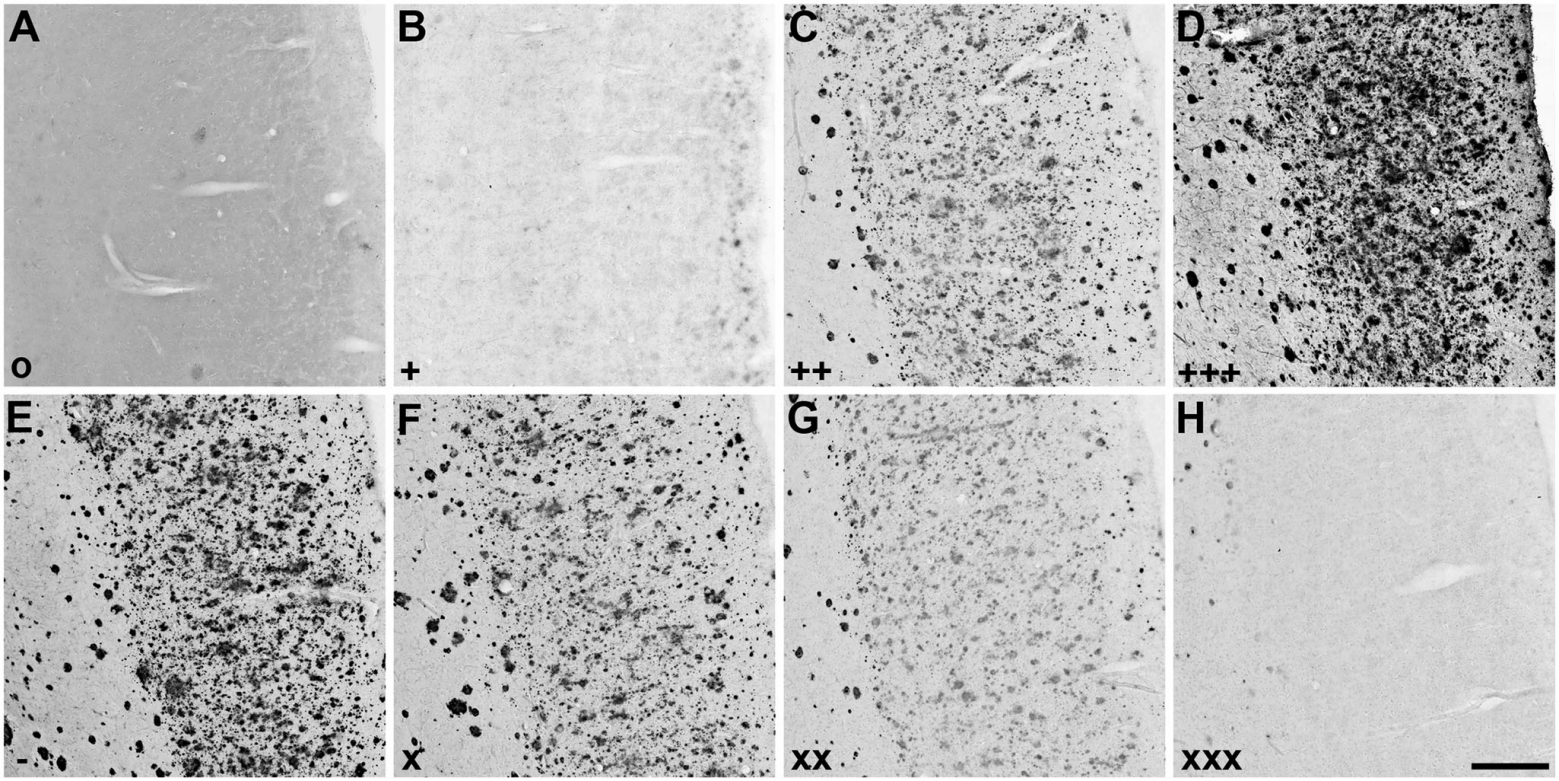
Representative photomicrographs of histochemical staining at pH 6.8 in human orbitofrontal cortex sections. The sections were from the same brain to illustrate staining patterns demonstrating the intensity of ChE staining of AD pathology using thioesters (A-D) and the reduction of staining intensity for BChE at pH 6.8 by ester compounds (E-H). Thioester staining intensity, summarised in Table 2, was categorised as (A) o (no staining), (B) + (weak), (C) ++ (moderate), or (D) +++ (strong). The reduction in staining by esters, also summarised in Table 2, was categorised as (E) – (no inhibition), (F) x (slight), (G) xx (moderate) or (H) xxx (strong). Scale bar = 400 µm.

**Figure 3.**
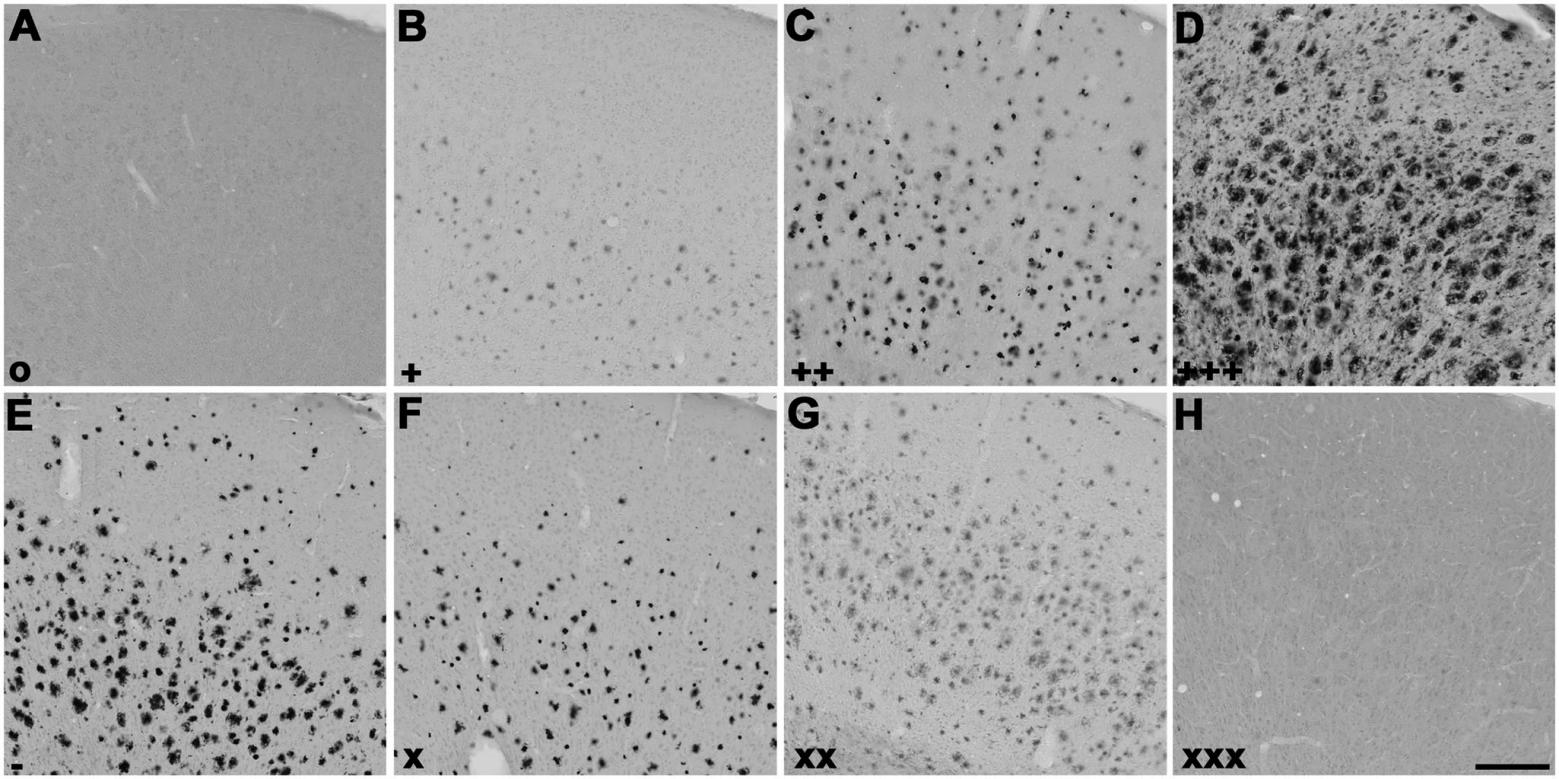
Representative photomicrographs of histochemical staining at pH 6.8 in 5XFAD mouse cerebral cortex sections demonstrating the intensity of ChE staining of AD pathology using thioesters (A-D) and the reduction of staining intensity for BChE at pH 6.8 by ester compounds (E-H). Thioester staining intensity, summarised in Table 2, was categorised as (A) o (no staining), (B) + (weak), (C) ++ (moderate), or (D) +++ (strong). The reduction in staining by esters, also summarised in Table 2, was categorised as (E) – (no inhibition), (F) x (slight), (G) xx (moderate) or (H) xxx (strong). Scale bar = 200 µm.

A qualitative method was used to categorise and tabulate the interaction of thioesters and esters with human (Figure 2) and mouse (Figure 3) brain tissue ChEs as shown using the KR histochemical method. For thioesters, the staining intensity was categorised as: o (no staining), + (weak), ++ (moderate), or +++ (strong). For esters, the reduction in AChE and BChE staining intensity was categorised as: - (no reduction), x (slight), xx (moderate), or xxx (strong). All tissue sections were analysed independently by two observers using brightfield microscopy on an Olympus BX50 microscope. Any discrepancies were jointly discussed until a consensus was reached. Stained tissue sections were photographed with a Zeiss Axio Scan.Z1 slide scanner with Zen 3.1 Blue Edition software (Carl Zeiss Canada Ltd, Toronto, Ontario, Canada). The photomicrographs were assembled into figures using Adobe Photoshop (CS 5, Version 12.0, San Diego, California, United States). The brightness of the photographs was adjusted to match the background of each image.

## Results

### Enzyme kinetics

The specificity and catalytic parameters (*k*_cat_, V_max_, *K*_m_, and *k*_cat_/*K*_m_) of the thioester and ester derivatives examined (**5**–**30**) are summarised in Table 1. All compounds were found to be substrates for either AChE, BChE, or both. Generally, thioester compounds showed slightly higher cholinesterase affinity (*K_m_* values) than their ester counterparts (Table 1). Compounds with longer carbon chains or groups bulkier than an acetyl group were not substrates for AChE, likely because the active site gorge of AChE has a smaller volume than that of BChE^61^.

ATChI (**1**) and ACh (**2**) were excellent substrates for both AChE and BChE. BTChI (**3**) and BCh (**4**), as well as *p*-fluoro (**5**) and *p*-iodo (**6**) choline esters were specific substrates for BChE. In alkyl *N*-methyl piperidinyl series, the acetyl thioester (**7**) and ester (**8**) were substrates for AChE and BChE, while the butyryl thioester (**9**) and ester (**10**) were specific for BChE. All aryl *N*-methyl piperidinyl thioesters and esters (**11–14**) were specific substrates for BChE. In the alkyl *N*-methyl pyrrolidinyl series, both *R* and *S* enantiomers of the thioesters and esters (**15–18**) were substrates for AChE and BChE. On the other hand, the butyryl thioesters and esters (**19–22**) were specific substrates for BChE. All aryl *N*-methyl pyrrolidinyl thioesters and esters (**23–30**) were specific substrates for BChE. Spectrophotometric data for **7**, **9**, **11**, **12**, **13** were comparable to those described previously^49^. Comparison of the *R* and *S* enantiomers of the alkyl and aryl *N* methyl piperidines and pyrrolidines showed enantiomeric effect for most pairs. Compound **25** (*S*) had both a higher turnover number (*k*_cat_ value) and BChE affinity compared to compound **29** (*R*). Compound **20** (*S*) had higher BChE affinity, but compound **22** (*R*) had a higher turnover number. Compound **24** (*S*) had higher BChE affinity while compound **28** (*R*) had a higher turnover number. Compound **26** (*S*) had higher BChE affinity while compound **30** (*R*) had a higher turnover number. Compounds **23** (S) and **27** (*R*) did not show any enantiomeric effect. These enantiomer effects were not seen in histochemical staining.

### Plaque deposition

In the brains of 5XFAD mice and human AD cases chosen for this study, there was robust deposition of Aβ plaques in the cerebral cortex, and AChE and BChE activities were associated with many of these plaques (Figure 1). Plaques stained for Aβ, AChE, and BChE showed similar staining characteristics. As this study primarily focussed on staining intensity or inhibition of AD plaques by thioester and ester substrates in subsequent experiments, plaque morphology was not investigated at this time.

### Histochemical staining with thioester derivatives

**Figure 4.**
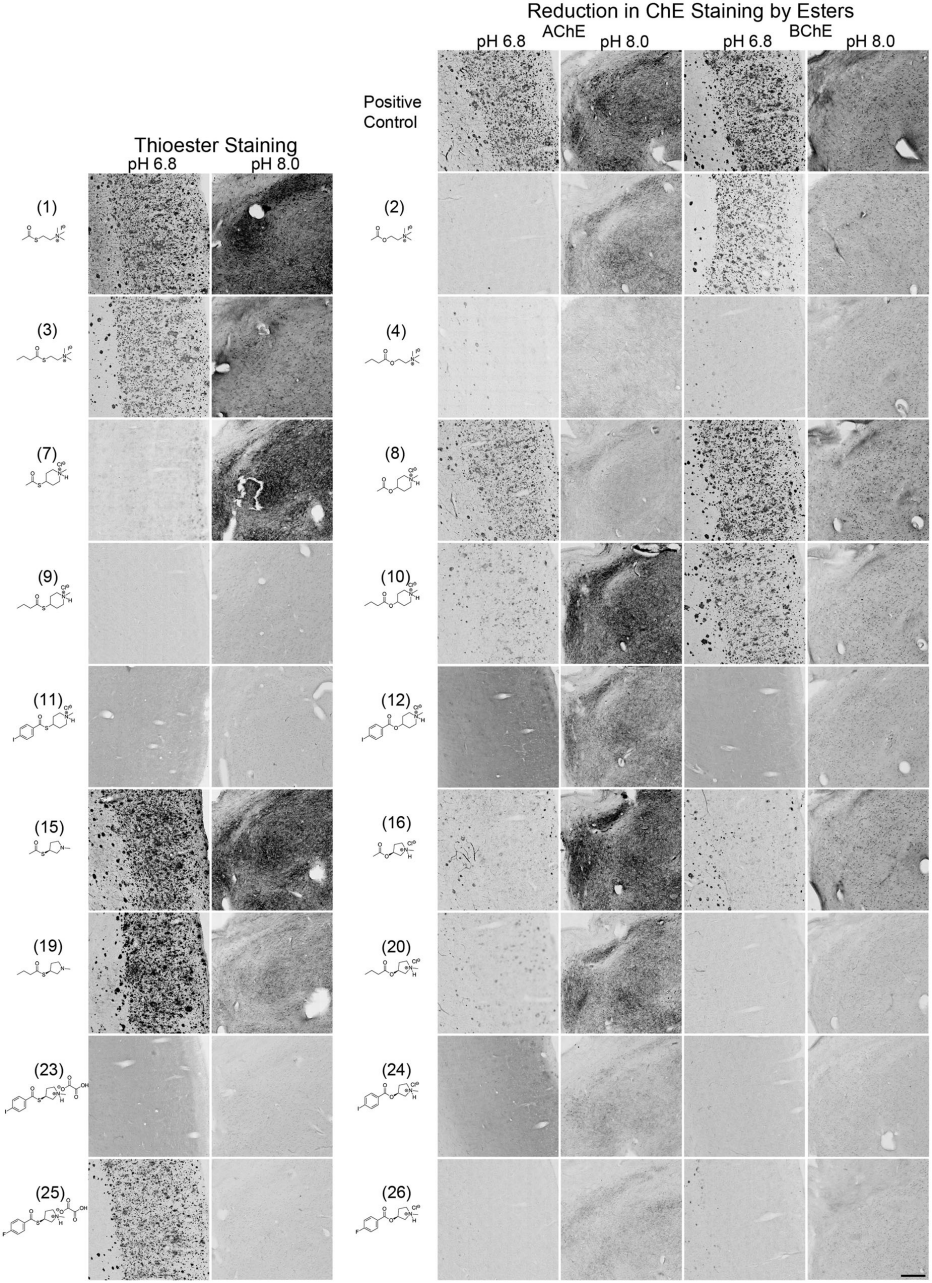
Photomicrographs of histochemical staining in human brain tissues demonstrating the hydrolysis of thioesters by ChEs at pH 6.8 or 8.0 (columns 1 and 2) or the reduction in AChE and BChE staining in the presence of esters (columns 3–6). Note, positive control staining of tissue (i.e. AChE or BChE staining at pH 6.8 and 8.0) is provided in the 1^st^ row of columns 3–6. Note, histochemical staining at pH 6.8 demonstrated ChE activity associated with Aβ plaques while that at pH 8.0 is associated with neural elements. Staining with thioester compounds allowed for a direct visualisation of the kinetic interaction of the compound with ChEs. The degree to which staining for AChE or BChE activity was reduced by the presence of esters provided indirect evidence that the compound interacted with the enzyme targets. Scale bar = 400 µm.

**Figure 5.**
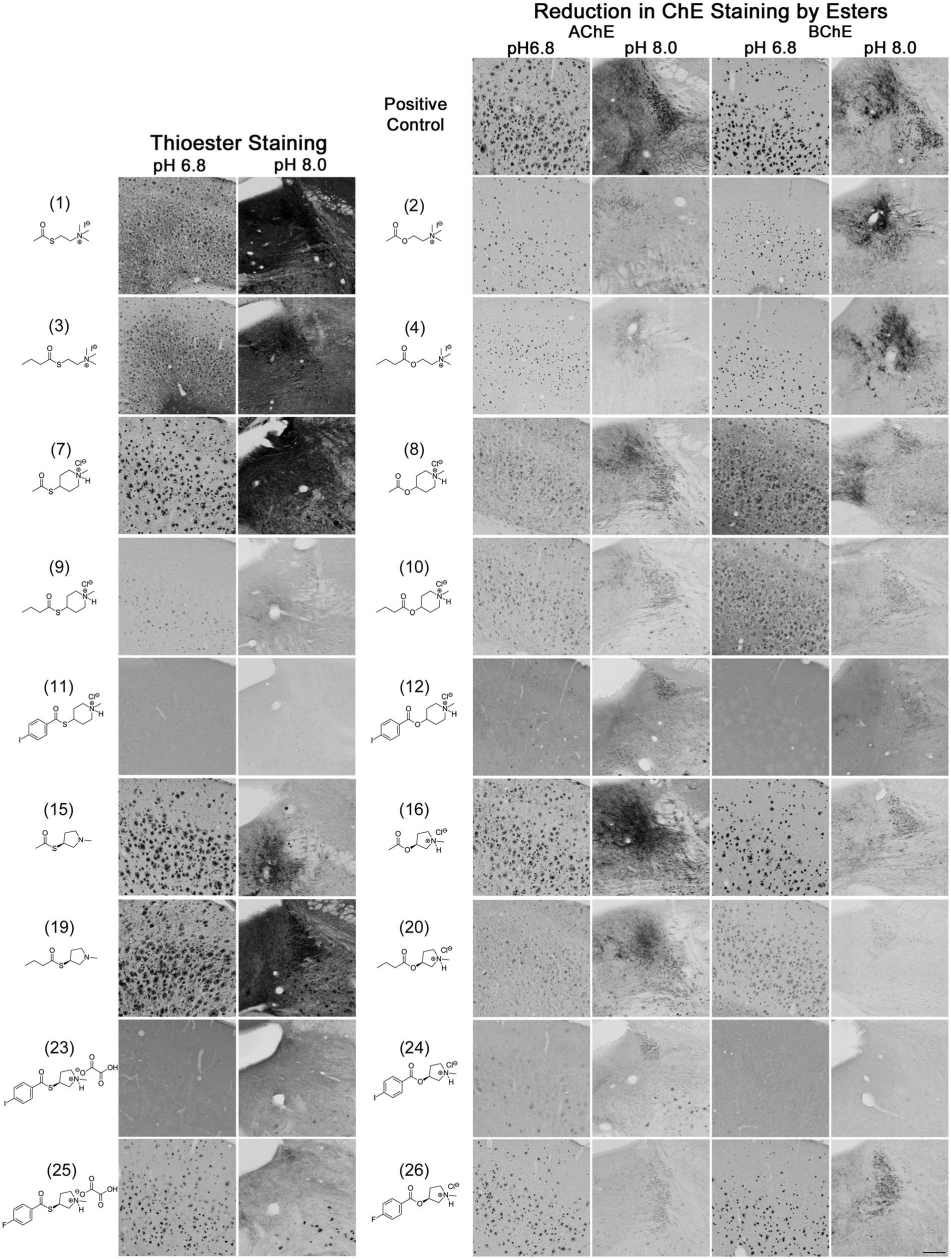
Photomicrographs of histochemical staining in mouse brain tissues demonstrating the hydrolysis of thioesters by ChEs at pH 6.8 or 8.0 (columns 1 and 2) or the reduction in AChE and BChE staining in the presence of esters (columns 3–6). Note, positive control staining of tissue (i.e. AChE or BChE staining at pH 6.8 and 8.0) is provided in the 1^st^ row of columns 3–6. Note, histochemical staining at pH 6.8 demonstrated ChE activity associated with Aβ plaques while that at pH 8.0 is associated with neural elements. Staining with thioester compounds allowed for a direct visualisation of the kinetic interaction of the compound with ChEs. The degree to which staining for AChE or BChE activity was reduced by the presence of esters provided indirect evidence that the compound interacted with the enzyme targets. Scale bar = 200 µm.

The use of thioester compounds in the KR histochemical method resulted in the staining of Aβ plaques (pH 6.8) and neural elements (pH 8.0) in human and mouse brain tissues with intensities that varied from no staining (o) to strong staining (+++) (Figures 2,3). The qualitative analysis of thioester staining in human (round brackets) and mouse [square brackets] brain tissue is provided in Table 2. Photomicrographs from representative thioesters are shown in Figure 4 for human and Figure 5 for mouse brain tissue. Mouse brain tissues were cut into 40 μm serial sections to maximise the number of sections obtained per brain, while human brain tissues we cut into 50 μm-thick sections. The size and thickness of tissue sections can have an effect on how fast the components of the KR staining protocol can penetrate the tissue. To mitigate this factor, we have employed shorter incubation times for mouse tissue sections (1–2 h) than for human tissue sections (1.75–2.5 h). As the staining characteristics noted for AChE and BChE were similar between mouse and human brain tissues, the difference in the thickness of tissues was negligible when using a qualitative method to evaluate the ChE staining.

**Table 2.** Qualitative analysis of the thioester and ester staining results with the Karnovksy-Roots (KR) histochemical staining method in human (round brackets) and mouse [square brackets] brain tissues. Intensity of thioester staining with the KR method was categorised as: o (no staining), + (weak), ++ (moderate) or +++ (strong); as shown in Figures 2 and 3. The inhibition in AChE or BChE staining resulting from ester substrate competition was categorised as: – (no inhibition), x (slight), xx (moderate) or xxx (strong); as depicted in Figures 2 and 3. ND = not done.

Structure (Number)	Intensity of Thioester Staining		Structure (Number)	Reduction in Staining by Esters			
Thioester	pH 6.8	pH 8.0	Ester	AChE pH 6.8	AChE pH 8.0	BChE 6.8	BChE 8.0
**Choline Thioesters & Esters**							
**(1)** 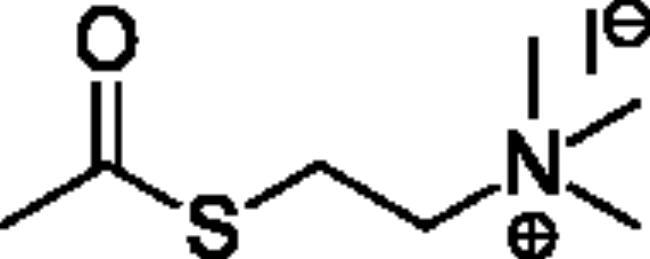	(+++) [+++]	(+++) [+++]	(**2)** 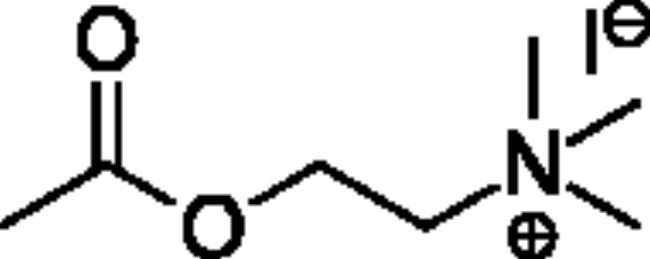	(xxx) [x]	(xx) [xx]	(x) [x]	(x) [x]
(**3)** 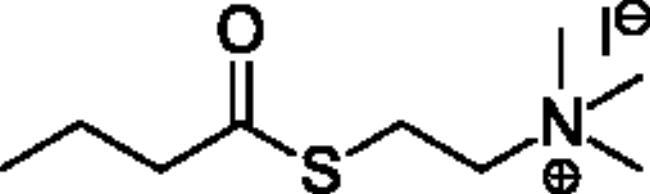	(+++) [+++]	(+++) [+++]	**(4)** 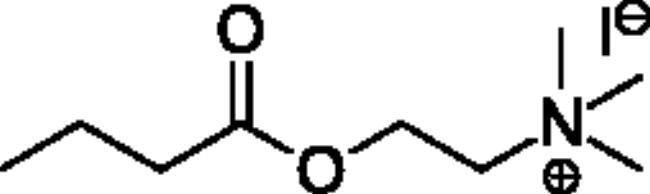	(xxx) [xx]	(xxx) [xxx]	(xxx) [xx]	(xx) [xx]
**(5)** 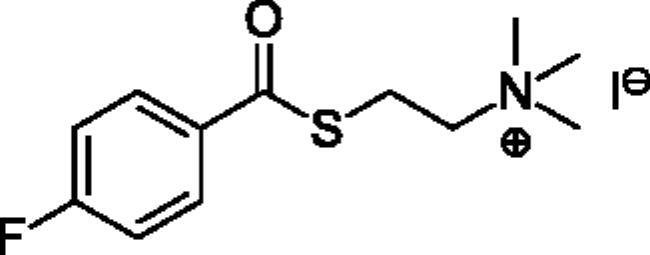	(o) [+]	(o) [+]	ND	ND	ND	ND	ND
(**6)** 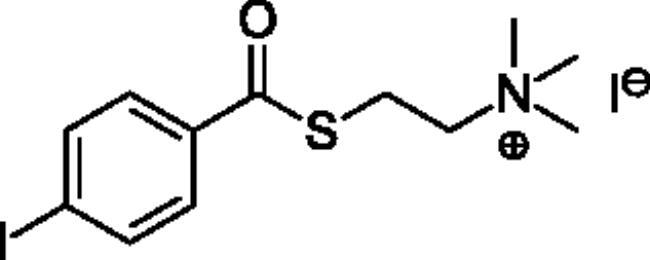	(o) [+]	(o) [+]	ND	ND	ND	ND	ND
**Alkyl *N*-Methylpiperidine Thioesters & Esters**							
(**7)** 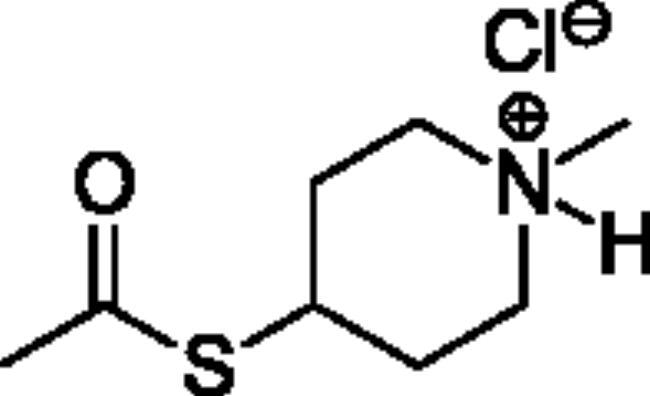	(+) [+++]	(+++) [+++]	(**8**) 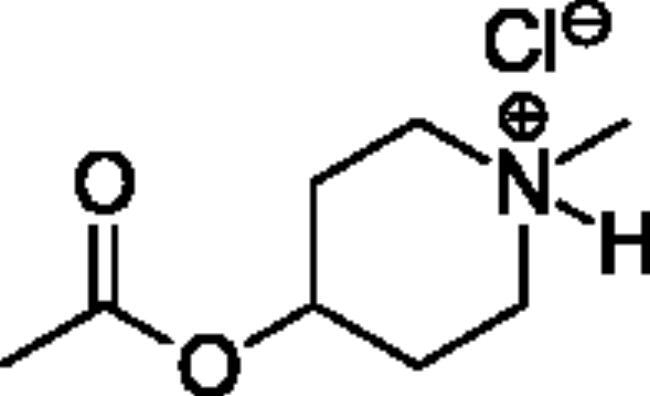	(x) [xx]	(xx) [xx]	(−) [−]	(−) [−]
(**9)** 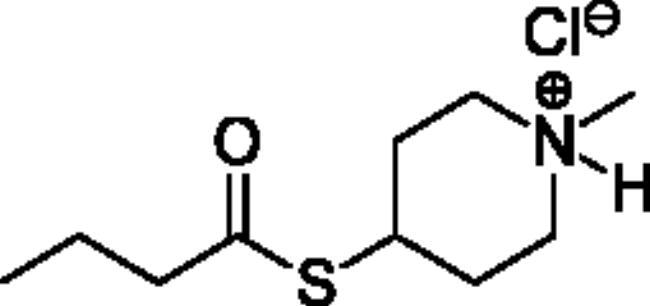	(o) [+]	(o) [+]	(**10)** 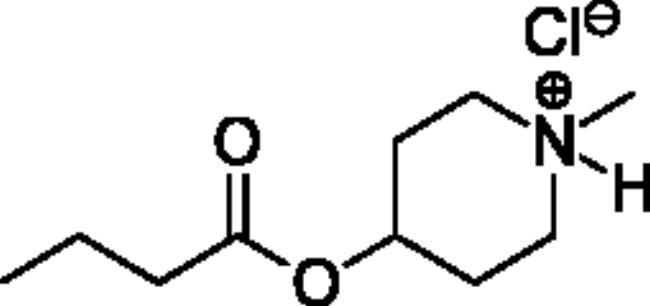	(xx) [xx]	(x) [xx]	(x) [x]	(x) [xx]
**Aryl *N*-Methylpiperidine Thioesters & Esters**							
(**11)** 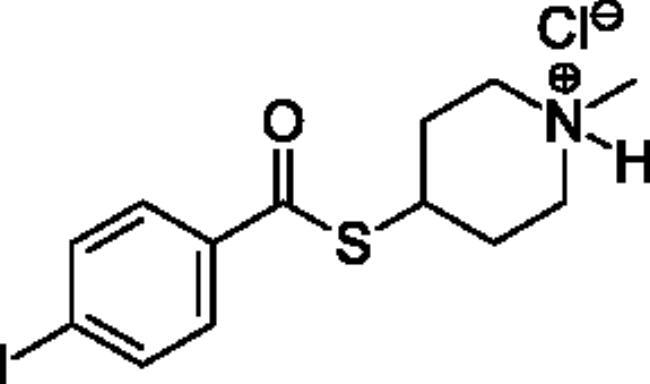	(o) [o]	(o) [o]	(**12)** 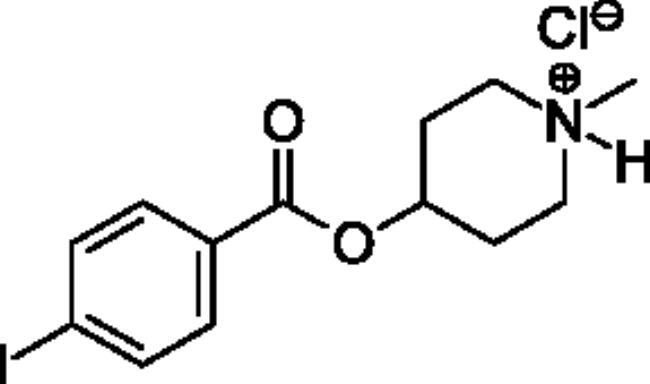	(xxx) [xxx]	(xx) [xxx]	(xxx) [xxx]	(xxx) [xxx]
(**13)** 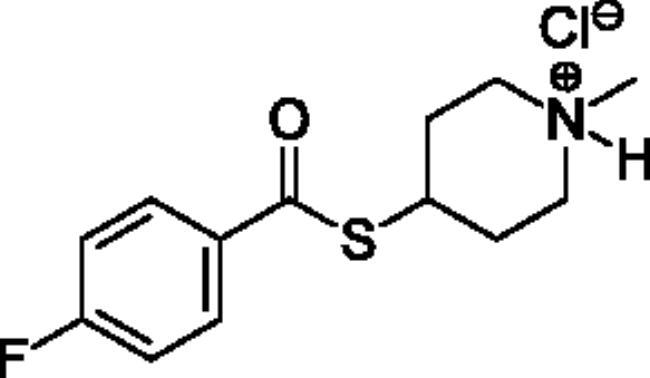	(o) [o]	(o) [+]	(**14**) 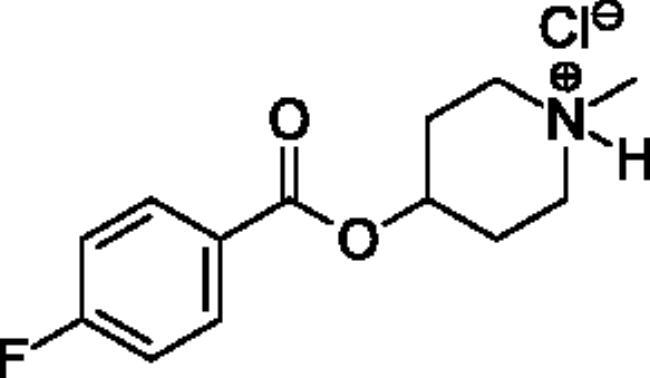	(xxx) [xxx]	(xx) [xxx]	(xxx) [xxx]	(xx) [xx]
**Alkyl *N*-Methylpyrrolidine Thioesters & Esters**							
(**15)** 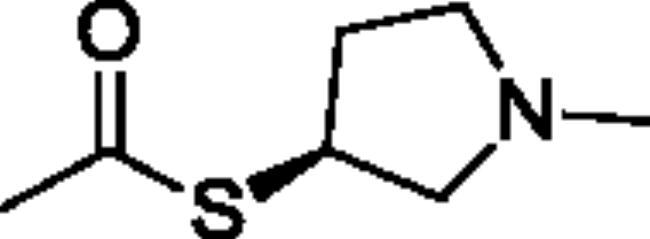	(+++) [+++]	(+++) [+++]	(**16)** 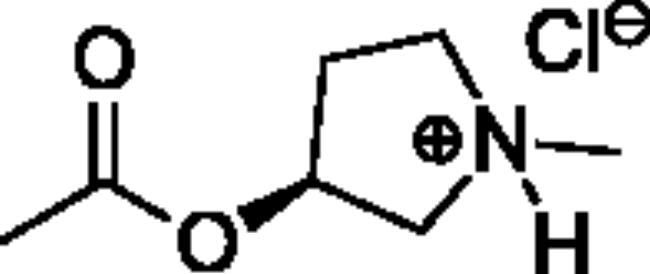	(xx) [−]	(−) [−]	(xx) [x]	(−) [x]
(**17)** 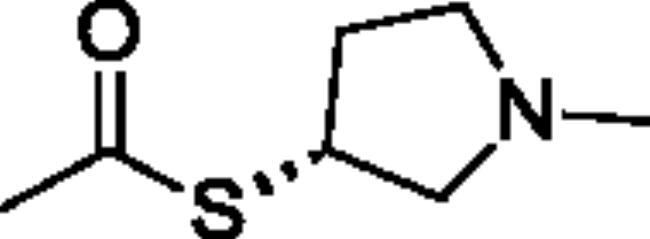	(+++) [+++]	(+++) [+++]	(**18)** 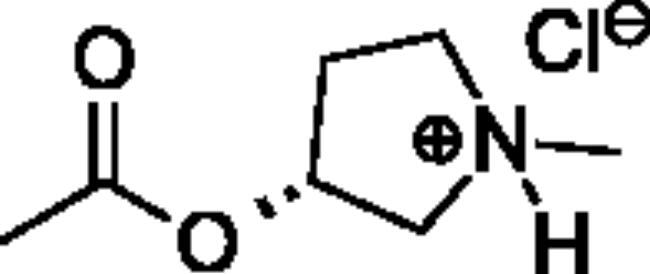	(xx) [x]	(x) [x]	(x) [x]	(x) [−]
(**19)** 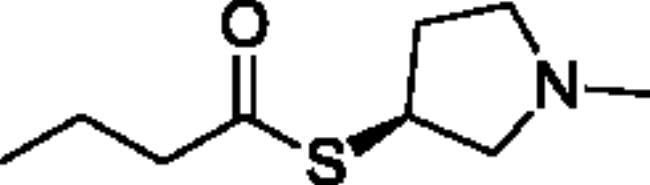	(+++) [+++]	(++) [+++]	(**20**) 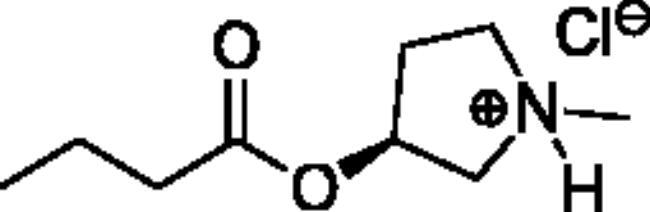	(xxx) [xx]	(xx) [xx]	(xxx) [xx]	(xxx) [xxx]
(**21**) 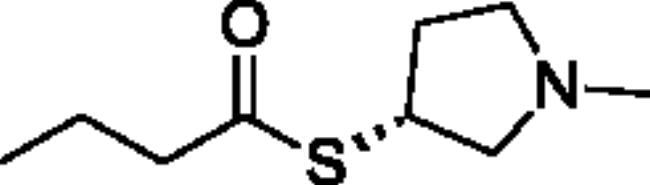	(+++) [++]	(++) [++]	(**22)** 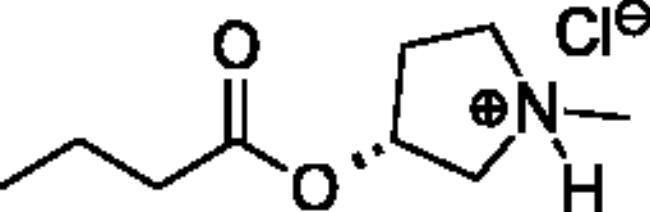	(xx) [xx]	(xx) [xx]	(xx) [x]	(xx) [x]
**Aryl *N*-Methylpyrrolidine Thioesters & Esters**							
(**23)** 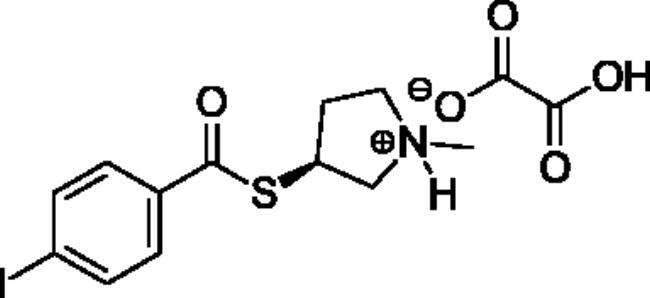	(o) [o]	(o) [+]	(**24**) 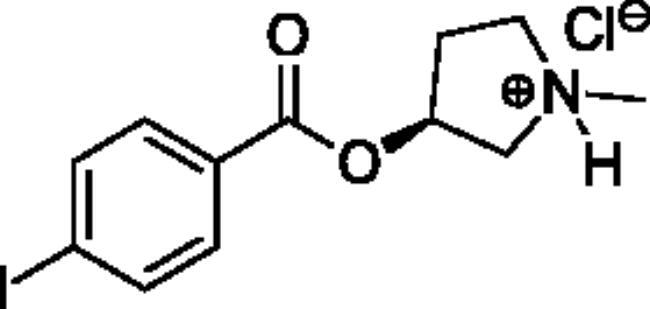	(xxx) [xxx]	(xxx) [xxx]	(xxx) [xxx]	(xxx) [xxx]
(**25)** 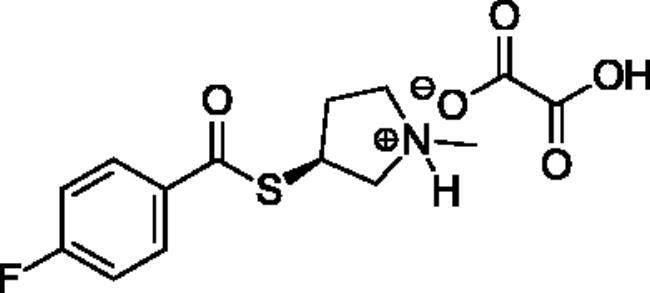	(+++) [++]	(o) [++]	(**26**) 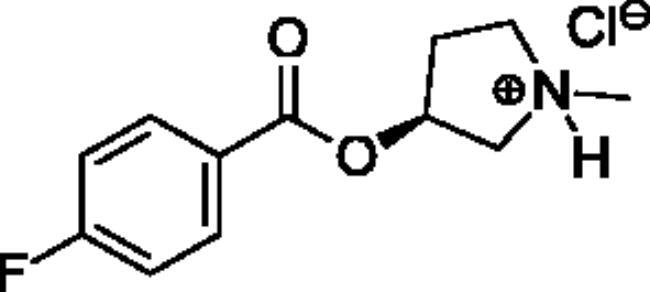	(xxx) [xx]	(xxx) [xxx]	(xxx) [x]	(xxx) [x]
(**27)** 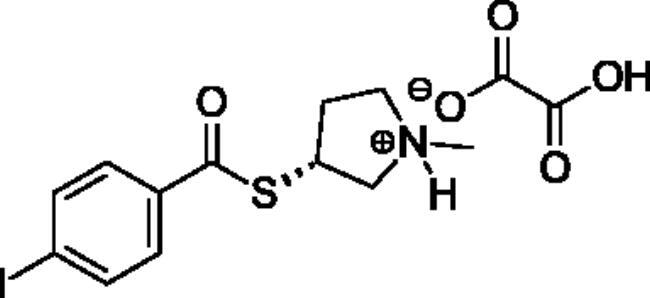	(o) [o]	(o) [+]	(**28**) 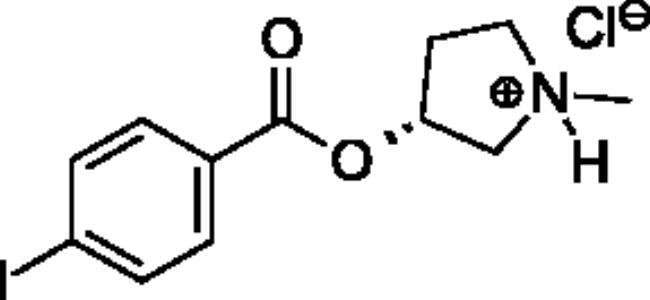	(xxx) [xx]	(xx) [xx]	(xxx) [xxx]	(xxx) [xxx]
(**29)** 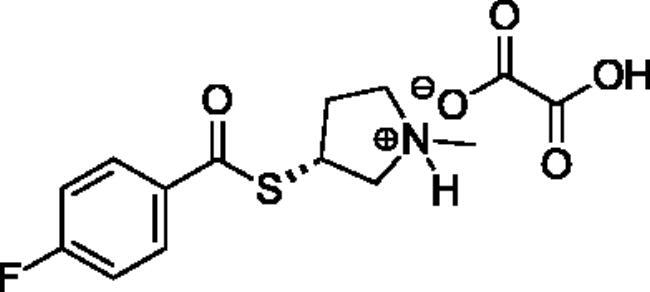	(++) [+++]	(o) [++]	(**30**) 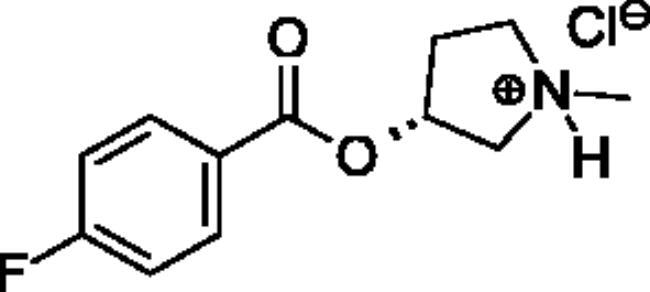	(−) [x]	(x) [xx]	(x) [x]	(xx) [x]

Staining of human and mouse brain tissues using the choline thioester derivatives ATChI (**1**), BTChI (**3**), **5**, and **6** resulted in staining with varying intensity. In the human and mouse cortex, histochemistry with ATChI (**1**) and BTChI (**3**) at pH 6.8 resulted in strong staining associated with Aβ plaques, as described previously^22^^,^^26^^,^^28^. At pH 8.0, ATChI (**1**) and BTChI (**3**) yielded strong staining associated with neurons, neuropil and axons with distinct distributions in the human thalamus and mouse midbrain, as reported previously^19^^,^^47^. Thioesters **5** and **6** did not result in staining in human brain tissue but did yield weak ChE staining associated with Aβ plaques and neural elements in the mouse.

In the human brain tissue, the alkyl *N*-methylpiperidinyl thioester **7** yielded weak staining associated with Aβ plaques and strong staining of neural elements. There was no staining with **9** in human tissue. In mouse tissue, there was strong staining associated with Aβ plaques and neural elements when using **7,** while **9** showed weak staining of plaques and neural elements.

The aryl *N*-methylpiperidinyl thioesters **11** and **13** did not result in staining of ChEs associated with Aβ plaques or neural elements in the human brain. Similarly, **11** and **13** did not produce staining of ChEs associated with Aβ plaques in mouse tissue, but **13** did result in weak staining of neural elements.

Thioesters of alkyl *N*-methylpyrrolidinyl, **15**, **17**, **19**, and **21**, all proved to be good substrates for staining ChEs associated with Aβ plaques and neural elements as demonstrated by staining in both human and mouse tissue. An enantiomeric effect was not observed for **15** and **17** or **19** and **21** and thus only one enantiomer has been shown in Figures 4,5 for these compounds.

In the aryl *N*-methyl pyrrolidinyl thioester series, the *R* and *S* enantiomers **23** and **27** respectively, did not yield staining in human brain tissue. Similarly, **23** and **27** did not result in staining associated with Aβ plaques in the mouse but did provide weak staining of neural elements. The *R* and *S* enantiomers of aryl *N*-methyl pyrrolidinyl thioesters **25** and **29,** respectively, stained Aβ plaques but not neural elements in human brain tissue, though they stained both structures in the mouse brain.

In the staining conditions employed here, there were several thioesters that either yielded weak staining or no staining, including **5**, **6**, **9**, **11**, **13**, **23**, and **27**. To determine if these compounds were engaging with ChEs but undergoing slow hydrolysis, they were employed as competitive substrates of ATChI (**1**) and BTChI (**2**) for AChE and BChE staining respectively. That is, thioester probes were added to the staining medium for AChE or BChE activity to determine if they would reduce overall staining. Tested in this way, compounds **5**, **6**, **9**, **11**, **13**, **23**, and **27** all reduced AChE and BChE staining, thus engaging with these enzymes and altering the rate of hydrolysis of their usual respective substrate (Supplementary Material, Figures S1 and S2).

### Inhibition of histochemical staining by ester derivatives

The addition of esters to the KR histochemical method for staining of AChE or BChE activity resulted in a reduction of staining of Aβ plaques (pH 6.8) and neural elements (pH 8.0) in human and mouse brain tissues that ranged from no inhibition (−) to strong inhibition (xxx) (Figures 2,3). The qualitative analysis of how esters affected AChE and BChE staining in human (round brackets) and mouse [square brackets] brain tissues is summarised in Table 2. Figures 4,5 show representative photomicrographs of histochemical staining in the presence of esters in human and mouse tissue, respectively.

Staining of human and mouse tissue for AChE or BChE activity using the modified KR histochemical method at pH 6.8 or 8 yielded staining associated with Aβ plaques and neural elements, respectively. Without the addition of an ester, this tissue served as a positive control in terms of staining intensity. Adding the ester substrate AChI (**2**) to the KR histochemical staining method for AChE activity resulted in a reduction of staining intensity for this enzyme in both human and mouse tissues. This indicates that the ester AChI **(2)** was effective in competing with the thioester substrate ATChI (**1**). Similarly, the ester BChI (**4**) reduced the staining intensity for BChE by the thioester BTChI (**3**). These experiments demonstrate that this KR histochemical method can be used to determine whether esters engage with ChEs associated with neural elements and AD pathology in brain tissues.

In human and mouse brain tissues, the acetyl *N*-methyl piperidinyl ester **8** (5 mM) resulted in a slight to moderate reduction of AChE staining associated with Aβ plaques and neural elements but did not reduce BChE staining. The *N*-butyryl piperidinyl ester **10** (5 mM) showed a slight to moderate reduction of AChE and BChE staining associated with Aβ plaques and neural elements in human and mouse tissue.

The aryl *N*-methylpiperidinyl esters **12** (1 mM) and **14** (5 mM) resulted in a moderate to strong reduction of AChE and BChE staining associated with Aβ plaques and neural elements in both human and mouse tissues.

In the human brain, the *S* enantiomer of acetyl *N*-methylpyrrolidinyl ester **16** (5 mM) resulted in a moderate reduction of staining intensity for AChE and BChE associated with neural elements but not Aβ plaques. In the mouse, **16** did not inhibit AChE associated with either neural elements or plaques but did slightly inhibit BChE staining in these structures. The *R* enantiomer, compound **18** (5 mM), showed a slight to moderate reduction of AChE and BChE staining associated with Aβ plaques and neural elements in human tissue. Staining in the presence of this compound was comparable in mouse tissue except for BChE staining of neural elements, which was not reduced. Butyryl *N*-methyl pyrrolidinyl ester **20** (5 mM) showed a moderate to the strong reduction in AChE and BChE staining associated with Aβ plaques and neural elements in human and mouse tissue. The *R* enantiomer, compound **22** (5 mM), resulted in a moderate reduction of AChE staining associated with Aβ plaques and neural elements in human and mouse tissues. For BChE staining, the reduction in staining associated with plaques and neural elements by **22** was moderate in human tissue and slight in mouse.

The aryl *N*-methylpyrrolidinyl ester derivatives **24** (1 mM), **26** (1 mM), and **28** (1 mM) all resulted in a moderate to the strong reduction of AChE and BChE staining associated with plaques and neural elements in human tissue. Staining was generally comparable in mouse tissue with the exception that **26** only slightly reduced staining of BChE associated with Aβ plaques and neural elements. In human tissue, compound **30** (1 mM) showed no effect on AChE staining associated with plaques but slightly reduced staining associated with neural elements, while only a slight reduction to BChE staining associated with plaques and moderate reduction in staining of neural elements was observed. In the mouse, **30** resulted in a slight to moderate reduction in AChE and BChE staining associated with plaques and neural elements.

## Discussion

Nuclear imaging probes targeting the cholinergic system have the potential to aid in AD diagnosis as well as treatment monitoring^62^. Substrate-based imaging probes have been successful in demonstrating ChE activity in regions that are comparable to the distribution of neural elements containing these enzymes^29–31^^,^^33–35^^,^^41–43^. In addition, AChE ligands have shown decreased uptake in AD^31–33^^,^^36–38^, a finding that recapitulates what has been observed in ChE isolation studies^26^^,^^27^. In contrast to what as been observed histochemically^21^^,^^22^^,^^24^, ChE probes have not shown increased uptake in regions where these enzymes are associated with Aβ plaques in the AD brain^30^^,^^31^^,^^43^. These observations may be the result of the altered biochemical properties of AChE and BChE when associated with Aβ plaques and NFTs^22^^,^^26^^,^^48^ which may affect the kinetics of any interaction between a substrate and its enzyme target and should be considered when screening potential probes.

The enzyme kinetic spectrophotometric methods used herein remain some of the most rapid and sensitive means to determine kinetic parameters of substrate or inhibitor probes for AChE and BChE^51–53^^,^^63^. However, these methods employ ChEs that have been isolated and purified and are not in the biochemically altered form of those associated with AD pathology. Isolating ChEs from human AD brain tissue has shown that ChEs appear to revert to their ‘normal’ biochemical properties in the enzyme kinetic process^26^. Similarly, when BChE purified from plasma is permitted to bind with Aβ plaques in a 5XFAD/BChE-knockout mouse model, its biochemical properties change to that seen in AD^64^. These observations highlight the importance of screening potential imaging probes with ChEs when they are associated with AD pathology and in their biochemically altered form.

The KR histochemical method provides a means to examine the interaction of probes with ChEs associated with AD pathology. This method for staining of AChE or BChE activity typically employs the thioesters ATChI (**1**) and BTChI (**3**), respectively, to map the distribution of ChEs associated with neural elements^16–18^^,^^20^^,^^47^^,^^65^ or Aβ plaques and NFTs^24^^,^^26^. To evaluate the KR method as a potential screening method for the development of ChE probes for AD diagnosis, several thioester and corresponding esters were synthesised and subsequently evaluated with this method. The distribution and intensity of the resulting staining provided a means to evaluate how well these compounds engaged with ChE-associated elements. Thioesters with better enzyme affinity and rate of hydrolysis would yield more intense staining than those that did not. However, the interaction of esters with AChE or BChE could not be visualised directly with the KR staining technique since hydrolysis of these substrates does not produce the chromogenic products necessary for histochemical visualisation. Therefore, employing these compounds as competitors of ATChI or BTChI for enzyme access, their interaction with ChEs can be studied. Spectrophotometric evaluation of the enzyme kinetic parameters of non-chromogenic esters has been done previously^51–53^^,^^57^ but has not been done with the KR histochemistry staining technique. Yet, the basic principle remains the same, as esters with a high affinity for AChE or BChE should compete well with their respective thioesters (ATChI or BTChI) and result in a reduced staining intensity compared to an ester with low ChE affinity. As a proof-of-principle, the esters AChI and BChI were found to cause a reduction in staining and indicated they were competing with their respective thioesters, ATChI and BTChI, for access to brain tissue ChEs (Table 2, Figures 4,5). This demonstrated that the interaction of ester substrates with brain tissue ChEs can be visualised using the KR method.

Comparing the kinetic parameters obtained from the spectrophotometric method and the resulting staining with the KR method revealed discrepancies for a number of thioesters examined. That is, the kinetic parameters of several thioesters, which were comparable to BTChI, indicated they should stain brain tissue ChEs using the KR method, but did not. For example, the aryl *N*-methylpyrrolidinyl thioesters **23** and **27** were found to be specific substrates for BChE with parameters comparable to BTChI (Table 1). However, these compounds resulted in no staining in human brain tissue and weak staining in neural elements of the mouse (Table 2). Similarly, the BChE specific butyryl *N*-methylpiperidinyl derivative **9** demonstrated favourable kinetic parameters but resulted in no staining in human brain tissue and only weak staining in that of mouse. Yet, the BChE-specific alkyl *N*-methylpyrrolidine thioesters **19** and **21**, with kinetic parameters comparable to **23** and **27** (Table 1), provided excellent staining of plaques and neural elements in both human and mouse brain tissues (Table 2). These results highlight the importance of evaluating compounds with brain tissue ChEs when associated with AD pathology in addition to determining kinetic parameters *in vitro*. The disconnect between spectrophotometric and KR methods may be the result of the altered biochemical properties of ChEs when associated with AD pathology. However, the lack of neural element staining also suggests there may be other factors involved as well.

Thioesters that failed to produce staining, or had very weak staining, with the KR histochemical method may have been the result of the compound either failing to engage with the enzyme target or that it was being hydrolysed too slowly to stain the brain tissues. To determine the cause of the poor staining with certain thioesters (i.e. **5**, **6**, **9**, **11**, **13**, **23**, and **27**) they were employed as competitive substrate inhibitors of ATChI and BTChI hydrolysis in the KR method for AChE and BChE staining, respectively. Thioesters used in this way all resulted in a reduction of AChE and BChE staining. Although the exact mechanism is not clear, these results suggest compounds were engaging with the enzyme target but showed weak substrate activity. ChEs associated with AD pathology, or neural elements for that matter, may have changes to their enzyme conformations that alter their kinetic parameters. It is possible that these compounds bind to the enzymes at the mouth of the active site gorge, preventing ATChI or BTChI substrate access to the catalytic site at the bottom of the gorge, and thus inhibit ChE staining. These observations support the notion that KR histochemical method may be a better method to screen substrate-based probes for ChEs.

The intense KR staining provided by the alkyl *N*-methylpyrrolidinyl thioesters suggest this class of compounds may have favourable properties as imaging probes (Table 2, Figures 4,5). The *R* and the *S* enantiomers of acetyl *N*-methylpyrrolidine, **15** and **17**, showed the most intense staining of ChEs associated with neural elements and with AD pathology observed in this study (Table 2, Figures 4, 5). Similarly, the *R* and the *S* enantiomers of butyryl *N*-methyl pyrrolidine thioesters, **19** and **21**, showed robust staining (Table 2, Figures 4,5). Comparing staining results of the alkyl *N*-methyl pyrrolidinyl and alkyl *N*-methyl piperidinyl thioesters suggests that compounds with five-membered rings may have more favourable properties as imaging probes compared to the six-membered ring counterparts.

Thioester staining with the KR method suggests the isotope used may impact a compound’s properties as an imaging probe. In contrast to their iodo derivatives, **23** and **27**, the *R* and *S* enantiomers of *p*-fluoroaryl *N*-methylpyrrolidinyl thioesters, **25** and **29,** yielded intense staining of ChEs associated with AD pathology in human and mouse brain tissue. Although neural elements were stained in mouse tissue by these compounds, the staining in human tissue suggests some ability to distinguish between ChEs associated with normal neural elements and that with AD pathology.

Additional compounds assessed, such as the acetyl *N*-methylpiperidinyl thioester **7**, yielded strong staining of neural elements (Table 2, Figure 4) consistent with a previous study in the human brain^49^. Compound **7** resulted in weak staining of ChEs associated with AD pathology in human tissue, suggesting that imaging studies conducted with its analogous radiolabelled probe^38^ most likely showed activity associated with neural elements. Since this compound stained ChEs associated with both normal and pathological structures in the mouse (Table 2, Figure 5), it will be of interest to compare the performance of this compound as a PET agent in the 5XFAD mouse model to gain further insights to its utility as a radioprobe.

Macdonald et al.^49^ also examined several thioesters for their interaction with ChEs in human brain tissues *via* the KR histochemical staining method. Compound **7**, re-examined here, generally yielded comparable results. It is of note that **9** showed BChE staining previously, following a longer incubation period (18 h)^49^ than used in the present study (2.5 h). However, for the purposes of evaluating imaging probes, longer incubation periods are not of practical use. Compounds **11** and **13**, were not examined previously due to poor solubility. To improve solubility and allow histochemical evaluation herein, compounds **11** and **13** were made as HCl salts. Although compound **13** still yielded poor histochemical staining, the solubility was sufficient to determine that the lack of staining was due to poor interaction with ChEs rather than insufficient concentration. However, despite being made a salt, the solubility for **11** was still too low to determine if this compound did not interact well with ChEs or if the solubility was simply too low to yield sufficient staining product.

Generally, ester compounds showed inhibition of ChE staining with KR. The alkyl *N*-methylpiperidinyl, aryl *N*-methylpiperidinyl, alkyl *N*-methylpyrrolidinyl, and aryl *N*-methylpyrrolidinyl esters all resulted in a reduction of ChE staining in human and mouse tissues (Table 2, Figures 4,5). The degree to which staining was reduced was generally consistent with the affinity of these compounds for AChE or BChE. That is, esters with low *K*_m_ values (Table 1) resulted in the greatest reduction in staining (e.g. **24**, *K*_m_: 28.6 × 10^−6^ M) while those with a high *K*_m_ showed little reduction in staining (e.g. **8**, *K*_m_: 3730 × 10^−6^ M). Although a reduction in staining resulting from ester compounds competing for enzyme access indicates interaction with ChEs associated with neural elements and AD pathology, it does not provide insight as to whether this interaction is sufficient for a probe to succeed as a molecular imaging agent. This becomes apparent when comparing certain ester and thioesters analogues. For example, the aryl *N*-methylpiperidinyl ester, **12**, significantly reduced the staining intensity of both AChE and BChE yet its thioester analogue, **11**, failed to stain tissue, despite having comparable kinetic parameters. In fact, **11** inhibited ChE staining, further underlining the notion that interactions with ChEs in tissue are different than those in spectrophotometric methods. This further highlights the point that to effectively screen radioprobes for AD, it is necessary to examine their properties towards ChEs associated with AD pathology. Thus, evaluating potential imaging probes using histochemical method may prove to have a number of benefits, namely, examining a compounds interaction with biochemically altered plaque-bound ChEs and providing a rapid and cost-effective means to screen compounds prior to radiolabeling.

This study has several limitations. The 5XFAD mouse model is not a perfect animal model of AD, but is adequate to screen these compounds. There may be differences in the structures between human and mouse ChEs to account for variances in staining between human and 5XFAD mouse tissues. In addition, these differences could be accounted for by differences in fixation methods. However, this does not preclude the use of this well-characterised model of amyloidosis as a screening tool for AD imaging probes. The KR histochemical method has demonstrated that ChEs associated with AD pathology have altered biochemical properties. However, the method used here to evaluate thioesters and esters cannot determine kinetic parameters such as *k_cat_* and *K_m_*. Future studies will be required to explore the feasibility of using histochemical methods or brain sections to determine such parameters. In addition, this histochemical method provides only qualitative information on the extent to which a thioester will be hydrolysed and stain, or an ester will reduce staining of ChEs in tissues. However, this method does provide information as to whether a particular compound interacts with brain tissue ChEs, either as the substrate or competitive substrate in brain tissue. It was also found that compounds that are specific for BChE, as determined by spectrophotometric method, interacted with brain tissue AChE as illustrated by a reduction in the histochemical staining for this enzyme. This implies that such compounds may act as potential probes for both AChE and BChE rather than specifically for one enzyme or the other. While this may be a limitation with respect to specificity, this method may provide insights into predicting which compounds may or may not be specific if taken further for incorporation of radioisotope and subsequent autoradiographic or imaging studies.

## Conclusions

Here we propose a possible solution to the fundamental issues currently identified in the development of ChE probes for imaging. The solution proposed includes the examination of potential probes with ChEs associated with AD pathology in brain tissues, prior to the costly and time-consuming process of evaluating radiolabeled compounds. In addition, some compounds identified as good substrates for AChE or BChE using spectrophotometric methods may not be suitable for imaging ChEs associated with AD pathology, and thus, require additional methods to screen potential imaging probes. To address these issues, the evaluation of novel compounds as substrate-based probes for ChEs using the KR histochemical method may provide valuable insights into the behaviour of such compounds when interacting with AD pathology-bound ChEs and may prove to be a rapid and effective means to screen compounds for future radiolabelling and imaging. These findings will need to be confirmed with radiolabelling, autoradiography, and SPECT/PET imaging experiments with key compounds assessed with the KR method.

## Supplementary Material

Supplemental Material

## Data Availability

The data that support the findings of this study are available from the corresponding author, SD, upon reasonable request.
