# Supplementary material for: A method for the efficient evaluation of substrate-based cholinesterase imaging probes for Alzheimer’s disease
Source: J Enzyme Inhib Med Chem. 2023 Jun 26;38(1):2225797. doi: 10.1080/14756366.2023.2225797 (PMC10294744; doi:10.1080/14756366.2023.2225797)
Supplement: Supplemental Material [file IENZ_A_2225797_SM6062.pdf]

Supplementary Material

A method for the efficient evaluation of substrate-based cholinesterase imaging probes for Alzheimer's disease

Sultan Darvesh[1,2,3], Scott Banfield[1], Maeve Dufour[1], Katrina L. Forrestall[1], Hillary Maillet[1], G. Andrew Reid[1], Dane Sands[1], Ian R. Pottie[3,4]

[1]Department of Medical Neuroscience, Dalhousie University, Halifax, Nova Scotia, Canada
[2]Department of Medicine (Geriatric Medicine & Neurology), Halifax, Nova Scotia, Canada
[3]Department of Chemistry and Physics, Mount St. Vincent University, Halifax, Nova Scotia, Canada
[4]Department of Chemistry, Saint Mary's University, Halifax, Nova Scotia, Canada

Corresponding Author:
Sultan Darvesh
Room 1308
Camp Hill Veterans' Memorial Building
5955 Veterans' Memorial Lane
Halifax, Nova Scotia, Canada B3H 2E1
Telephone: (902) 473-2490
Fax: (902) 473-7133
Email: sultan.darvesh@dal.ca

Table of Contents:

A. Synthetic Procedures and Spectroscopic Data:

Aryl Thiocholine Derivatives

(5) 2-[(*p*-Fluorobenzoyl)thio]-1-(trimethylammonio)ethane Iodide

(6) 2-[(*p*-Iodobenzoyl)thio]-1-(trimethylammonio)ethane Iodide

Alkyl N-Methylpiperidinyl Derivatives

(7) *S*-(1-Methylpiperid-4-yl) Ethanethioate Hydrochloride

(8) 4-Acetoxy-1-methyl-1-piperidinium Chloride

(9) *S*-(1-Methylpiperid-4-yl) Butanethioate Hydrochloride

(10) 4-Butyroxy-1-methyl-1-piperidinium Chloride

Aryl N-Methylpiperidinyl Derivatives

(11) (1-Methylpiperidin-4-yl) *p*-Iodobenzenecarbothioate Hydrochloride

(12) 4-(*p*-Iodobenzoyloxy)-1-methyl-1-piperidinium Chloride

(13) *S*-(1-Methylpiperidin-4-yl) *p*-Fluorobenzenecarbothioate Hydrochloride

(14) 4-(*p*-Fluorobenzoyloxy)-1-methyl-1-piperidinium Chloride

Alkyl N-Methylpyrrolidinyl Derivatives

(15) (*3S*)-*S*-(1-Methylpyrrolidin-3-yl) Ethanethioate

(16) *(3S)*-3-Acetoxy-1-methyl-1-pyrrolidinium Chloride

(17) (*3R*)-*S*-(1-Methylpyrrolidin-3-yl) Ethanethioate

(18) *(3R)*-3-Acetoxy-1-methyl-1-pyrrolidinium Chloride

(19) (*(3S)*-1-Methylpyrrolidin-3-yl) Butanethioate

(20) *(3S)*-3-Butyroxy-1-methyl-1- pyrrolidinium Chloride

(21) ((*3R*)-1-Methylpyrrolidin-3-yl) Butanethioate

(22) *(3R)*-3-Butyroxy-1-methyl-1- pyrrolidinium Chloride

Aryl N-Methylpyrrolidinyl Derivatives

(23) ((*3S*)-1-Methylpyrrolidin-3-yl) *p*-Iodobenzenecarbothioate Oxylate

(24) (*3S*)-3-(*p*-Iodobenzoyloxy)-1-methyl-1-pyrrolidinium Chloride

(25) ((*3S*)-1-Methylpyrrolidin-3-yl) *p*-Fluorobenzenecarbothioate Oxylate

(26) (*3S*)-3-(*p*-Fluorobenzoyloxy)-1-methyl-1-pyrrolidinium Chloride

(27) ((*3R*)-1-Methylpyrrolidin-3-yl) *p*-Iodobenzenecarbothioate Oxylate

(28) (*3R*)-3-(*p*-Iodobenzoyloxy)-1-methyl-1-pyrrolidinium Chloride

(29) ((*3R*)-1-Methylpyrrolidin-3-yl) *p*-Fluorobenzenecarbothioate Oxylate

(30) (*3R*)-3-(*p*-Fluorobenzoyloxy)-1-methyl-1-pyrrolidinium Chloride

References

B. Figures for histochemistry

Figure S1. Human brain tissues.

Figure S2. Mouse brain tissues

A. Synthetic Procedures and Spectroscopic Data:

Aryl Thiocholine Derivatives

(5) 2-[(*p*-Fluorobenzoyl)thio]-1-(trimethylammonio)ethane Iodide:

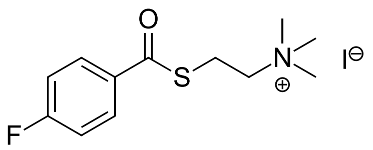

Synthesis:

2-[(*p*-Fluorobenzoyl)thio]-1-(trimethylammonio)ethane Iodide: 2-(Dimethylamino) ethanethiol hydrochloride (1.23 g, 8.66 mmol) was suspended in anhydrous dichloromethane (17 mL) and cooled in an ice bath. Triethylamine (3.0 mL, 21.7 mmol) was added dropwise; followed by 4-dimethylaminopyridine (0.106 g, 0.866 mmol) and 4-fluorobenzoyl chloride (1.2 mL, 10.4 mmol). The mixture was warmed to room temperature and stirred for 16 hrs where a white precipitate formed. After this stirring time, the reaction was gravity filtered to remove the precipitate, and the filter cake was washed with dichloromethane (2 × 10 mL). The filtrate was washed with 0.04 *N* $HCl_{(aq)}$ (50 mL), saturated $NaHCO_{3(aq)}$ (50 mL), and H_2O (50 mL). The organic layer was dried over Na_2SO_4, gravity filtered, and the filtrate was concentrated *in vacuo* to afford 1.58 g of an off-white solid. This material (1.51 g, 6.65 mmol) was used immediately without purification. It was dissolved in anhydrous THF (10 mL) and the reaction was cooled to 0^oC using an ice bath. Iodomethane (0.50 mL, 7.98 mmol) was added dropwise to the above solution resulting in the formation of a white precipitate. The mixture was warmed to room temperature and stirred for 2 hrs at this temperature. The precipitate was then collected via suction filtration, and the filter cake was washed with Et_2O (100 mL). The filter cake was

transferred to a round bottom flask and placed under a vacuum (1mm Hg) for 5 hrs to afford 2-[(*p*-fluorobenzoyl)thio]-1-(trimethylammonio)ethane iodide (1.92 g, 60% yield) as a white solid.

Spectroscopic data:

$MP_{(THF)}$: 197–199°C; IR(ATR) 3105, 3087, 3011, 2969, 2932, 1662, 1595, 1205, 906 cm^{-1}; ^{1}H NMR (400 MHz, DMSO-*d6*) δ 8.06-8.02 (m, 2H), 7.46-7.42 (m, 2H), 3.55-3.45 (m, 4H), 3.17 (s, 9H); ^{13}C NMR (100.7 MHz, DMSO-*d6*) δ 188.8 (0), 165.6 (d,$^{1}J_{C,F}$ = 253.5 Hz, 0), 132.3 (d, $^{4}J_{C,F}$ = 2.8 Hz, 0), 130.1 (d,$^{3}J_{C,F}$ = 9.7 Hz, 1), 116.5 (d,$^{2}J_{C.F}$ = 22.3 Hz, 1), 63.7 (2), 52.3 (3), 21.6 (2); LRMS (ESI^{+}): 242.1 (M^{+}); ^{19}F NMR (376.6 MHz, DMSO-*d6*) δ -103.8; HRMS (ESI^{+}): calculated for $C_{12}H_{17}FNOS^{+}$: 242.1009 amu; found for $C_{12}H_{17}FNOS^{+}$: 242.1011 amu; HPLC purity at 230 nm (25% CH_3CN : 75% CH_3OH, retention time: 1.837 mins): 99%.

(5) 2-[(*p*-Fluorobenzoyl)thio]-1-(trimethylammonio)ethane Iodide IR(ATR)

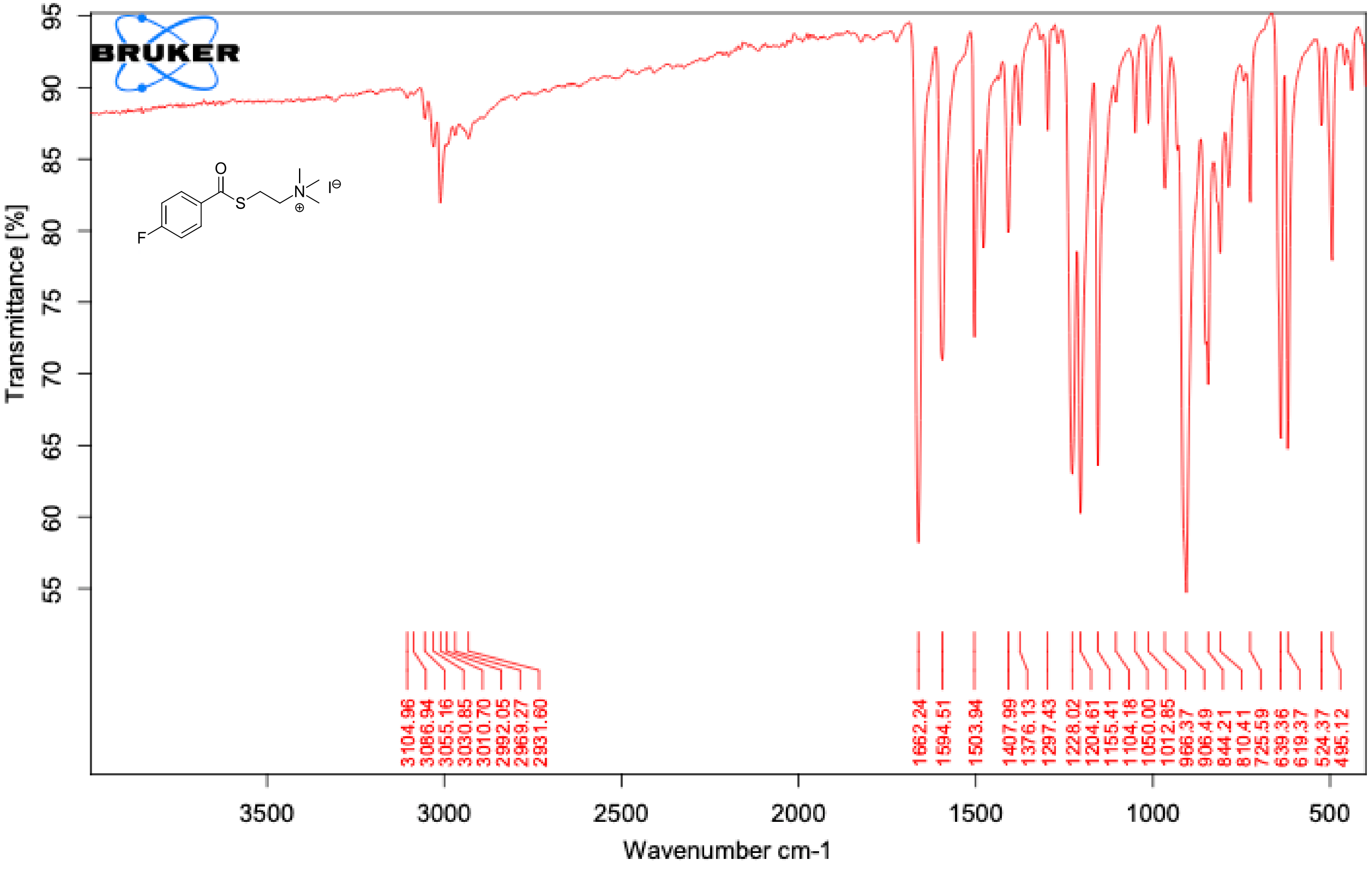

(5) 2-[(*p*-Fluorobenzoyl)thio]-1-(trimethylammonio)ethane Iodide ^{1}H NMR 400 MHz (DMSO-*d6*)

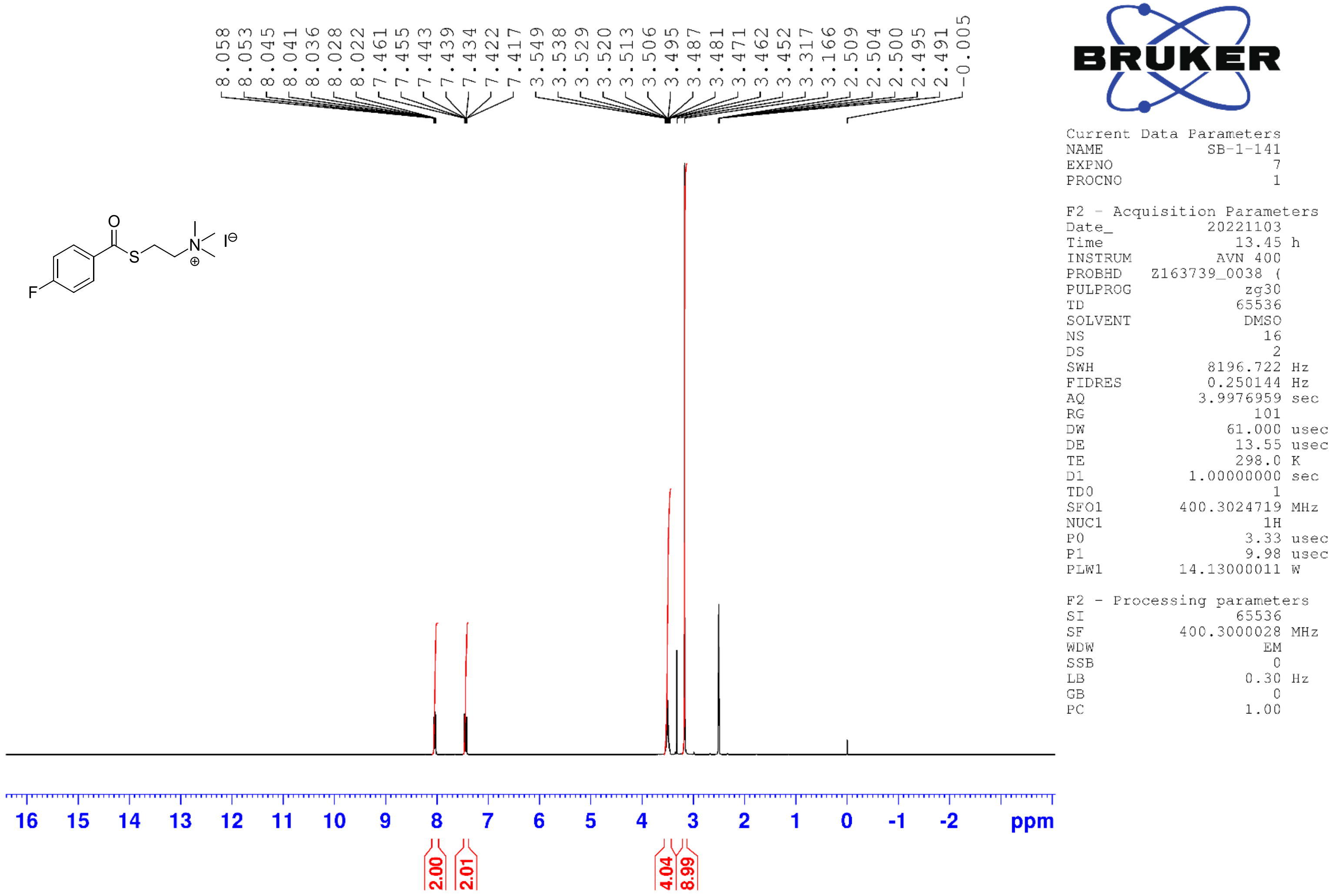

(5) 2-[(*p*-Fluorobenzoyl)thio]-1-(trimethylammonio)ethane Iodide ^{13}C NMR 100 MHz (DMSO-*d6*)

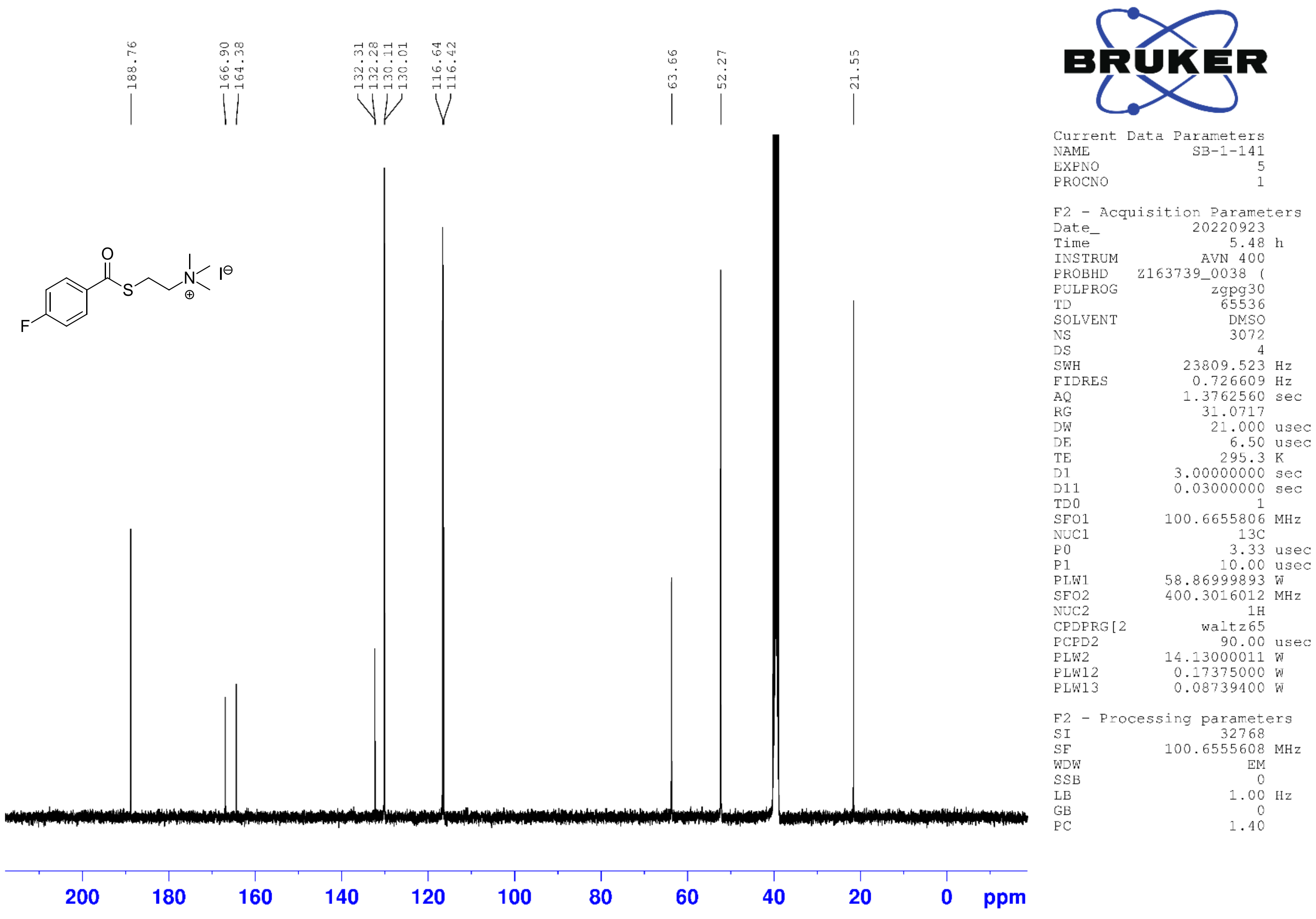

(5) 2-[(*p*-Fluorobenzoyl)thio]-1-(trimethylammonio)ethane Iodide High Resolution Mass Spectroscopy (ESI, positive mode)

Mass Spectrum SmartFormula Report

Analysis Info

Analysis Name	D:\Data\Xiao\Oct 14 2022000003.d	Acquisition Date	2022-10-14 10:43:25 AM	
Method	Xiao all 1.m	Operator	x	
Sample Name	TRV 6021	Instrument	compact	8255754.20059
Comment				

Acquisition Parameter

Source Type	ESI	Ion Polarity	Positive	Set Nebulizer	0.5 Bar
Focus	Not active	Set Capillary	3500 V	Set Dry Heater	181 °C
Scan Begin	50 m/z	Set End Plate Offset	-500 V	Set Dry Gas	4.0 l/min
Scan End	1500 m/z	Set Charging Voltage	2000 V	Set Divert Valve	Source
		Set Corona	0 nA	Set APCI Heater	0 °C

Meas. m/z	Ion Formula	m/z	err [ppm]
242.1011	C12H17FNOS	242.1009	-0.6

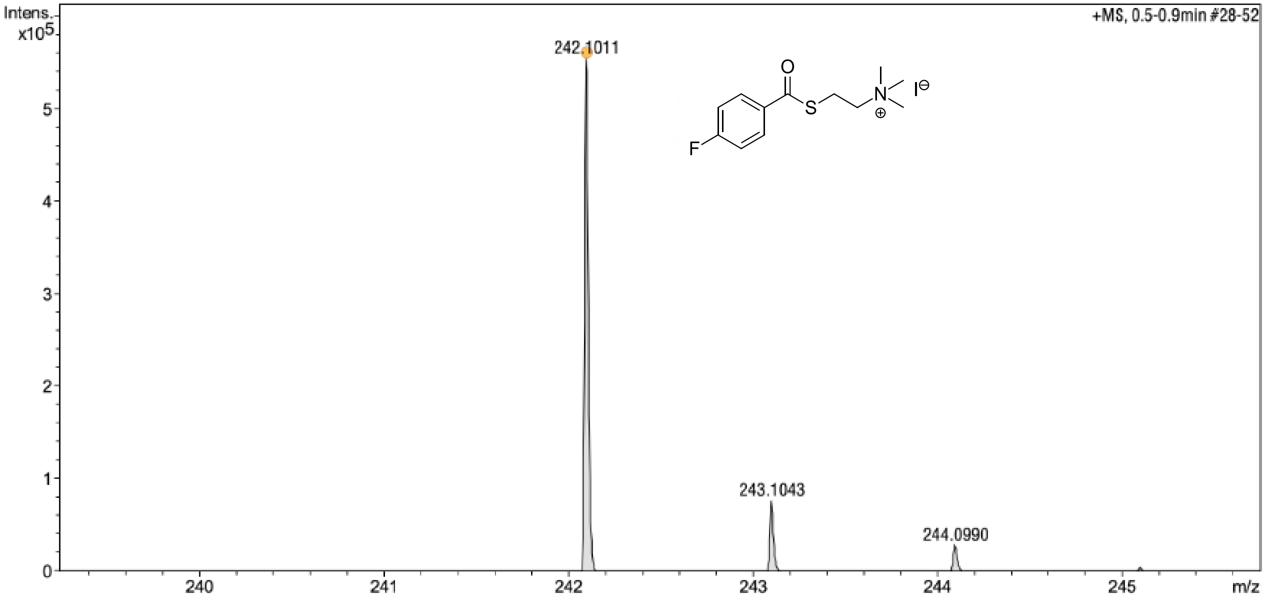

Oct 14 2022000003.d

Bruker Compass DataAnalysis 4.3 printed: 2022-10-14 10:47:21 AM by: x Page 1 of 1

(5) 2-[(*p*-Fluorobenzoyl)thio]-1-(trimethylammonio)ethane Iodide HPLC Trace

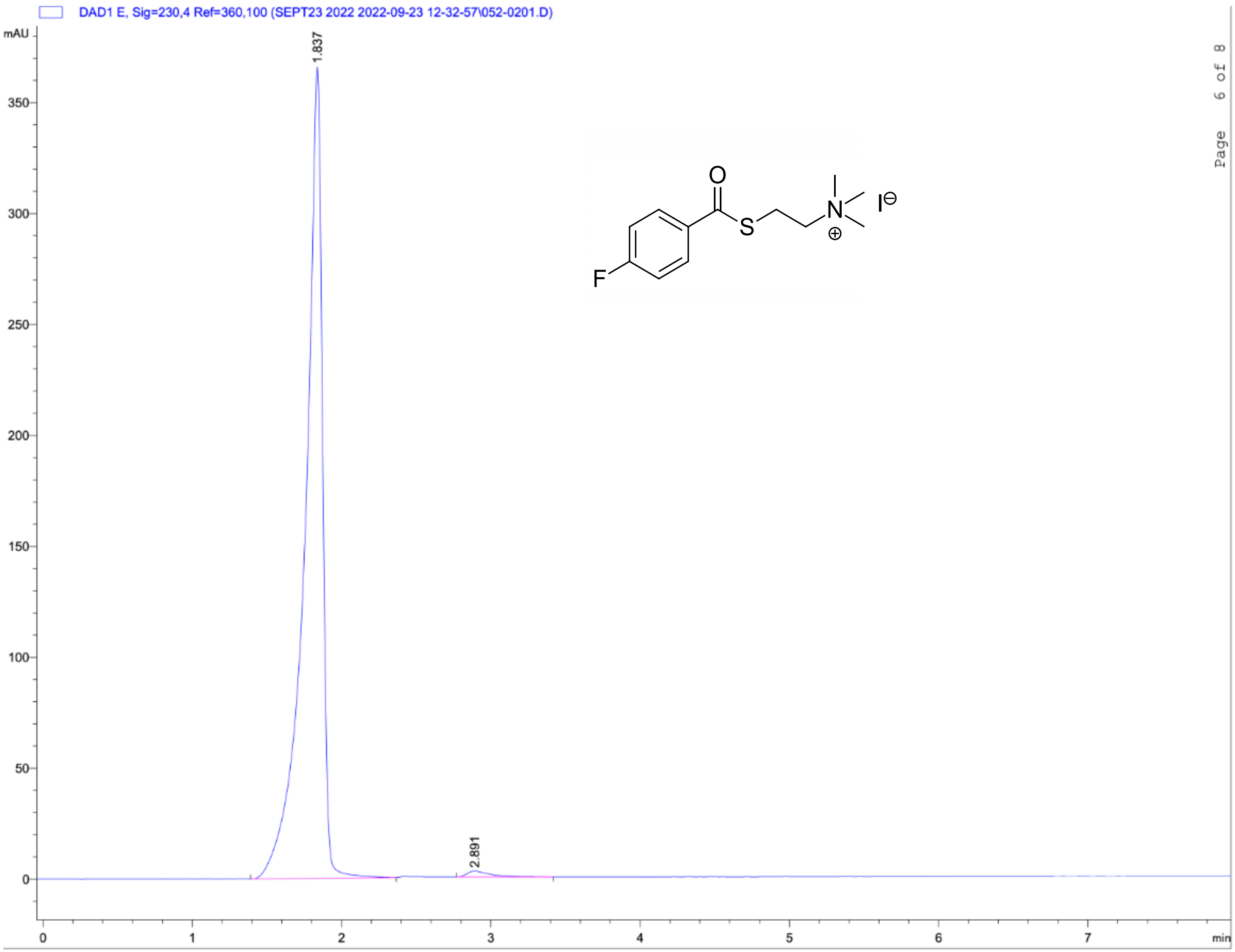

(6) 2-[(*p*-Iodobenzoyl)thio]-1-(trimethylammonio)ethane Iodide:

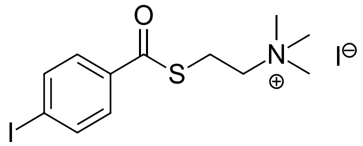

Synthesis:

2-[(*p*-Iodobenzoyl)thio]-1-(trimethylammonio)ethane Iodide: 2-(Dimethylamino)ethanethiol hydrochloride (1.16 g, 8.20 mmol) was suspended in anhydrous dichloromethane (16 mL) and cooled in an ice bath. Triethylamine (2.9 mL, 20.8 mmol) was added dropwise; followed by 4-dimethylaminopyridine (0.100 g, 0.82 mmol) and 4-iodobenzoyl chloride (2.62 mL, 9.84 mmol). The mixture was warmed to room temperature and stirred for 16 hrs where a white precipitate formed. After this, the reaction was gravity filtrated to remove the precipitate, and the filter cake was washed with dichloromethane (2 × 10 mL). The filtrate was washed with 0.04 N $HCl_{(aq)}$ (50 mL), saturated $NaHCO_{3(aq)}$ (50 mL), and H_2O (50 mL). The organic layer was dried over Na_2SO_4, gravity filtered, and the filtrate was concentrated *in vacuo* to afford 2.33 g of an off-white solid. This material (2.33 g, 6.95 mmol) was used immediately without purification. It was dissolved in anhydrous THF (10 mL) and cooled in an ice bath. Iodomethane (0.52 mL, 8.34 mmol) was added dropwise to the above solution resulting in the formation of a white precipitate. The mixture was warmed to room temperature and stirred for 2 hrs at this temperature. The precipitate was then collected *via* suction filtration, and the filter cake was washed with Et_2O (100 mL). The filter cake was transferred to a round bottom flask and placed under a vacuum (1 mm Hg) for 5 hrs to afford 2-[(*p*-iodobenzoyl)thio]-1-(trimethylammonio)ethane iodide (3.21 g, 82% yield) as a white solid.

Spectroscopic data:

$MP_{(THF)}$: >250°C; IR(ATR) 3041, 3006, 2942, 1680, 1650, 1576, 1207, 913, 835, 820 cm^{-1}; ^{1}H NMR (400 MHz, DMSO-*d6*) δ 8.00-7.98 (m, 2H), 7.71-7.69 (m, 2H), 3.54-3.45 (m, 4H), 3.17 (s, 9H); ^{13}C NMR (100.7 MHz, DMSO-*d6*) δ 189.7 (0), 138.3 (1), 134.9 (0), 128.5 (1), 103.5 (0), 63.6 (2), 52.3 (3), 21.5 (2); LRMS (ESI^+): 350.0 (M^+); HRMS (ESI^+): calculated for $C_{12}H_{17}INOS^+$: 350.0070 amu; found for $C_{12}H_{17}INOS^+$: 350.0068 amu; HPLC purity at 230 nm (25% CH_3CN : 75% CH_3OH, retention time: 1.790 mins): 98%.

(6) 2-[(*p*-Iodobenzoyl)thio]-1-(trimethylammonio)ethane Iodide IR(ATR)

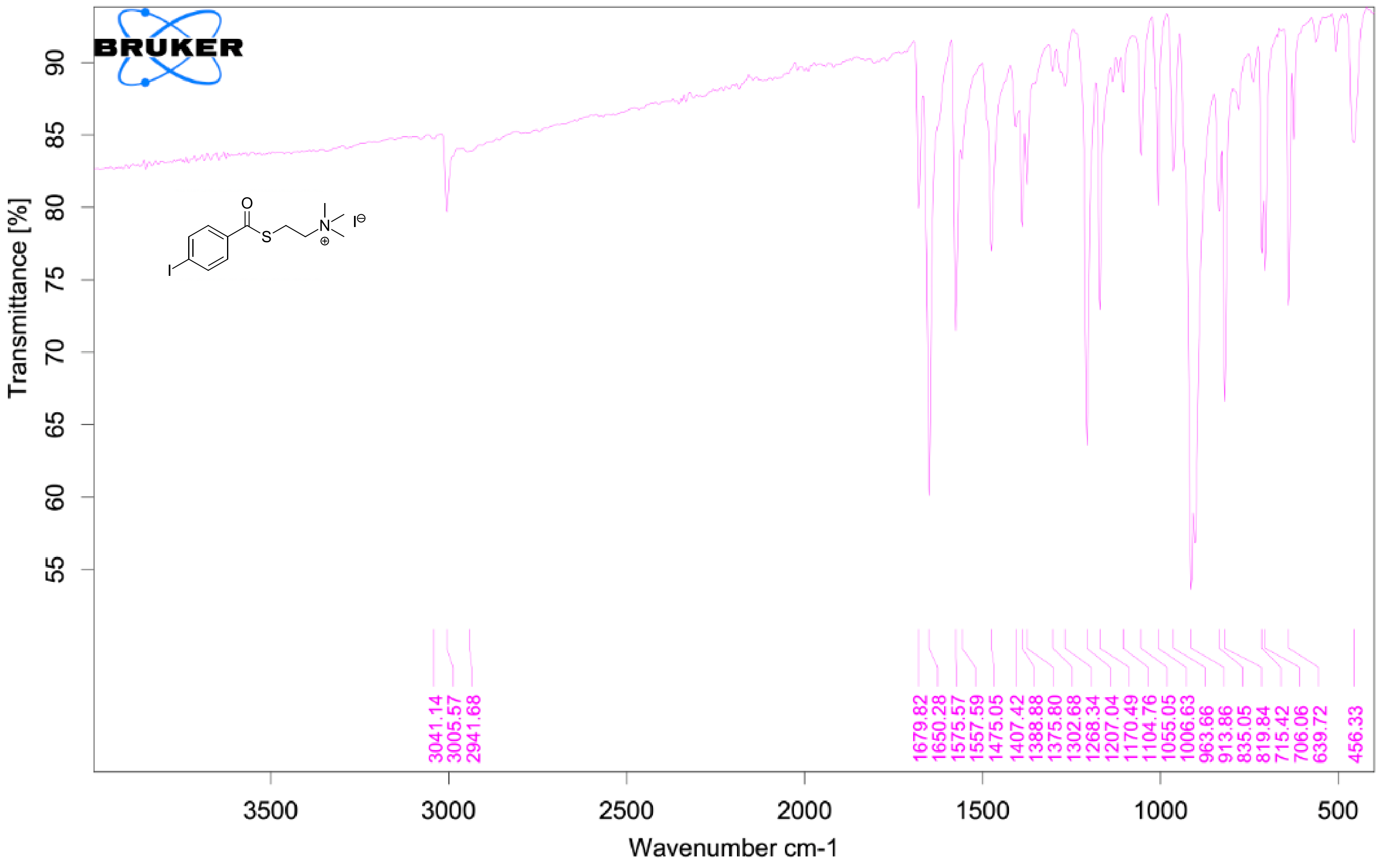

(6) 2-[(*p*-Iodobenzoyl)thio]-1-(trimethylammonio)ethane Iodide ^{1}H NMR 400 MHz (DMSO-*d6*)

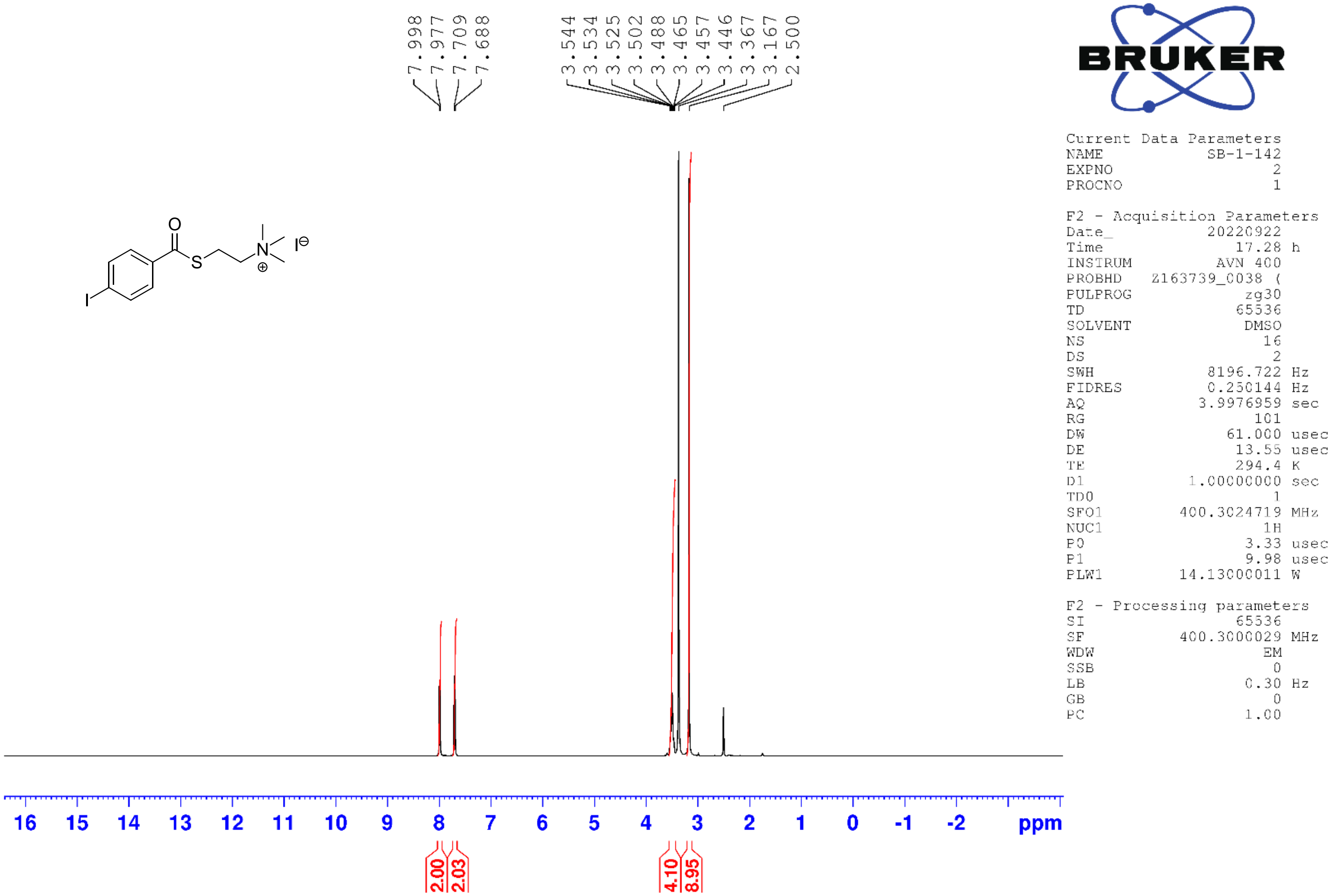

(6) 2-[(*p*-Iodobenzoyl)thio]-1-(trimethylammonio)ethane Iodide ^{13}C NMR 100 MHz (DMSO-*d6*)

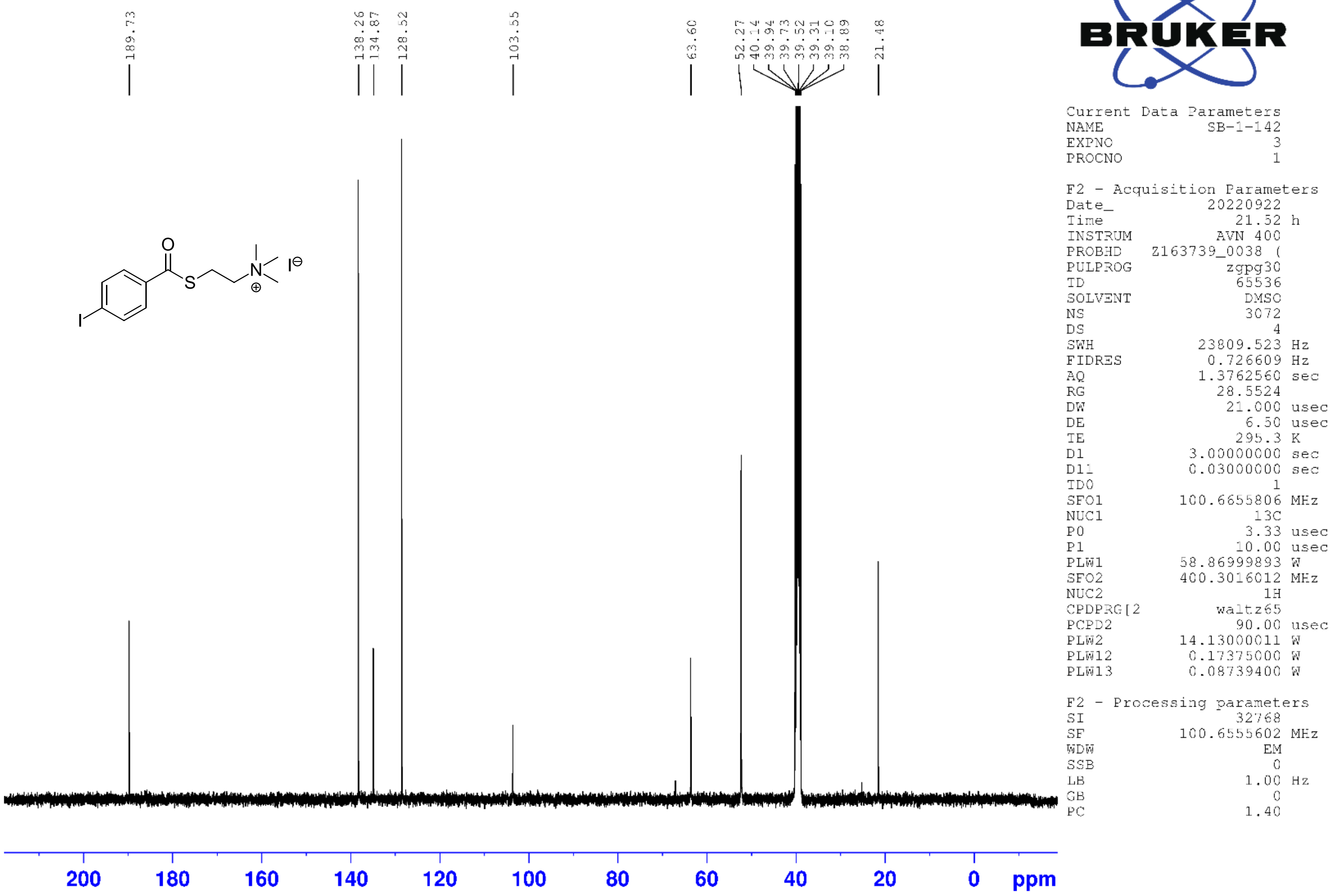

(6) 2-[(*p*-Iodobenzoyl)thio]-1-(trimethylammonio)ethane Iodide High Resolution Mass Spectrum (ESI, positive mode)

Mass Spectrum SmartFormula Report

Analysis Info

Analysis Name	D:\Data\Xiao\Oct 14 2022000004.d	Acquisition Date	2022-10-14 10:53:58 AM
Method	Xiao all 1.m	Operator	x
Sample Name	TRV 6022	Instrument	compact 8255754.20059
Comment			

Acquisition Parameter

Source Type	ESI	Ion Polarity	Positive	Set Nebulizer	0.5 Bar
Focus	Not active	Set Capillary	3500 V	Set Dry Heater	181 °C
Scan Begin	50 m/z	Set End Plate Offset	-500 V	Set Dry Gas	4.0 l/min
Scan End	1500 m/z	Set Charging Voltage	2000 V	Set Divert Valve	Source
		Set Corona	0 nA	Set APCI Heater	0 °C

Meas. m/z	Ion Formula	m/z	err [ppm]
350.0068	C12H17INOS	350.0070	0.6

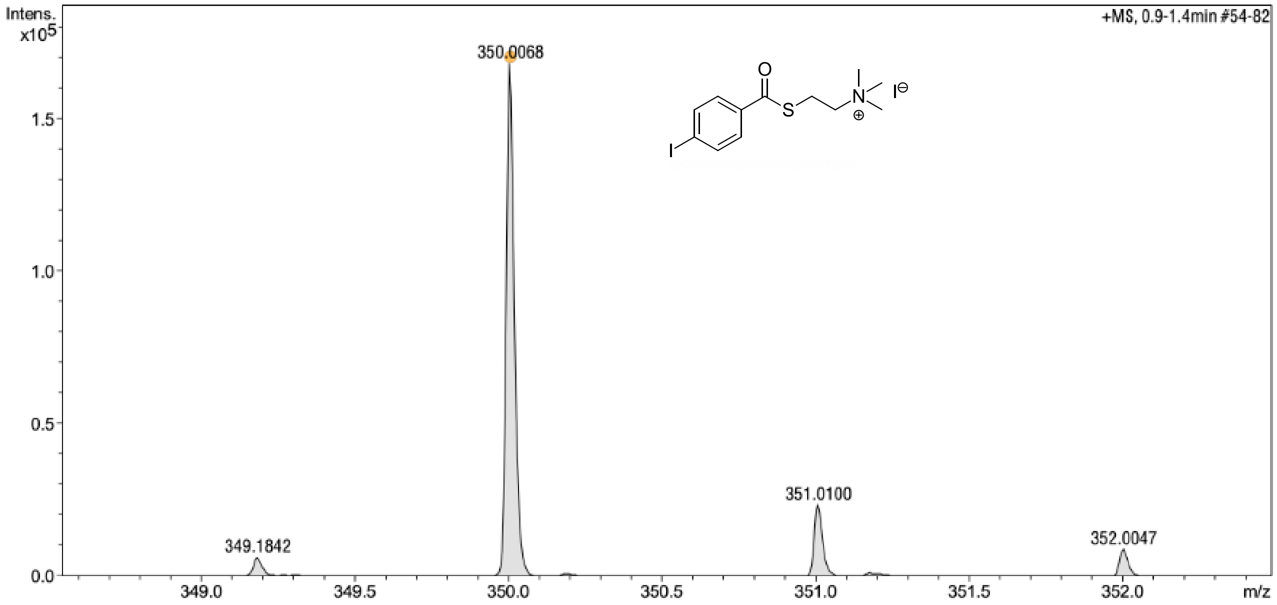

Oct 14 2022000004.d

Bruker Compass DataAnalysis 4.3 printed: 2022-10-14 10:57:16 AM by: x Page 1 of 1

(6) 2-[(*p*-Iodobenzoyl)thio]-1-(trimethylammonio)ethane Iodide HPLC

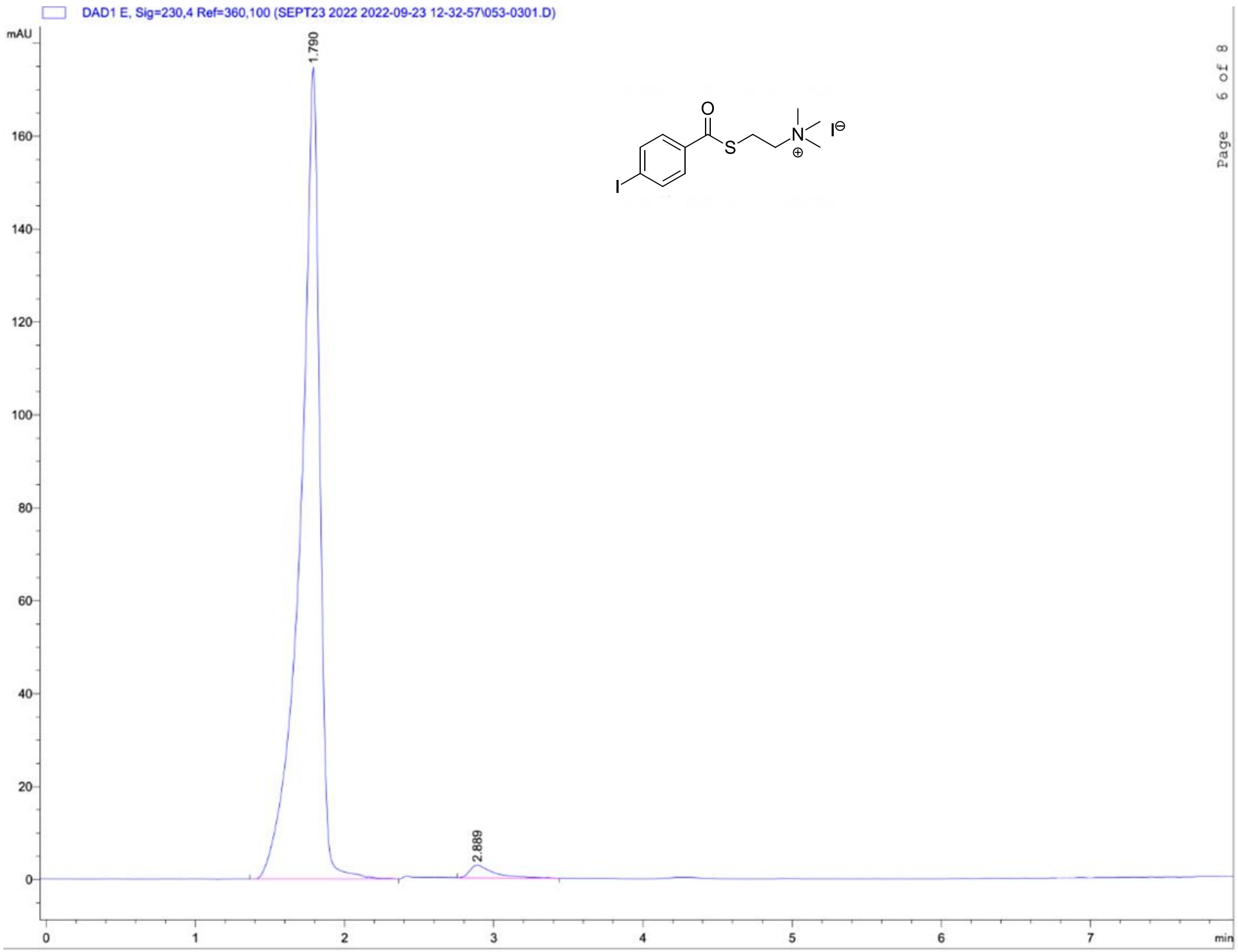

Alkyl N-Methylpiperidinyl Derivatives

(7) *S*-(1-Methylpiperid-4-yl) Ethanethioate Hydrochloride

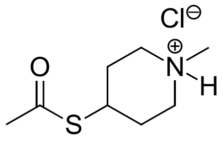

Synthesis:

***S*-(1-Methylpiperid-4-yl) Ethanethioate**: *N*-Methyl-4-piperidinethiol (0.50 mL, 4.2 mmol) was dissolved in anhydrous dichloromethane (5 mL), cooled to 0°C with an ice bath and stirred under an argon atmosphere. Acetyl chloride (0.895 mL, 12.6 mmol) was added dropwise at 0°C. The ice bath was removed after 5 mins, and reaction was stirred for 2 hrs at room temperature. After this time, saturated $NaHCO_{3(aq)}$ (10 mL) was added to the reaction and the layers were separated. The organic layer was extracted with dichloromethane (3 × 15 mL). The combined organic layers were dried over Na_2SO_4, gravity filtered and the filtrate was concentrated *in vacuo* to produce a clear colourless oil *S*-(1-methylpiperid-4-yl) ethanethioate (0.303 g, 42%). The analytical data was similar to previously published data.[1]

***S*-(1-Methylpiperid-4-yl) Ethanethioate Hydrochloride**: *S*-(1-Methylpiperid-4-yl) ethanethioate (0.245 g, 2.78 mmol) was dissolved in anhydrous diethyl ether (28 mL) under an argon atmosphere. HCl in diethyl ether (1 N - 2.11 mL, 2.11 mmol) was added dropwise and stirred for 1 hr. The solvent was removed *in vacuo* resulting in a white solid. The compound was triturated with diethyl ether (3 × 20 mL). Residual diethyl ether was removed *in vacuo* to produce *S*-(1-methylpiperid-4-yl) ethanethioate hydrochloride (0.198 g, 67% yield) as a pair of isomers (25:75).

Spectroscopic data:

$MP_{(diethyl\ ether)}$: 157−159°C; IR(ATR) 3007, 2921, 2478, 2418, 1687, 1120, 973, 635 cm^{-1}; ^{1}H NMR (400 MHz, DMSO-*d6*) δ isomer A: 10.29 (br s, 1H), 3.51-3.38 (m, 3H), 3.10-3.03 (m, 2H), 2.70 (s, 3H), 2.33 (s, 3H), 2.04-2.01 (m, 2H), 1.88-1.79 (m, 2H), isomer B: 10.29 (br s, 1H), 3.84 (br s, 1H), 3.41-3.38 (m, 2H), 2.98-2.92 (m, 2H), 2.75 (s, 3H), 2.33 (s, 3H), 2.25-2.18 (m, 2H), 1.88-1.79 (m, 2H); ^{13}C NMR (100.7 MHz, DMSO-*d6*) δ isomer A: 194.6 (0), 52.9 (2), 42.4 (3), 36.7 (1), 30.7 (3), 28.8, isomer B: 194.7 (0), 50.1 (2), 41.8 (3), 36.3 (1), 28.8 (2), 27.2 (3); LRMS (ESI^+): 174.1 (M^+); HRMS (ESI^+): calculated for $C_8H_{16}NOS^+$: 174.0947 amu; found for $C_8H_{16}NOS^+$: 174.0946 amu; HPLC purity at 254 nm (75% CH_3CN : 10% CH_3OH : 15% aqueous triethylamine [0.1% triethylamine in H_2O], retention time 3.685 mins): 98.7%.

(7) *S*-(1-Methylpiperid-4-yl) Ethanethioate Hydrochloride IR(ATR)

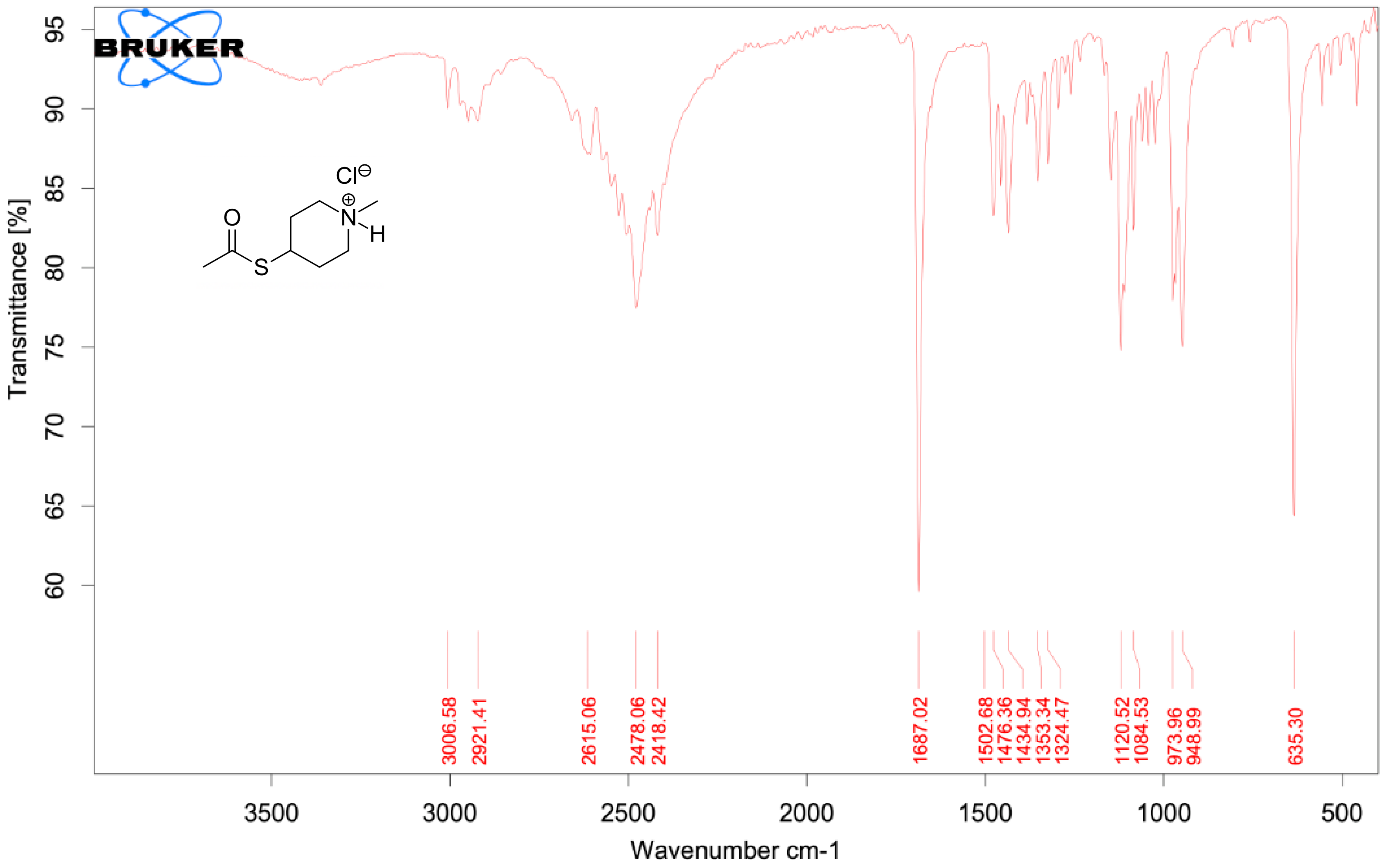

(7) *S*-(1-Methylpiperid-4-yl) Ethanethioate Hydrochloride ^{1}H NMR 400 MHz (DMSO-*d6*)

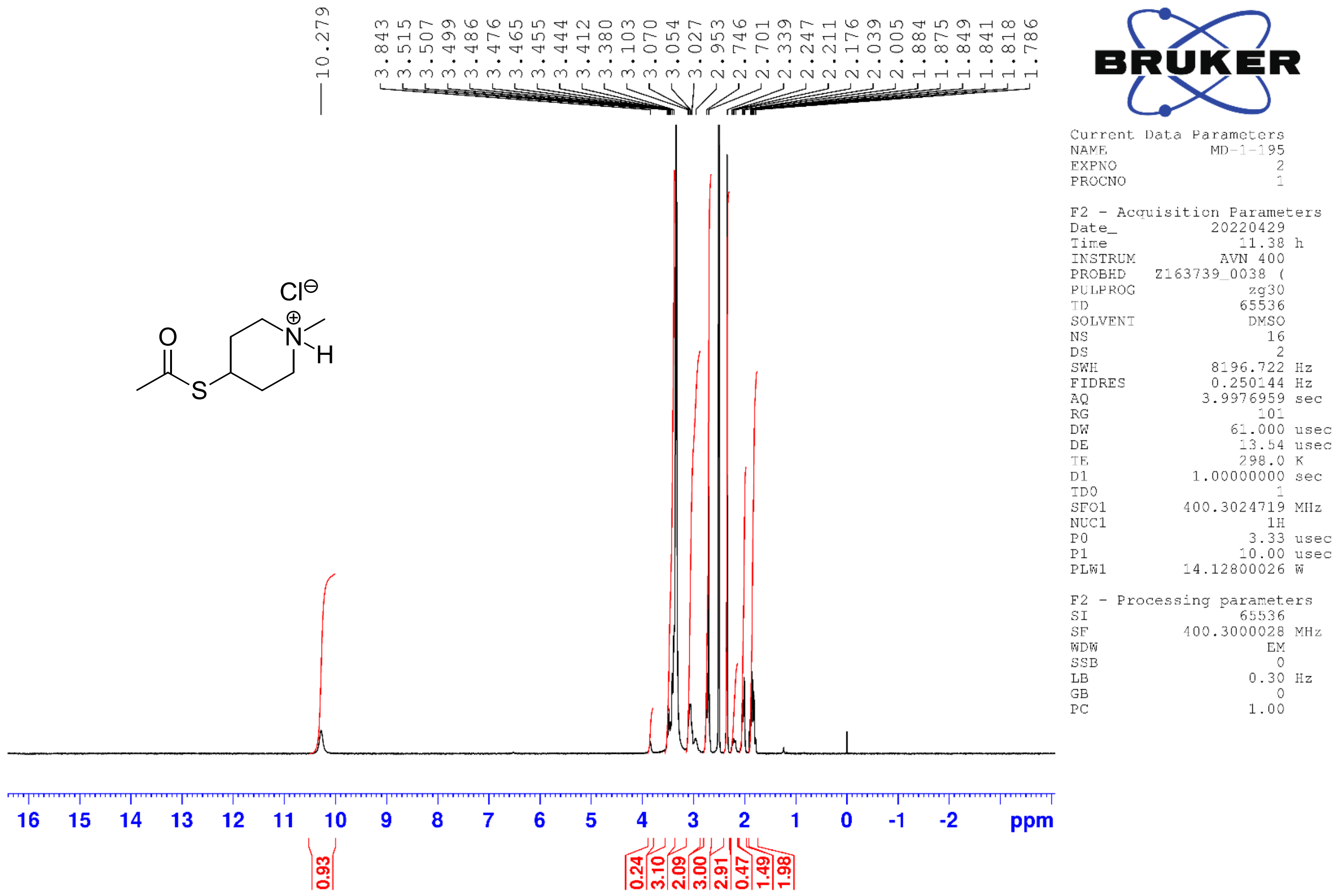

(7) *S*-(1-Methylpiperid-4-yl) Ethanethioate Hydrochloride ^{13}C NMR 100 MHz (DMSO-*d6*)

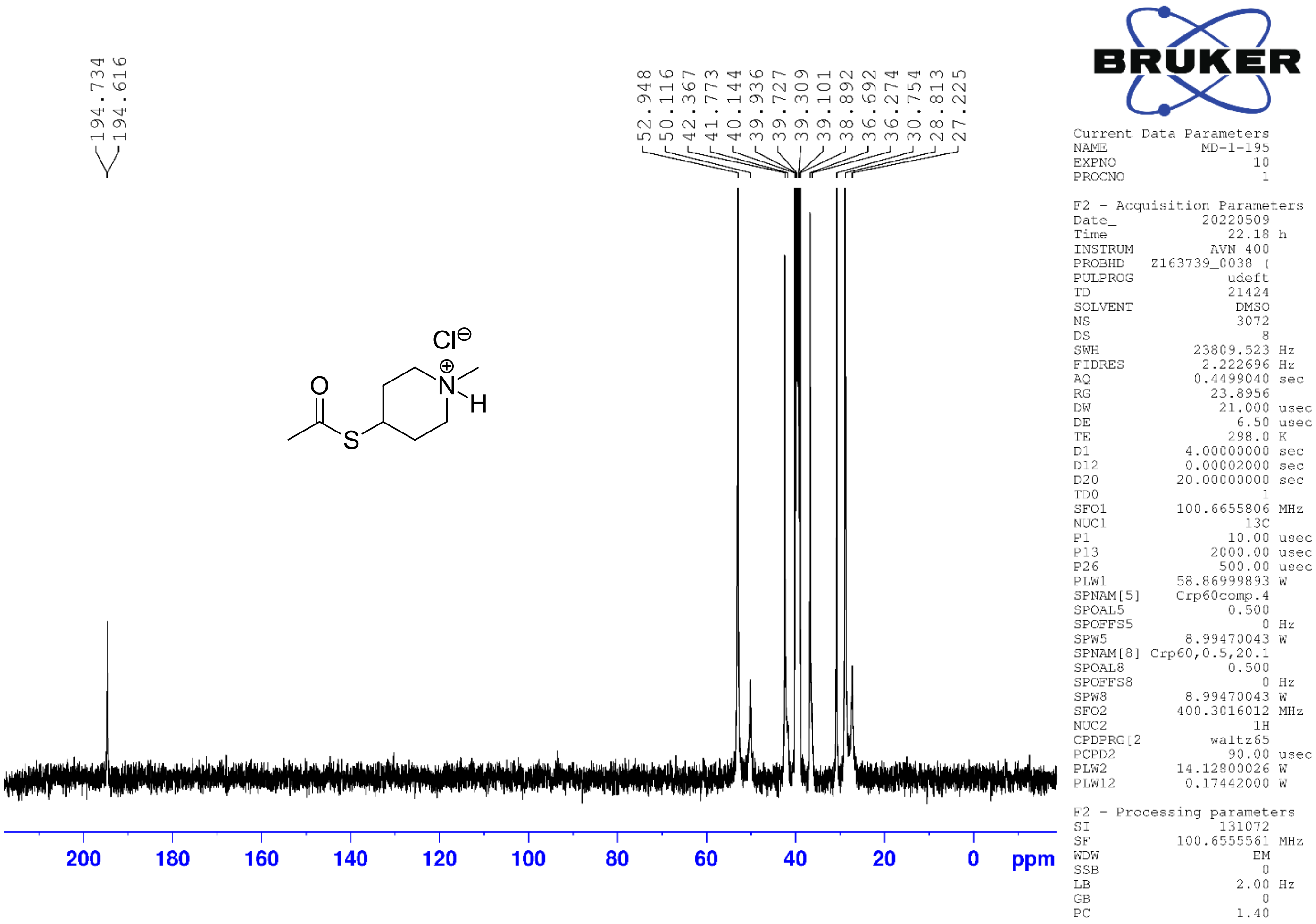

(7) *S*-(1-Methylpiperid-4-yl) Ethanethioate Hydrochloride High Resolution Mass Spectrum (ESI, positive mode)

Mass Spectrum SmartFormula Report

Analysis Info

Analysis Name	D:\Data\Xiao\July 07 2022000002.d	Acquisition Date	7/7/2022 9:07:46 AM	
Method	Xiao all 1.m	Operator	x	
Sample Name	TRV 6008 HCl	Instrument	compact	8255754.20059
Comment				

Acquisition Parameter

Source Type	ESI	Ion Polarity	Positive	Set Nebulizer	0.5 Bar
Focus	Not active	Set Capillary	3500 V	Set Dry Heater	180 °C
Scan Begin	50 m/z	Set End Plate Offset	-500 V	Set Dry Gas	4.0 l/min
Scan End	1500 m/z	Set Charging Voltage	2000 V	Set Divert Valve	Source
		Set Corona	0 nA	Set APCI Heater	0 °C

Meas. m/z	Ion Formula	m/z	err [ppm]
174.0946	C8H16NOS	174.0947	0.6

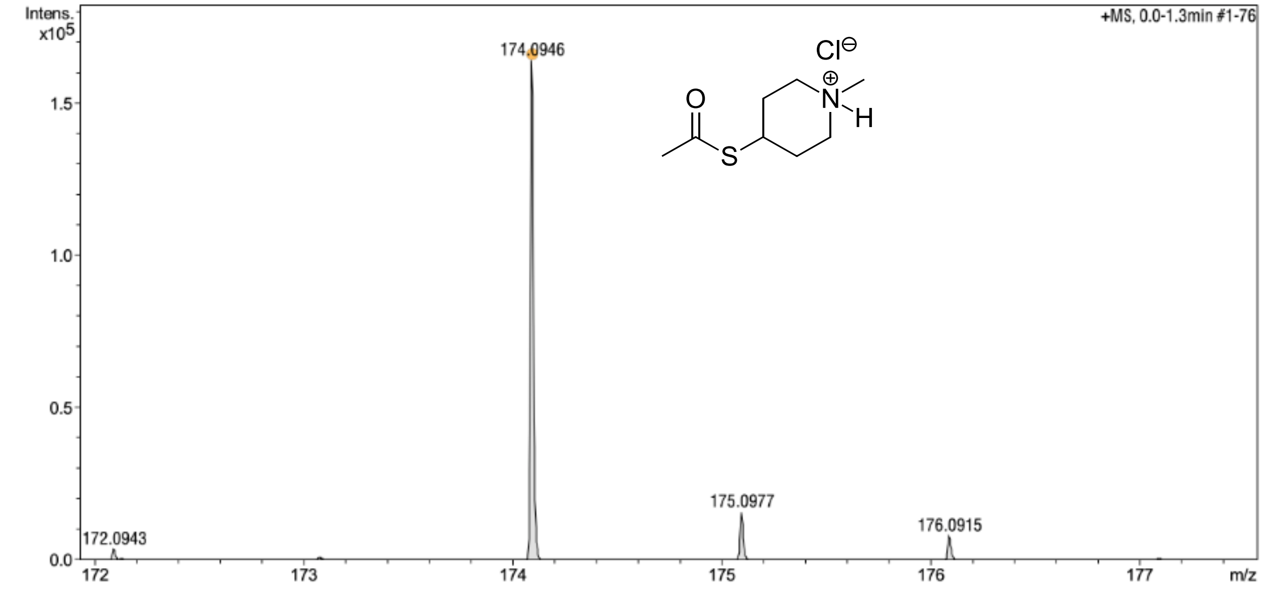

July 07 2022000002.d

Bruker Compass DataAnalysis 4.3 printed: 7/7/2022 9:10:37 AM by: x Page 1 of 1

(7) *S*-(1-Methylpiperid-4-yl) Ethanethioate Hydrochloride HPLC

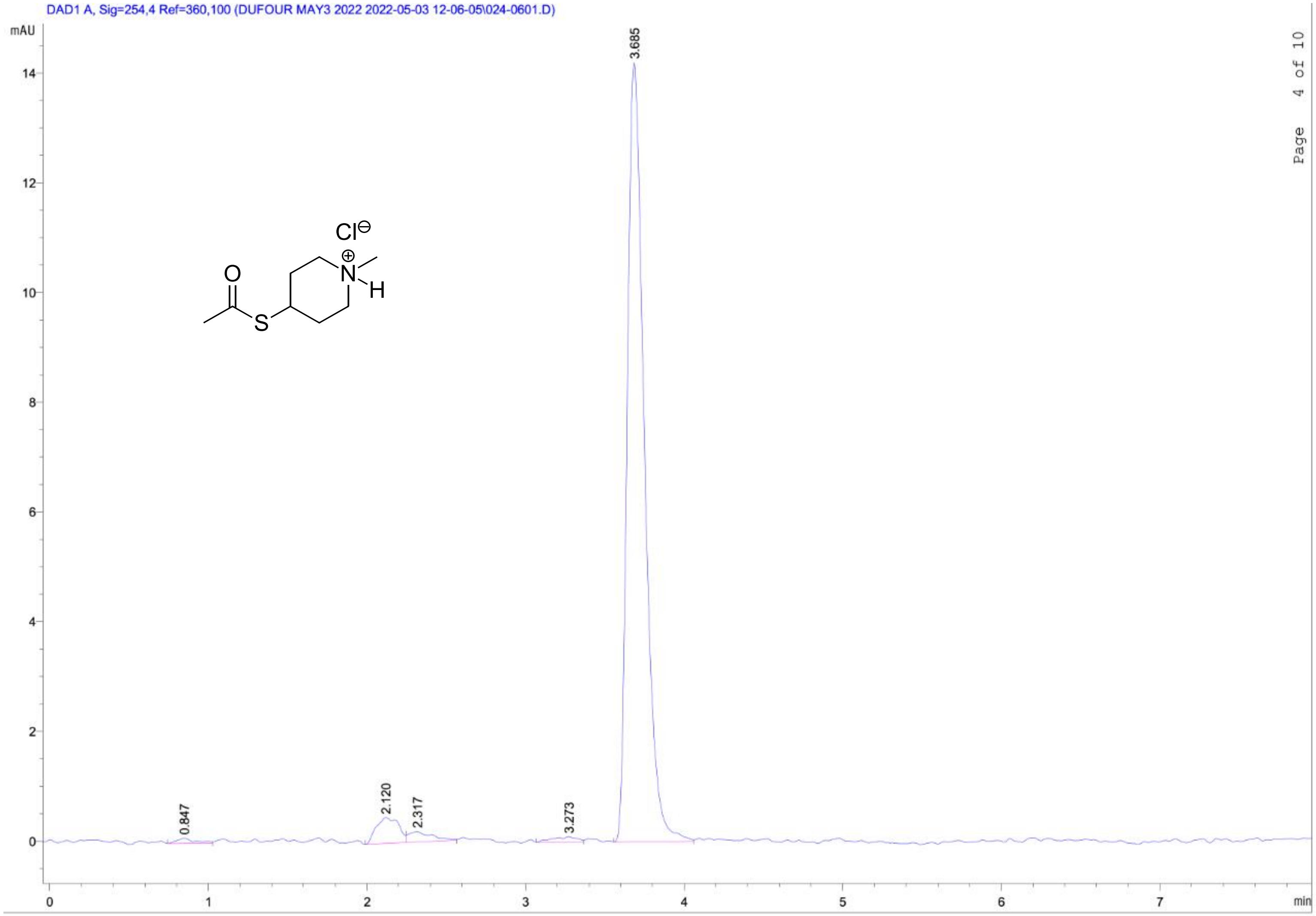

(8) 4-Acetoxy-1-methyl-1-piperidinium Chloride

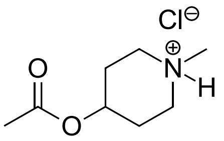

Synthesis:

1-Methyl-4-piperidyl Acetate: A slightly modified procedure published by Bormans *et al.*[2] was used to produce 1-methyl-4-piperidyl acetate. Briefly, *N*-methyl-4-piperidinol (1.52 g, 9.80 mmol) was dissolved in anhydrous dichloromethane (26 mL) and stirred under an argon atmosphere. Triethylamine (2.73 mL, 19.6 mmol) was added, and the reaction mixture was cooled to 0°C with an ice bath. Acetyl chloride (0.906 mL, 12.74 mmol) was added dropwise to the reaction mixture at 0°C. The ice bath was removed after 5 mins, and reaction was stirred for 2 hrs at room temperature. After this time, brine (20 mL) was added to the reaction and the layers were separated. The aqueous layer was extracted with dichloromethane (3 × 15 mL), and combined organic layers were dried over Na_2SO_3 then gravity filtered. The filtrate was concentrated *in vacuo* to produce an orange oil, 1-methyl-4-piperidyl acetate (1.54 g, 97%). The analytical data was similar to previously published data.[2]

4-Acetoxy-1-methyl-1-piperidinium Chloride: To a flask charged with 1-methyl-4-piperidyl acetate (0.436 g, 2.78 mmol), anhydrous diethyl ether (55 mL) was added until complete dissolution under an argon atmosphere. HCl dissolved in diethyl ether (1 N - 4.16 mL, 4.16 mmol) was added dropwise to the 1-methyl-4-piperidyl acetate solution and stirred for 1h at room temperature. The solvent was removed *in vacuo* resulting in a white solid. The compound was triturated with diethyl ether (3 × 20 mL). Residue diethyl ether was removed *in vacuo* to produce 4-acetoxy-1-methyl-1-piperidinium chloride (0.538 g, 99%) as a pair of isomers

(46:54).

Spectroscopic data:

MP$_{(diethyl\ ether)}$: 82-87°C; IR(ATR) 2958, 2603, 2460, 2427, 1730, 1237, 1037 cm^{-1}; ^{1}H NMR (400 MHz, DMSO-*d6*) δ isomer A: 10.90 (br s, 1H), 4.83-4.75 (m, 1H), 3.45-3.43 (m, 1H), 3.28-3.25 (m, 1H), 3.10-3.00 (m, 2H), 2.68 (d, J = 4.8 Hz, 3H), 2.08-2.01 (m, 2H), 2.01 (s, 3H), 1.92-1.86 (m, 2H), isomer B: 10.77 (br s, 1H), 4.95-4.93 (m, 1H), 3.45-3.43 (m, 1H), 3.28-3.25 (m, 1H), 3.10-3.00 (m, 2H), 2.73 (d, J = 4.9 Hz, 3H), 2.08-2.01 (m, 2H), 2.05 (s, 3H), 1.92-1.86 (m, 2H); ^{13}C NMR (100.7 MHz, DMSO-*d6*) δ isomer a: 169.8 (0), 67.3 (1), 48.7 (2), 41.9 (3), 27.8 (2), 21.0 (3), isomer B: 169.0 (0), 63.7 (1), 51.4 (2), 42.4 (3), 26.5 (2), 21.0 (3); LRMS (ESI^{+}): 158.1 (M^{+}); HRMS (ESI^{+}): calculated for $C_8H_{16}NO_2^+$: 158.1176 amu; found for $C_8H_{16}NO_2^+$: 158.1176 amu; HPLC purity at 210 nm (75% CH_3CN : 10% CH_3OH : 15% aqueous triethylamine [0.1% triethylamine in H_2O], retention time 3.333 mins): >99%. Product was confirmed through comparison of key peaks reported from ^{1}H NMR in the literature.[3]

(**8**) 4-Acetoxy-1-methyl-1-piperidinium Chloride IR(ATR)

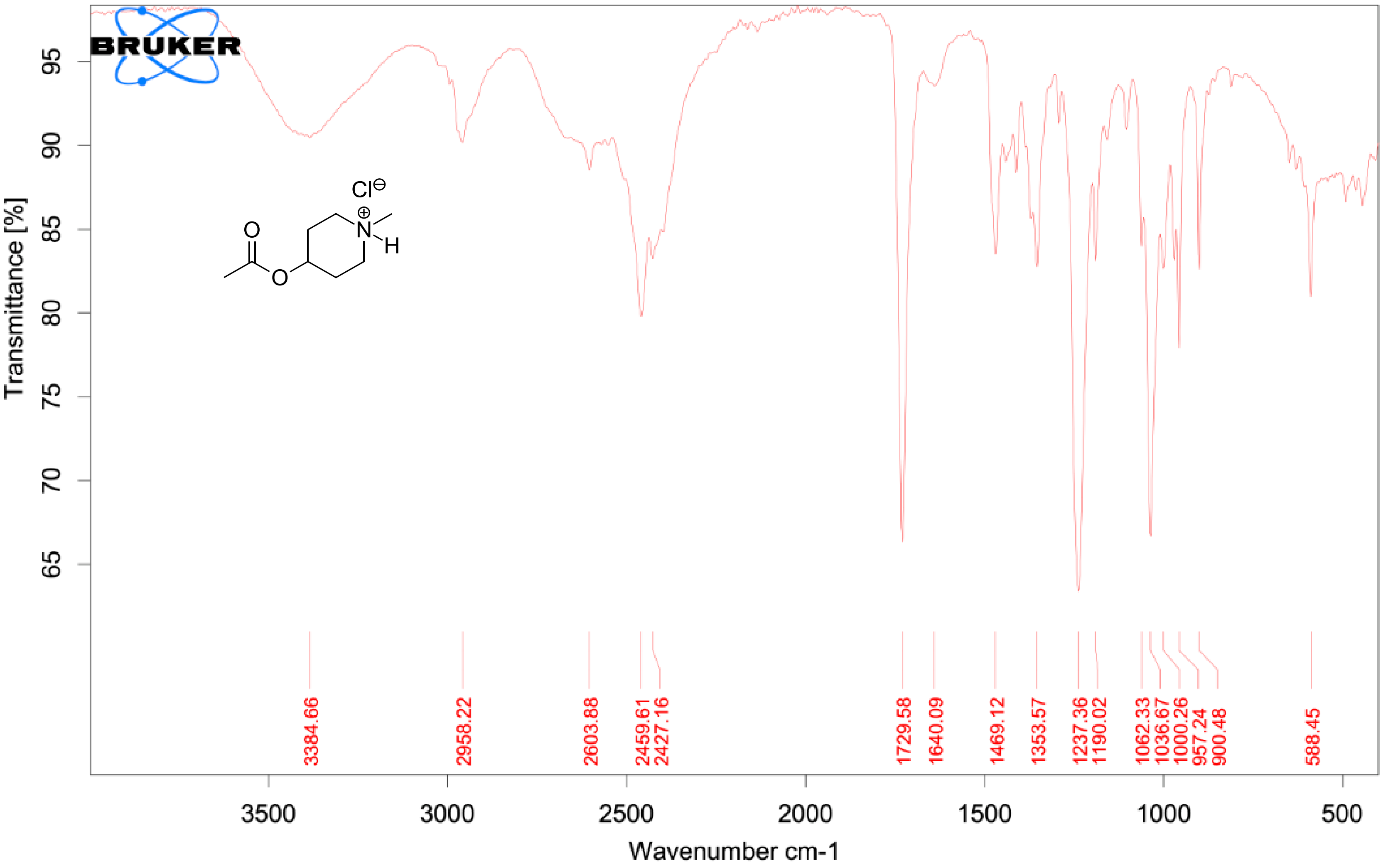

(**8**) 4-Acetoxy-1-methyl-1-piperidinium Chloride ^{1}H NMR 400 MHz (DMSO-*d6*)

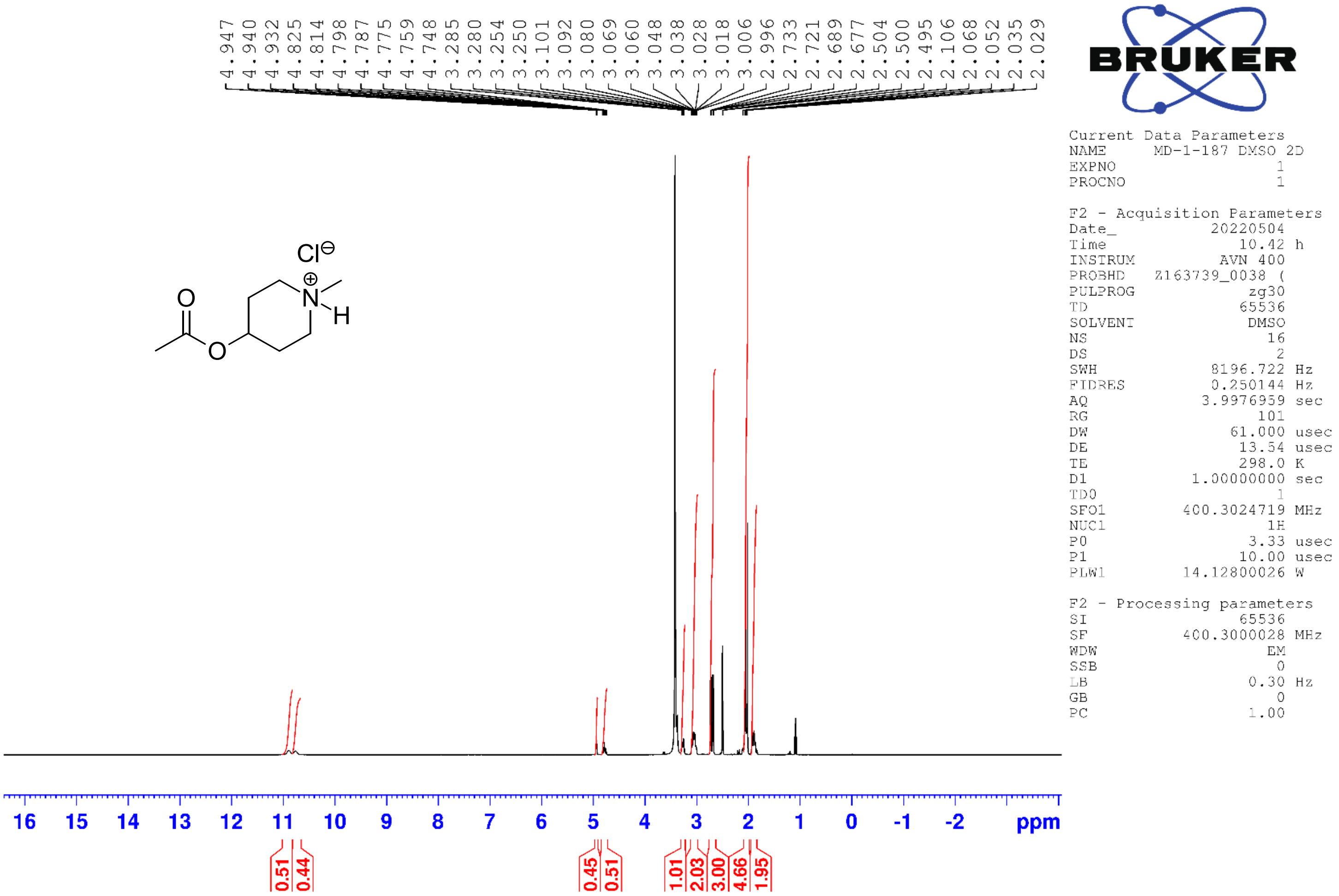

(8) 4-Acetoxy-1-methyl-1-piperidinium Chloride ^{13}C NMR 100 MHz (DMSO-*d6*)

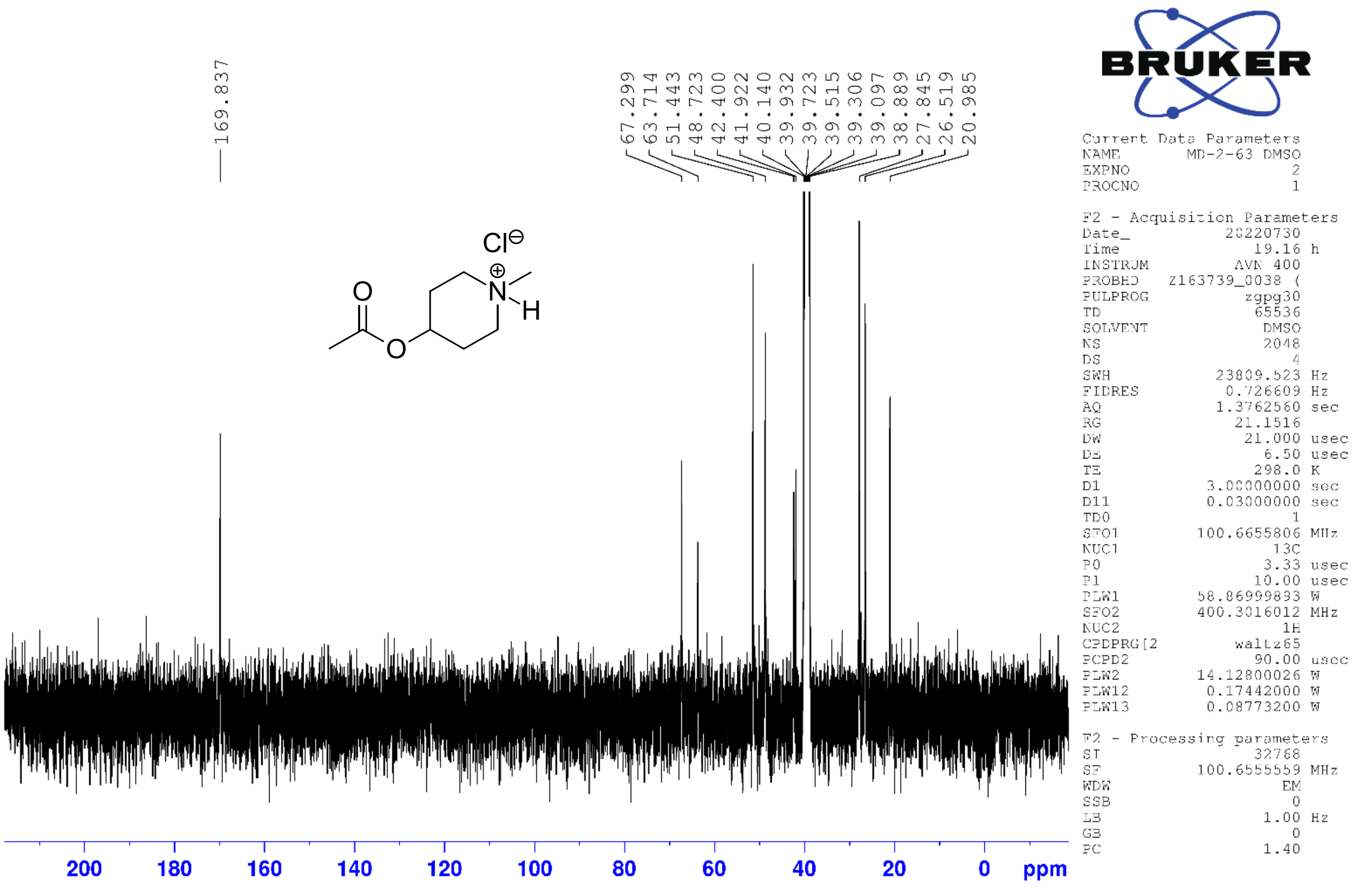

(8) 4-Acetoxy-1-methyl-1-piperidinium Chloride High Resolution Mass Spectrum (ESI, positive mode)

Mass Spectrum SmartFormula Report

Analysis Info

Analysis Name	D:\Data\Xiao\July 07 2022000008.d	Acquisition Date	7/7/2022 9:38:26 AM
Method	Xiao all 1.m	Operator	x
Sample Name	TRV 6014 HCl	Instrument	compact 8255754.20059
Comment			

Acquisition Parameter

Source Type	ESI	Ion Polarity	Positive			Set Nebulizer	0.5 Bar
Focus	Not active	Set Capillary	3500 V			Set Dry Heater	180 °C
Scan Begin	50 m/z	Set End Plate Offset	-500 V			Set Dry Gas	4.0 l/min
Scan End	1500 m/z	Set Charging Voltage	2000 V			Set Divert Valve	Source
		Set Corona	0 nA			Set APCI Heater	0 °C

Meas. m/z	Ion Formula	m/z	err [ppm]
158.1176	C8H16NO2	158.1176	-0.3

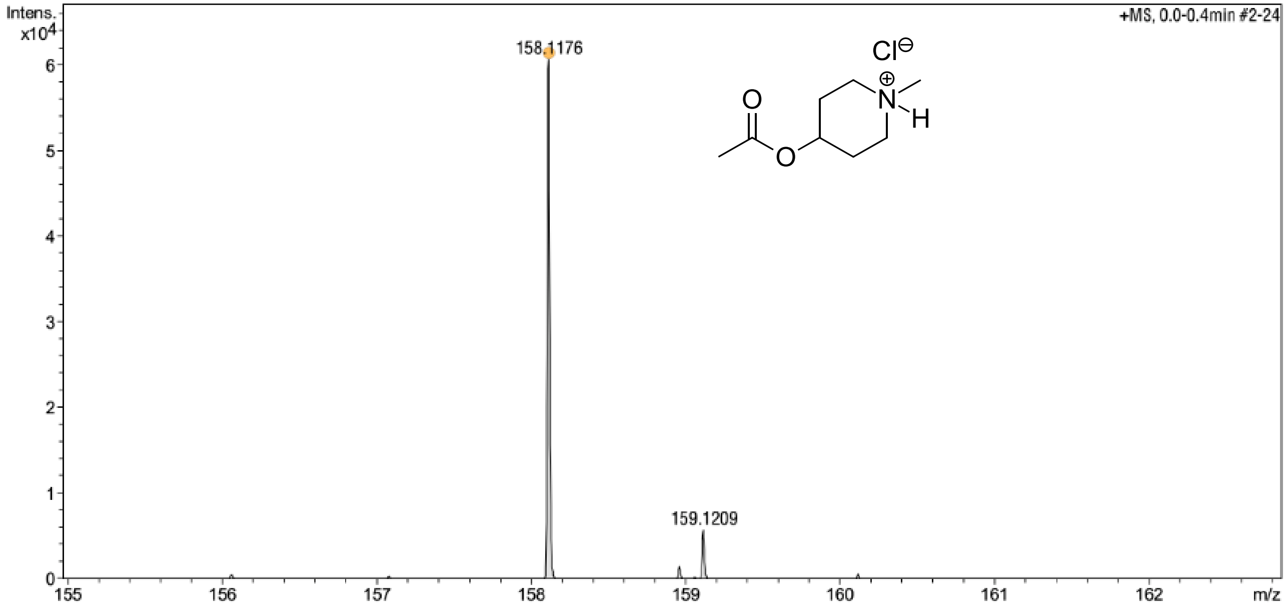

July 07 2022000008.d

Bruker Compass DataAnalysis 4.3 printed: 7/7/2022 9:42:05 AM by: x Page 1 of 1

(8) 4-Acetoxy-1-methyl-1-piperidinium Chloride HPLC. Note: Noise from the injection has a retention time from 1.6 min until 2.5 min. The compound of interest has a retention time of 3.333 min.

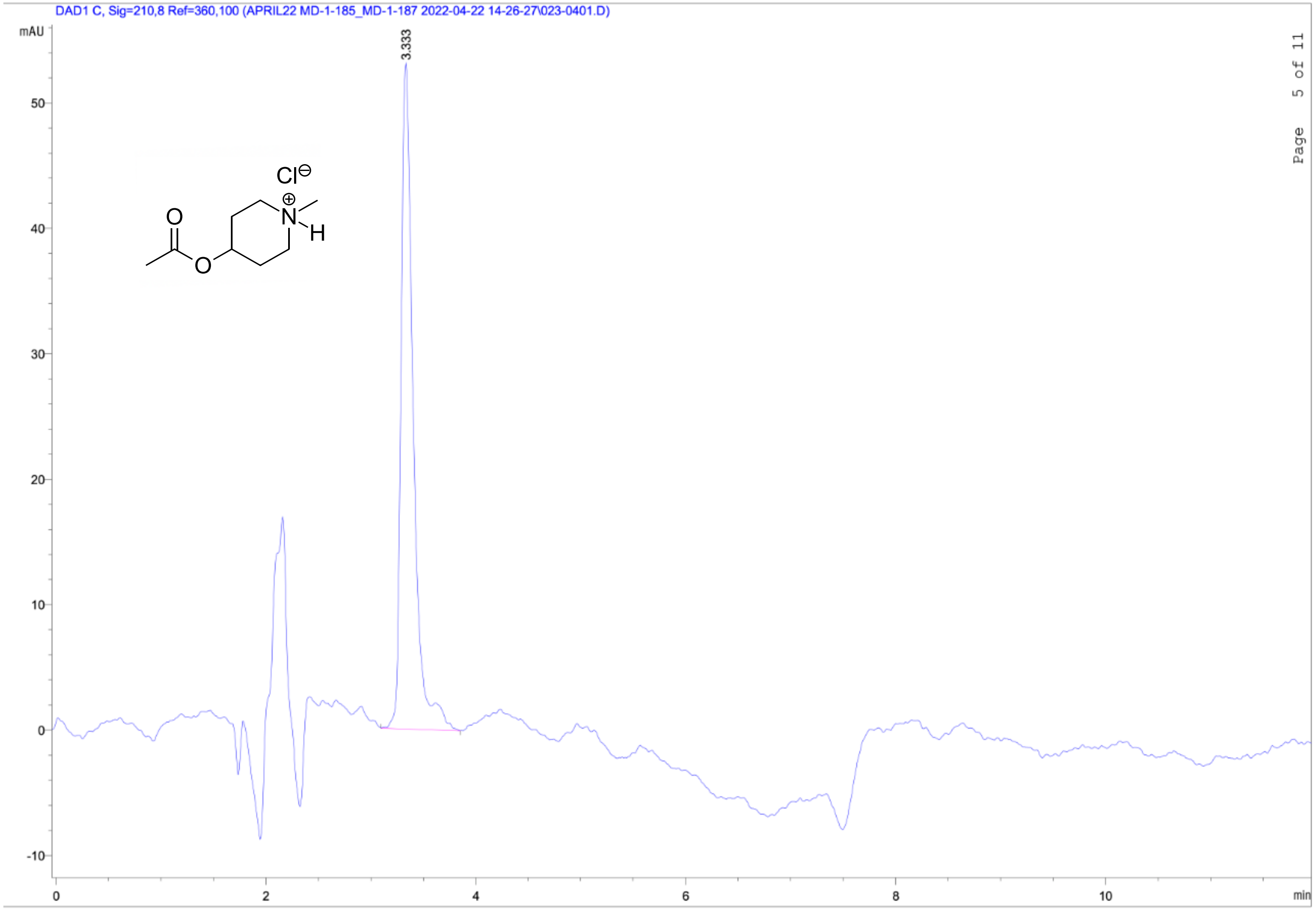

(9) *S*-(1-Methylpiperid-4-yl) Butanethioate Hydrochloride

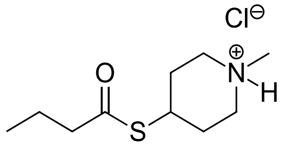

Synthesis: ***S*-(1-Methylpiperid-4-yl) butanethioate**: To a solution of *N*-methyl-4-piperidinethiol (0.50 mL, 4.2 mmol) in anhydrous dichloromethane (5 mL) cooled in an ice bath was added butyryl chloride (1.30 mL, 12.6 mmol) dropwise *via* syringe under an argon atmosphere. After 15 mins, the ice bath was removed and the mixture was allowed to stir for 16 hrs, until TLC indicated complete consumption of starting material. The mixture was diluted with dichloromethane (20 mL). The organic layer was washed with saturated $NaHCO_{3(aq)}$, water and dried over Na_2SO_4. The mixture was gravity filtered and the filtrate was concentrated *in vacuo* to afford 0.846 g of a colorless liquid. Purification *via* flash chromatography (5 % MeOH / DCM) afforded 0.5373 g (64% yield) of **10** as a translucent colorless oil. The analytical data was similar to previously published data.[1]

***S*-(1-Methylpiperid-4-yl) butanethioate, hydrochloride (10)**: *S*-(1-Methylpiperid-4-yl) butanethioate (0.172 g, 0.854 mmol) was dissolved in anhydrous diethyl ether (17 mL) under an argon atmosphere. To this solution, HCl dissolved in diethyl ether (1 N - 1.28 mL, 1.28 mmol) was added dropwise and stirred for 1 hr. The solvent was removed *in vacuo* resulting in a white solid. The compound was triturated with diethyl ether (20 mL), and solvent was removed from the solid with a pipet. The process was repeated twice. The compound was dried *in vacuo* to produce **10** (0.180 g, 89%) as a white solid.

Spectroscopic data: $MP_{(diethyl\ ether)}$: 142−146°C; IR (ATR) 3010, 2963, 2948, 2481, 1691, 1113, 998, 770; ^{1}H NMR (400 MHz, DMSO-*d6*) d Isomer A: 10.71 (br s, 1H), 3.52-3.46 (m, 1H),

3.40-3.37 (m, 2H), 3.10-3.02 (m, 2H), 2.68 (s, 3H), 2.56 (t, J = 7.2 Hz, 2H), 2.02-1.99 (m, 2H), 1.93-1.82 (m, 2H), 1.58 (sext, J = 7.3 Hz, 2H), 0.88 (t, J = 7.4 Hz, 3H), isomer B: 10.71 (br s, 1H), 3.86 (app br s, 1H), 3.40-3.37 (m, 2H), 2.97-2.90 (m, 2H), 2.73 (s, 3H), 2.56 (t, J = 7.2 Hz, 2H), 2.29, 2.22 (m, 2H), 1.93-1.82 (m, 2H), 1.58 (sext, J = 7.3 Hz, 2H), 0.88 (t, J = 7.4 Hz, 3H); ^{13}C NMR (100.7 MHz, DMSO-*d6*) d isomer A: 198.0 (0), 53.1 (2), 45.3 (2), 42.4 (3), 36.4 (1), 28.9 (2), 18.6 (2), 13.2 (3), isomer B: 198.0 (0), 50.2 (2), 45.6 (2), 41.7 (3), 35.8 (1), 27.3 (2), 18.6 (2), 13.2 (3); LRMS (ESI$^+$): 202.1 (M+); HRMS (ESI$^+$): calculated for $C_{10}H_{20}NOS^+$: 202.1260; found for $C_{10}H_{20}NOS^+$: 202.1261; HPLC purity at 254nm (75% CH_3CN : 10% CH_3OH : 15% aqueous triethylamine [0.1% triethylamine in H_2O], retention time 5.212 mins): 99.1%.

(9) *S*-(1-Methylpiperid-4-yl) Butanethioate Hydrochloride IR(ATR)

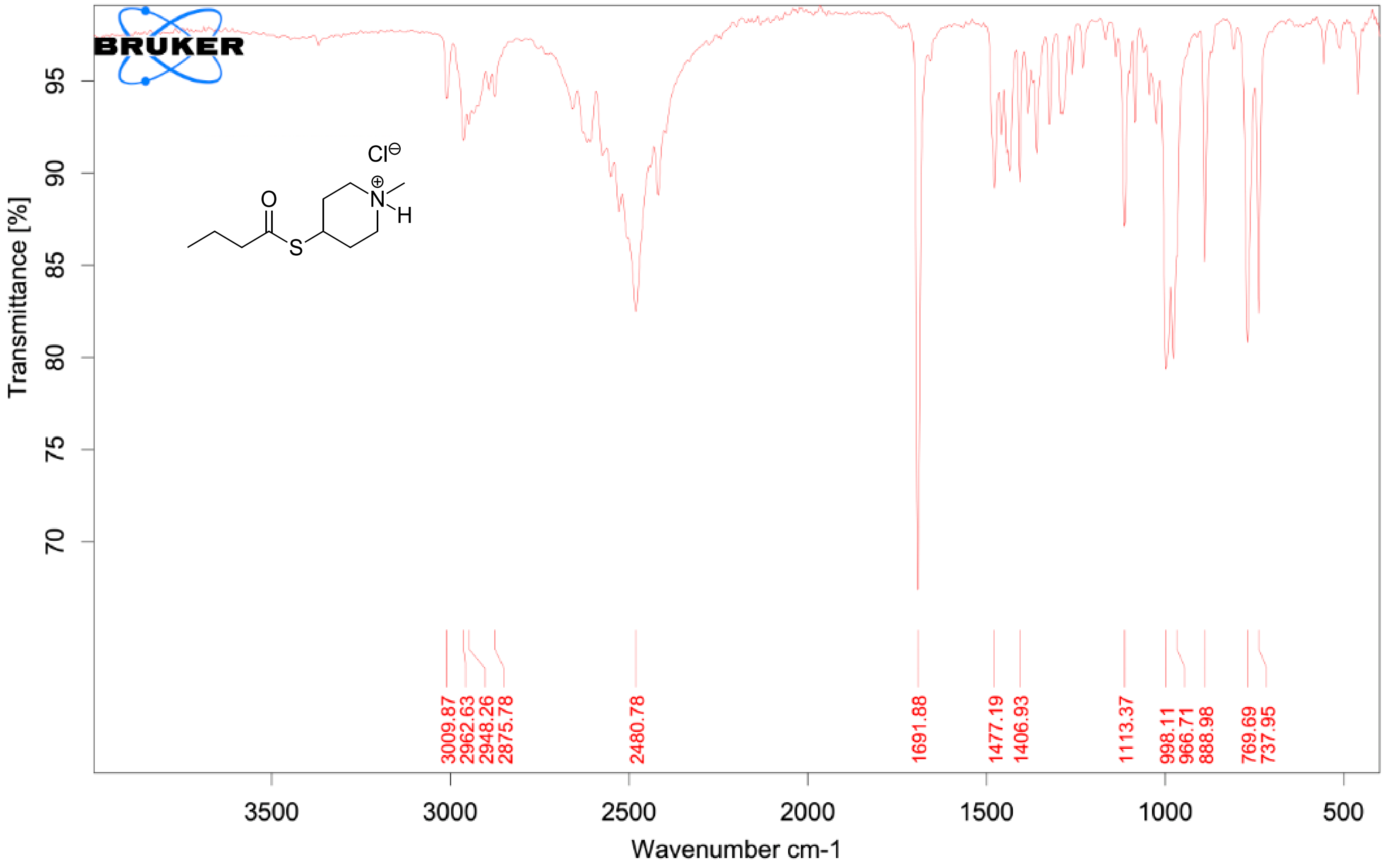

(9) *S*-(1-Methylpiperid-4-yl) Butanethioate Hydrochloride ^{1}H NMR 400 MHz (DMSO-*d6*)

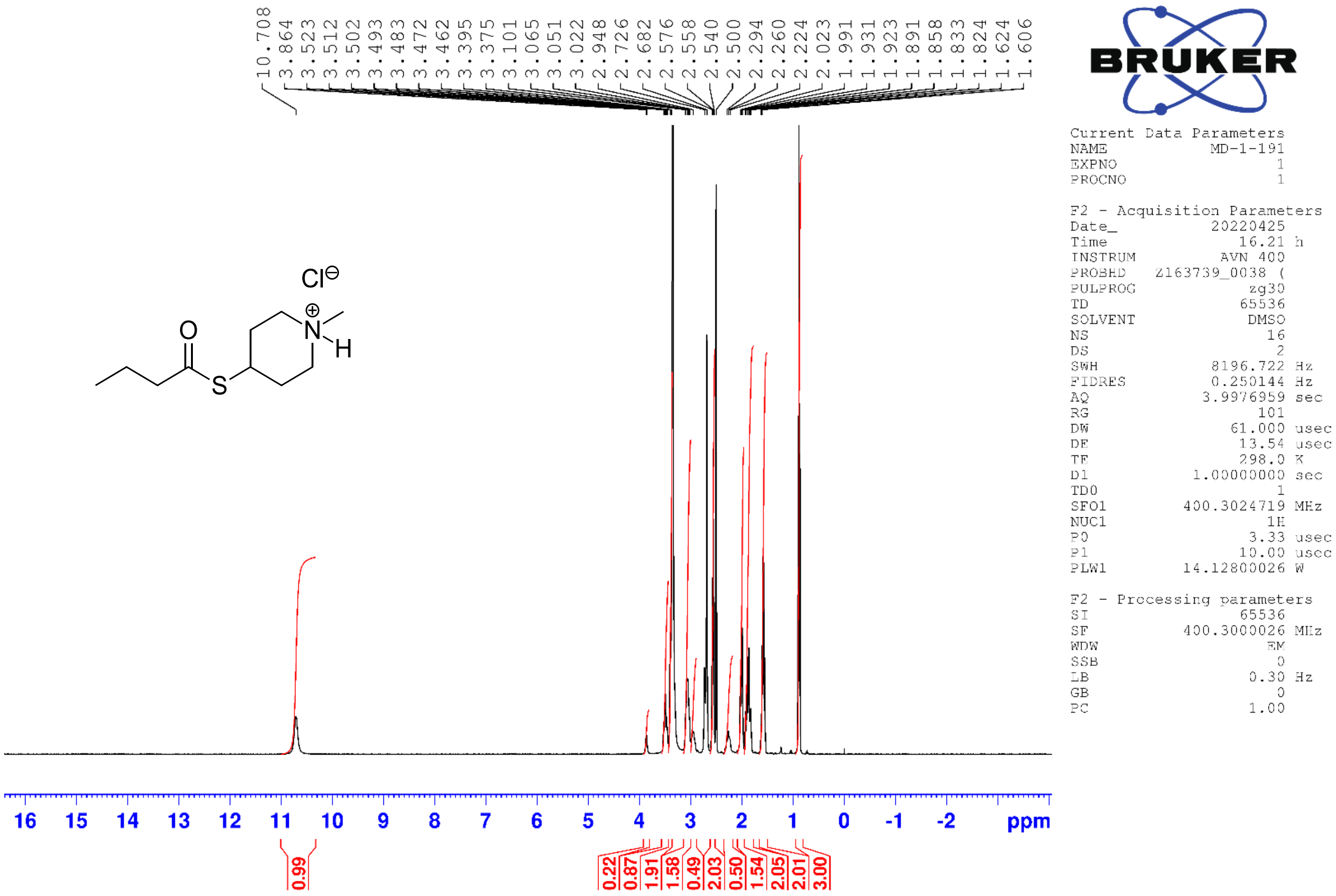

(9) *S*-(1-Methylpiperid-4-yl) Butanethioate Hydrochloride ^{13}C NMR 100 MHz (DMSO-*d6*)

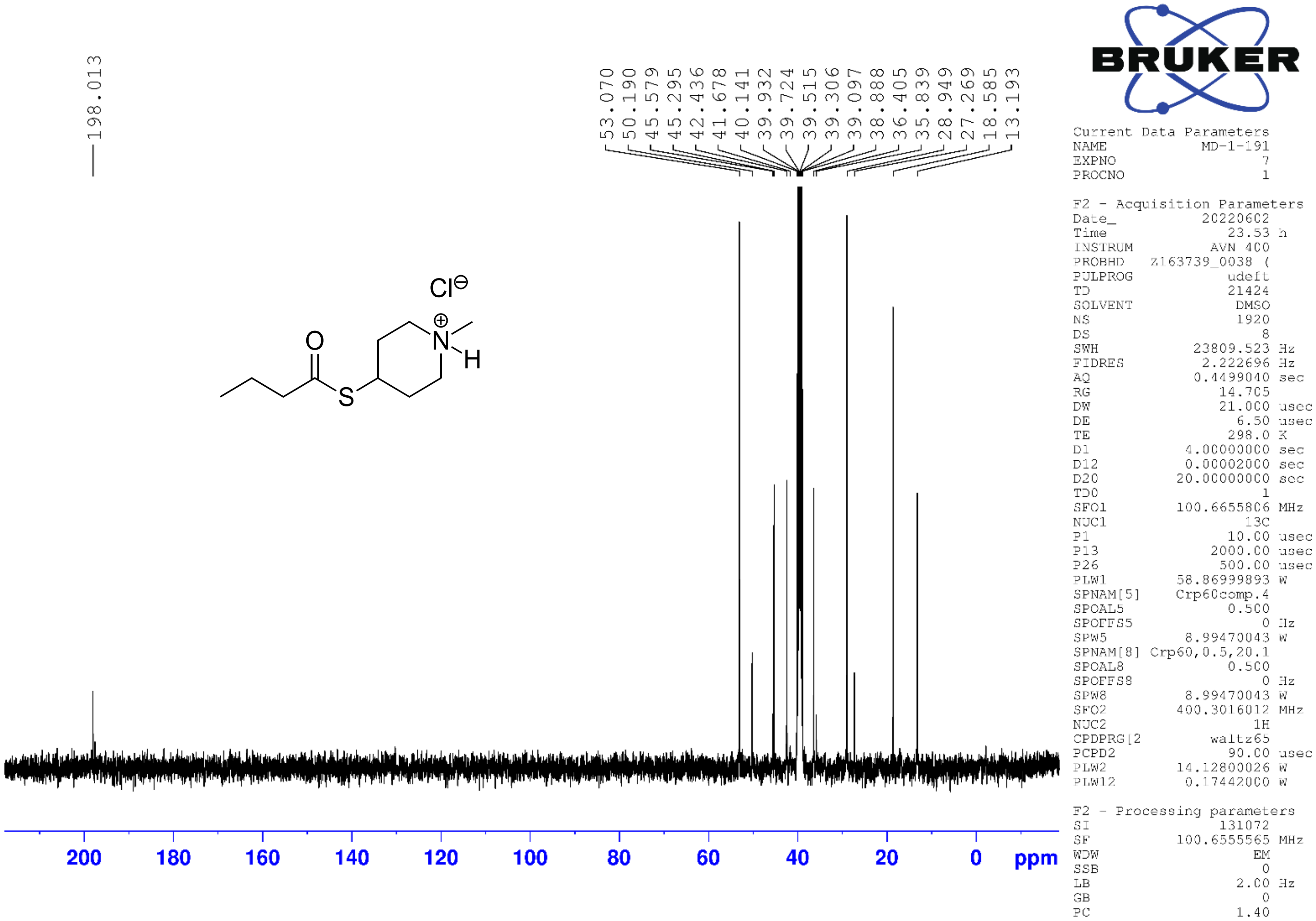

(9) *S*-(1-Methylpiperid-4-yl) Butanethioate Hydrochloride High Resolution Mass Spectrum (ESI, positive mode)

Mass Spectrum SmartFormula Report

Analysis Info

Analysis Name	D:\Data\Xiao\July 07 2022000004.d	Acquisition Date	7/7/2022 9:17:31 AM
Method	Xiao all 1.m	Operator	x
Sample Name	TRV 6010 HCl	Instrument	compact 8255754.20059
Comment			

Acquisition Parameter

Source Type	ESI	Ion Polarity	Positive	Set Nebulizer	0.5 Bar
Focus	Not active	Set Capillary	3500 V	Set Dry Heater	180 °C
Scan Begin	50 m/z	Set End Plate Offset	-500 V	Set Dry Gas	4.0 l/min
Scan End	1500 m/z	Set Charging Voltage	2000 V	Set Divert Valve	Source
		Set Corona	0 nA	Set APCI Heater	0 °C

Meas. m/z	Ion Formula	m/z	err [ppm]
202.1261	C10H20NOS	202.1260	-0.4

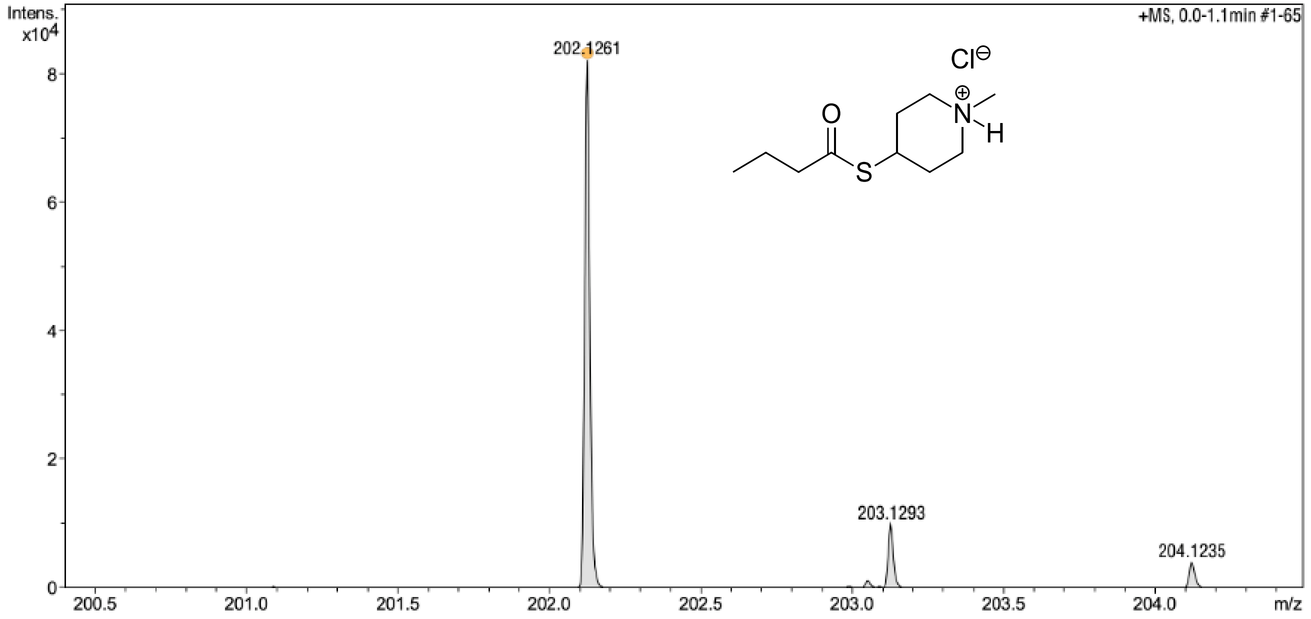

July 07 2022000004.d

Bruker Compass DataAnalysis 4.3 printed: 7/7/2022 9:20:10 AM by: x Page 1 of 1

(9) *S*-(1-Methylpiperid-4-yl) Butanethioate Hydrochloride HPLC

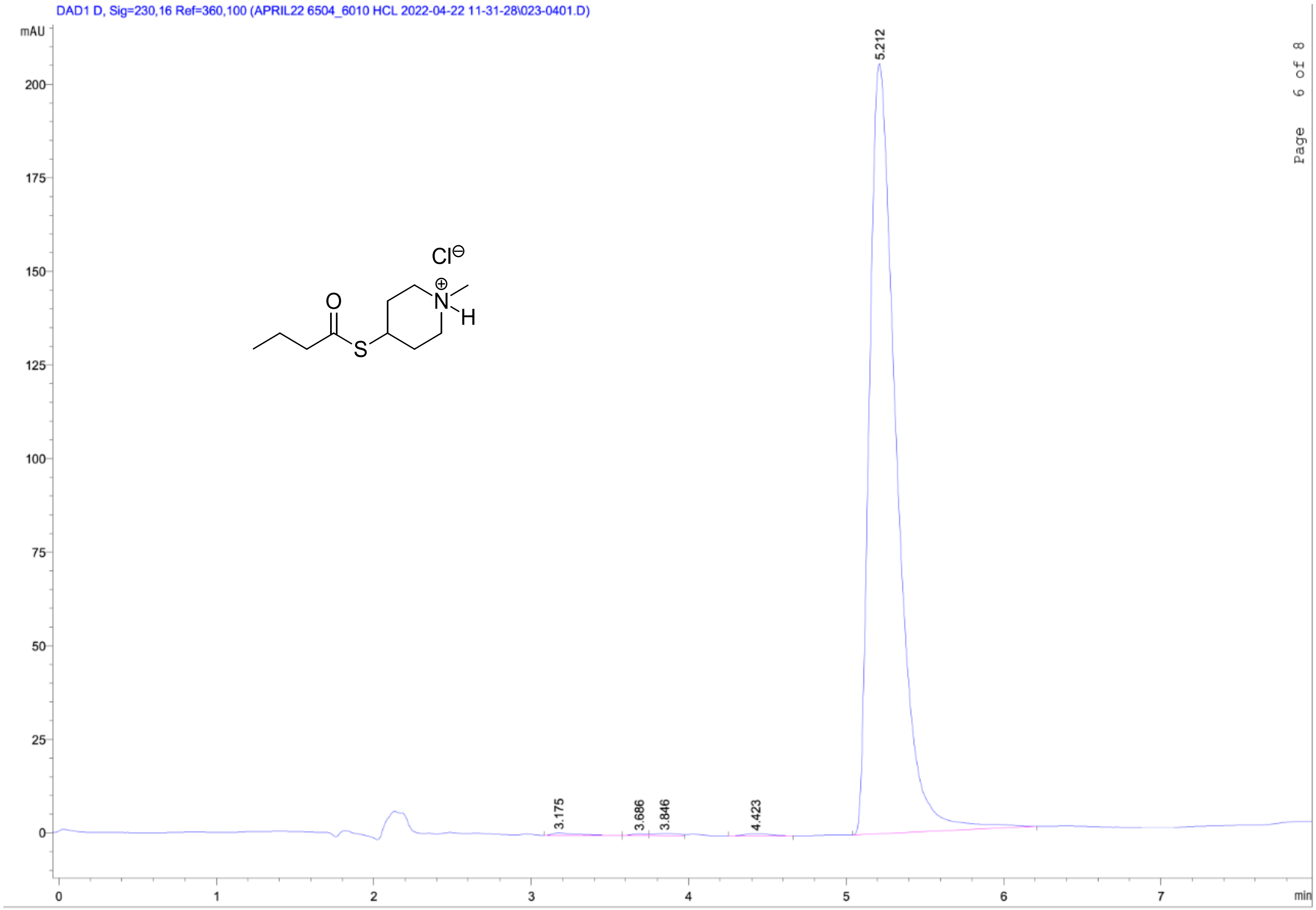

(10) 4-Butyroxy-1-methyl-1-piperidinium Chloride

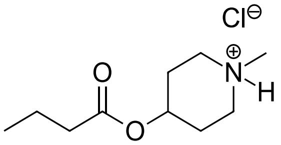

Synthesis:

***N*-Methylpiperidin-4-yl Butyrate**: *N*-Methyl-4-piperidinol (0.803 g, 5.18 mmol) was dissolved in anhydrous dichloromethane (10 mL) and stirred under an argon atmosphere. Triethylamine (1.44 mL, 10.4 mmol) was added, and the reaction was cooled to 0°C with an ice bath. Butyryl chloride (0.699 mL, 6.73 mmol) was added dropwise at 0°C. The ice bath was removed after 5 mins, and reaction was stirred for 3 hrs. The reaction was quenched with brine (20 mL), extracted with dichloromethane (3 × 15 mL) and dried over Na_2SO_3. The solvent was removed *in vacuo* to produce a yellow oil. The crude mixture was purified by silica gel column chromatography (5% MeOH/DCM) to produce a yellow oil, *N*-methylpiperidin-4-yl butyrate (0.954 g, 99%). The analytical data was similar to previously published data.[4]

4-Butyroxy-1-methyl-1-piperidinium chloride (9): To a flask charged with *N*-methylpiperidin-4-yl butyrate (0.48 g, 2.59 mmol) anhydrous diethyl ether was added until complete dissolution (52 mL) under an argon atmosphere. HCl in diethyl ether (1 N - 3.88 mL, 3.88 mmol) was added dropwise and stirred for 1 hr. The solvent was removed *in vacuo* resulting in a white solid. The compound was triturated with diethyl ether (20 mL), and solvent was removed from the solid with a pipet. This process was repeated two more times. The compound was dried *in vacuo* to produce 4-butyroxy-1-methyl-1-piperidinium chloride (0.583 g, 90%) as a white solid as a pair of isomers (46:54).

Spectroscopic data: MP$_{(diethyl\ ether)}$: 110–113°C; IR(ATR) 2957, 2437, 1724, 1244, 1195, 963 cm^{-1}; ^{1}H NMR (400 MHz, DMSO-*d6*) δ isomer A: 11.12 (br s, 1H), 4.98-4.95 (m, 1H), 3.41-3.77 (m, 2H), 3.10-3.00 (m, 2H), 2.72 (s, 3H), 2.33-2.26 (m, 2H), 2.14-2.06 (m, 2H), 1.92-1.85 (m, 2H), 1.60-1.51 (m, 2H), 0.89-0.86 (m, 3H) isomer B: 11.03 (br s, 1H), 4.84-4.79 (m, 1H), 3.27-3.25 (m, 2H), 3.10-3.00 (m, 2H), 2.68 (s, 3H), 2.33-2.26 (m, 2H), 2.06-2.2.02 (m, 2H), 1.92-1.85 (m, 2H), 1.60-1.51 (m, 2H), 0.89-0.86 (m, 3H); ^{13}C NMR (100.7 MHz, DMSO-*d6*) δ isomer a: 172.0 (0), 63.5(1), 48.6 (2), 42.3 (3), 35.6 (2), 26.5 (2), 17.9 (2), 13.4 (3), isomer B: 172.2 (0), 67.2 (1), 51.3 (2), 41.8 (3), 35.4 (2), 27.8 (2), 17.9 (2), 13.3 (3); LRMS (ESI^+): 186.1 (M^+); HRMS (ESI^+): calculated for $C_{10}H_{20}NO_2^+$: 186.1488 amu; found for $C_{10}H_{20}NO_2^+$: 186.1492 amu; HPLC purity at 210 nm (75% CH_3CN : 10% CH_3OH : 15% aqueous triethylamine [0.1% triethylamine in H_2O], retention time 4.406 mins): >99%.

(10) 4-Butyroxy-1-methyl-1-piperidinium Chloride IR(ATR)

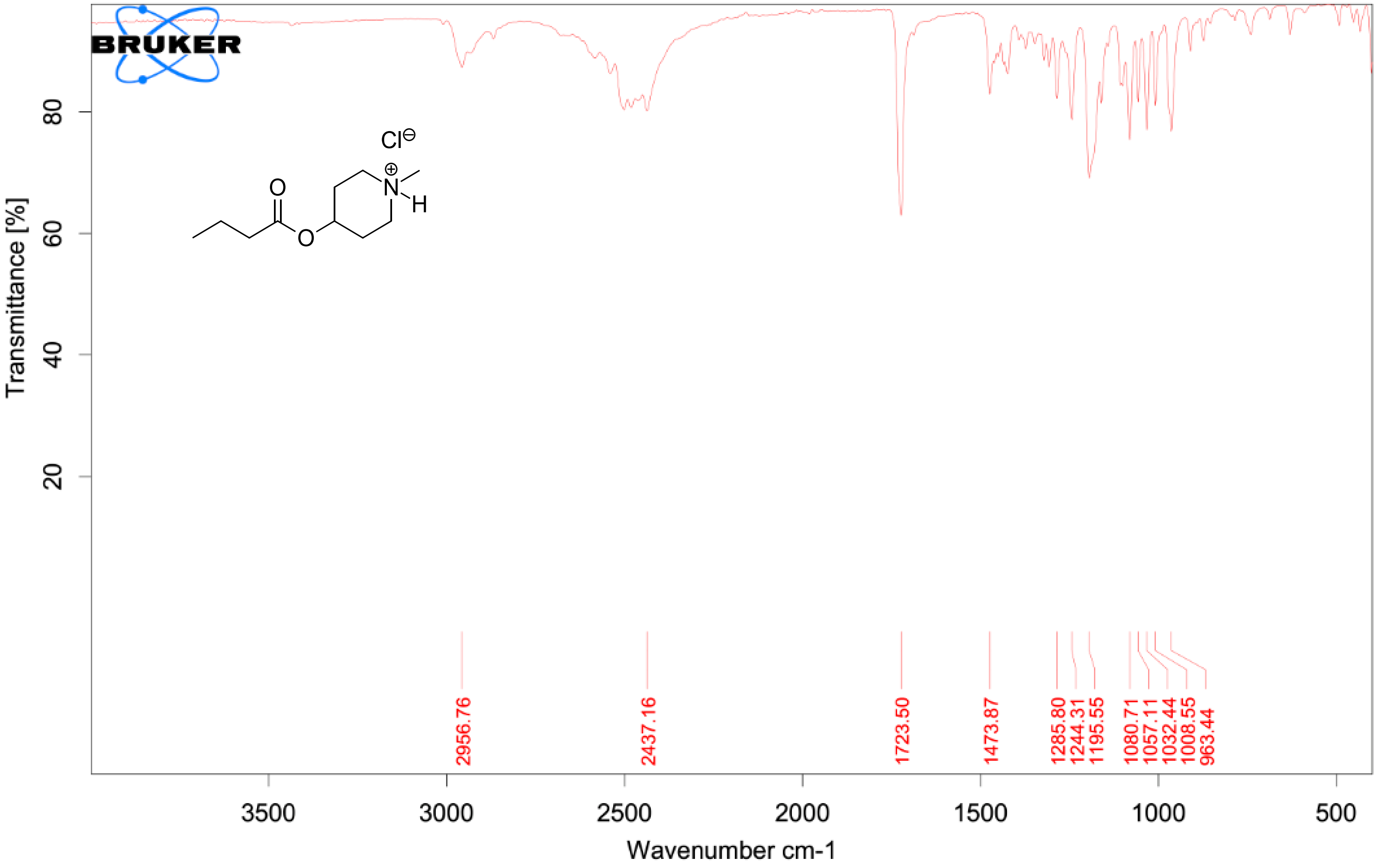

(10) 4-Butyroxy-1-methyl-1-piperidinium Chloride ^{1}H NMR 400 MHz (DMSO-*d6*)

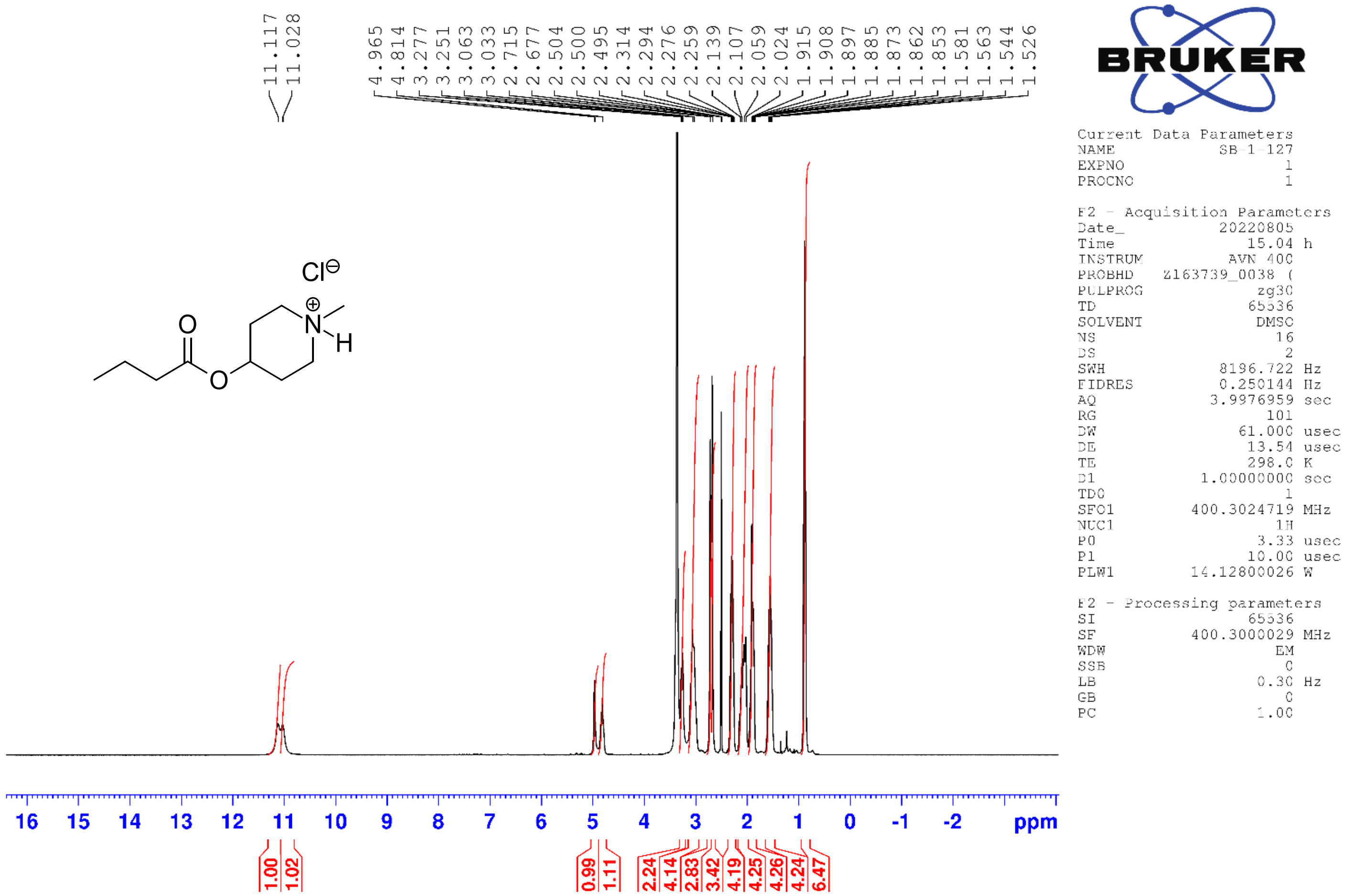

(10) 4-Butyroxy-1-methyl-1-piperidinium Chloride ^{13}C NMR 100 MHz (DMSO-*d6*)

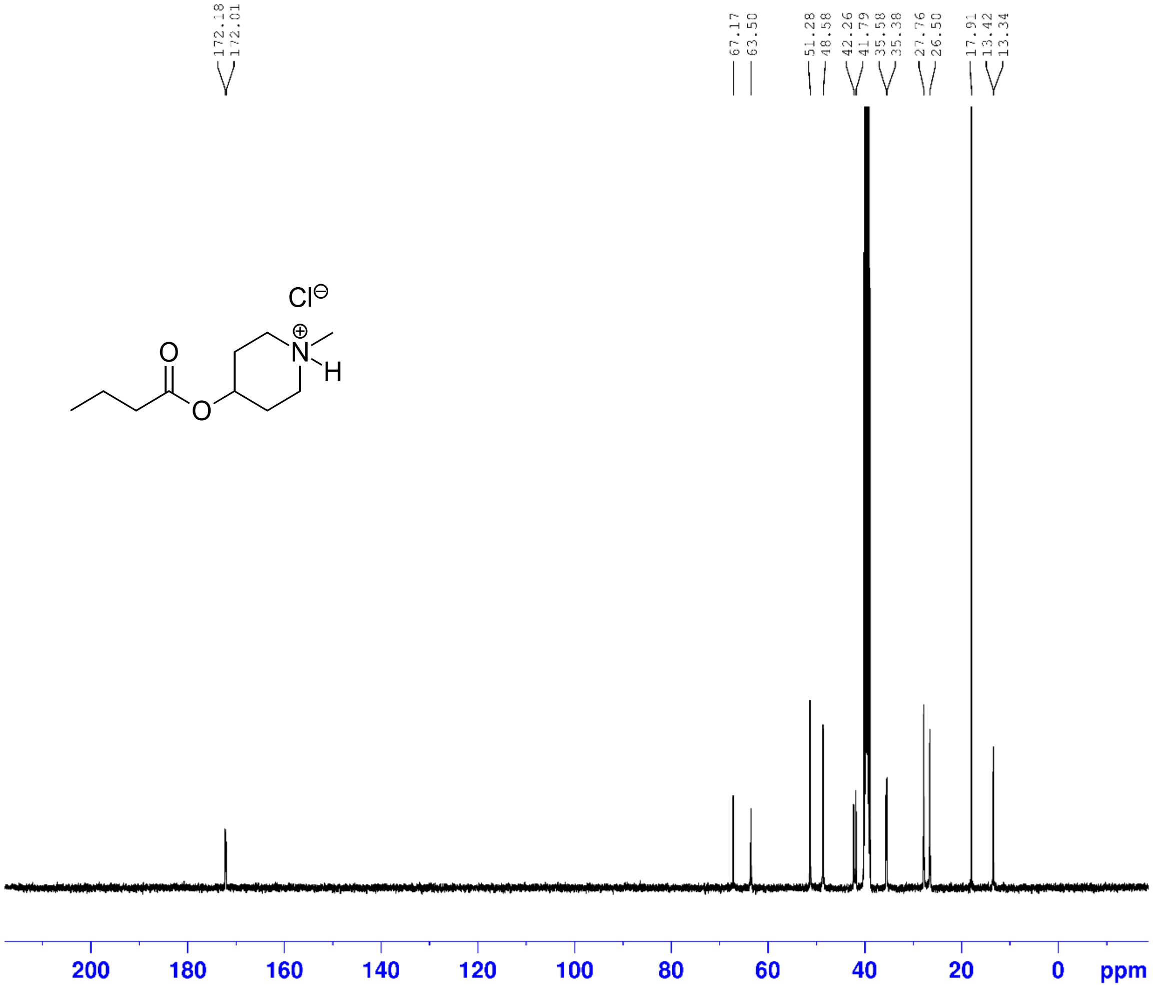


```
Current Data Parameters
NAME              SB-1-127
EXPNO                    2
PROCNO                   1

F2 - Acquisition Parameters
Date_             20220806
Time                 17.16 h
INSTRUM            AVN 400
PROBHD    Z163739_0038 (
PULPROG            zgpg30
TD                  65536
SOLVENT              DMSO
NS                   6144
DS                      4
SWH             23809.523 Hz
FIDRES           0.726609 Hz
AQ              1.3762560 sec
RG                25.1533
DW                 21.000 usec
DE                   6.50 usec
TE                  298.0 K
D1             3.00000000 sec
D11            0.03000000 sec
TD0                     1
SFO1          100.6655806 MHz
NUC1                  13C
P0                   3.33 usec
P1                  10.00 usec
PLW1          58.86999893 W
SFO2          400.3016012 MHz
NUC2                   1H
CPDPRG[2          waltz65
PCPD2               90.00 usec
PLW2          14.12800026 W
PLW12          0.17442000 W
PLW13          0.08773200 W

F2 - Processing parameters
SI                  32768
SF            100.6555612 MHz
WDW                    EM
SSB                     0
LB                   1.00 Hz
GB                      0
PC                   1.40
```

(10) 4-Butyroxy-1-methyl-1-piperidinium Chloride High Resolution Mass Spectrum (ESI, positive mode)

Mass Spectrum SmartFormula Report

Analysis Info

Analysis Name	D:\Data\Xiao\July 07 2022000006.d	Acquisition Date	7/7/2022 9:25:32 AM
Method	Xiao all 1.m	Operator	x
Sample Name	TRV 6013 HCl	Instrument	compact 8255754.20059
Comment			

Acquisition Parameter

Source Type	ESI	Ion Polarity	Positive	Set Nebulizer	0.5 Bar
Focus	Not active	Set Capillary	3500 V	Set Dry Heater	180 °C
Scan Begin	50 m/z	Set End Plate Offset	-500 V	Set Dry Gas	4.0 l/min
Scan End	1500 m/z	Set Charging Voltage	2000 V	Set Divert Valve	Source
		Set Corona	0 nA	Set APCI Heater	0 °C

Meas. m/z	Ion Formula	m/z	err [ppm]
186.1492	C10H20NO2	186.1489	-2.0

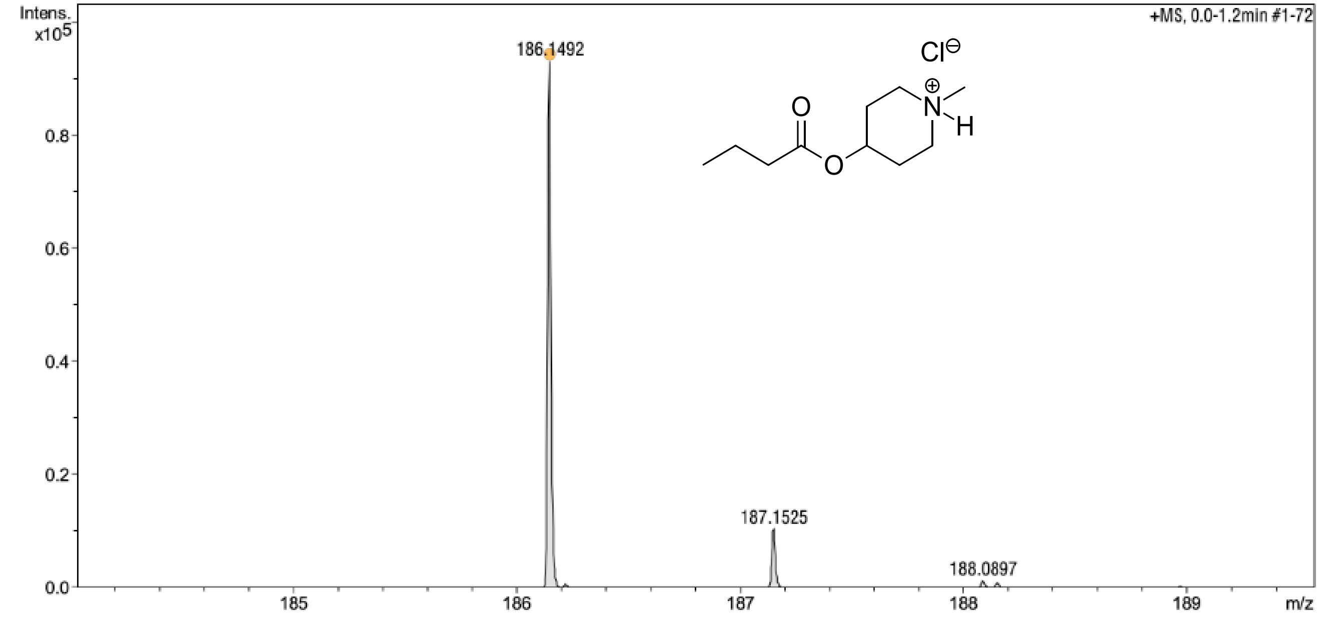

July 07 2022000006.d

Bruker Compass DataAnalysis 4.3 printed: 7/7/2022 9:28:17 AM by: x Page 1 of 1

(10) 4-Butyroxy-1-methyl-1-piperidinium Chloride HPLC. Note: Noise from the injection has a retention time from 1.6 min until 2.5 min. The compound of interest has a retention time of 4.406 min.

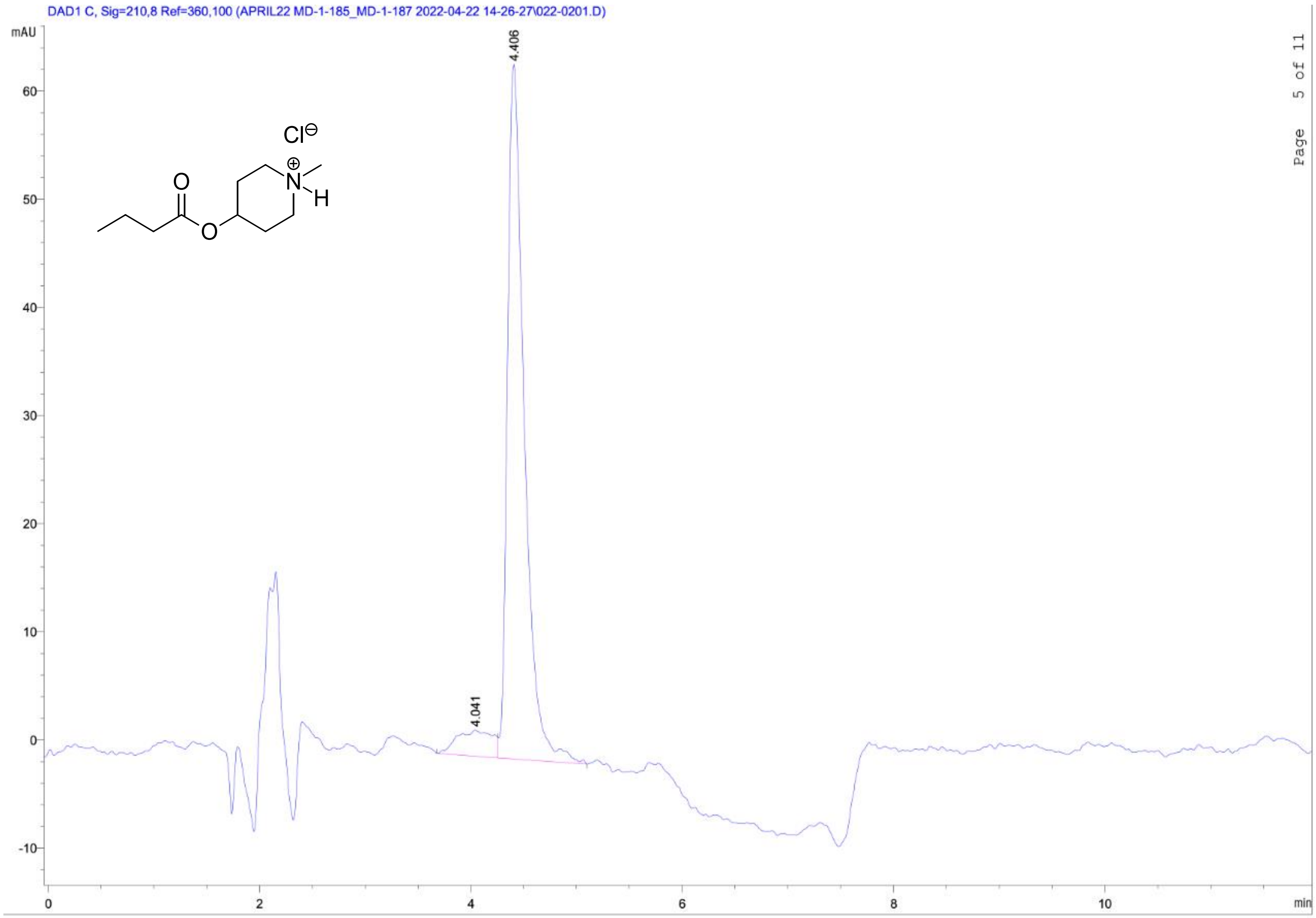

Aryl N-Methylpiperidinyl Derivatives

(11) (1-Methylpiperidin-4-yl) *p*-Iodobenzenecarbothioate Hydrochloride

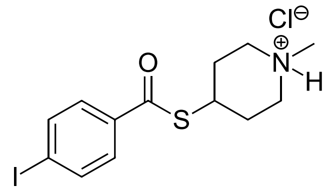

Synthesis: **(1-Methylpiperidin-4-yl) *p*-iodobenzenecarbothioate**: *N*-methyl-4-piperidinethiol (0.538 g, 4.1 mmol) and triethylamine (0.90 mL, 6.2 mmol) were dissolved in anhydrous dichloromethane (10 mL) under an argon atmosphere. 4-Iodobenzoyl chloride (1.31 g, 4.9 mmol) was added at 0°C. The ice bath was removed after 5 mins and the reaction was stirred for 16 hrs at room temperature. The reaction was quenched with brine (20 mL), extracted with dichloromethane (3 × 15 mL), and the combined organic layers were dried over Na_2SO_3. After gravity filtration, the solvent was removed *in vacuo* to produce 1.49 g of a brown solid. The crude mixture was purified by silica gel column chromatography (step gradient: 5% MeOH/DCM to 10 % MeOH/DCM) to produce a white solid, (1-methylpiperidin-4-yl) *p*-iodobenzenecarbothioate (0.644 g, 43% yield). The analytical data was similar to previously published data.[1]

(1-Methylpiperidin-4-yl) *p*-iodobenzenecarbothioate hydrochloride (12): (1-methylpiperidin-4-yl) *p*-iodobenzenecarbothioate (0.47 g, 1.3 mmol) was dissolved in anhydrous diethyl ether (86 mL) under an argon atmosphere. HCl in diethyl ether (1 N solution, 2.0 mL, 2.0 mmol) was added dropwise resulting in the immediate formation of a white precipitate. The reaction was stirred for an additional 60 mins. The white solid was collected *via*

suction filtration, washed with Et_2O (2 × 30 mL), to afford (1-methylpiperidin-4-yl) *p*-iodobenzenecarbothioate hydrochloride, (0.450 g, 87% yield) as a white solid.

Spectroscopic data: $MP_{(diethyl\ ether)}$: >250°C; IR(ATR) 3083, 3068, 3038, 2975, 2951, 2436, 2393, 1655, 1578, 1465, 918; ^{1}H NMR (400 MHz, CD_3OD) δ 7.93-7.91 (m, 2H), 7.70-7.68 (m, 2H), 3.94-3.80 (m, 1H), 3.62-3.47 (m, 2H), 3.34-3.18 (m, 2H), 2.90 (s, 3H), 2.34-2.31 (m, 2H), 2.09-1.98 (m, 2H); ^{13}C NMR (100.7 MHz, CD_3OD) δ 191.4, 139.5, 137.3, 129.5, 102.5, 55.4, 43.8, 37.9, 30.6; LRMS (ESI^+): 362.0 (M^+); HRMS (ESI^+): calculated for $C_{13}H_{17}INOS^+$: 362.0070 amu; found for $C_{13}H_{17}INOS^+$: 362.0073 amu; HPLC purity at 254nm (75% CH_3CN : 10% CH_3OH : 15% aqueous triethylamine [0.1% triethylamine in H_2O], retention time 11.364 mins): 98.2 %.

(11) (1-Methylpiperidin-4-yl) *p*-Iodobenzenecarbothioate Hydrochloride IR(ATR)

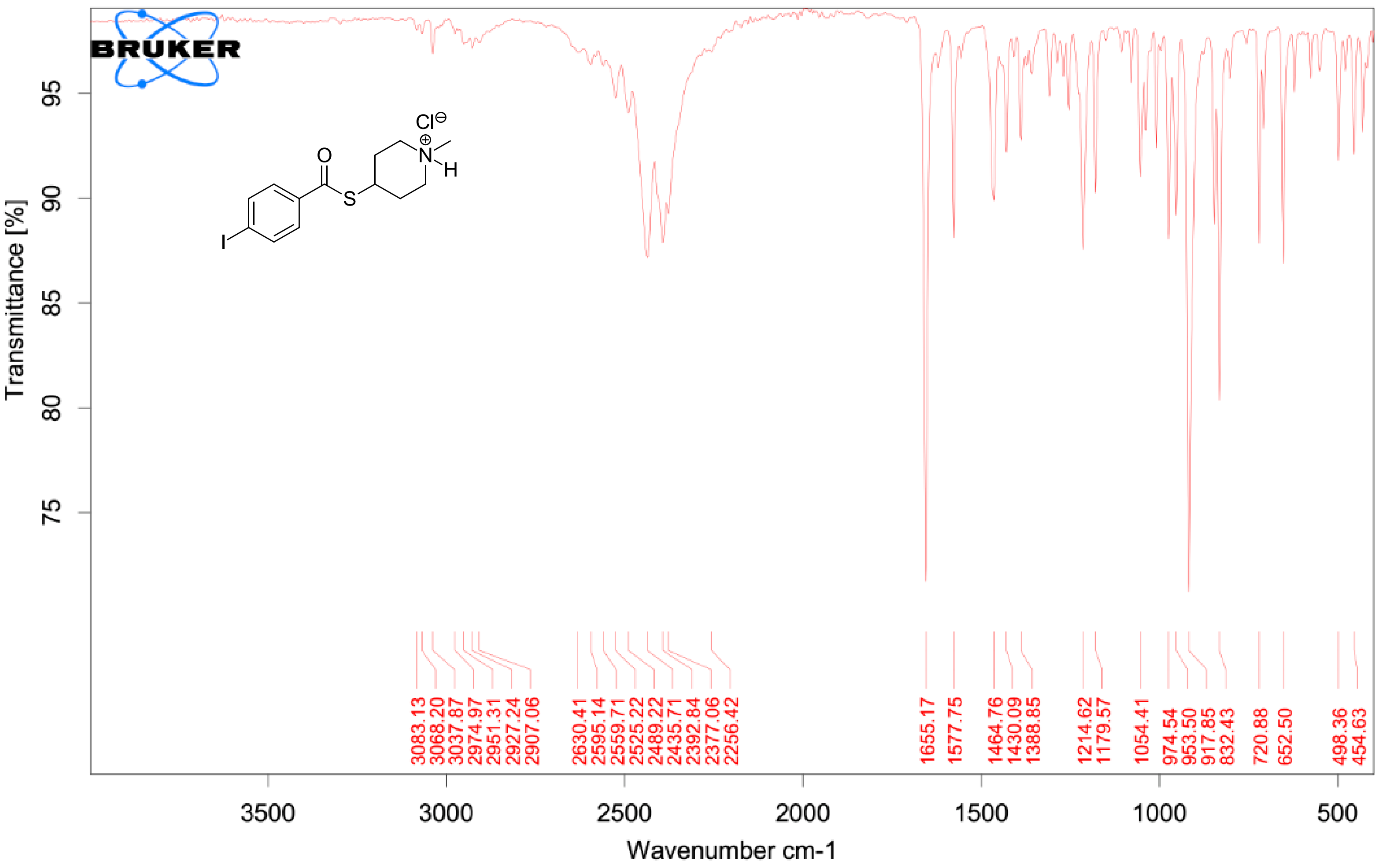

(11) (1-Methylpiperidin-4-yl) *p*-Iodobenzenecarbothioate Hydrochloride ^{1}H NMR 400 MHz (CD_3OD-*d4*)

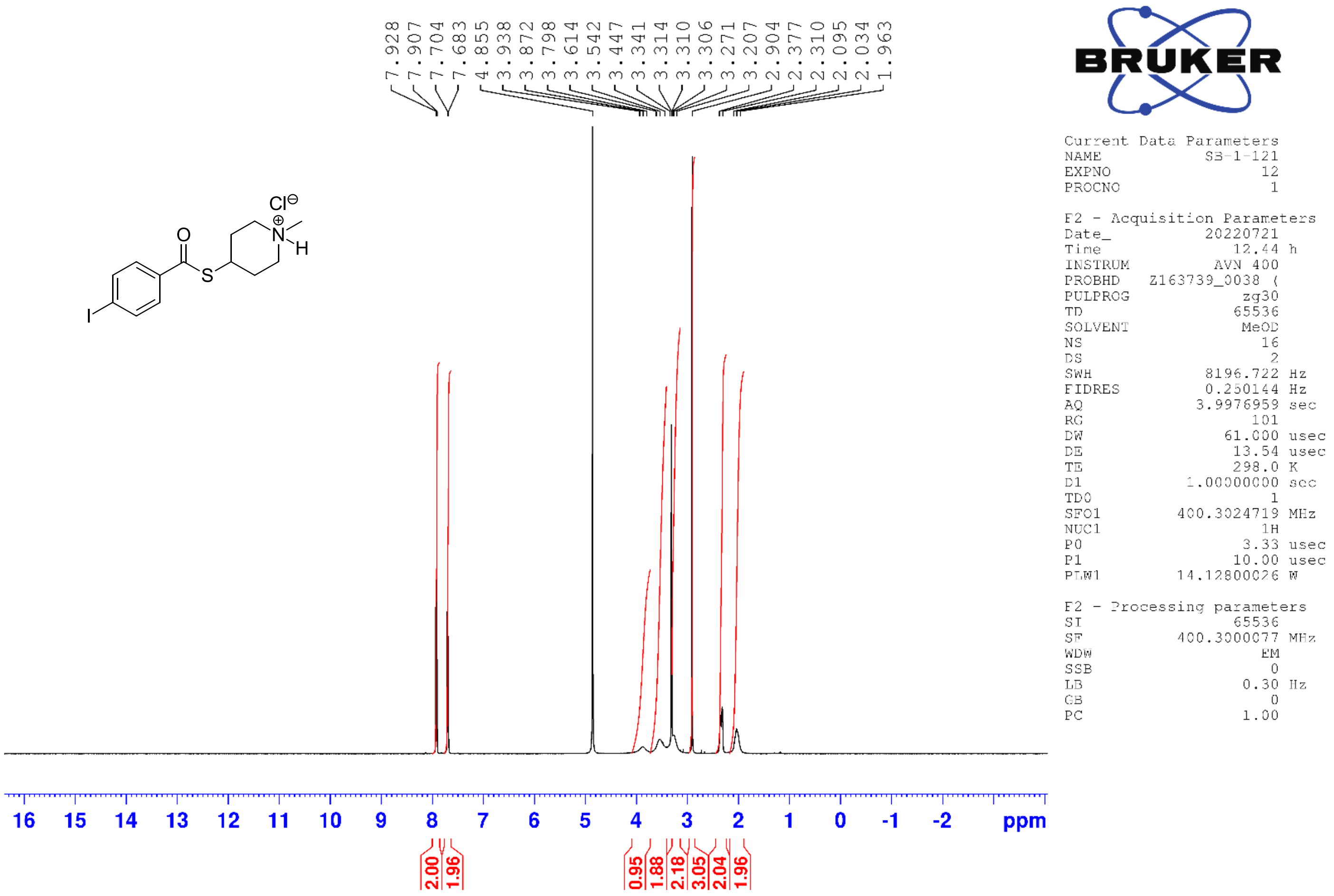

(11) (1-Methylpiperidin-4-yl) *p*-Iodobenzenecarbothioate Hydrochloride ^{13}C NMR 100 MHz (CD_3OD-*d4*)

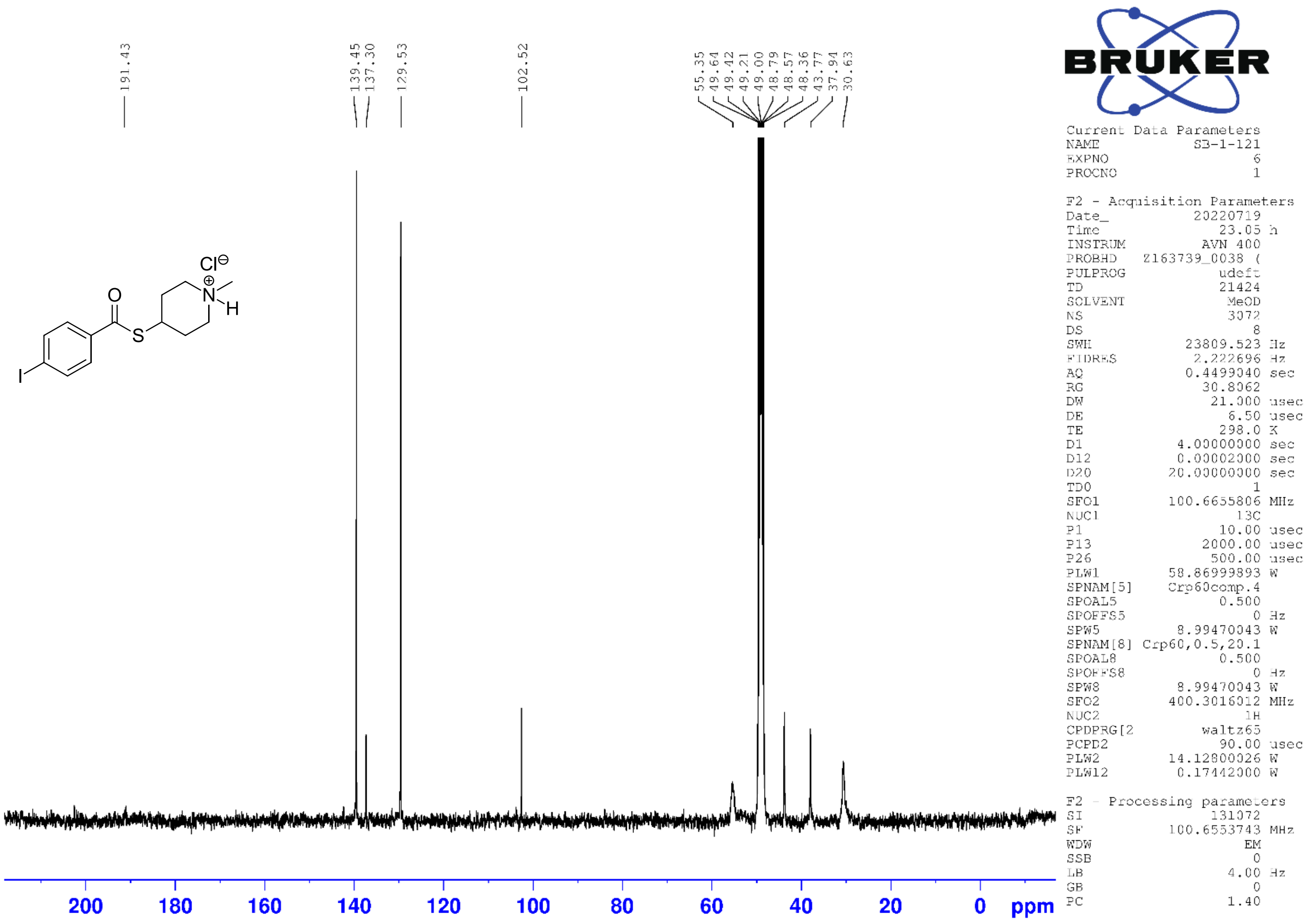

(11) (1-Methylpiperidin-4-yl) *p*-Iodobenzenecarbothioate Hydrochloride High Resolution Mass Spectrum (ESI, positive mode)

Generic Display Report

Analysis Info

Analysis Name	D:\Data\Xiao\July 21 2022000002.d	Acquisition Date	7/21/2022 10:12:51 AM
Method	Xiao all 1.m	Operator	x
Sample Name	TRV 6007 HCl	Instrument	compact
Comment			

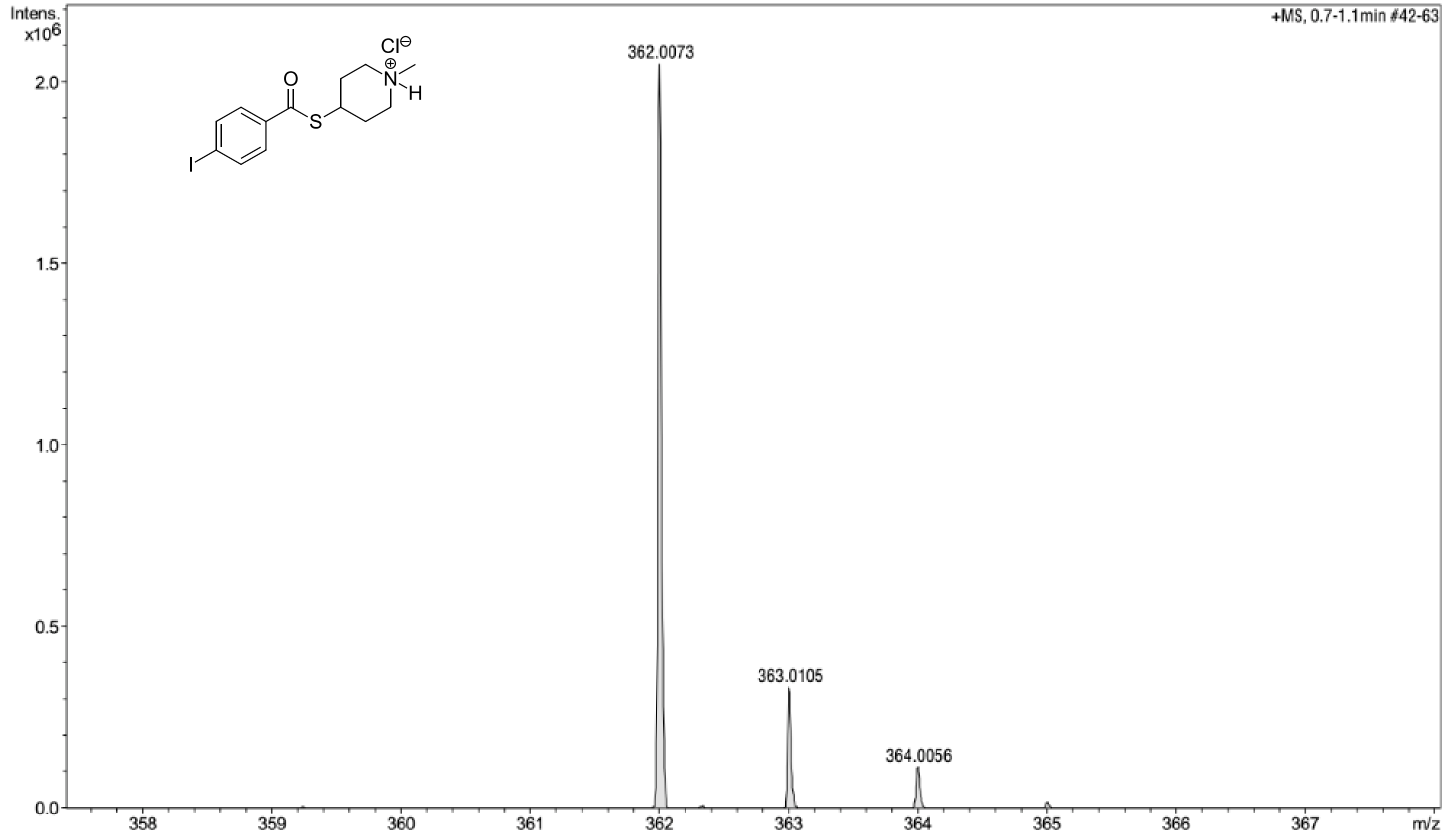

Bruker Compass DataAnalysis 4.3 printed: 7/21/2022 10:18:22 AM by: x Page 1 of 1

(11) (1-Methylpiperidin-4-yl) *p*-Iodobenzenecarbothioate Hydrochloride HPLC

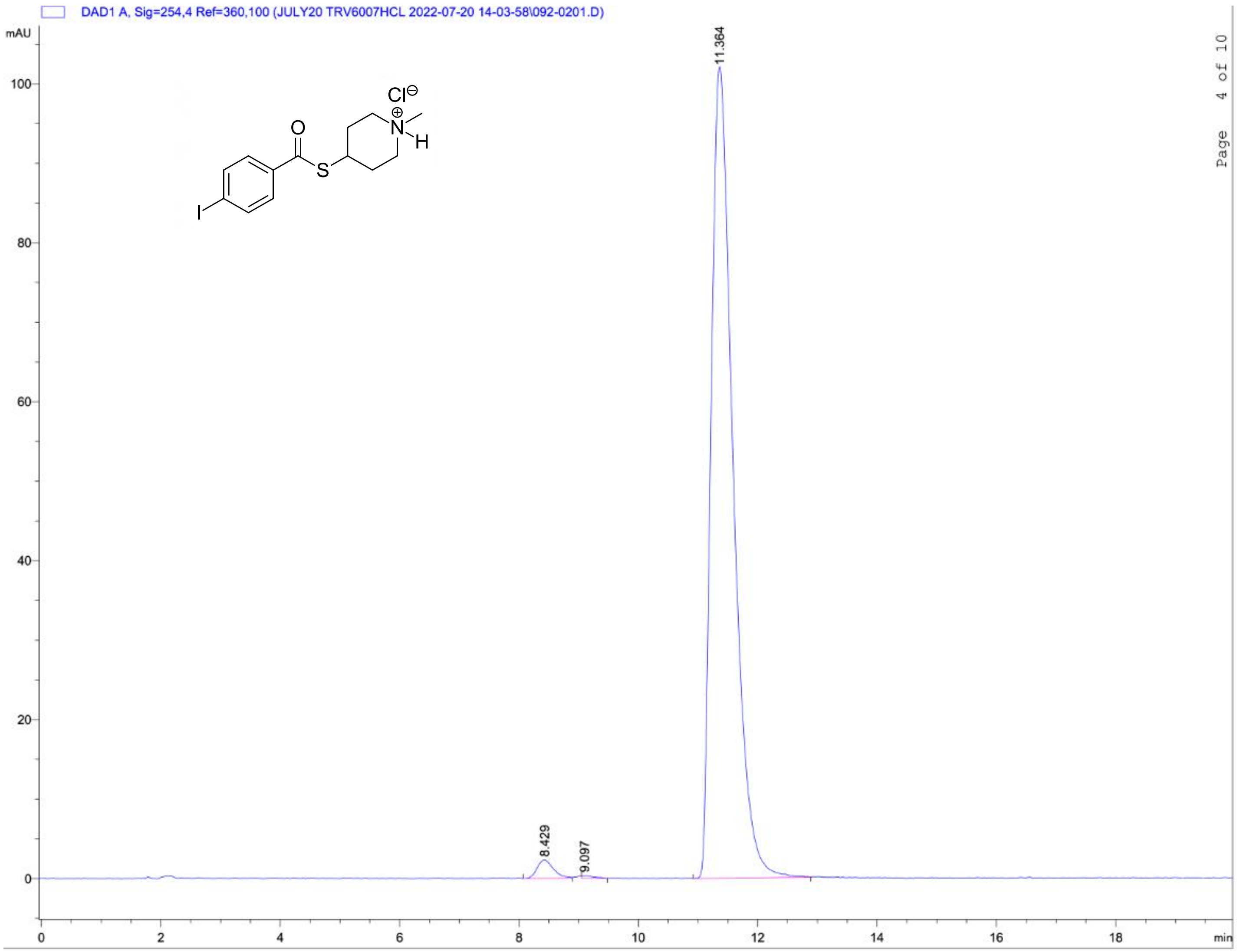

(12) 4-(*p*-Iodobenzoyloxy)-1-methyl-1-piperidinium Chloride

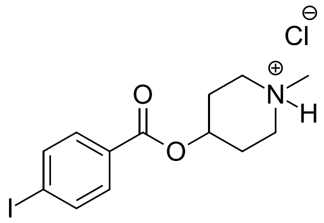

Synthesis: **1-Methylpiperidin-4-yl 4-iodobenzoate**: A sample from a previous study was obtained from our lab and the analytical data was similar to previously published data.[1]

4-(*p*-Iodobenzoyloxy)-1-methyl-1-piperidinium chloride (**11**): *N*-methylpiperidin-4-yl 4-iodobenzoate (0.4032 g, 1.2 mmol) was dissolved in anhydrous diethyl ether (39 mL) under an argon atmosphere. HCl in diethyl ether (1 N solution, 1.8 mL, 1.8 mmol) was added dropwise resulting in the immediate formation of a white precipitate. The reaction was stirred for an additional 60 mins. The precipitate was collected via suction filtration, washed with Et_2O (2 x 15 mL), to afford 4-(*p*-iodobenzoyloxy)-1-methyl-1-piperidinium chloride (0.200 mg, 44 % yield) as a white solid and a mixture of isomers (59:41).

Spectroscopic data: $MP_{(diethyl\ ether)}$: >250°C(Lit $MP_{(acetone/EtOH/H2O)}$:267-268.5^{o}C);[5] IR(ATR) 3014, 3003, 2944, 2612, 2512, 1721, 1586, 1272, 1118, 1008, 748; ^{1}H NMR (400 MHz, DMSO-*d6*) δ isomer A: 10.88 (br s, 1H), 7.94-7.92 (m, 2H), 7.73-7.71 (m, 2H), 5.11-5.02 (m, 1H), 3.33-3.30 (m, 2H), 3.17-3.07 (m, 2H), 2.73 (s, 3H), 2.21-2.15 (m, 2H), 2.06-2.00 (m, 2H), isomer B: 10.69 (br s, 1H), 7.94-7.92 (m, 2H), 7.86-784 (m, 2H), 5.23 (app br s, 1H), 3.47-3.44 (m, 2H), 3.25-3.17 (m, 2H), 2.79 (s, 3H), 2.21-2.15 (m, 2H), 2.06-2.00 (m, 2H); ^{13}C NMR (100.7 MHz, DMSO-*d6*) δ isomer A: 164.7 (0), 137.8 (1), 131.0 (1), 128.9 (0), 102.14 (0), 68.4 (1), 51.2 (2), 41.8 (3), 27.7 (2), isomer B: 164.6 (0), 137.6 (1), 131.3 (1), 129.2 (0), 102.07 (0), 64.8 (1), 48.7 (2), 42.4 (3), 26.7 (2); LRMS (ESI$^+$): 346.0 (M$^+$); HRMS (ESI$^+$): calculated for $C_{13}H_{17}INO_2^+$:

346.0298; found for $C_{13}H_{17}INO_2^+$: 346.0296; HPLC purity at 254nm (75% CH_3CN : 10% CH_3OH : 15% aqueous triethylamine [0.1% triethylamine in H_2O], retention time 7.883 mins): 99.3%.

(12) 4-(*p*-Iodobenzoyloxy)-1-methyl-1-piperidinium Chloride IR(ATR)

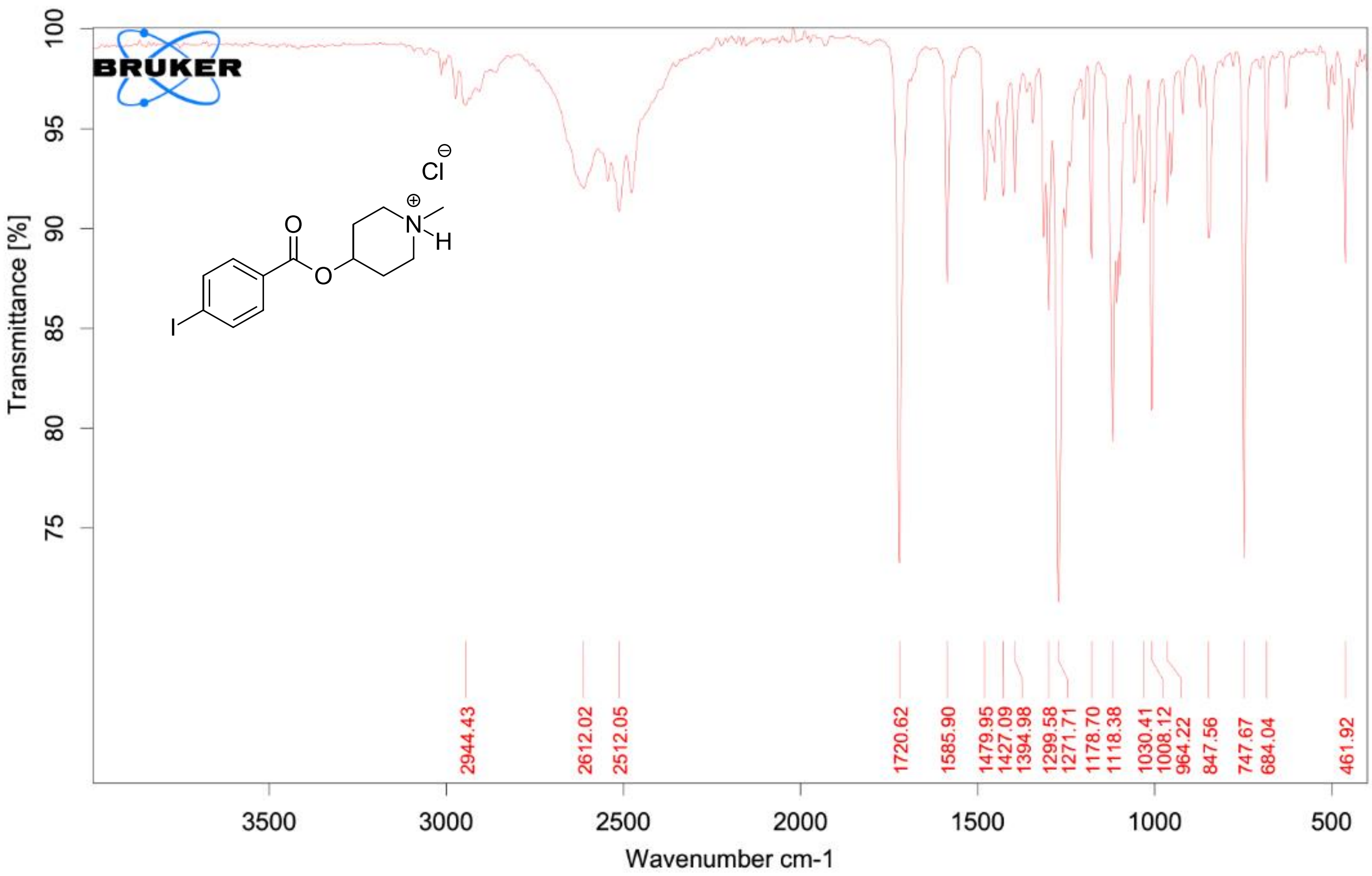

C:\Users\Administrator\Documents\Bruker\OPUS_7.5.18\DATA\MEAS\TRV6001 HCl.0	TRV6001 HCl	Instrument type and / or accessory	7/4/2022

(12) 4-(*p*-Iodobenzoyloxy)-1-methyl-1-piperidinium Chloride ^{1}H NMR 400 MHz (DMSO-*d6*)

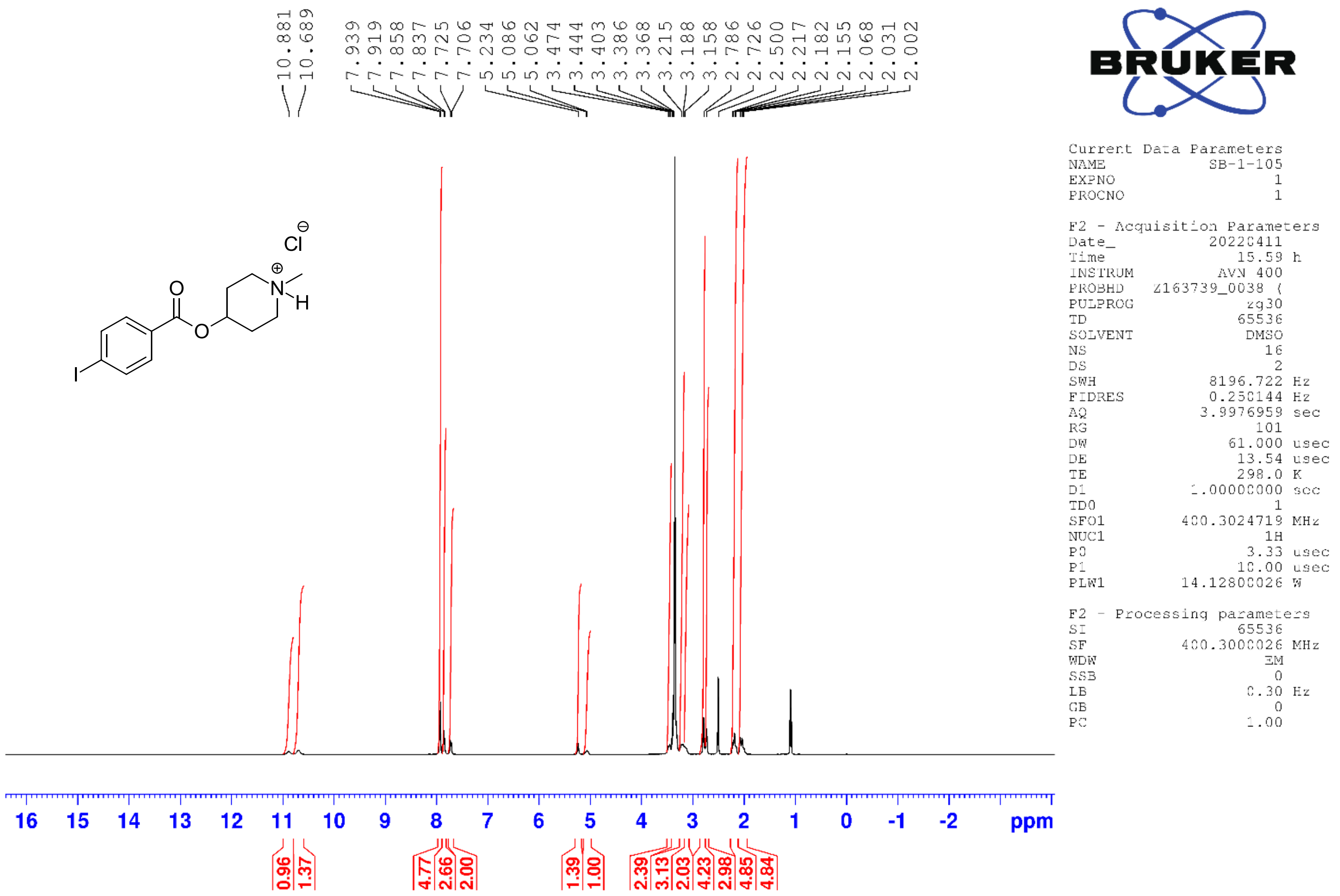

(12) 4-(*p*-Iodobenzoyloxy)-1-methyl-1-piperidinium Chloride ^{13}C NMR 100 MHz (DMSO-*d6*)

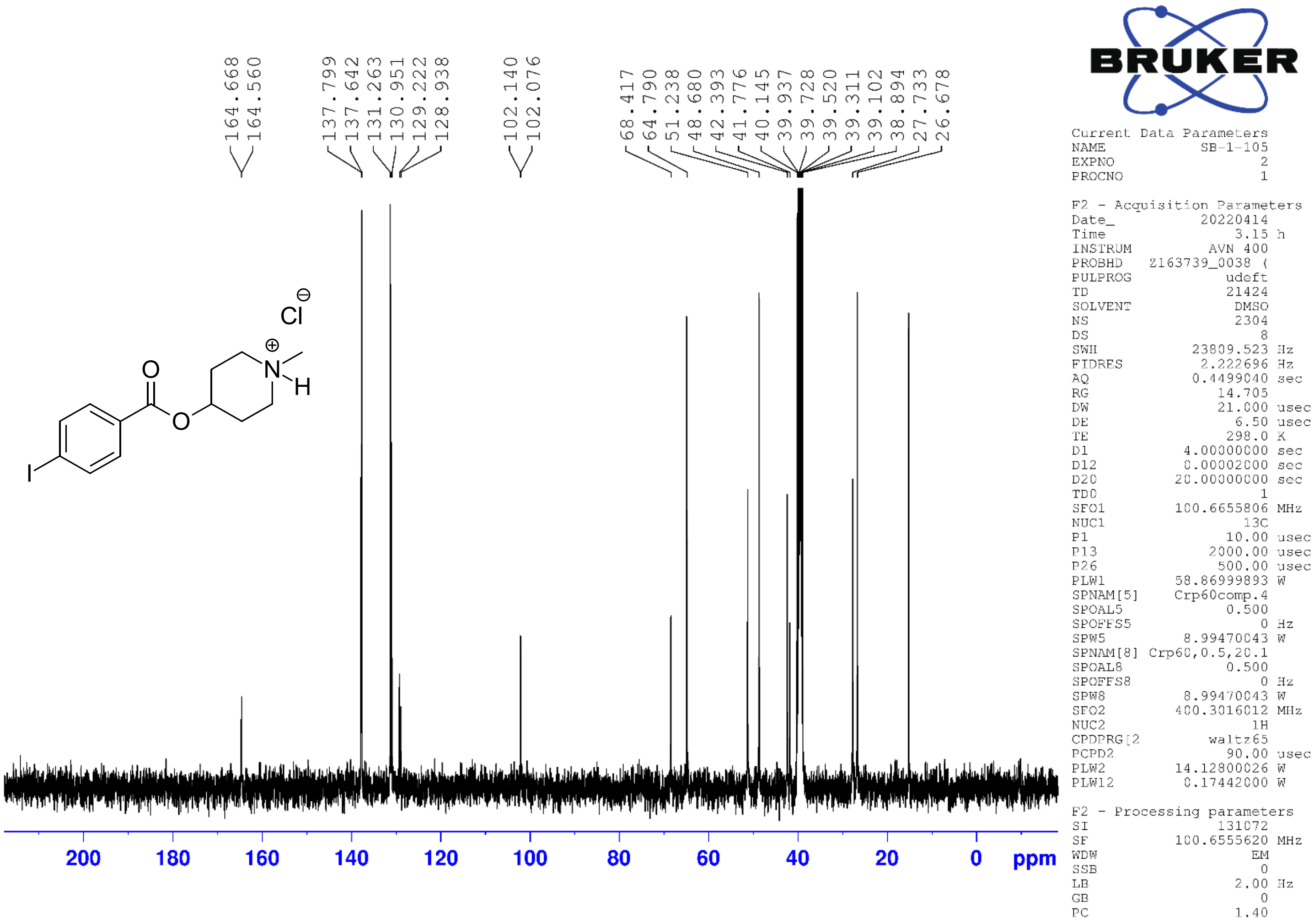

(12) 4-(*p*-Iodobenzoyloxy)-1-methyl-1-piperidinium Chloride High Resolution Mass Spectrum (ESI, positive mode)

Mass Spectrum SmartFormula Report

Analysis Info

Analysis Name	D:\Data\Xiao\July 06 2022000008.d	Acquisition Date	7/6/2022 2:44:43 PM	
Method	Xiao all 1.m	Operator	x	
Sample Name	TRV 6001 HCl	Instrument	compact	8255754.20059
Comment				

Acquisition Parameter

Source Type	ESI	Ion Polarity	Positive	Set Nebulizer	0.5 Bar
Focus	Not active	Set Capillary	3500 V	Set Dry Heater	181 °C
Scan Begin	50 m/z	Set End Plate Offset	-500 V	Set Dry Gas	4.0 l/min
Scan End	1500 m/z	Set Charging Voltage	2000 V	Set Divert Valve	Source
		Set Corona	0 nA	Set APCI Heater	0 °C

Meas. m/z	Ion Formula	m/z	err [ppm]
346.0296	C13H17INO2	346.0299	0.8

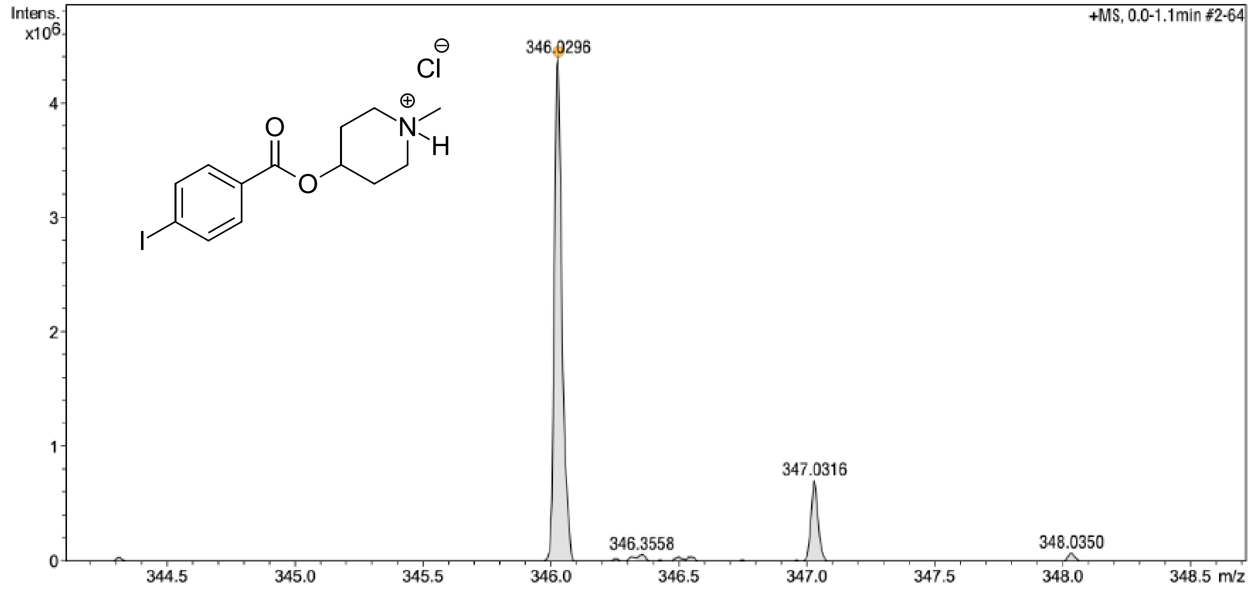

July 06 2022000008.d

Bruker Compass DataAnalysis 4.3 printed: 7/6/2022 2:48:15 PM by: x Page 1 of 1

(12) 4-(*p*-Iodobenzoyloxy)-1-methyl-1-piperidinium Chloride HPLC

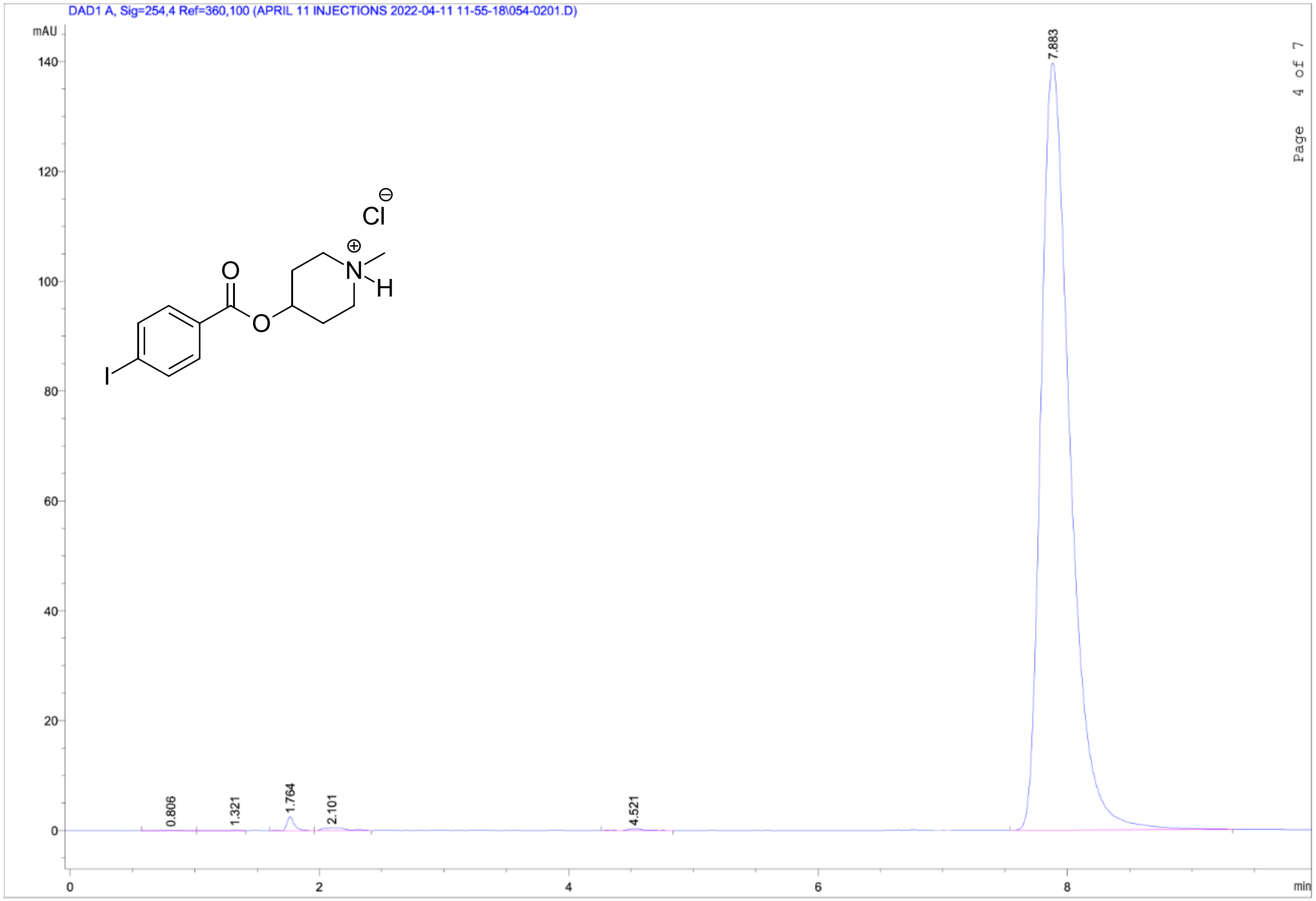

(13) *S*-(1-Methylpiperidin-4-yl) *p*-Fluorobenzenecarbothioate Hydrochloride

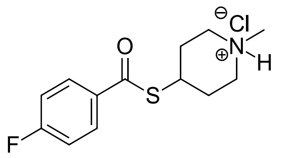

Synthesis: ***S*-(1-Methylpiperidin-4-yl) *p*-fluorobenzenecarbothioate**: To a solution of *N*-methyl-4-piperidinethiol (0.4987 g, 3.8 mmol) and triethylamine (0.80 mL, 5.7 mmol) in anhydrous dichloromethane (20 mL) was added 4-florobenzoyl chloride (0.55 mL, 4.6 mmol) dropwise via syringe under an argon atmosphere. The mixture was heated to reflux for 3 hrs until TLC indicated complete consumption of starting material. Upon cooling to room temperature, the mixture was diluted with DCM (20 mL). The organic layer was washed with saturated $NaHCO_{3(aq)}$ (20 mL), water (20 mL) and dried over Na_2SO_4. The mixture was gravity filtered and concentrated to afford the crude material. Purification *via* flash chromatography (5 % MeOH / DCM) afforded *S*-(1-methylpiperidin-4-yl) *p*-fluorobenzenecarbothioate **(**0.448 g, 47 % yield) as an off-white solid. The analytical data was similar to previously published data.[1]

***S*-(1-Methylpiperidin-4-yl) *p*-fluorobenzenecarbothioate hydrochloride (14):** To a flask charged with *S*-(1-methylpiperidin-4-yl) *p*-fluorobenzenecarbothioate (0.206 g, 0.815 mmol) anhydrous diethyl ether was added until complete dissolution (16 mL) under an argon atmosphere. HCl in diethyl ether 1 N - 1.22 mL, 1.22 mmol) was added dropwise and stirred for 1hr. The solvent was removed *in vacuo* resulting in a white solid. The product was triturated three times with diethyl ether (20 mL), solvent was removed with a pipet and compound was dried to produce *S*-(1-methylpiperidin-4-yl) *p*-fluorobenzenecarbothioate hydrochloride (0.230 g, 97%).

Spectroscopic data: $MP_{(diethyl\ ether)}$: 209-211°C, IR(ATR): 3416, 3317, 3105, 3082, 2994, 2946, 2696, 2509, 2432, 1659, 1594, 1504, 1216, 919, 638 cm^{-1}; ^{1}H NMR (400 MHz, DMSO-*d6*) δ isomer A: 10.63 (br s, 1H), 8.01-7.97 (m, 2H), 7.42-7.38 (m, 2H), 4.09 (br s, 1H), 3.40-3.38 (m, 2H), 3.09-3.07 (m, 2H), 2.77 (s, 3H), 2.38-2.33 (m, 2H), 2.04-1.94 (m, 2H); isomer B: 10.63 (br s, 1H), 8.01-7.97 (m, 2H), 7.42-7.38 (m, 2H), 3.76-3.69 (m, 1H), 3.46-3.43 (m, 2H), 3.17-3.10 (m, 2H), 2.73 (s, 3H), 2.17-2.13 (m, 2H), 2.04-1.94 (m, 2H); ^{13}C NMR (100.7 MHz, DMSO-*d6*) δ isomer A: 188.6, 164.3 (d, $^{1}J_{C,F}$ = 252.9 Hz), 132.9 (d, $^{4}J_{C,F}$ = 2.7 Hz), 129.9 (d, $^{3}J_{C,F}$ = 9.7 Hz), 116.4 (d, $^{2}J_{C,F}$ = 22.4 Hz), 50.3, 41.7, 36.6, 27.3, isomer B: 188.8, 166.8 (d, $^{1}J_{C,F}$ = 252.9 Hz), 132.9 (d, $^{4}J_{C,F}$ = 2.7 Hz), 129.9 (d, $^{3}J_{C,F}$ = 9.7 Hz), 116.4 (d, $^{4}J_{C,F}$ = 22.4 Hz), 53.1, 42.5, 37.1, 29.0; LRMS (ESI^{+}): 254.1 (M^{+}); HRMS (ESI^{+}): calculated for $C_{13}H_{17}FNOS^{+}$: 254.1010 amu; found for $C_{13}H_{17}FNOS^{+}$: 254.1010 amu; HPLC purity at 254nm (75% CH_3CN : 10% CH_3OH : 15% aqueous triethylamine [0.1% triethylamine in H_2O], retention time 5.912 mins): 98.9 %.

(13) *S*-(1-Methylpiperidin-4-yl) *p*-Fluorobenzenecarbothioate Hydrochloride IR(ATR)

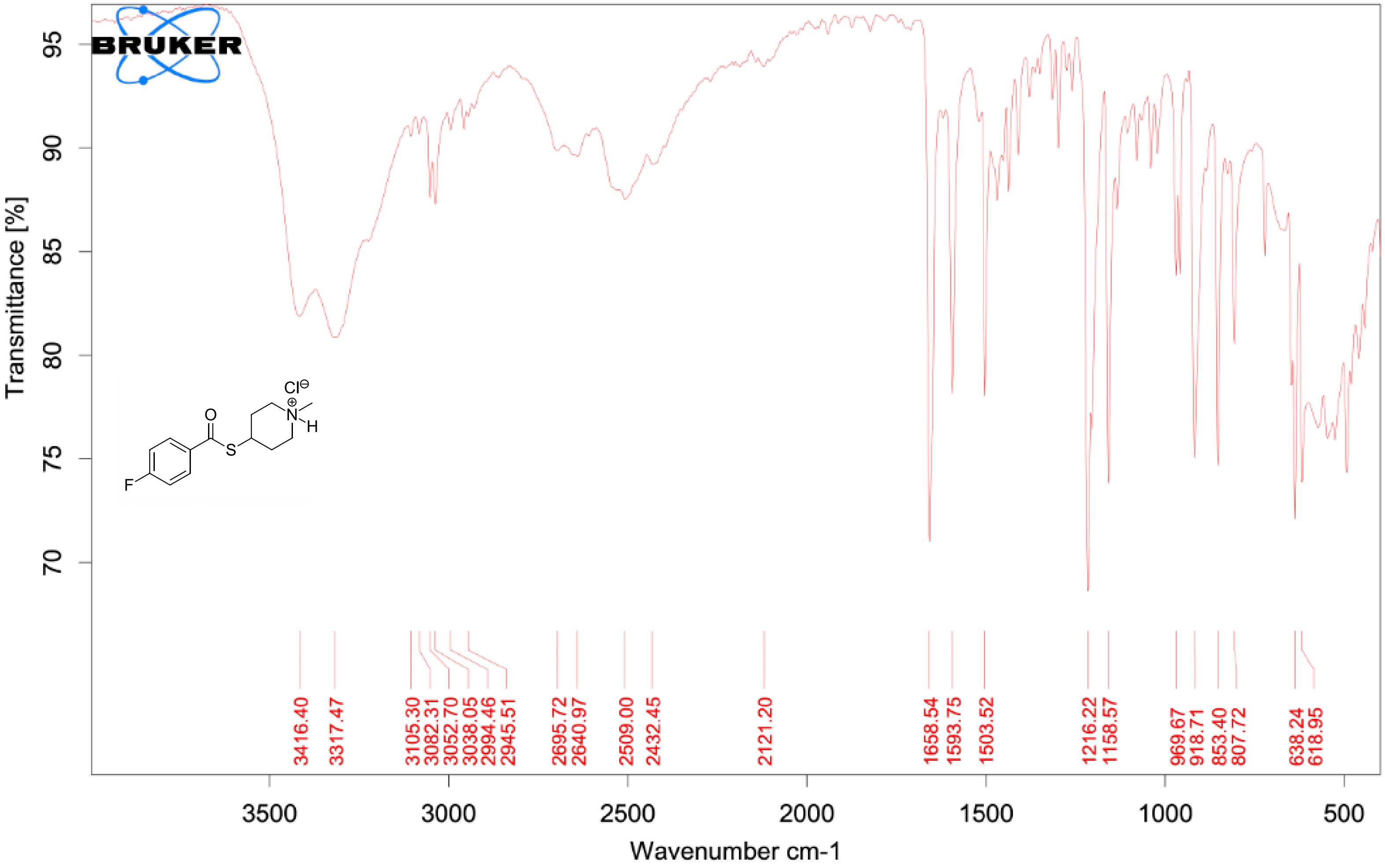

(13) *S*-(1-Methylpiperidin-4-yl) *p*-Fluorobenzenecarbothioate Hydrochloride ^{1}H NMR 400 MHz (DMSO-*d6*)

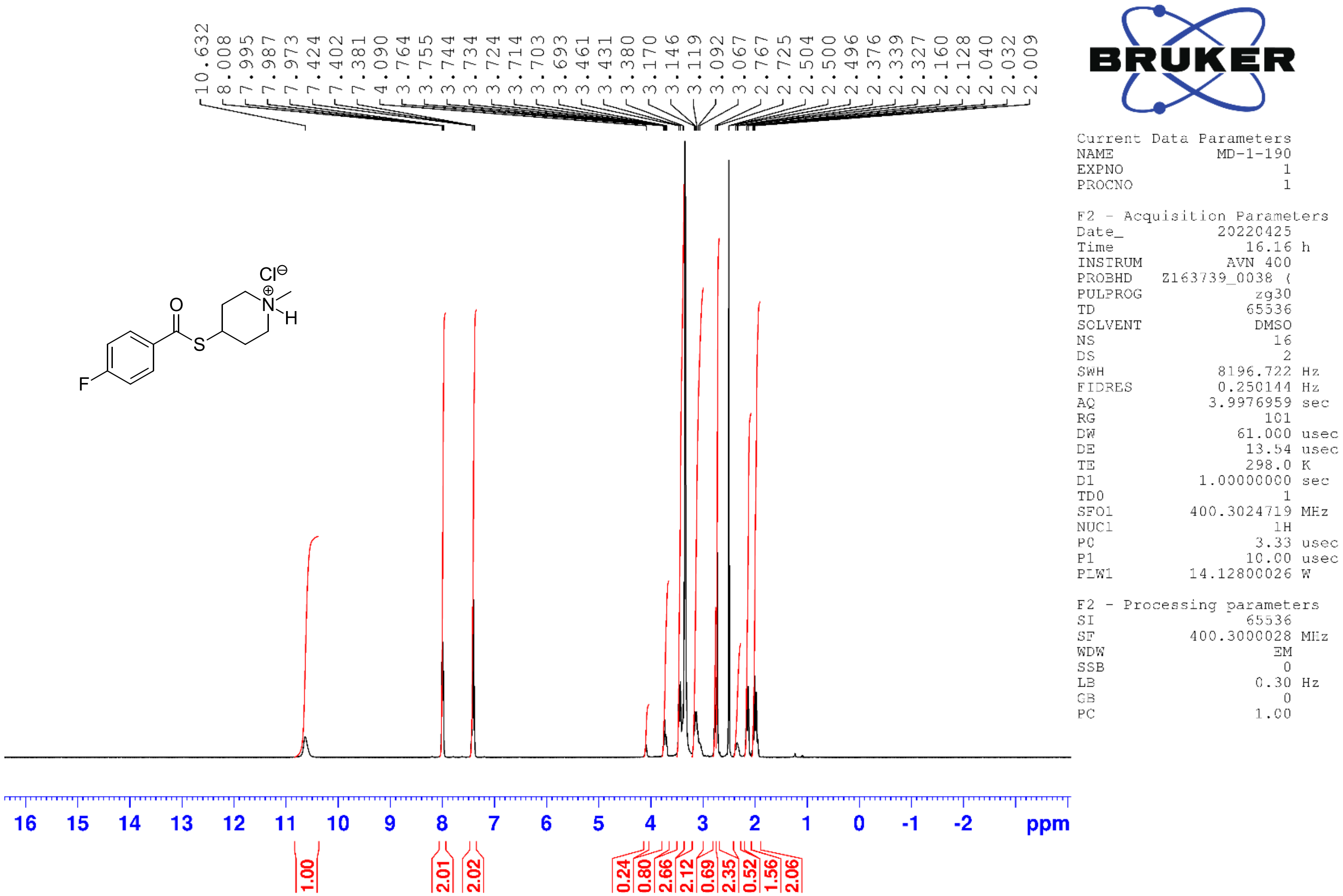

(13) *S*-(1-Methylpiperidin-4-yl) *p*-Fluorobenzenecarbothioate Hydrochloride ^{13}C NMR 100 MHz (DMSO-*d6*)

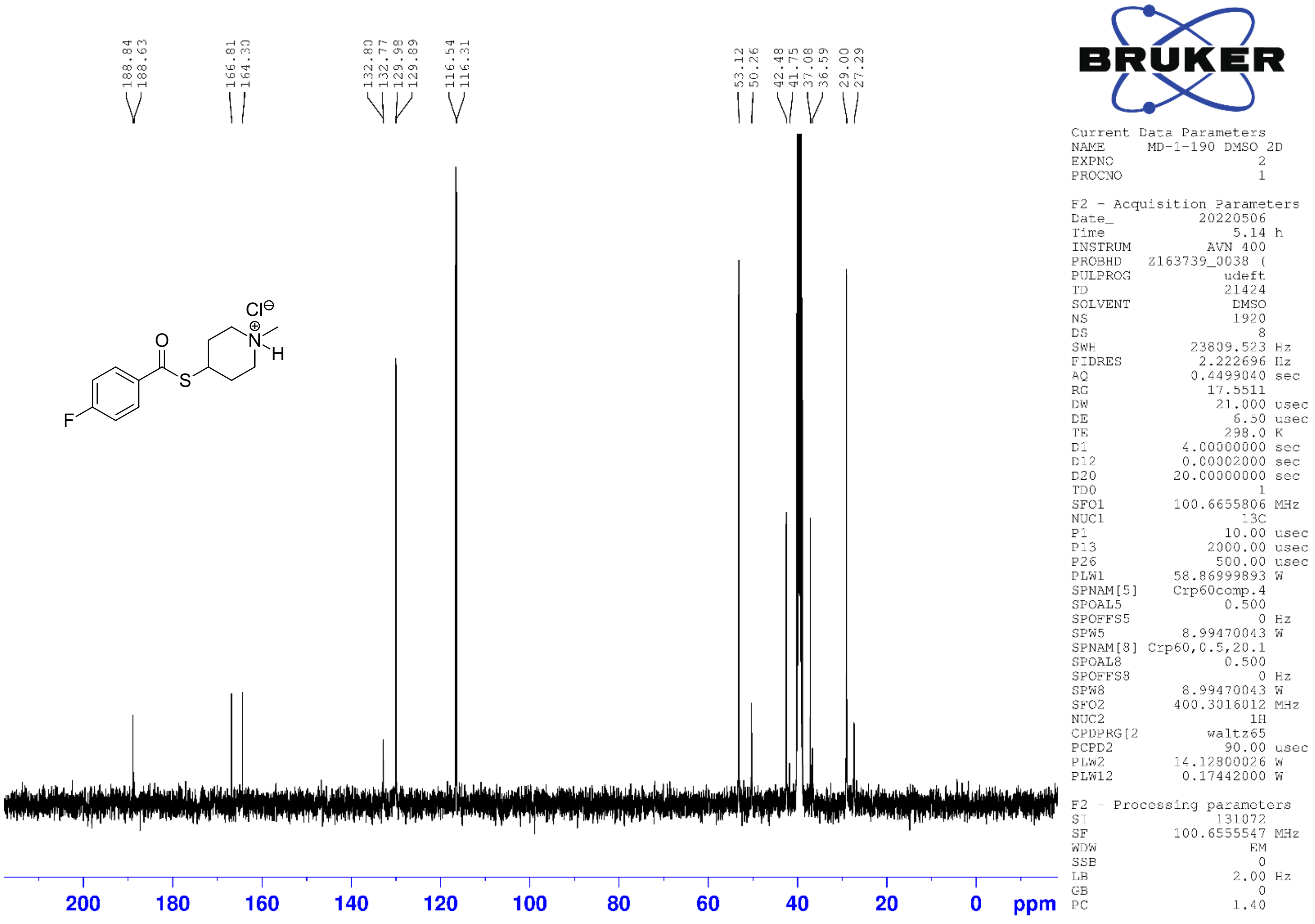

(13) *S*-(1-Methylpiperidin-4-yl) *p*-Fluorobenzenecarbothioate Hydrochloride High Resolution Mass Spectrum (ESI, positive mode)

Mass Spectrum SmartFormula Report

Analysis Info

Analysis Name	D:\Data\Xiao\July 07 2022000015.d	Acquisition Date	7/7/2022 10:12:10 AM	
Method	Xiao all 1.m	Operator	x	
Sample Name	TRV 6504 HCl	Instrument	compact	8255754.20059
Comment				

Acquisition Parameter

Source Type	ESI	Ion Polarity	Positive	Set Nebulizer	0.5 Bar
Focus	Not active	Set Capillary	3500 V	Set Dry Heater	180 °C
Scan Begin	50 m/z	Set End Plate Offset	-500 V	Set Dry Gas	4.0 l/min
Scan End	1500 m/z	Set Charging Voltage	2000 V	Set Divert Valve	Source
		Set Corona	0 nA	Set APCI Heater	0 °C

Meas. m/z	Ion Formula	m/z	err [ppm]
254.1010	C13H17FNOS	254.1009	-0.3

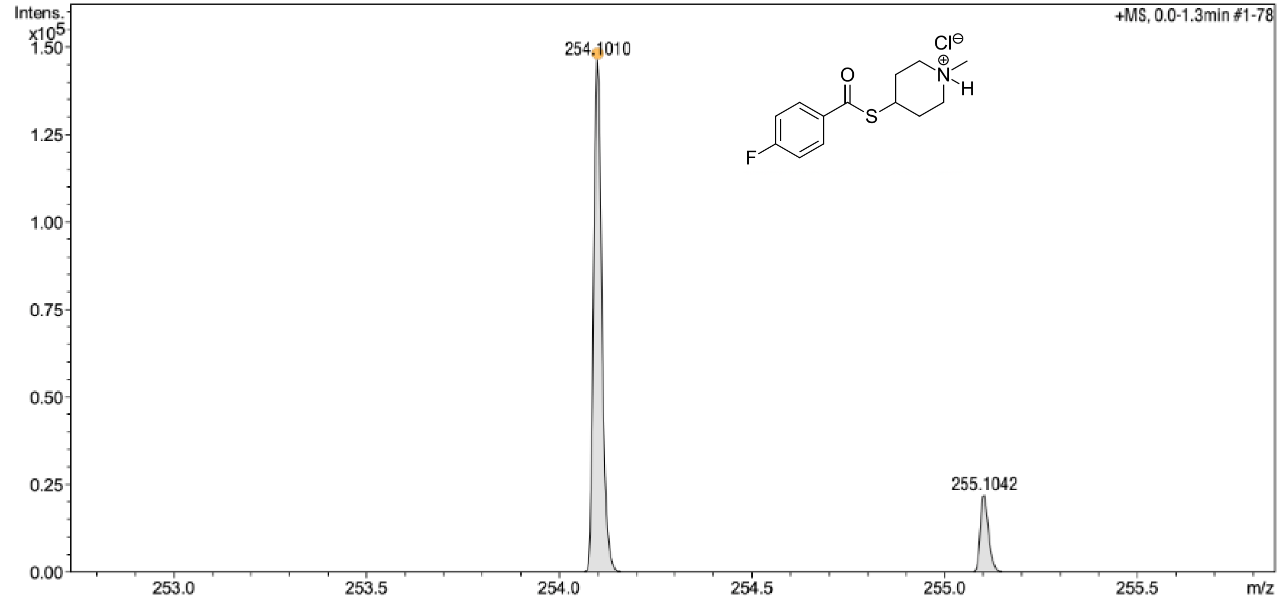

July 07 2022000015.d

Bruker Compass DataAnalysis 4.3 printed: 7/7/2022 10:15:30 AM by: x Page 1 of 1

(13) *S*-(1-Methylpiperidin-4-yl) *p*-Fluorobenzenecarbothioate Hydrochloride HPLC

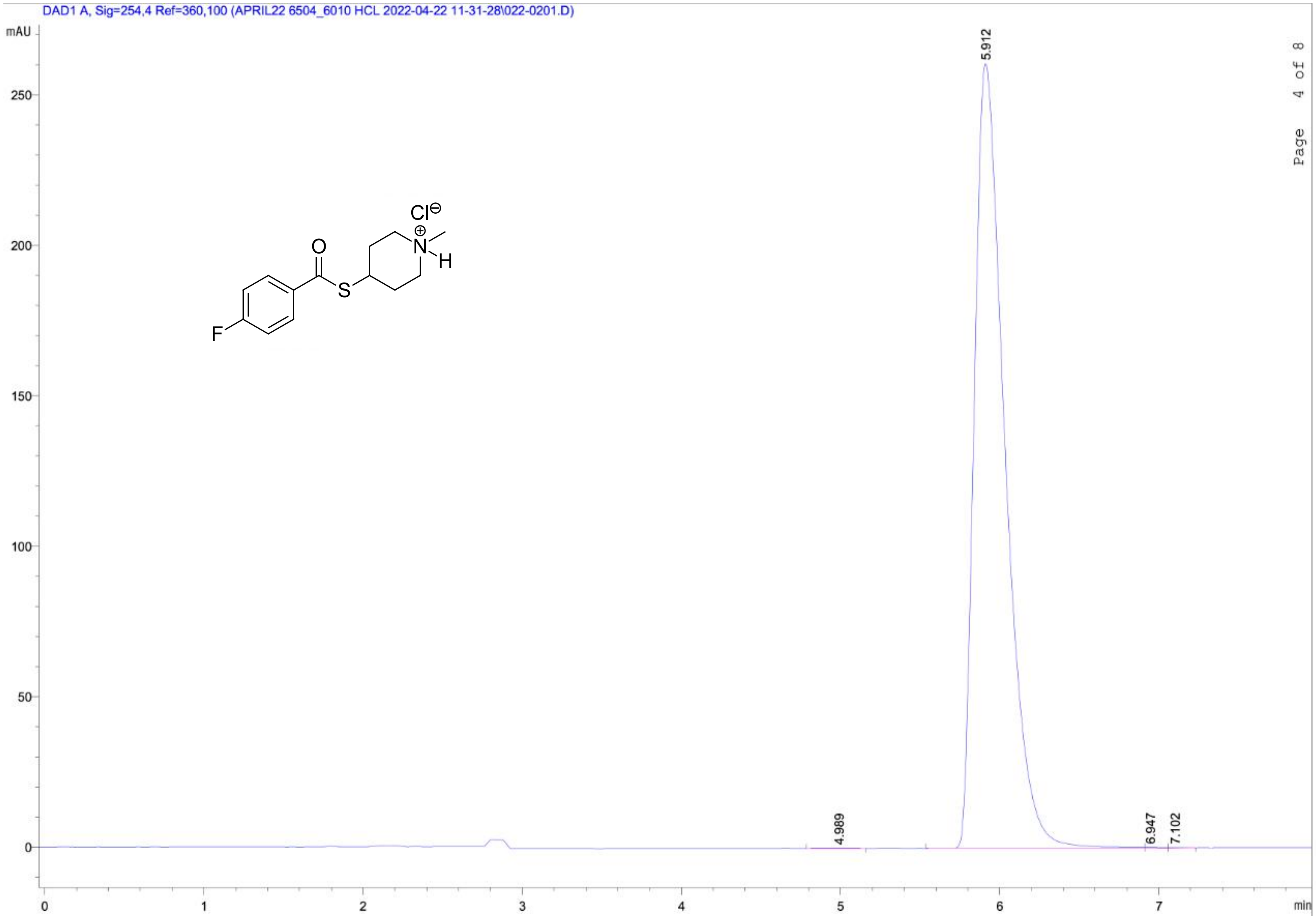

(14) 4-(*p*-Fluorobenzoyloxy)-1-methyl-1-piperidinium Chloride

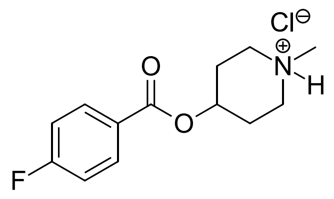

Synthesis: ***N*-Methylpiperidin-4-yl 4-fluorobenzoate**: 4-fluorobenzoyl chloride (0.76 mL, 6.3 mmol), triethylamine (2.2 mL, 15.8 mmol) and DMAP (0.138 g, 1.13 mmol) were dissolved in anhydrous dichloromethane (32 mL) under an argon atmosphere. *N*-Methyl-4-piperidinol (0.979 g, 6.3 mmol) was added to the stirred solution in one portion. The reaction mixture was stirred for 3 hrs at room temperature. After this time, the reaction was quenched with brine (30 mL) and extracted with dichloromethane (3 × 15 mL). The combined organic layers were dried over Na_2SO_3, gravity filtered, and the filtrate was concentrated *in vacuo* to produce a clear colourless oil. The crude mixture was purified by silica gel column chromatography (4% MeOH/DCM) to produce a clear colourless oil, *N*-Methylpiperidin-4-yl 4-fluorobenzoate (1.47 g, 98%). The analytical data was similar to previously published data.[1]

4-(*p*-Fluorobenzoyloxy)-1-methyl-1-piperidinium chloride (13): To a flask charged with *N*-methylpiperidin-4-yl 4-fluorobenzoate (0.748 g, 3.15 mmol) anhydrous diethyl ether was added until complete dissolution (63 mL) under an argon atmosphere. HCl in diethyl ether 1N (4.73 mL, 4.73 mmol) was added dropwise and stirred for 1 hr at room temperature. The solvent was removed *in vacuo* resulting in a white solid. The compound was triturated with diethyl ether (20 mL) and solvent was removed with a pipet. This process was repeated two more times. The material was dried *in vacuo* to produce 4-(*p*-fluorobenzoyloxy)-1-methyl-1-piperidinium chloride (**13**, 0.773 g, 90%, 2:3 mixture of isomers) as a white solid.

Spectroscopic data: $MP_{(diethyl\ ether)}$: 219-221°C (Lit $MP_{(acetone/ethanol)}$: 227-228°C); IR (ATR) 2942, 2498, 2431, 1729, 1274, 1257, 766, 605 cm^{-1}; ^{1}H NMR (400 MHz, DMSO-*d6*) δ isomer A: 10.78 (br s, 1H), 8.06-8.02 (m, 2H), 7.40-7.34 (m, 2H), 5.10-5.03 (m, 1H), 3.40-3.37 (m, 2H), 3.18-3.10 (m, 2H), 2.73 (d, J = 4.0 Hz, 3H), 2.22-2.14 (m, 2H), 2.07-1.96 (m, 2H); isomer B: 10.59 (br s, 1H), 8.19-8.16 (m, 2H), 7.40-7.34 (m, 2H), 5.26-5.23 (m, 1H), 3.48-3.45 (m, 2H), 3.26-3.18 (m, 2H), 2.80 (d, J = 4.5 Hz, 3H), 2.22-2.14 (m, 2H), 2.07-1.96 (m, 2H); ^{13}C NMR (100.7 MHz, CD_3OD) δ isomer A: 165.3 (d, $^{1}J_{C,F}$ = 246.4 Hz), 163.9, 132.2 (d, $^{3}J_{C,F}$ = 9.7 Hz), 126.0, 115.9 (d, $^{2}J_{C,F}$ = 22.5 Hz), 68.3, 51.2, 41.8, 27.7; isomer B: 165.3 (d, $^{1}J_{C,F}$ = 246.4 Hz), 164.0, 132.5 (d, $^{3}J_{C,F}$ = 9.6 Hz), 126.3, 115.8 (d, $^{2}J_{C,F}$ = 21.9 Hz), 64.6, 48.7, 42.4, 26.7; LRMS (ESI^{+}): 238.1 (M^{+}); HRMS (ESI^{+}): calculated for $C_{13}H_{17}FNO_2^{+}$: 238.1238 amu; found for $C_{13}H_{17}FNO_2^{+}$: 238.1244 amu; HPLC purity at 254nm (75% CH_3CN : 10% CH_3OH : 15% aqueous triethylamine [0.1% triethylamine in H_2O], retention time 4.854 mins): 100 %.[6]

(14) 4-(*p*-Fluorobenzoyloxy)-1-methyl-1-piperidinium Chloride IR(ATR)

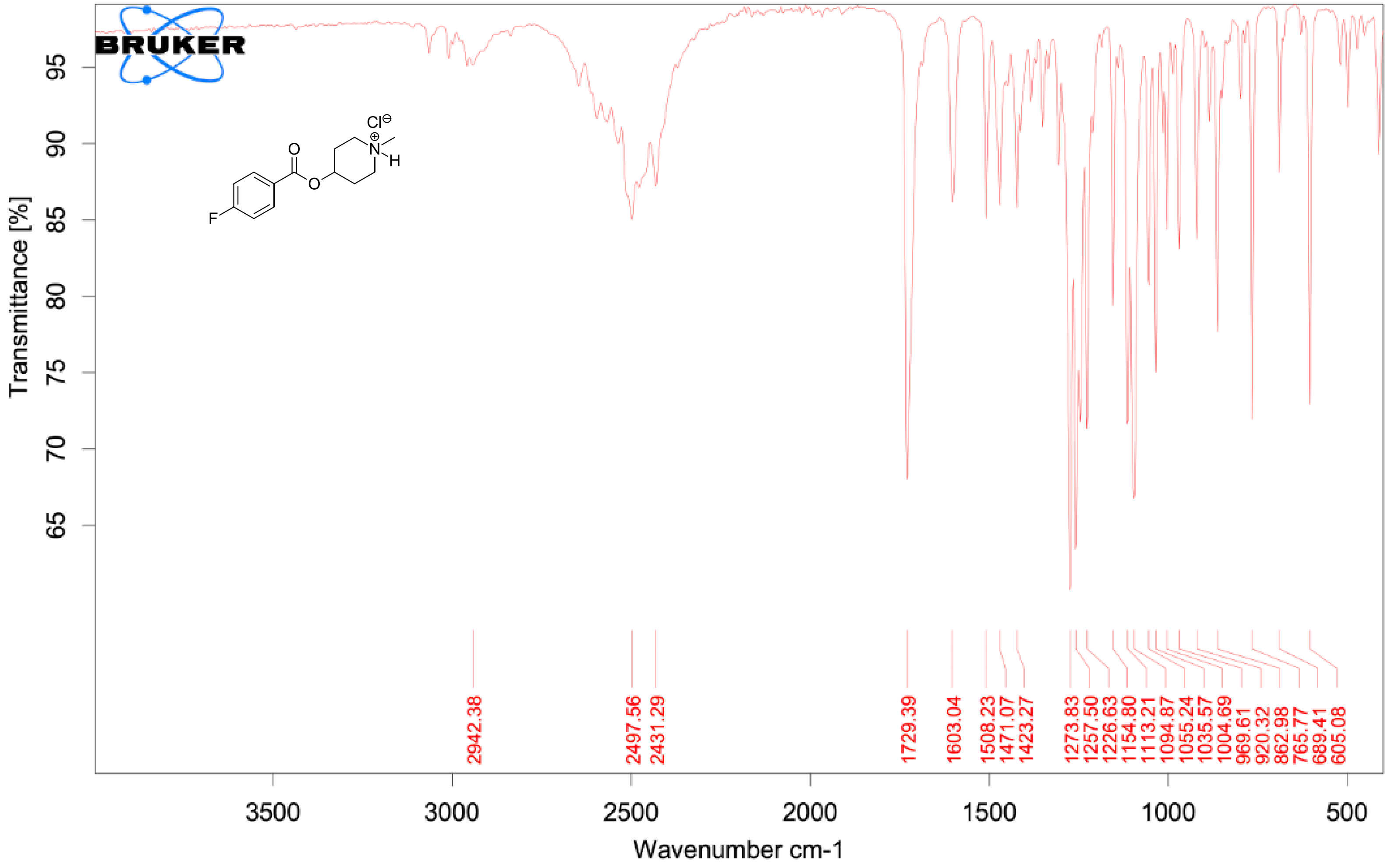

(14) 4-(*p*-Fluorobenzoyloxy)-1-methyl-1-piperidinium Chloride ^{1}H NMR 400 MHz (DMSO-*d6*)

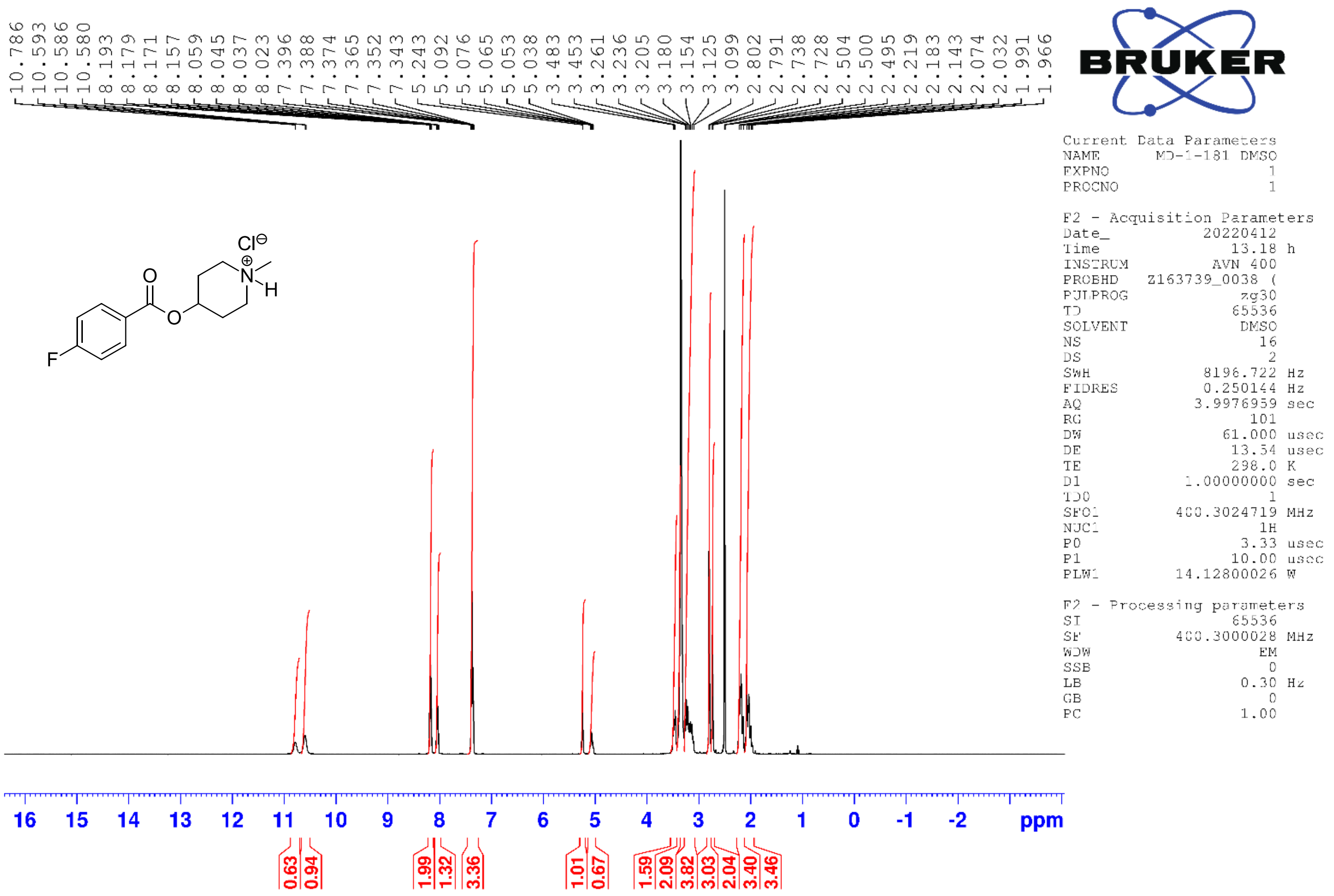

(14) 4-(*p*-Fluorobenzoyloxy)-1-methyl-1-piperidinium Chloride ^{13}C NMR 100 MHz (DMSO-*d6*)

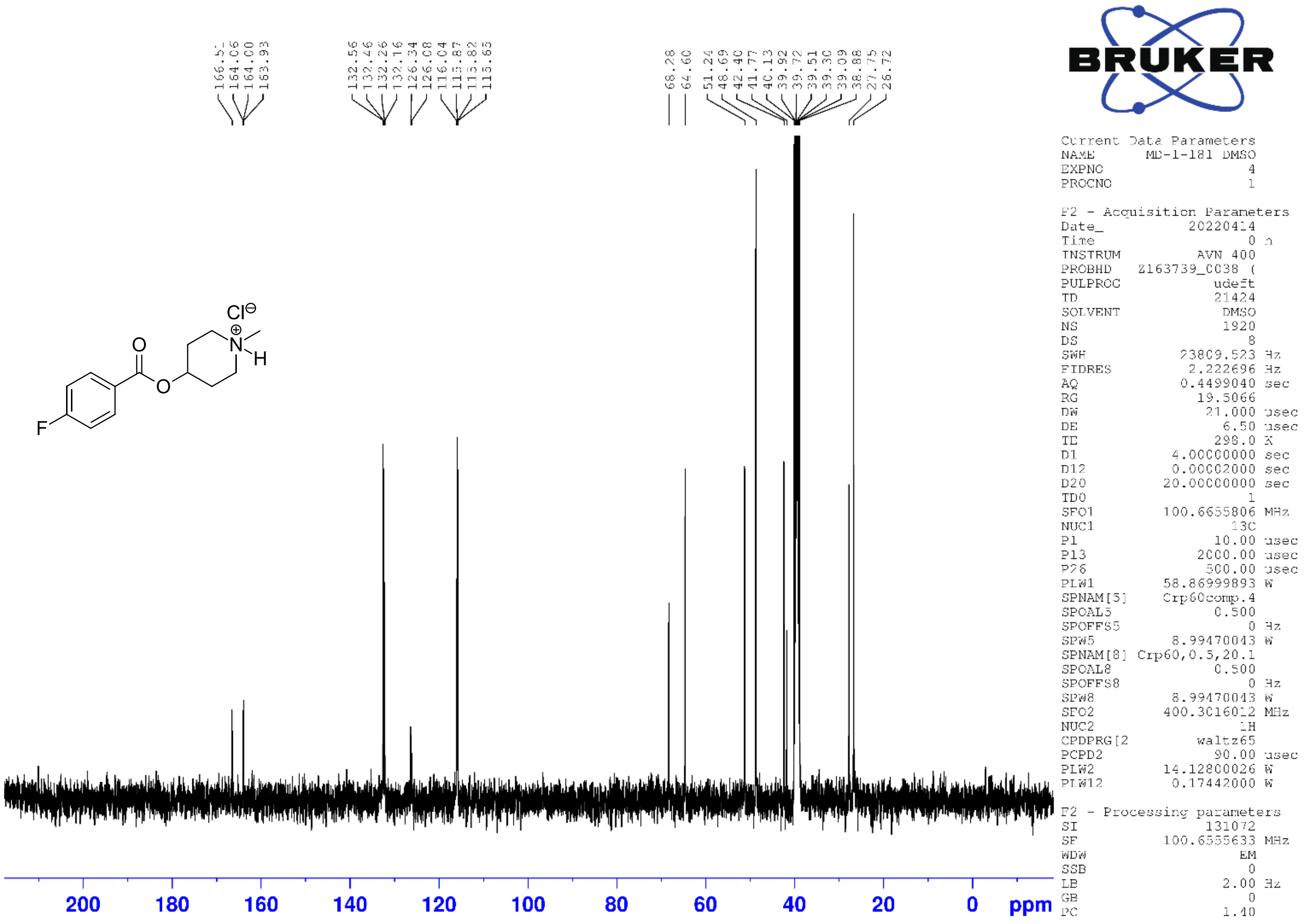

(14) 4-(*p*-Fluorobenzoyloxy)-1-methyl-1-piperidinium Chloride High Resolution Mass Spectrum (ESI, positive mode)

Mass Spectrum SmartFormula Report

Analysis Info

Analysis Name	D:\Data\Xiao\July 07 2022000018.d	Acquisition Date	7/7/2022 10:21:47 AM
Method	Xiao all 1.m	Operator	x
Sample Name	TRV 6501 HCl	Instrument	compact 8255754.20059
Comment			

Acquisition Parameter

Source Type	ESI	Ion Polarity	Positive	Set Nebulizer	0.5 Bar
Focus	Not active	Set Capillary	3500 V	Set Dry Heater	180 °C
Scan Begin	50 m/z	Set End Plate Offset	-500 V	Set Dry Gas	4.0 l/min
Scan End	1500 m/z	Set Charging Voltage	2000 V	Set Divert Valve	Source
		Set Corona	0 nA	Set APCI Heater	0 °C

Meas. m/z	Ion Formula	m/z	err [ppm]
238.1244	C13H17FNO2	238.1238	-2.4

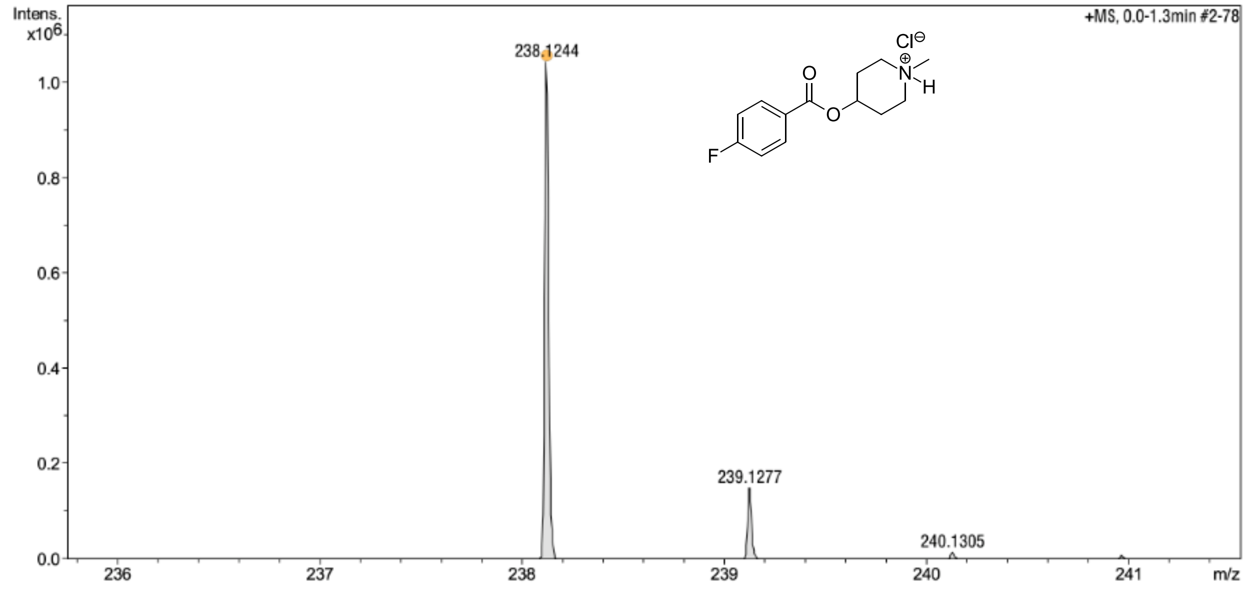

July 07 2022000018.d

Bruker Compass DataAnalysis 4.3 printed: 7/7/2022 10:24:59 AM by: x Page 1 of 1

(14) 4-(*p*-Fluorobenzoyloxy)-1-methyl-1-piperidinium Chloride HPLC

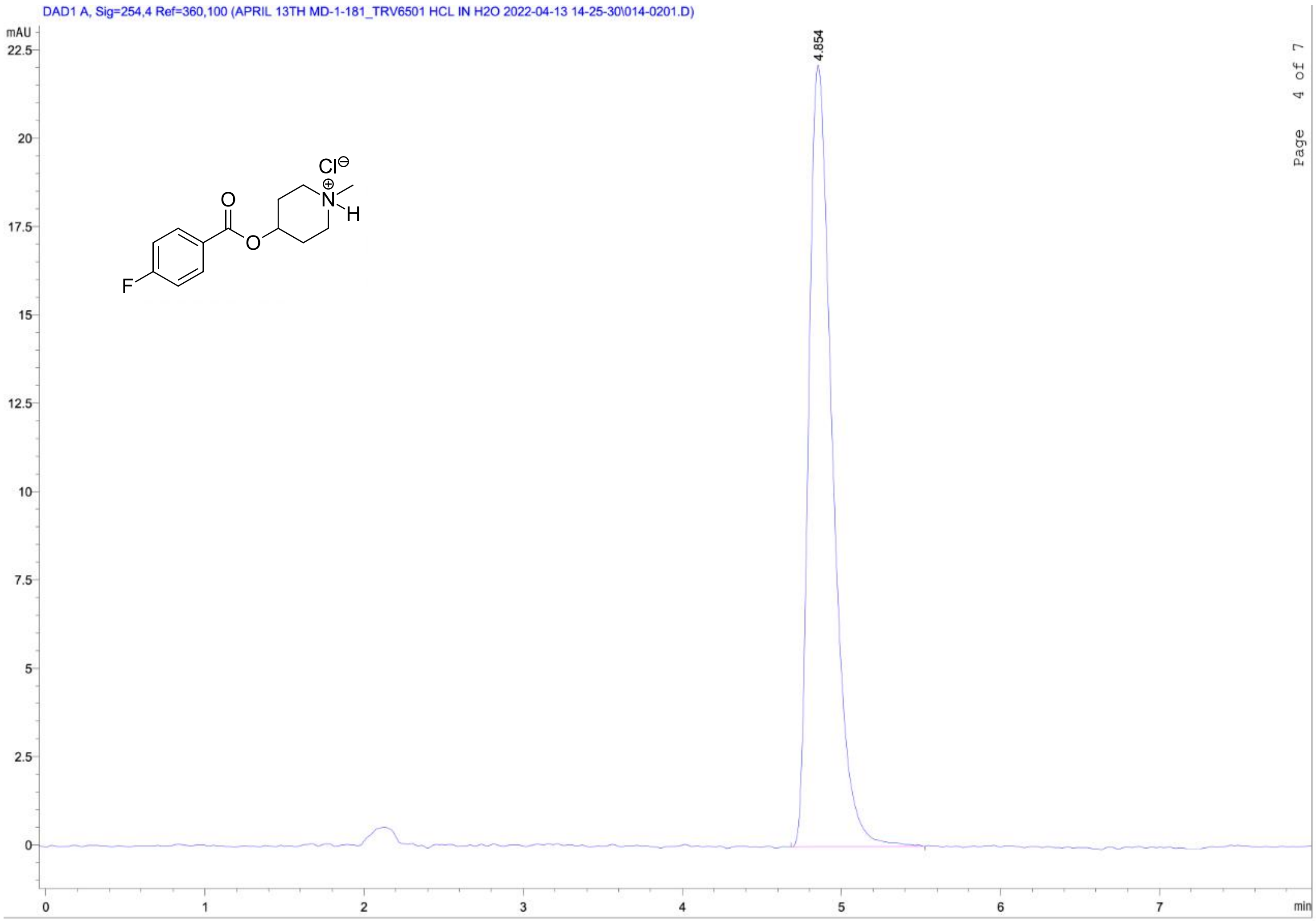

Alkyl N-Methylpyrrolidinyl Derivatives

(15) (*3S*)-*S*-(1-Methylpyrrolidin-3-yl) Ethanethioate

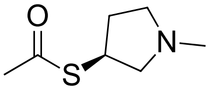

Synthesis: **(*3R*)-3-(Mesyloxy)-1-methylpyrrolidine**: (3*R*)-(-)-1-Methyl-3-pyrrolidinol (5.00 mL, 45.5 mmol) and triethylamine (9.52 mL, 68.3 mmol) were dissolved in anhydrous dichloromethane (152 mL) and cooled to 0°C with an ice bath. Methanesulfonyl chloride (5.22 mL, 52.4 mmol) was added dropwise at 0°C. The ice bath was removed after 5 mins, and the reaction was stirred overnight. The reaction was quenched with brine (50 mL), extracted with dichloromethane (3 × 15 mL). The combined organic layers were dried over Na_2SO_3, gravity filtered, and the filtrate was concentrated *in vacuo* to produce a viscous yellow liquid (4.14 g, 51%). Product confirmed by ^{1}H NMR and immediately carried through to next step.

Spectroscopic data: IR(ATR): 3026, 2942, 2784, 1346, 1166, 521 cm^{-1}; ^{1}H NMR (400 MHz, $CDCl_3$) d 5.23-5.19 (m, 1H), 3.01 (s, 3H), 2.90 (dd, J = 11.3, 2.2 Hz, 1H), 2.87-2.83 (m, 1H), 2.72 (dd, J = 11.3, 5.7 Hz, 1H), 2.39-2.28 (m, 2H), 2.38 (s, 3H), 2.14-2.06 (m, 1H); ^{13}C NMR (100.7 MHz, $CDCl_3$) δ 80.6 (1), 62.4 (2), 54.6(2), 41.9 (3), 38.7 (3), 33.1 (2); HRMS (ESI$^+$): calculated for $C_6H_{14}NO_3S^+$: 180.0689 amu; found for $C_6H_{14}NO_3S^+$: 180.0689 amu.

(*3S*)-S-(1-Methylpyrrolidin-3-yl) ethanethioate: (*3R*)-3-(Mesyloxy)-1-methylpyrrolidine (2.35 g, 13.1 mmol) was dissolved in anhydrous DMF (44 mL) under an argon atmosphere. Potassium thioacetate (4.50 g, 39.4 mmol) was added in 3 portions, allowing the salt to dissolve before adding more. After the potassium thioacetate addition and dissolution, the reaction was heated to

30°C for 1hr. After this time, the reaction was cooled to room temperature, diluted with water (50 mL) and extracted with EtOAc (3 × 15 mL). The combined organic layers were washed with water (5 × 20 mL), dried over Na_2SO_3, and gravity filtered. The filtrate was concentrated *in vacuo* to afford a viscous red liquid (1.88 g). A portion of this material (0.462 g) was purified using flash column chromatography through silica gel (5% MeOH/DCM) to produce a viscous red liquid (0.3183 g, 40% yield).

Spectroscopic Data: IR(ATR): 2963, 2940, 2774, 1685, 1116, 953, 629 cm^{-1}; ^{1}H NMR (400 MHz, DMSO-*d6*) δ 3.82-3.76 (m, 1H), 2.78 (dd, J = 9.7, 7.3 Hz, 1H), 2.58-2.53 (m, 1H), 2.38-2.32 (m, 2H), 2.30-2.23 (m, 1H), 2.28 (s, 3H), 2.23 (s, 3H), 1.64-1.57 (m,1H); ^{13}C NMR (100.7 MHz, DMSO-*d6*) δ 195.8 (0), 62.2 (2), 54.8 (2), 41.4 (3), 39.8 (1), 31.7 (2), 30.3 (3); HRMS (ESI^+): calculated for $C_7H_{14}NOS^+$: 160.0791 amu; found for $C_7H_{14}NOS^+$: 160.0793 amu; HPLC purity at 230nm (75% CH_3CN : 10% CH_3OH : 15% aqueous triethylamine [0.1% triethylamine in H_2O], retention time 3.330 mins): 91.5 %.

(15) (3*S*)-*S*-(1-Methylpyrrolidin-3-yl) Ethanethioate IR(ATR)

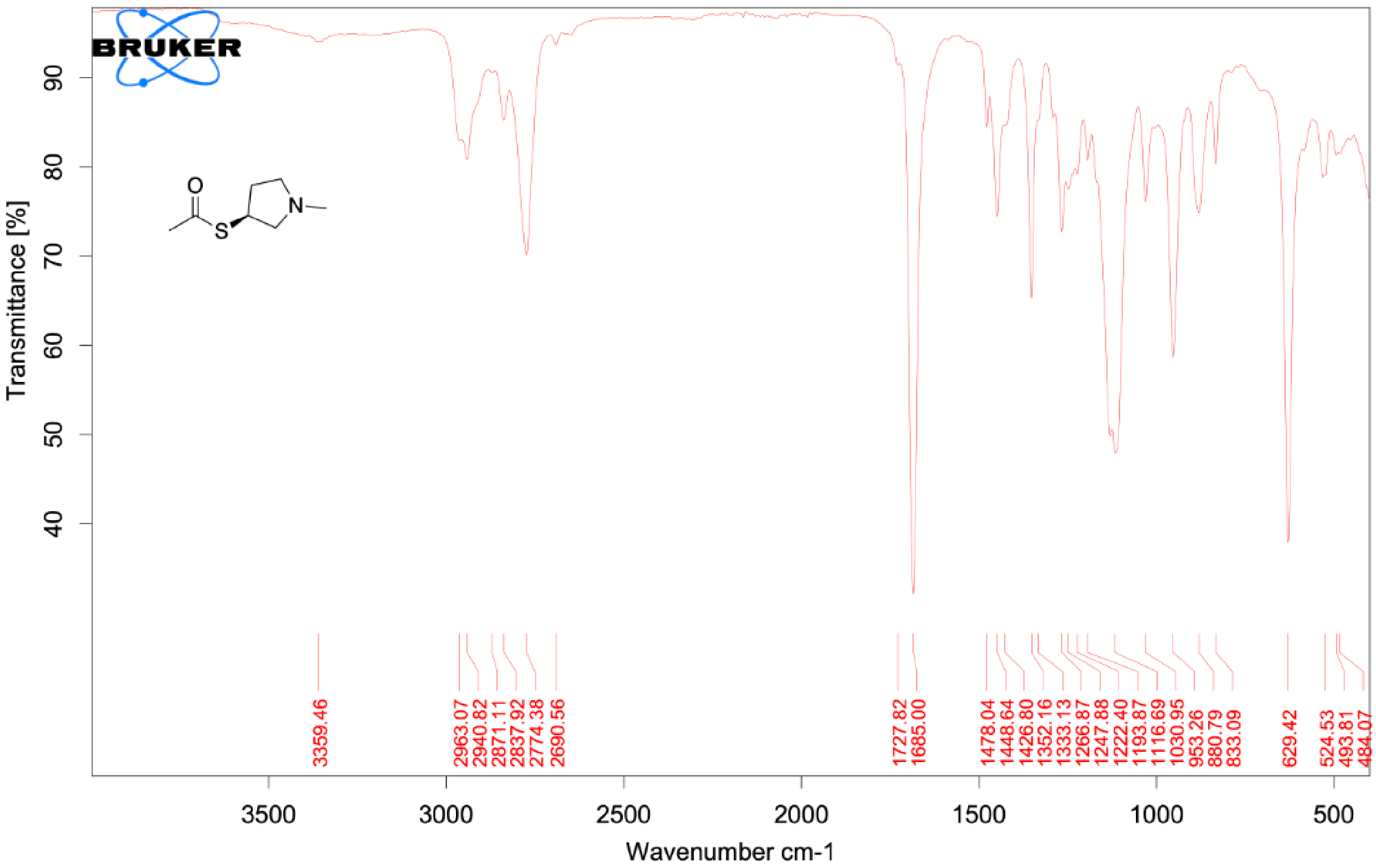

(15) (*3S*)-*S*-(1-Methylpyrrolidin-3-yl) Ethanethioate ^{1}H NMR 400 MHz (DMSO-*d6*)

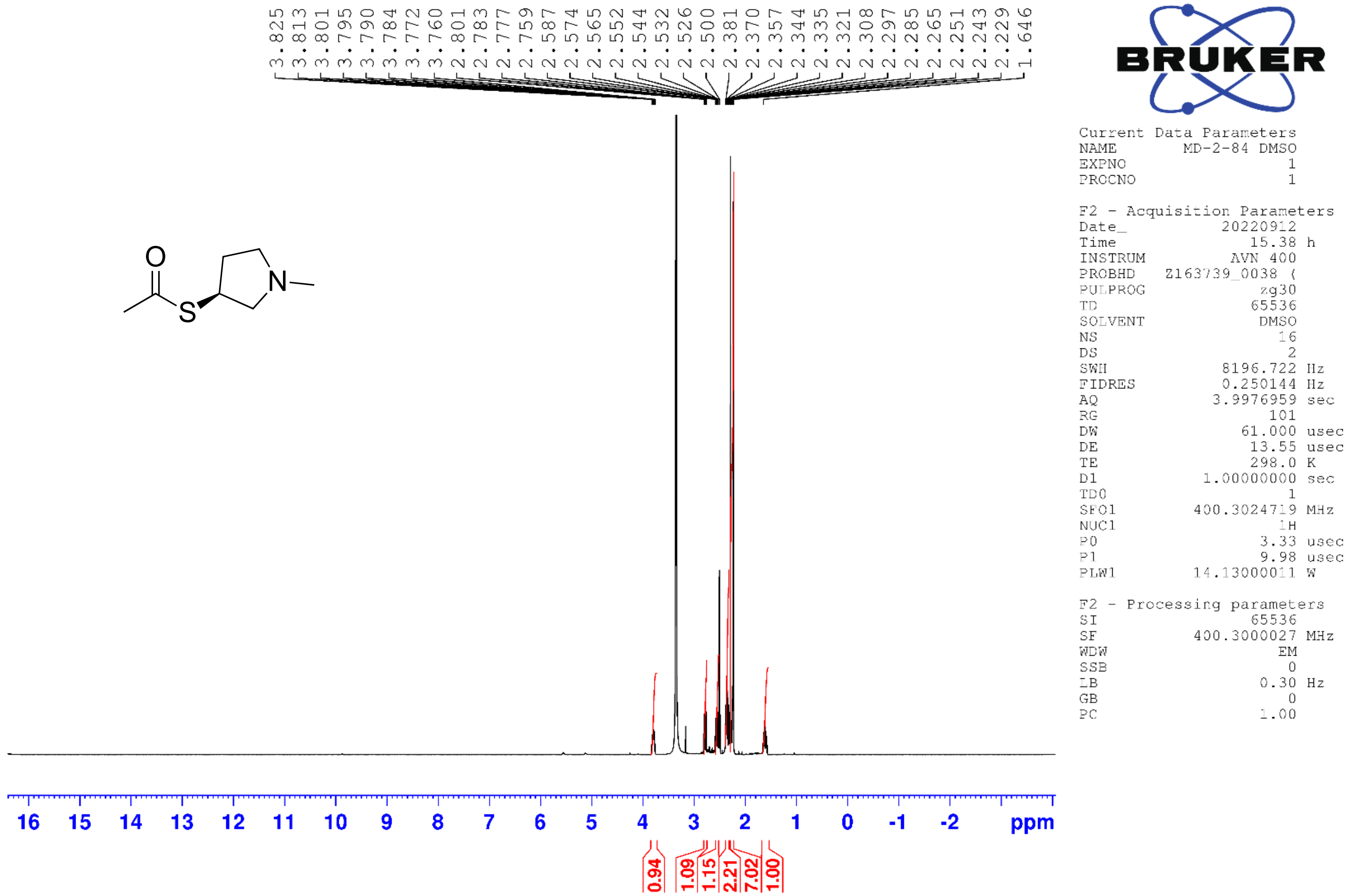

(15) (*3S*)-*S*-(1-Methylpyrrolidin-3-yl) Ethanethioate ^{13}C NMR 100 MHz (DMSO-*d6*)

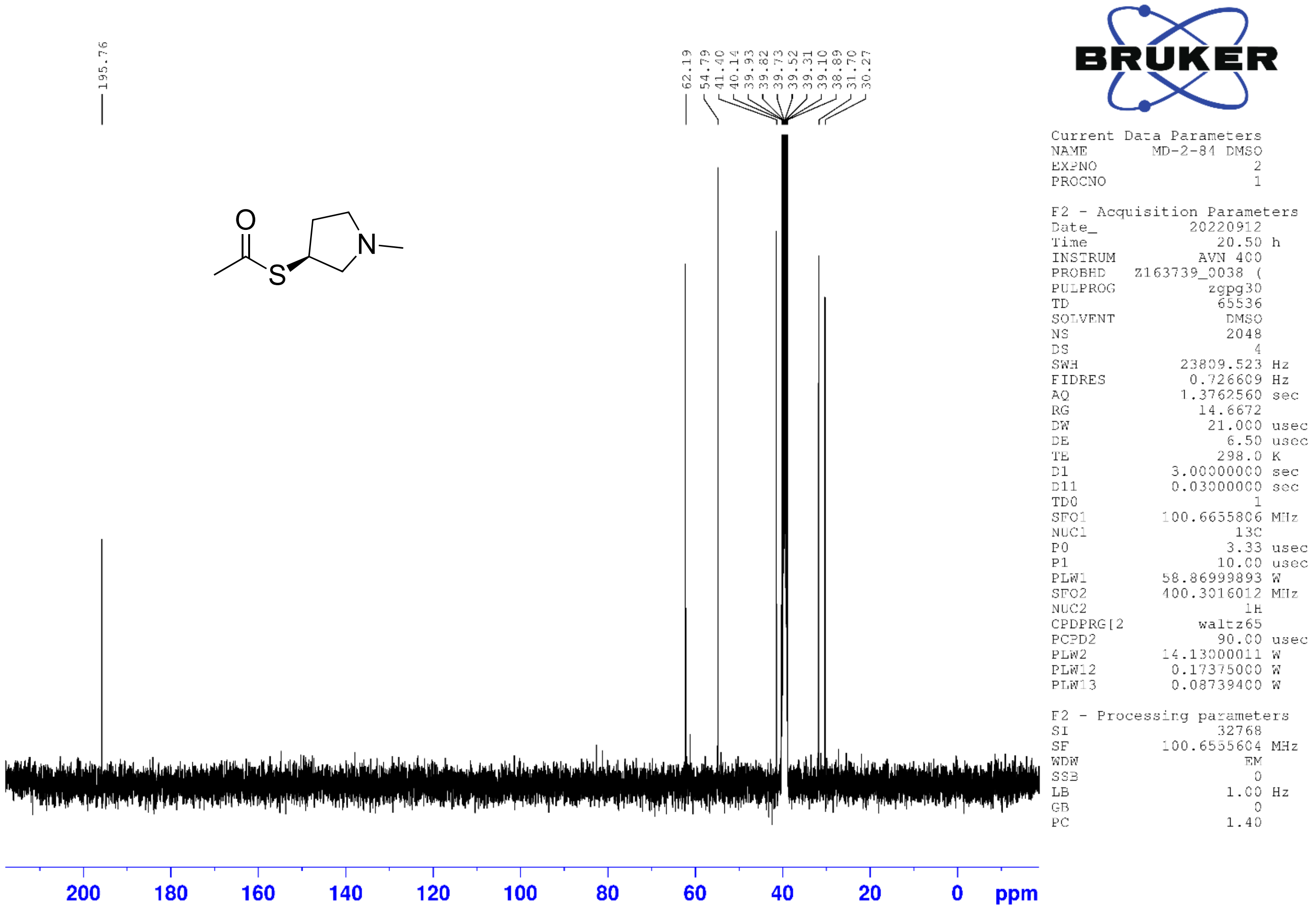

(15) (3*S*)-*S*-(1-Methylpyrrolidin-3-yl) Ethanethioate High Resolution Mass Spectrum (ESI, positive mode)

Mass Spectrum SmartFormula Report

Analysis Info

Analysis Name	D:\Data\Xiao\Aug 11 2022000018.d	Acquisition Date	2022-08-11 3:24:25 PM	
Method	Xiao all 1.m	Operator	x	
Sample Name	TRV-6019	Instrument	compact	8255754.20059
Comment				

Acquisition Parameter

Source Type	ESI	Ion Polarity	Positive	Set Nebulizer	0.5 Bar
Focus	Not active	Set Capillary	3500 V	Set Dry Heater	181 °C
Scan Begin	50 m/z	Set End Plate Offset	-500 V	Set Dry Gas	4.0 l/min
Scan End	1500 m/z	Set Charging Voltage	2000 V	Set Divert Valve	Source
		Set Corona	0 nA	Set APCI Heater	0 °C

Meas. m/z	Ion Formula	m/z	err [ppm]
160.0793	C7H14NOS	160.0791	-1.5

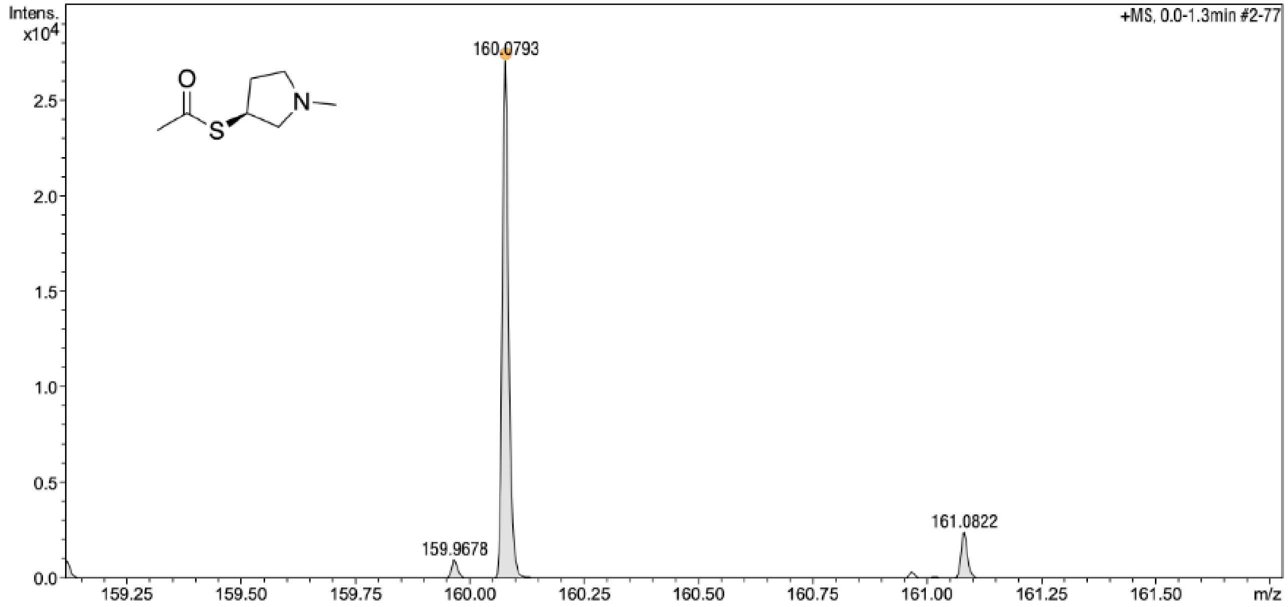

Aug 11 2022000018.d

Bruker Compass DataAnalysis 4.3 printed: 2022-08-11 3:28:29 PM by: x Page 1 of 1

(15) (3*S*)-*S*-(1-Methylpyrrolidin-3-yl) Ethanethioate HPLC

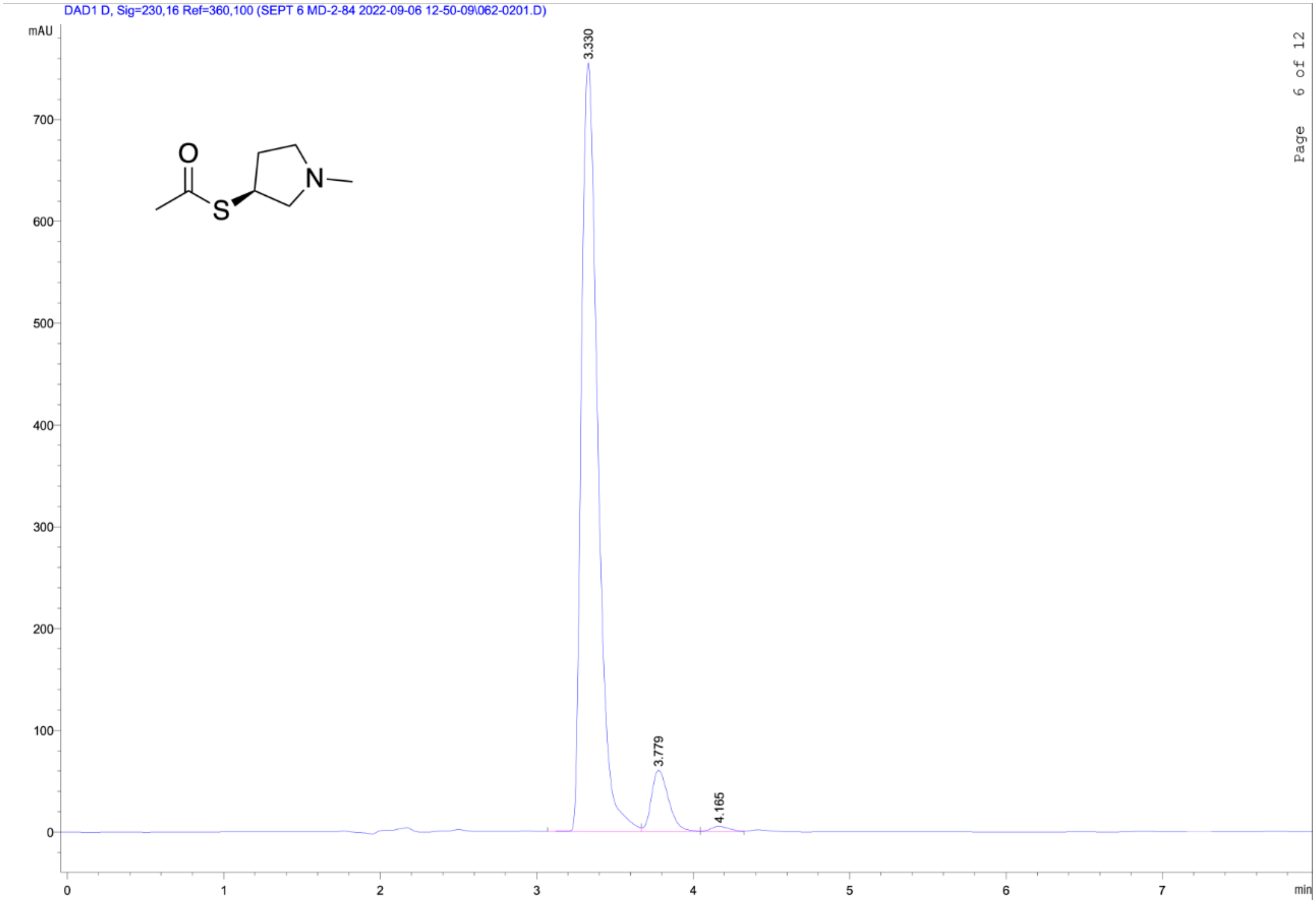

(16) *(3S)*-3-Acetoxy-1-methyl-1-pyrrolidinium Chloride

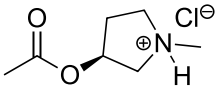

Synthesis: **(*S*)-1-Methyl-3-pyrrolidinyl acetate**: (*S*)-(+)-1-Methyl-3-pyrrolidinol (0.329 mL, 3 mmol) and triethylamine (1.05 mL, 7.5 mmol) were dissolved in anhydrous dichloromethane (10 mL) and cooled to 0°C with an ice bath. Acetyl chloride (0.213 mL, 3 mmol) was added dropwise at 0°C. The ice bath was removed after 5 mins, and the reaction was stirred for 6 hrs. The reaction was quenched with of brine (10 mL) and extracted with dichloromethane (3 × 15 mL). The combined organic layers were dried over Na_2SO_3, gravity filtered, and the filtrate was removed *in vacuo* to produce a yellow oil. The crude mixture was purified by silica gel column chromatography (5% MeOH/DCM) to produce (*S*)-1-methyl-3-pyrrolidinyl acetate (0.293 g, 68 %) as a yellow oil.

Spectroscopic data: ^{1}H NMR (400 MHz, DMSO-*d6*) δ 5.18-5.14 (m, 1H), 2.85-2.81 (m, 1H), 2.73 (dd, J = 10.9, 1.6 Hz, 1H), 2.57 (dd, J = 10.9, 5.9 Hz, 1H), 2.35 (s, 3H), 2.31-2.21 (m, 2H), 2.04 (s, 3H), 1.89-1.80 (1H); ^{13}C NMR (100.7 MHz, DMSO-*d6*) δ 171.1 (0), 74.8 (1), 62.3 (2), 55.1 (2), 42.1 (3), 32.7 (2), 21.4 (3).

***(3S)*-3-Acetoxy-1-methyl-1-pyrrolidinium chloride**: To a flask charged with (*S*)-1-Methyl-3-pyrrolidinyl acetate (0.124 g, 0.867 mmol) anhydrous diethyl ether was added until complete dissolution (17 mL) under an argon atmosphere. HCl in diethyl ether (1 N solution, 1.3 mL, 1.3 mmol) was added dropwise and stirred for 1 hr. The solvent was removed *in vacuo* resulting in a white solid. The compound was triturated three times with diethyl ether (20 mL), solvent was

removed with a pipet and compound placed under vacuum (1 mm Hg) to produce *(3S)*-3-acetoxy-1-methyl-1-pyrrolidinium chloride (0.141 g, 90%) as a white solid.

Spectroscopic data: IR(ATR): 3380, 2921, 2650, 2458, 1717, 1241, 1047 cm^{-1}; ^{1}H NMR (400 MHz, CD_3OD) δ 5.41-5.37 (m, 1H), 4.85 (br s, 2H), 3.67-3.55 (m, 2H), 3.42-3.38 (m, 1H), 2.98 (s, 3H), 2.58-2.47 (m, 1H), 2.25-2.17 (m, 1H), 2.09 (s, 3H); ^{13}C NMR (100.7 MHz, DMSO-*d6*) δ 171.7 (0), 73.8 (1), 61.7 (2), 55.5 (2), 41.9 (3), 31.7 (2), 20.8 (3); LRMS (ESI^+): 144.1 (M^+); HRMS (ESI^+): calculated for $C_7H_{14}NO_2^+$: 144.1019 amu; found for $C_7H_{14}INO_2^+$: 144.1022 amu; HPLC purity at 210nm (75% CH3CN : 10% CH3OH : 15% aqueous triethylamine [0.1% triethylamine in H_2O], retention time 3.066 mins): >99 %.

(16) *(3S)*-3-Acetoxy-1-methyl-1-pyrrolidinium Chloride IR(ATR)

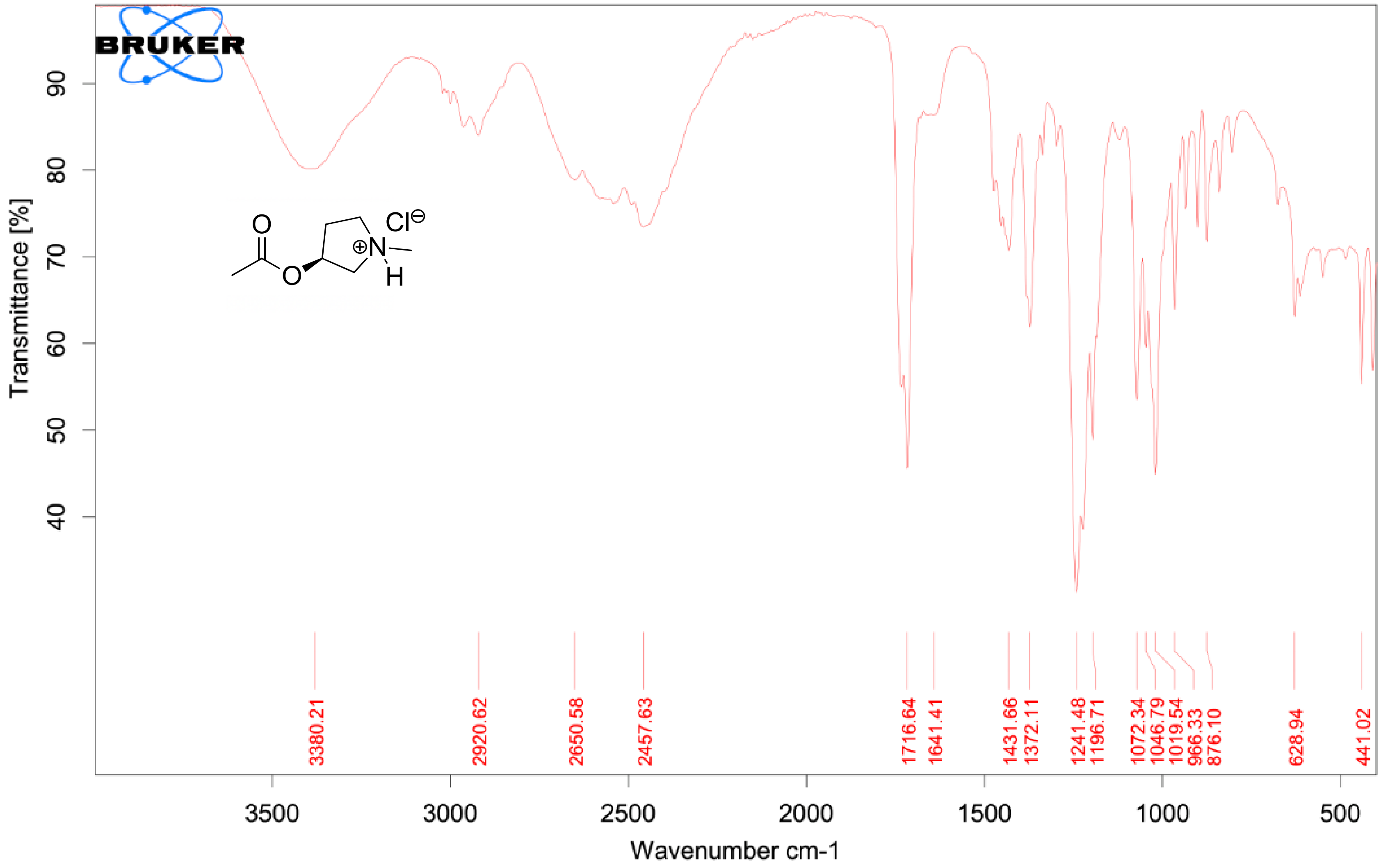

(16) *(3S)*-3-Acetoxy-1-methyl-1-pyrrolidinium Chloride ^{1}H NMR 400 MHz (CD_3OD-*d4*)

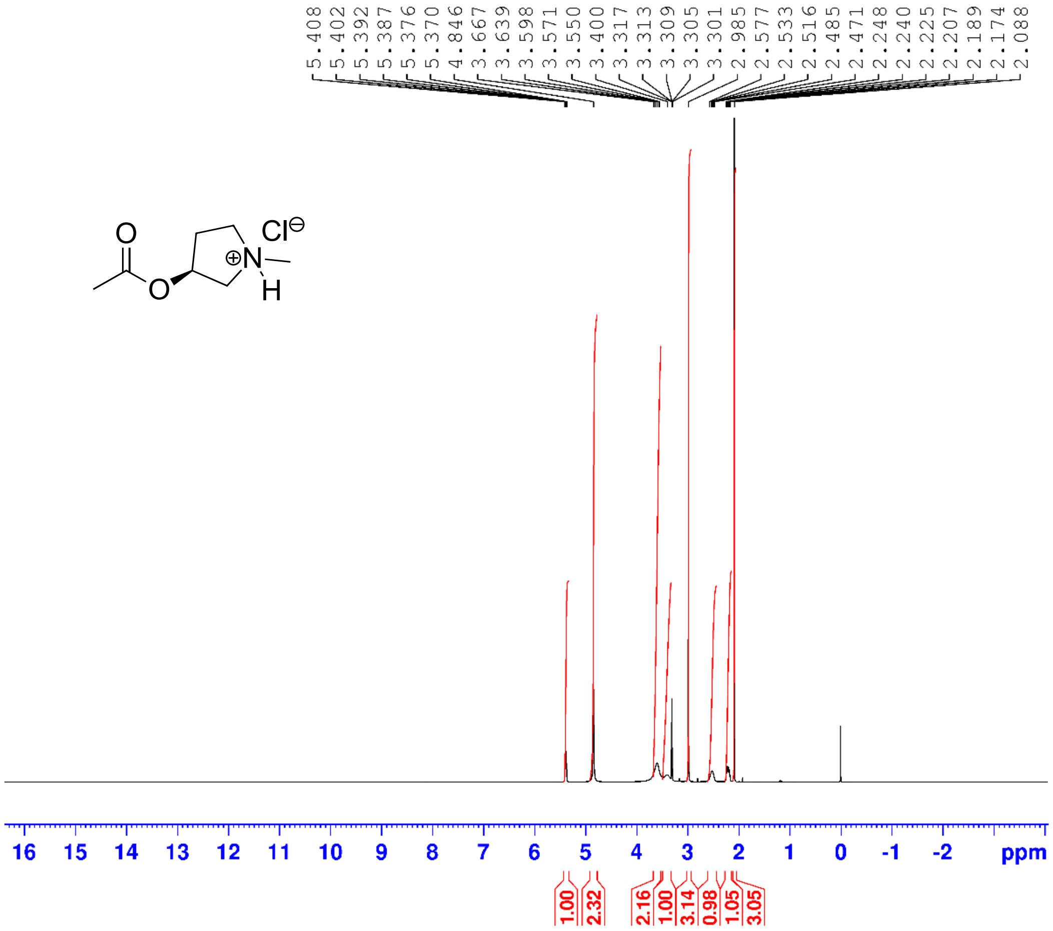

Current Data Parameters
NAME SB-1-132
EXPNO 8
PROCNO 1

F2 - Acquisition Parameters
Date_ 20220815
Time 16.14 h
INSTRUM AVN 400
PROBHD Z163739_0038 (
PULPROG zg30
TD 65536
SOLVENT MeOD
NS 16
DS 2
SWH 8196.722 Hz
FIDRES 0.250144 Hz
AQ 3.9976959 sec
RG 101
DW 61.000 usec
DE 13.63 usec
TE 298.0 K
D1 1.00000000 sec
TD0 1
SFO1 400.3024719 MHz
NUC1 1H
P0 3.16 usec
P1 9.49 usec
PLW1 14.13000011 W

F2 - Processing parameters
SI 65536
SF 400.3000081 MHz
WDW EM
SSB 0
LB 0.30 Hz
GB 0
PC 1.00

(16) *(3S)*-3-Acetoxy-1-methyl-1-pyrrolidinium Chloride ^{13}C NMR 100 MHz (CD_3OD-*d4*)

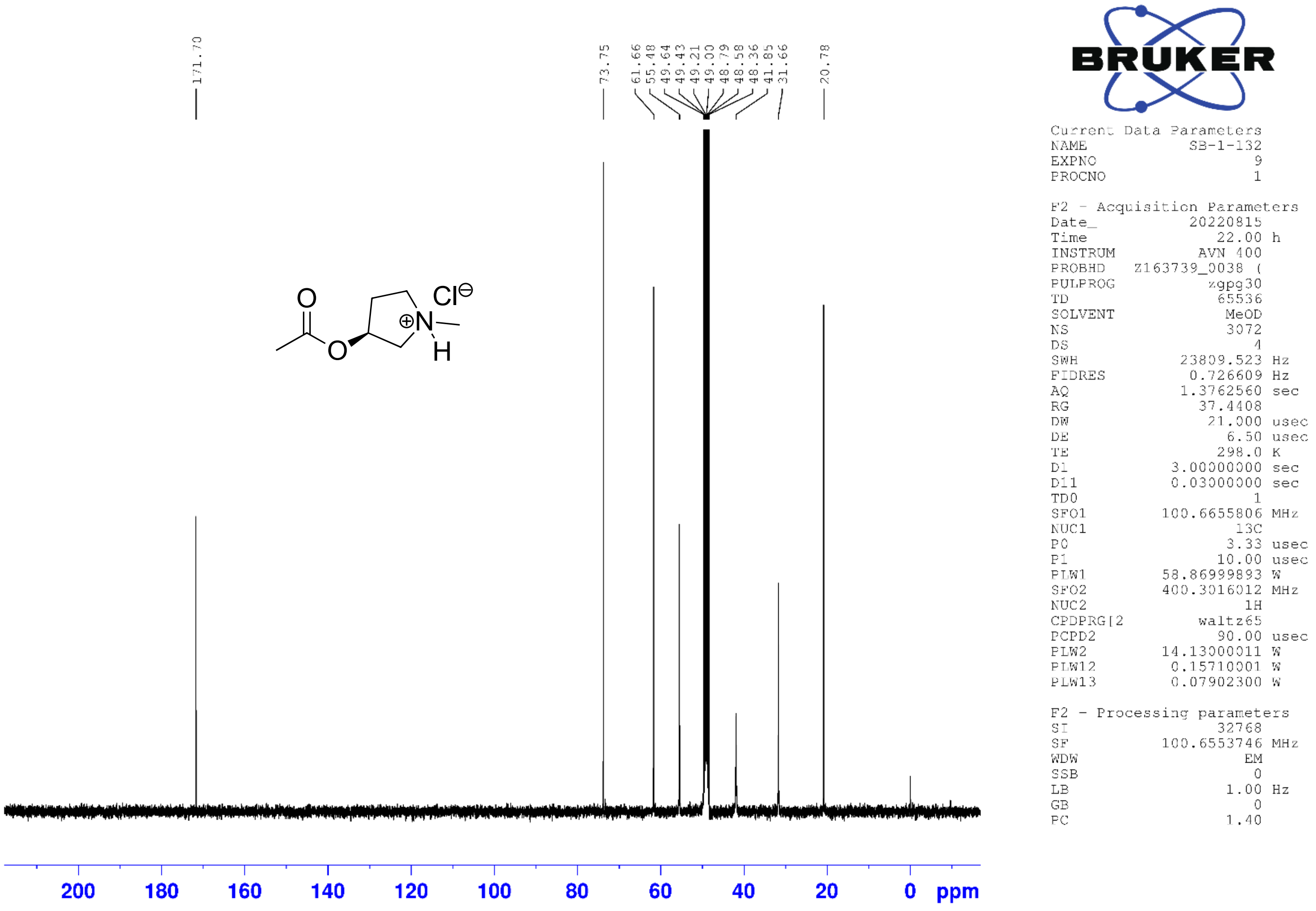

(16) *(3S)*-3-Acetoxy-1-methyl-1-pyrrolidinium Chloride High Resolution Mass Spectrum (ESI, positive mode)

Mass Spectrum SmartFormula Report

Analysis Info

Analysis Name	D:\Data\Xiao\July 07 2022000014.d	Acquisition Date	7/7/2022 10:04:14 AM
Method	Xiao all 1.m	Operator	x
Sample Name	TRV 6017 HCl	Instrument	compact 8255754.20059
Comment			

Acquisition Parameter

Source Type	ESI	Ion Polarity	Positive	Set Nebulizer	0.5 Bar
Focus	Not active	Set Capillary	3500 V	Set Dry Heater	180 °C
Scan Begin	50 m/z	Set End Plate Offset	-500 V	Set Dry Gas	4.0 l/min
Scan End	1500 m/z	Set Charging Voltage	2000 V	Set Divert Valve	Source
		Set Corona	0 nA	Set APCI Heater	0 °C

Meas. m/z	Ion Formula	m/z	err [ppm]
144.1022	C7H14NO2	144.1019	-2.0

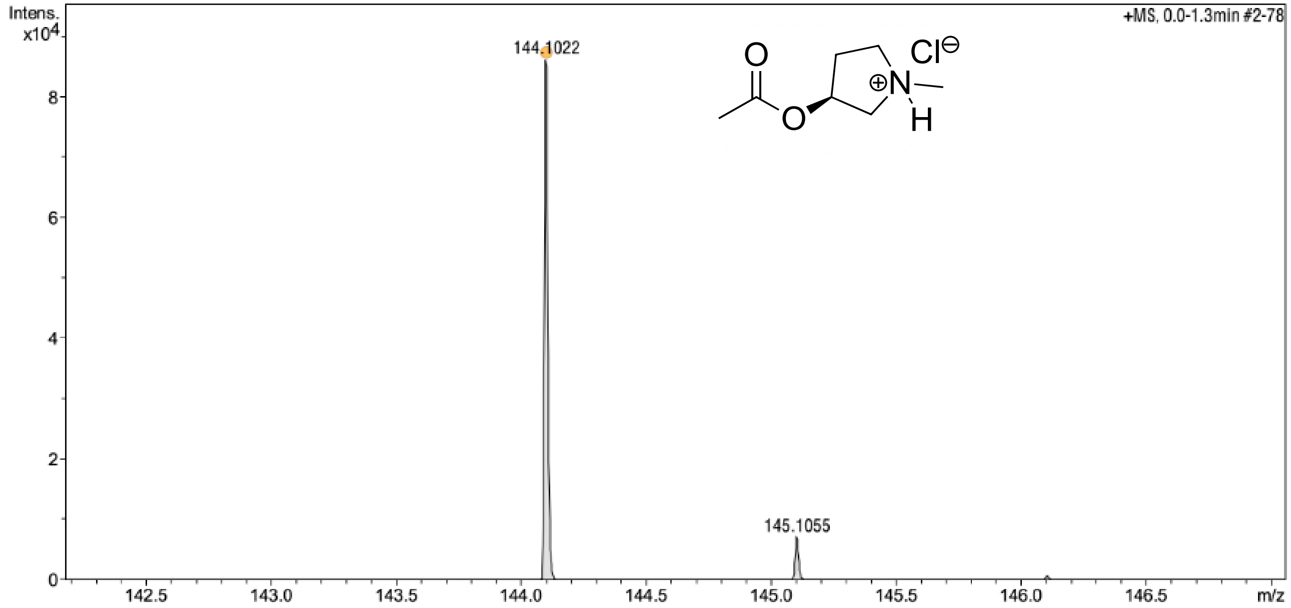

July 07 2022000014.d

Bruker Compass DataAnalysis 4.3 printed: 7/7/2022 10:07:12 AM by: x Page 1 of 1

(16) *(3S)*-3-Acetoxy-1-methyl-1-pyrrolidinium Chloride HPLC. Note: Noise from the injection has a retention time from 1.6 min until 2.5 min. The compound of interest has a retention time of 3.066 min.

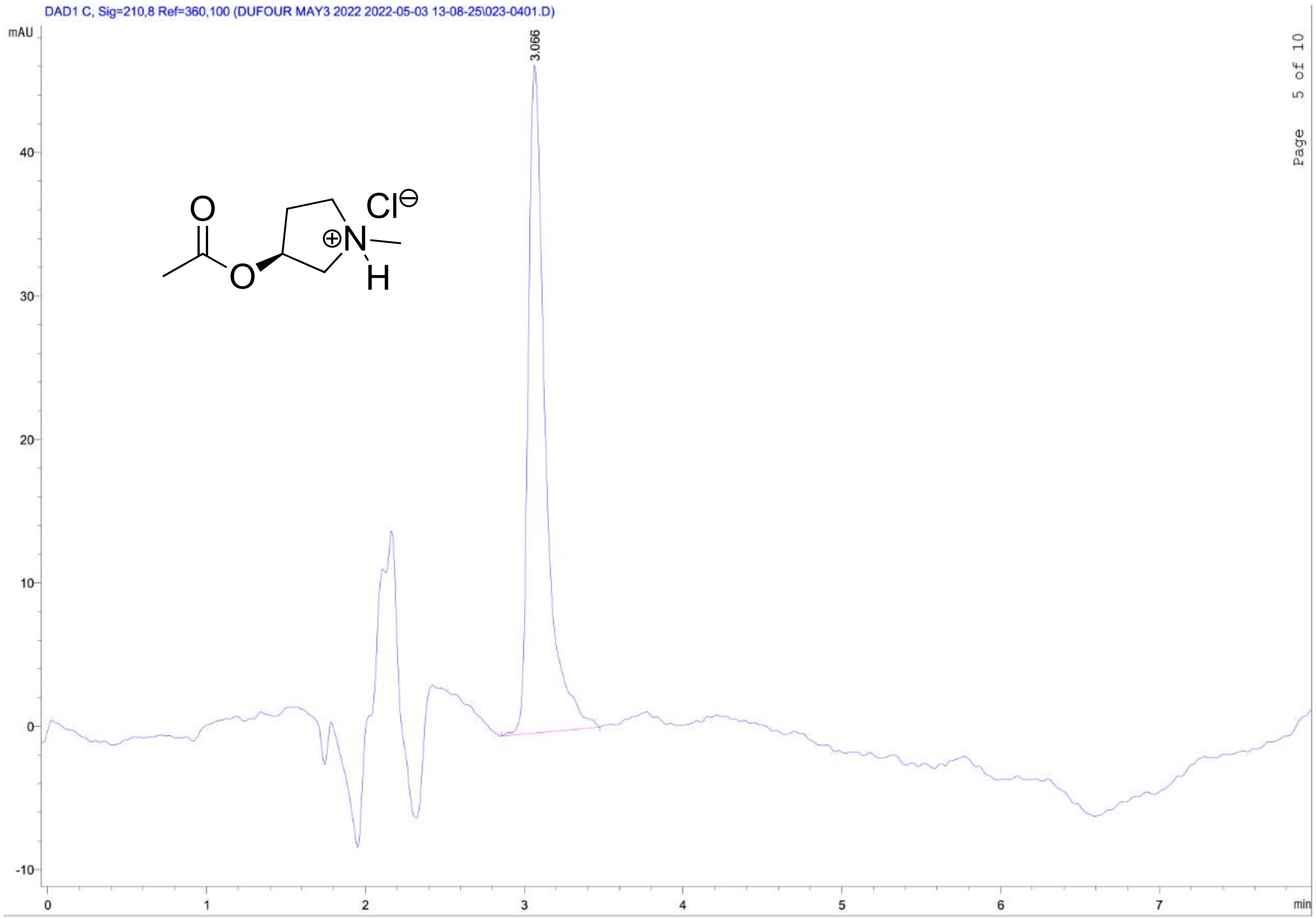

(17) (*3R*)-*S*-(1-Methylpyrrolidin-3-yl) Ethanethioate

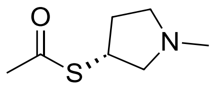

Synthesis: ***(3R)-S*-(1-methylpyrrolidin-3-yl) ethanethioate**: (*3S*)-(+)-1-Methyl-3-pyrrolidinol (6.00 mL, 54.6 mmol) was dissolved in dry dichloromethane (180 mL) and cooled in an ice bath. Triethylamine (11.4 mL, 81.9 mmol) was added dropwise. The mixture was stirred for 5 mins before the dropwise addition of methanesulfonyl chloride (4.90 mL, 62.6 mmol). The mixture was warmed slowly to room temperature and stirred for 16 hrs. The reaction was then quenched with the addition of brine (50 mL). The layers were separated, and the aqueous layer was back-extracted with dichloromethane (2 × 30 mL). The combined organics were washed with saturated $NaHCO_{3(aq)}$, dried over Na_2SO_4, gravity filtered, and the filtrate was concentrated to afford 8.01 g (82% yield) of the labile mesylate as an oil, which was used immediately without purification. The crude mesylate (6.80 g, 38.0 mmol) was charged to a round-bottom flask. To this was added 18-crown-6 ether (15.1 g, 57.0 mmol), THF (125 mL), and then potassium thioacetate (6.50 g, 57.0 mmol) in one portion. The mixture was heated to reflux and stirred overnight for 16 hrs. After cooling to room temperature, the reaction was diluted with H_2O (60 mL) and extracted with ethyl acetate (3 x 60 mL). The combined organic layers were washed with saturated $NH_4Cl_{(aq)}$, H_2O (3 × 60 mL), brine (60 mL), and dried with Na_2SO_4. The mixture was gravity filtered and the filtrate was concentrated to give 3.59 g of a purple oil. Vacuum distillation (heating at 115°C at 1 mmHg and collecting the distillate at 74-76°C) afforded 2.43 g of an orange oil. This material was then dissolved in ethyl acetate and extracted with 1N HCl (2 x 15 mL). The combined aqueous extracts were basified by the dropwise addition of 2N Na_2CO_3(aq) and then quickly extracted with dichloromethane (3 x 30 mL). The combined dichloromethane

extracts were washed with brine, dried (Na_2SO_4), gravity filtered, and the filtrate was concentrated *in vacuo* to give 1.57 g of a yellow oil. This material was dissolved in *i*-PrOH (100 mL) and treated with oxalic acid (0.875 g, 9.72 mmol). After stirring for 2 hrs, the thick white precipitate was collected by filtration, washing with *i*-PrOH (50 mL). The crude solid was then recrystallized from *i*-PrOH to give 1.85 g of a yellowish solid, which was immediately treated with 50 mL of saturated $NaHCO_3$(aq) and then extracted with DCM (3 x 60 mL). The combined organic extracts were washed with brine, dried (Na_2SO_4), filtered and concentrated to give (*3R*)-*S*-(1-methylpyrrolidin-3-yl) ethanethioate (0.985 g,16% yield) as a yellow viscous liquid.

Spectroscopic data: IR(ATR) 2962, 2941, 2774, 1685, 1116, 629 cm^{-1}; ^{1}H NMR (400 MHz, $CDCl_3$) δ 3.95-3.88 (m, 1H), 2.87 (dd, J = 10.1, 7.1 Hz, 1H), 2.71-2.64 (m, 1H), 2.51 (dd, J = 10.1, 4.6 Hz, 1H), 2.44-2.36 (m, 2H), 2.34 (s, 3H), 2.29 (s, 3H), 1.78-1.71 (m, 1H); ^{13}C NMR (100.7 MHz, CD_3OD-*d4*) δ 196.5 (0), 63.0 (2), 55.6 (2), 42.0 (3), 40.5(1), 32.1 (2), 30.5 (3); LRMS (ESI^+): 160.1 (M^+); HRMS (ESI^+): calculated for $C_7H_{14}NOS^+$: 160.0791 amu; found for $C_7H_{14}NOS^+$: 160.0788 amu; HPLC purity at 230 nm (25% CH_3CN : 75% CH_3OH, retention time: 3.323 mins): 95%.

(17) (*3R*)-*S*-(1-Methylpyrrolidin-3-yl) Ethanethioate IR(ATR)

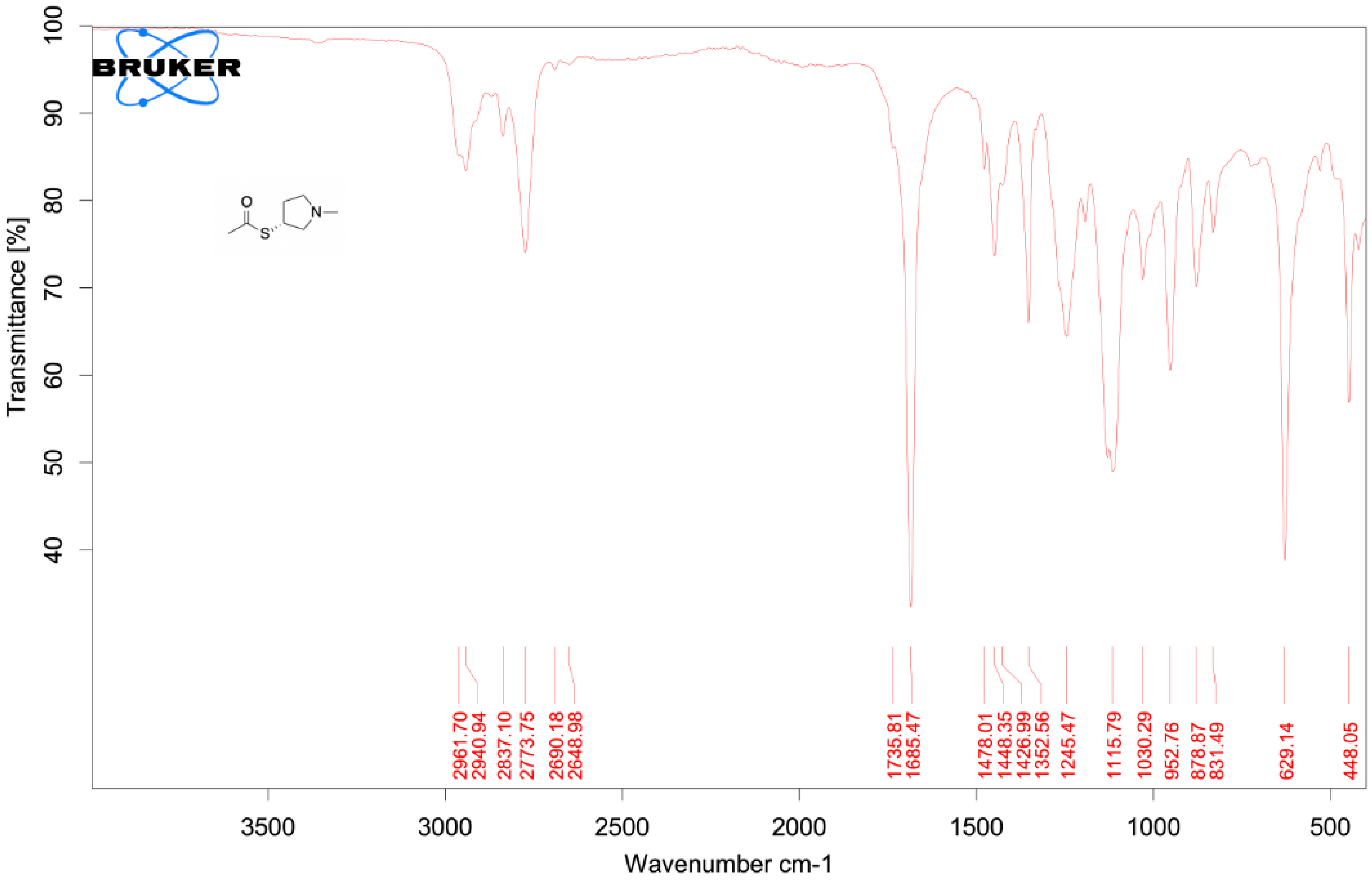

(17) (*3R*)-*S*-(1-Methylpyrrolidin-3-yl) Ethanethioate ^{1}H NMR 400 MHz ($CDCl_3$)

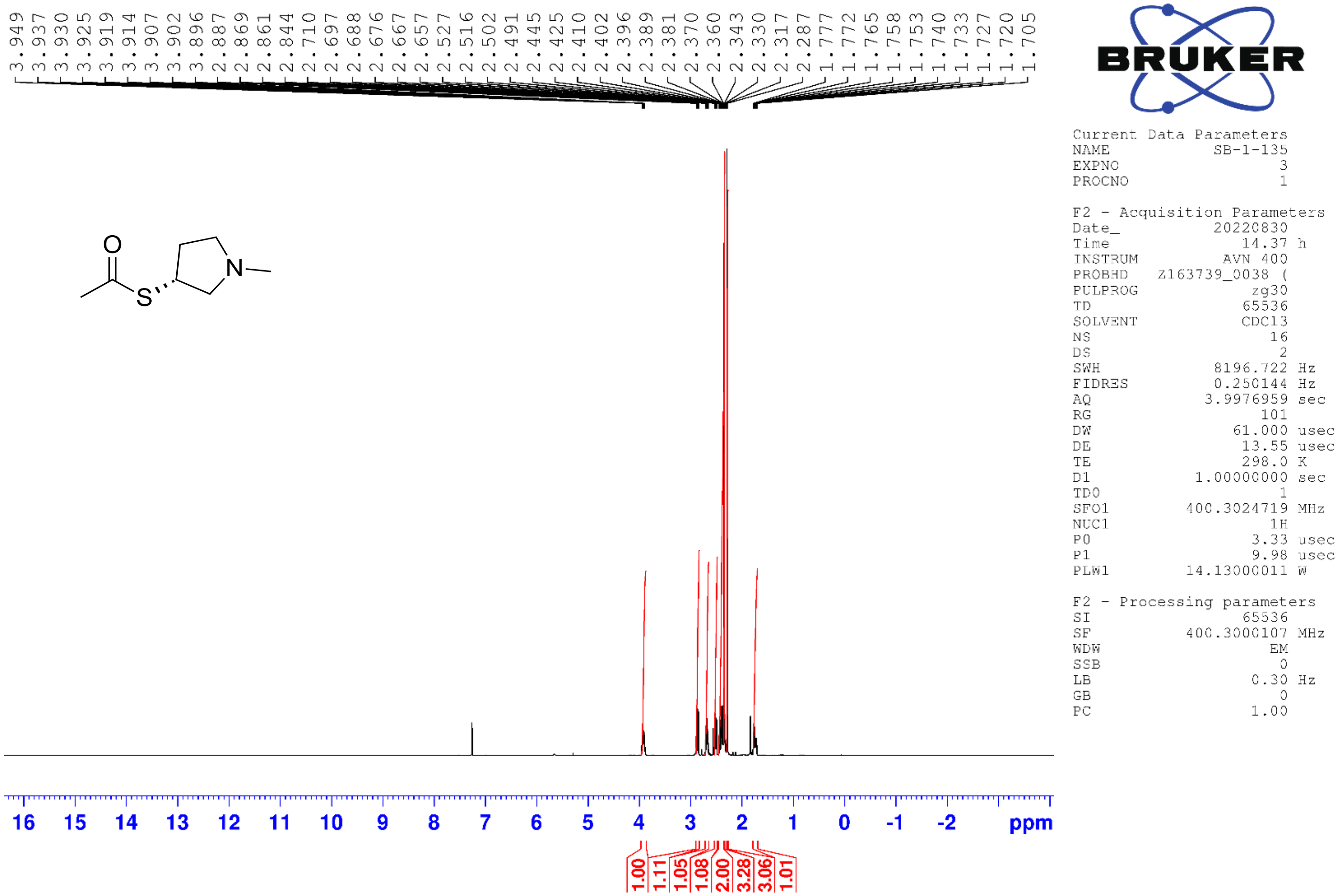

(17) (*3R*)-*S*-(1-Methylpyrrolidin-3-yl) Ethanethioate ^{13}C NMR 100 MHz ($CDCl_3$)

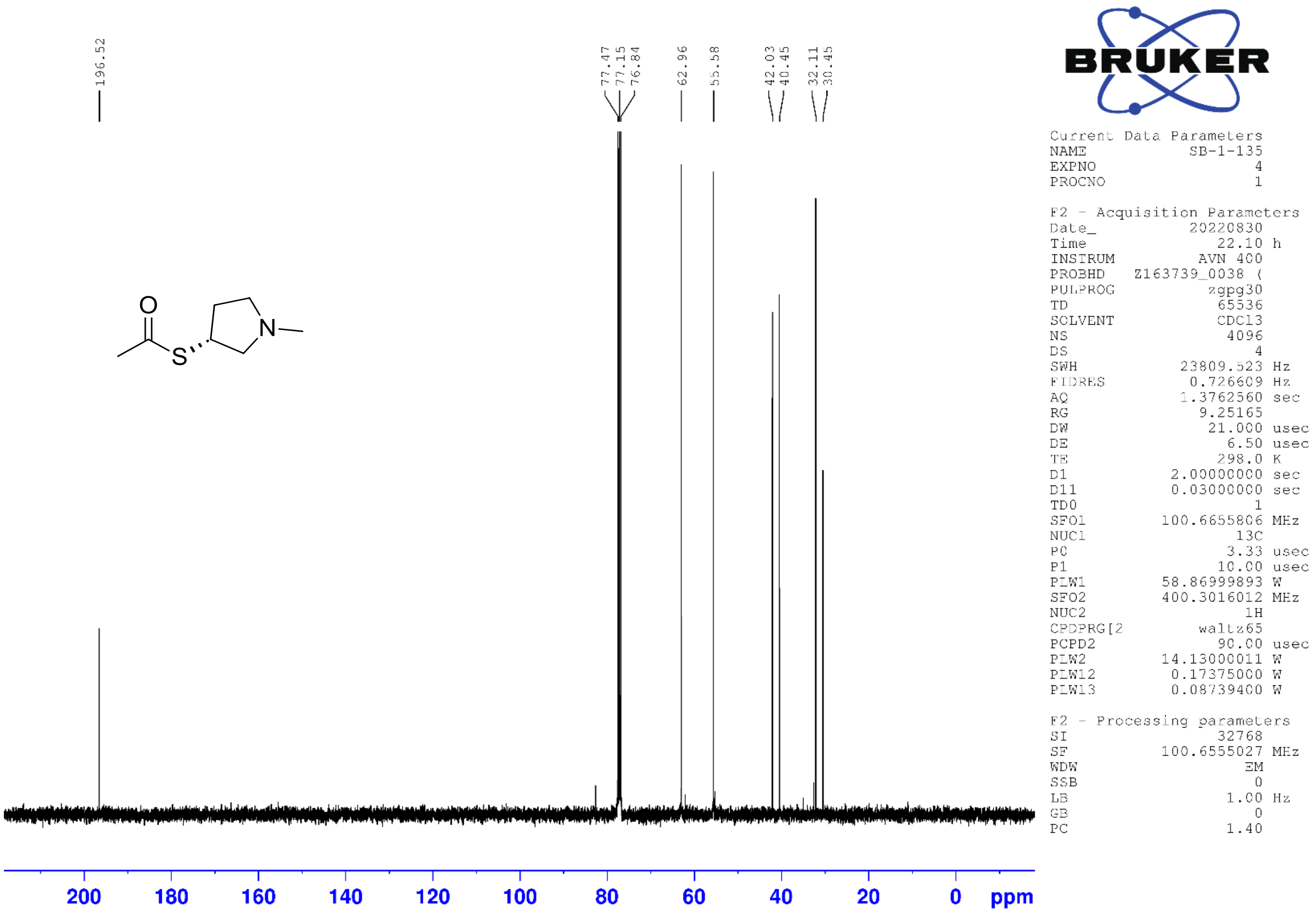

(17) (3*R*)-*S*-(1-Methylpyrrolidin-3-yl) Ethanethioate High Resolution Mass Spectrum (ESI, positive mode)

Mass Spectrum SmartFormula Report

Analysis Info

Analysis Name	D:\Data\Xiao\Oct 14 2022000001.d	Acquisition Date	2022-10-14 10:19:36 AM
Method	Xiao all 1.m	Operator	x
Sample Name	TRV 6020	Instrument	compact 8255754.20059
Comment			

Acquisition Parameter

Source Type	ESI	Ion Polarity	Positive	Set Nebulizer	0.5 Bar
Focus	Not active	Set Capillary	3500 V	Set Dry Heater	181 °C
Scan Begin	50 m/z	Set End Plate Offset	-500 V	Set Dry Gas	4.0 l/min
Scan End	1500 m/z	Set Charging Voltage	2000 V	Set Divert Valve	Source
		Set Corona	0 nA	Set APCI Heater	0 °C

Meas. m/z	Ion Formula	m/z	err [ppm]
160.0788	C7H14NOS	160.0791	1.4

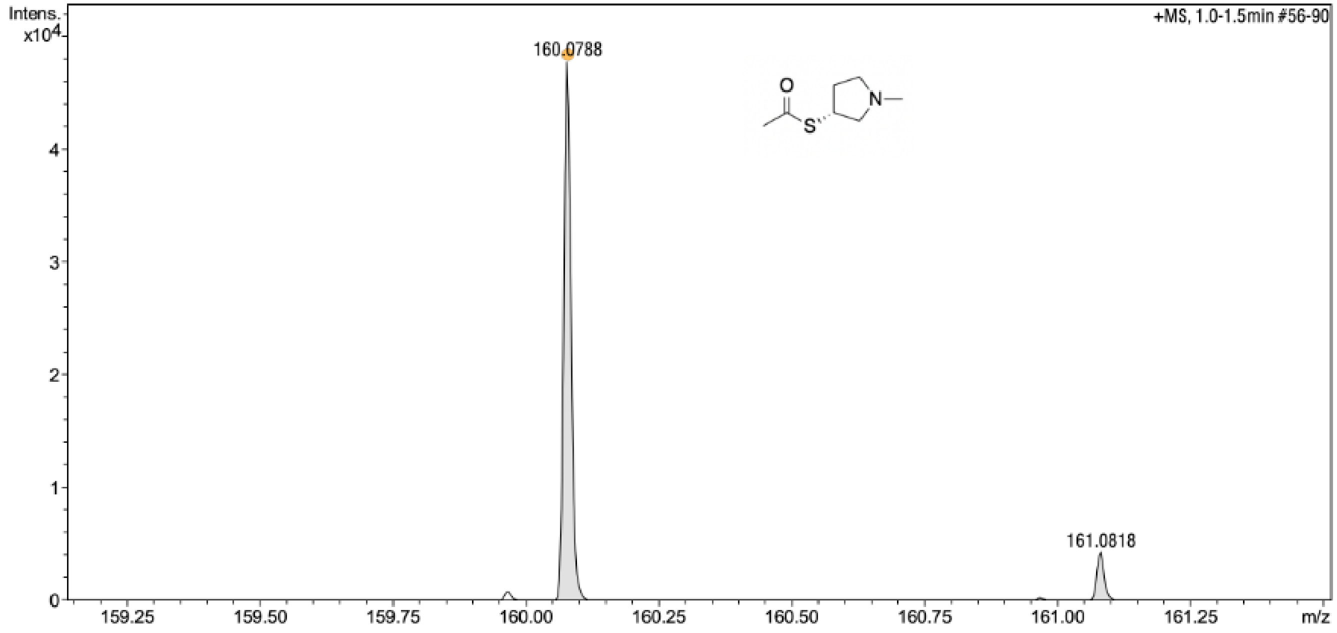

Oct 14 2022000001.d

Bruker Compass DataAnalysis 4.3 printed: 2022-10-14 10:22:58 AM by: x Page 1 of 1

(17) (*3R*)-*S*-(1-Methylpyrrolidin-3-yl) Ethanethioate HPLC

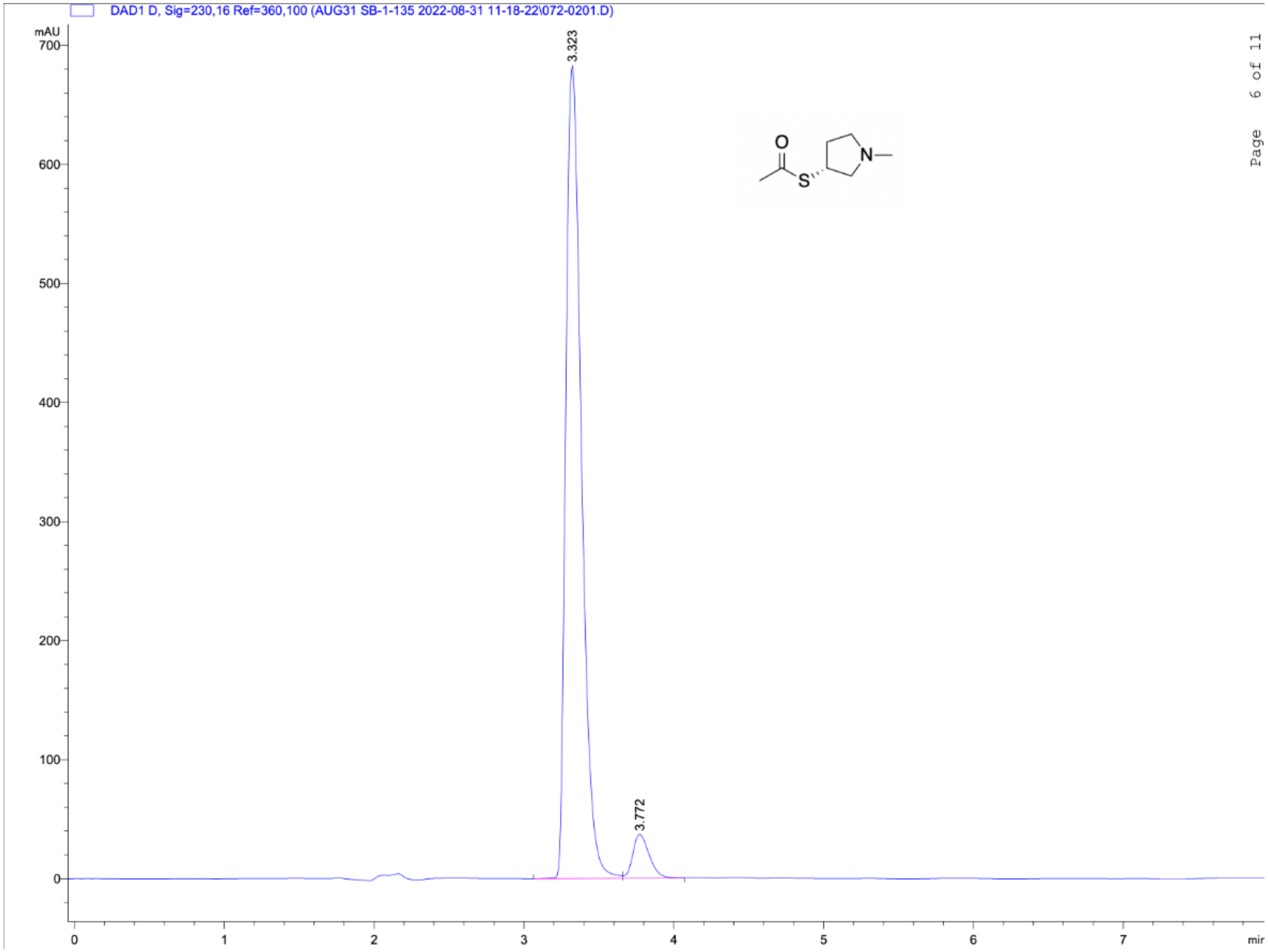

(18) *(3R)*-3-Acetoxy-1-methyl-1-pyrrolidinium Chloride

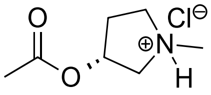

Synthesis: **(*3R*)-1-Methyl-3-pyrrolidinyl acetate**: (*3R*)-(-)-1-Methyl-3-pyrrolidinol (0.549 mL, 5.00 mmol) and triethylamine (1.74 mL, 12.5 mmol) were dissolved in anhydrous dichloromethane (17 mL) and cooled to 0°C with an ice bath. Acetyl chloride (0.356 mL, 5.00 mmol) was added dropwise at 0°C. The ice bath was removed after 5 min, and the reaction was stirred for 3 hrs. The reaction was quenched with 20 mL of brine, extracted with DCM (3×15 mL) and dried over Na_2SO_3. After gravity filtration of the drying agent, the solvent was removed *in vacuo* to produce a yellow oil. The crude mixture was purified by silica gel column chromatography (5% MeOH/DCM) to produce (*3R*)-1-methyl-3-pyrrolidinyl acetate (0.227 g, 32%) as a yellow oil.

Spectroscopic data: IR(ATR) 2944, 2839, 2780, 1735, 1236, 887cm^{-1}; ^{1}H NMR (400 MHz, $CDCl_3$) δ 5.21-5.16 (m, 1H), 2.89-2.84 (m, 1H), 2.73 (d, J = 10.6 Hz, 1H), 2.58 (dd, J = 10.9, 5.9 Hz, 1H), 2.36 (s, 3H), 2.30-2.23 (m, 2H), 2.04 (s, 3H), 1.89-1.83 (m, 1H); ^{13}C NMR (100.7 MHz, $CDCl_3$) δ 171.2 (0), 74.8 (1), 62.3 (2), 55.1 (2), 42.1 (3), 32.7 (2), 21.4 (3); HRMS (ESI$^+$): calculated for $C_7H_{14}NO_2^+$: 144.1019 amu; found for $C_7H_{14}NO_2^+$: 144.1019 amu; HPLC purity at 200 nm (25% CH_3CN : 75% CH_3OH, retention time: 3.09 mins): >99%.

***(3R)*-3-Acetoxy-1-methyl-1-pyrrolidinium Chloride**: To a flask charged with (*3R*)-1-methyl-3-pyrrolidinyl acetate (0.122 g, 0.85 mmol) anhydrous diethyl ether was added until complete dissolution (17 mL) under an argon atmosphere. HCl in diethyl ether (1 N - 1.28 mL, 1.28 mmol) was added dropwise and stirred for 1hr. The solvent was removed *in vacuo* resulting in a white

solid. The compound was triturated with diethyl ether (20 mL), and the solvent was removed with a pipet. This process was repeated two more times. The compound was dried *in vacuo* to produce *(3R)*-3-acetoxy-1-methyl-1-pyrrolidinium chloride (0.146 g, 96%).

Spectroscopic data: $MP_{(diethyl\ ether)}$: 90-93°C; IR(ATR) 2915, 2647, 2440, 1717, 1246, 1224, 967 cm^{-1}; ^{1}H NMR (400 MHz, CD_3OD-*d4*) δ 5.41-5.37 (m, 1H), 3.60 (app br s, 3H), 3.40 (app br s, 1H), 2.98 (s, 3H), 2.57-2.48 (m, 1H), 2.25-2.17 (m, 1H), 2.09 (s, 3H); ^{13}C NMR (100.7 MHz, CD_3OD-*d4*) δ 171.7 (0), 73.8 (1), 61.7 (2), 55.5 (2), 41.9 (3), 31.7 (2), 20.8 (3); LRMS (ESI^+): 144.1 (M^+); HRMS (ESI^+): calculated for $C_7H_{14}NO_2^+$: 144.1019 amu; found for $C_7H_{14}NO_2^+$: 144.1018 amu; HPLC purity at 230 nm (25% CH_3CN : 75% CH_3OH, retention time: 3.097 mins): >99%.

(18) *(3R)*-3-Acetoxy-1-methyl-1-pyrrolidinium Chloride IR(ATR)

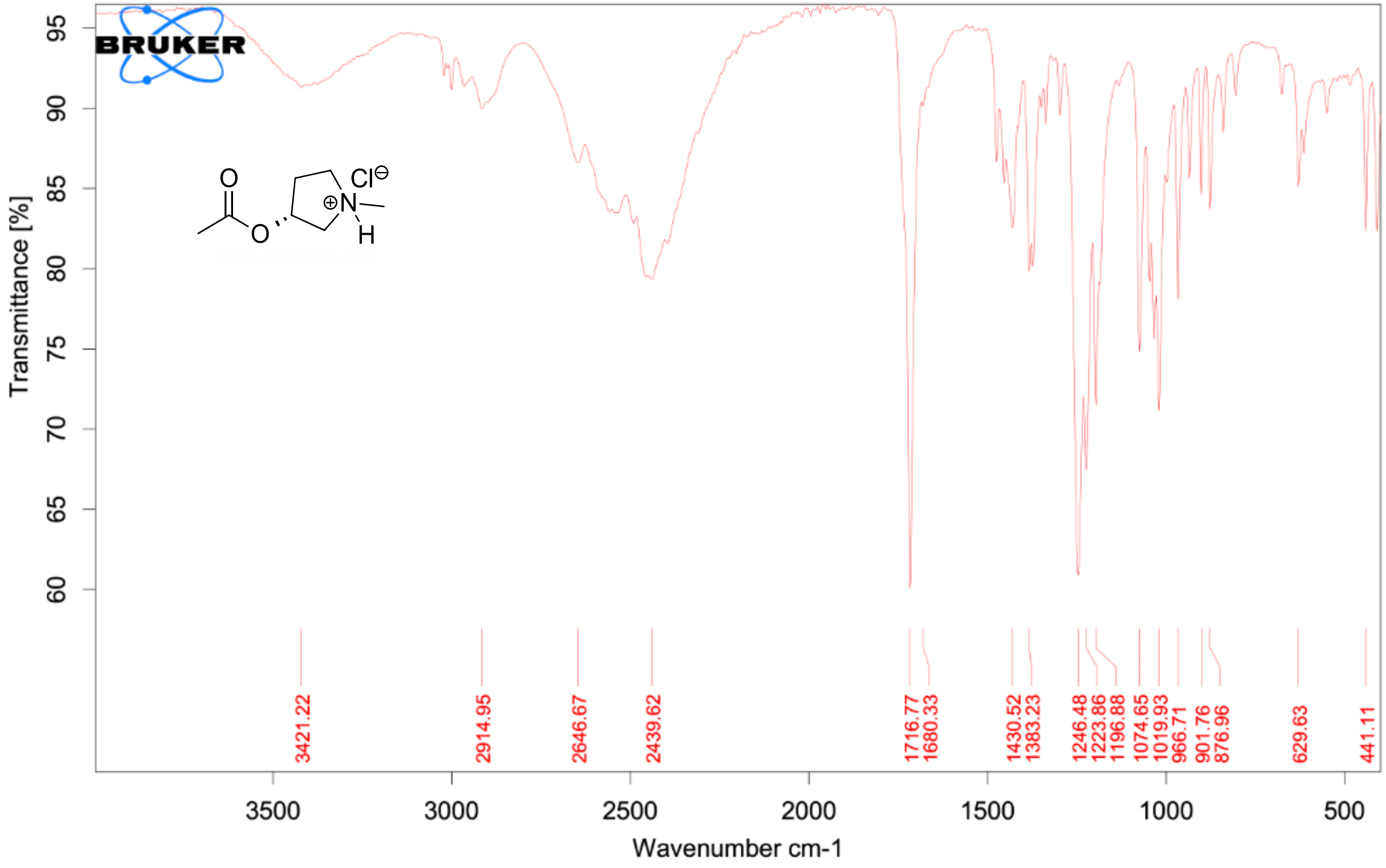

(18) *(3R)*-3-Acetoxy-1-methyl-1-pyrrolidinium Chloride ^{1}H NMR 400 MHz (CD$_3$OD-*d4*)

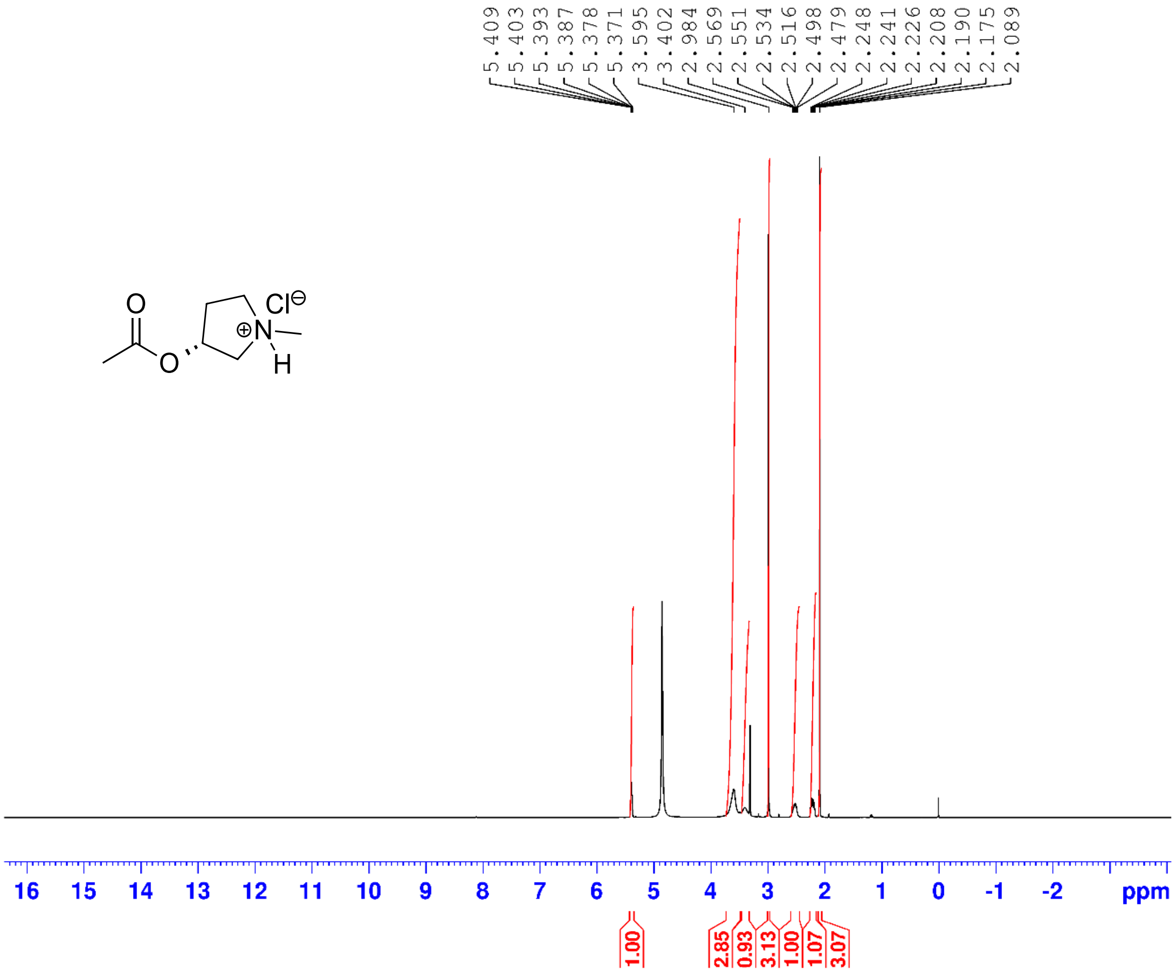

```
Current Data Parameters
NAME          MD-2-67 MeOD
EXPNO                    8
PROCNO                   1

F2 - Acquisition Parameters
Date_             20220803
Time                 12.02 h
INSTRUM            AVN 400
PROBHD     Z163739_0038 (
PULPROG               zg30
TD                   65536
SOLVENT               MeOD
NS                      16
DS                       2
SWH               8196.722 Hz
FIDRES            0.250144 Hz
AQ               3.9976959 sec
RG                     101
DW                  61.000 usec
DE                   13.54 usec
TE                   298.0 K
D1              1.00000000 sec
TD0                      1
SFO1           400.3024719 MHz
NUC1                    1H
P0                    3.33 usec
P1                   10.00 usec
PLW1           14.12800026 W

F2 - Processing parameters
SI                   65536
SF             400.3000077 MHz
WDW                     EM
SSB                      0
LB                    0.30 Hz
GB                       0
PC                    1.00
```

(18) *(3R)*-3-Acetoxy-1-methyl-1-pyrrolidinium Chloride ^{13}C NMR 100 MHz (CD_3OD-*d4*)

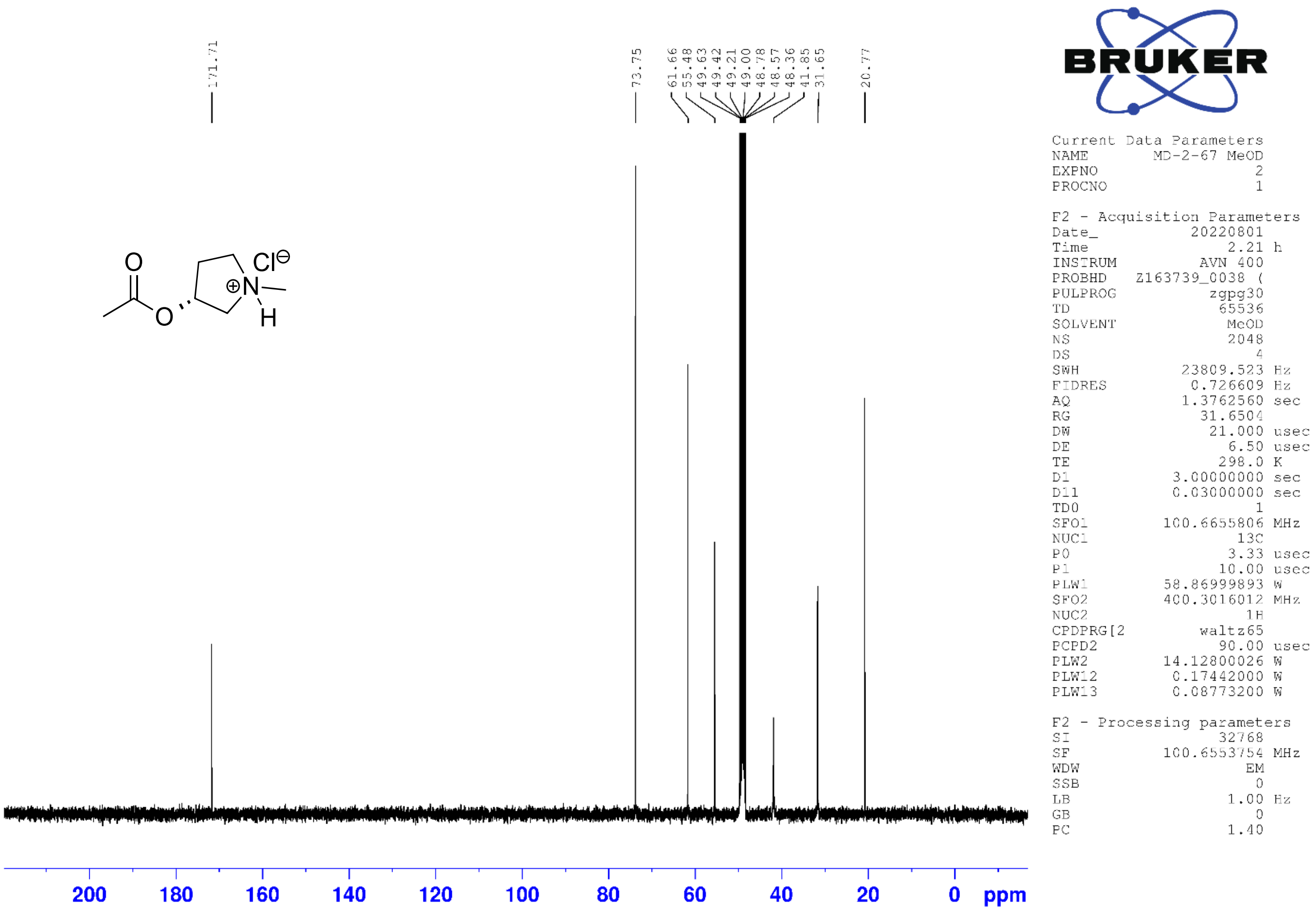

(18) *(3R)*-3-Acetoxy-1-methyl-1-pyrrolidinium Chloride High Resolution Mass Spectrum (ESI, positive mode)

Mass Spectrum SmartFormula Report

Analysis Info

Analysis Name	D:\Data\Xiao\July 06 2022000010.d	Acquisition Date	7/6/2022 2:53:34 PM
Method	Xiao all 1.m	Operator	x
Sample Name	TRV 6018 HCl	Instrument	compact 8255754.20059
Comment			

Acquisition Parameter

Source Type	ESI	Ion Polarity	Positive	Set Nebulizer	0.5 Bar
Focus	Not active	Set Capillary	3500 V	Set Dry Heater	181 °C
Scan Begin	50 m/z	Set End Plate Offset	-500 V	Set Dry Gas	4.0 l/min
Scan End	1500 m/z	Set Charging Voltage	2000 V	Set Divert Valve	Source
		Set Corona	0 nA	Set APCI Heater	0 °C

Meas. m/z	Ion Formula	m/z	err [ppm]
144.1018	C7H14NO2	144.1019	0.6

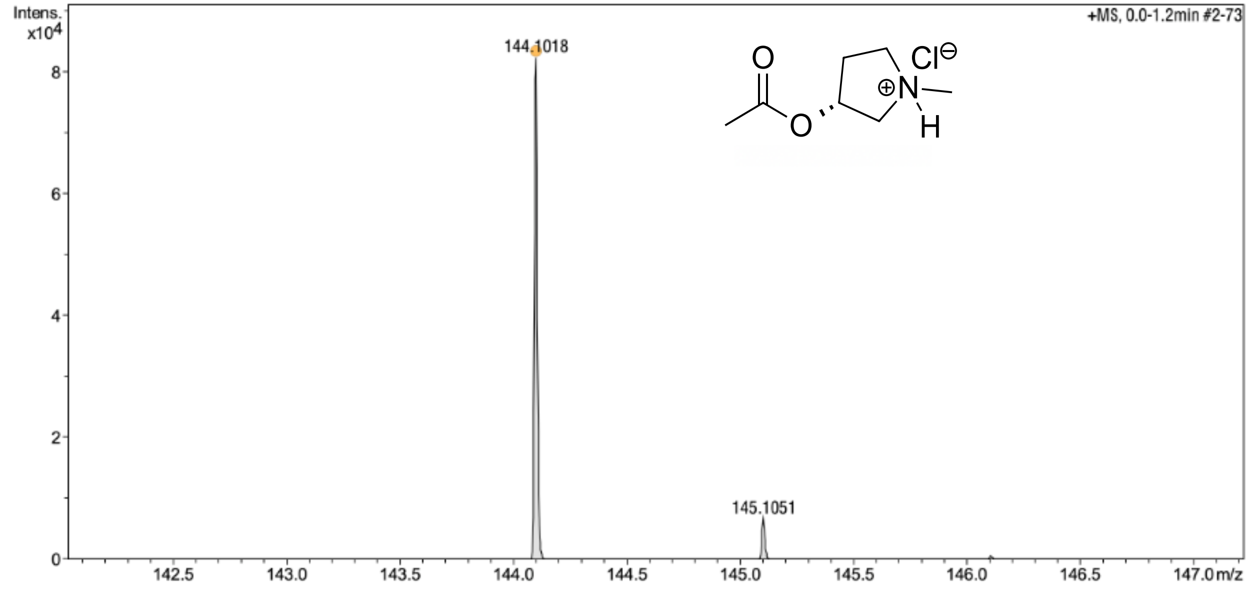

July 06 2022000010.d

Bruker Compass DataAnalysis 4.3 printed: 7/6/2022 2:57:11 PM by: x Page 1 of 1

(18) *(3R)*-3-Acetoxy-1-methyl-1-pyrrolidinium Chloride HPLC. Note: Noise from the injection has a retention time from 1.6 min until 2.5 min. The compound of interest has a retention time of 3.097 min.

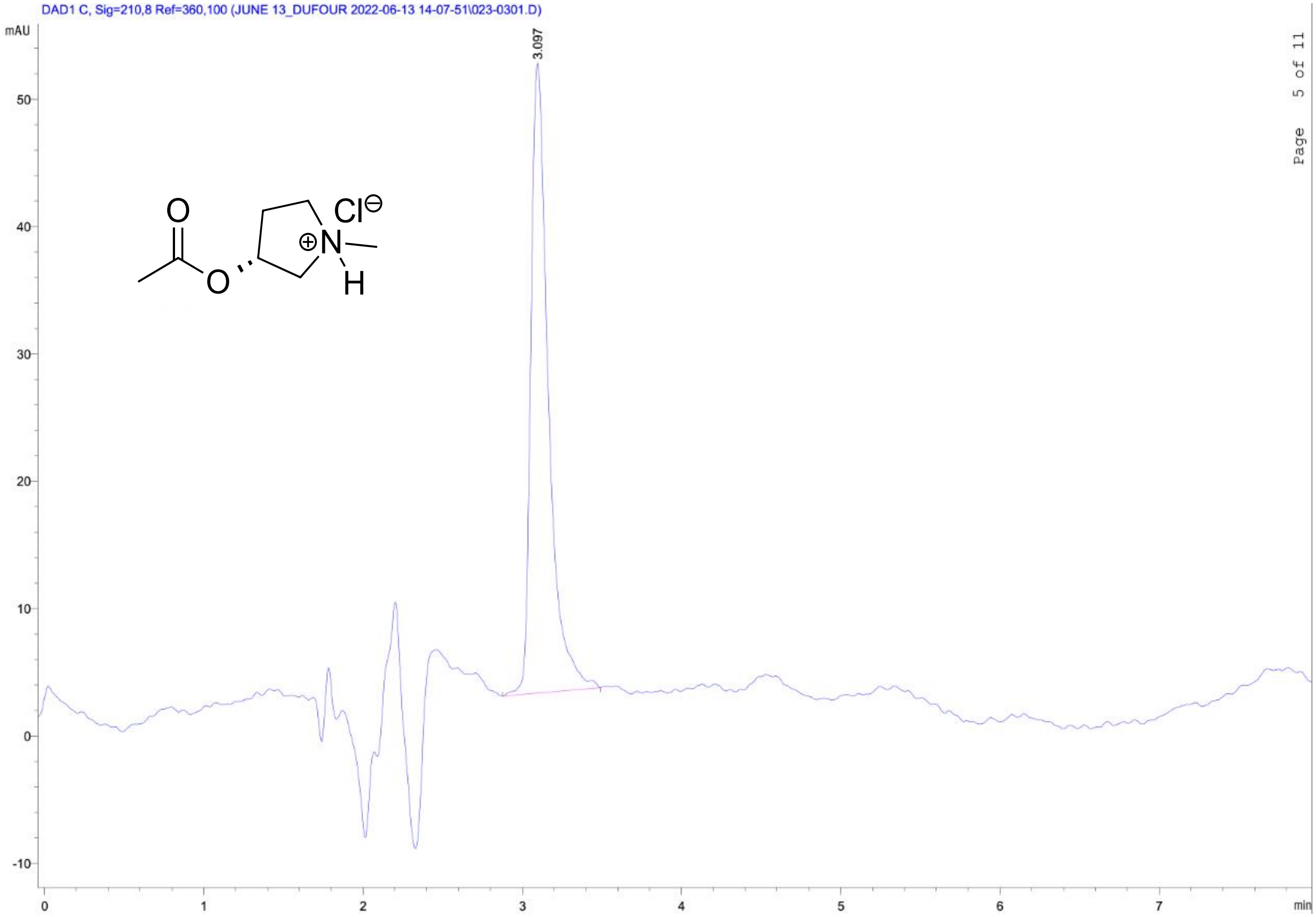

(19) (*(3S)*-1-Methylpyrrolidin-3-yl) Butanethioate

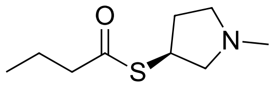

Synthesis: **(*3R*)- 3-(Mesyloxy)-1-methylpyrrolidine**: (*3R*)-(-)-1-Methyl-3-pyrrolidinol (6.0 mL, 54.6 mmol) was dissolved in anhydrous dichloromethane (180 mL) and cooled to 0°C with an ice bath. Triethylamine (11.4 mL, 81.9 mmol) and methanesulfonyl chloride (4.9 mL, 62.8 mmol) were added dropwise at 0°C. The ice bath was removed after 5 min, and the reaction was stirred overnight (16 hrs). The reaction was quenched with brine (50 mL), extracted with dichloromethane (3 × 15 mL) and dried over Na_2SO_3. The solvent was removed *in vacuo* to produce (*3R*)-3-(mesyloxy)-1-methylpyrrolidine as a yellow oil (6.11 g, 54%). Product confirmed by ^{1}H NMR and immediately carried through to next step.

Spectroscopic data: ^{1}H NMR (400 MHz, $CDCl_3$) δ 5.23-5.19 (m, 1H), 3.01 (s, 3H), 2.90 (dd, J = 11.6 Hz, 2.0 Hz, 1H), 2.87-2.83 (m, 1H), 2.71 (dd, J = 11.3, 5.7 Hz, 1H), 2.37 (s, 3H), 2.35-2.28 (m, 2H), 2.14-2.05 (m, 1H).

(*3S*)-S-(1-Methylpyrrolidin-3-yl) ethanethioate: (*3R*)-3-(Mesyloxy)-1-methylpyrrolidine (6.11g, 34.11 mmol), was dissolved in anhydrous THF (114 mL) under an argon atmosphere. 18-Crown-6-ether (9.91 g, 37.5 mmol) was added followed by potassium thioacetate (4.29 g, 37.5 mmol), added in 3 portions. Once all potassium thioacetate had gone into solution, the reaction was heated to 30°C and stirred overnight. The reaction was gravity filtered and the filtrate was diluted with water (50 mL). The layers were separated, and the aqueous layer was extracted with ethyl acetate (3 × 15 mL). The combined organic layers were washed with water (3 × 20 mL) and brine (3 × 20 mL), dried over Na_2SO_3, gravity filtered, and the filtrate was concentrated *in vacuo*

to produce a red oil. This crude material was distilled under vacuum (1 mm Hg at 95 °C) to give (*3S*)-S-(1-methylpyrrolidin-3-yl) ethanethioate as a yellow oil (2.33 g, 37%). Product confirmed by ^{1}H NMR and immediately carried through to next step.

Spectroscopic data: ^{1}H NMR (400 MHz, $CDCl_3$) δ 3.95-3.89 (m, 1H), 2.94 (dd, J = 10.2 Hz, 7.3 Hz, 1H), 2.76-2.71 (m, 1H), 2.56 (dd, J = 10.4, 4.8 Hz, 1H), 2.52-2.46 (m, 1H), 2.40-2.35 (m, 1H), 2.38 (s, 3H).

(*3S*)-1-Methyl-3-pyrrolidinethiol: (*3S*)-S-(1-Methylpyrrolidin-3-yl) ethanethioate (0.952 g, 5.98 mmol) was dissolved in MeOH (30 mL) and sodium methoxide (0.333 g, 5.98 mmol) was added to the stirring solution. After (*3S*)-S-(1-methylpyrrolidin-3-yl) ethanethioate was no longer visible by thin layer chromatography developed by a $KMnO_4$ stain, the reaction was concentrated *in vacuo.* The resulting material was taken up in dichloromethane and concentrated *in vacuo*, and this process was repeated twice.

Product confirmed by ^{1}H NMR and immediately carried through to next step.

Spectroscopic data: ^{1}H NMR (400 MHz, $CDCl_3$) δ 3.40-3.35 (m, 1H), 2.79-2.71 (m, 2H), 2.52-2.48 (m, 1H), 2.33-2.36 (m, 3H), 2.31 (s, 3H), 1.72-1.63 (m, 1H).

(*(3S)*-1-Methylpyrrolidin-3-yl) butanethioate: The entire material of (*3S*)-1-methyl-3-pyrrolidinethiol from the previous step was dissolved in dichloromethane (20 mL) and the resulting solution was cooled to 0°C in an ice bath. Butyryl chloride (0.621 mL, 5.98 mmol) was added followed by triethylamine (1.67 mL, 12.0 mmol). The ice bath was removed after 30 mins and the reaction was stirred overnight for 16 hrs. The reaction was concentrated *in vacuo* to afford a white material. This crude material was placed in diethyl ether (20 mL), gravity filtered to remove the insoluble material, and the filtrate was concentrated *in vacuo* to give (*(3S)*-1-methylpyrrolidin-3-yl) butanethioate as a clear oil (0.786 g, 70%).

Spectroscopic data: IR(ATR) 2963, 2938, 2774, 1684, 1116, 988, 883 cm^{-1}; ^{1}H NMR (400 MHz, $CDCl_3$) δ 3.96-3.90 (m, 1H), 2.94 (dd, J = 10.2, 7.3 Hz, 1H), 2.74-2.68 (m, 1H), 2.54 (dd, J = 10.12, 5.2 Hz, 1H), 2.47 (t, J = 7.5 Hz, 2H), 2.38-2.36 (m, 2H), 2.36 (s, 3H), 1.80-1.72 (m, 1H), 1.65 (sextet, J = 7.4 Hz, 2H), 0.94 (t, J = 7.4 Hz, 3H); ^{13}C NMR (100.7 MHz, DMSO-*d6*) δ 200.1 (0), 62.8 (2), 55.5 (2), 45.8 (2), 42.0 (2), 40.0 (1), 32.2 (2), 19.2 (2), 13.6 (3); HRMS (ESI^+): calculated for $C_9H_{18}NOS^+$: 188.1104 amu; found for $C_9H_{18}NOS^+$: 188.1100 amu; HPLC purity at 230 nm (75% CH_3CN : 10% CH_3OH : 15% aqueous triethylamine [0.1% triethylamine in H_2O], retention time 4.506 mins): 89%.

(19) ((*3S*)-1-Methylpyrrolidin-3-yl) Butanethioate IR(ATR)

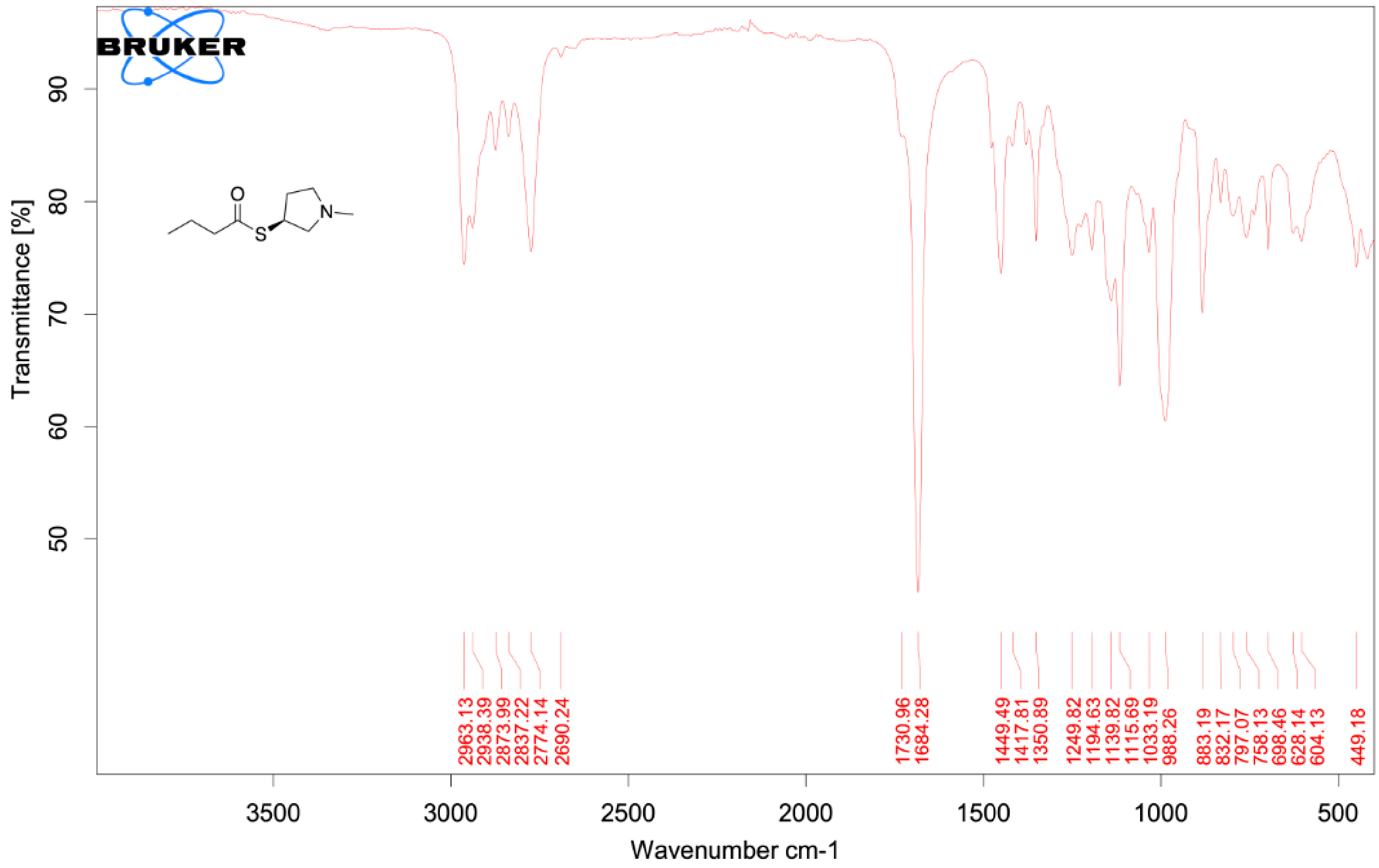

(19) ((*3S*)-1-Methylpyrrolidin-3-yl) Butanethioate ^{1}H NMR 400 MHz ($CDCl_3$)

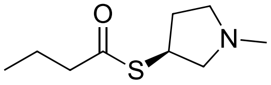

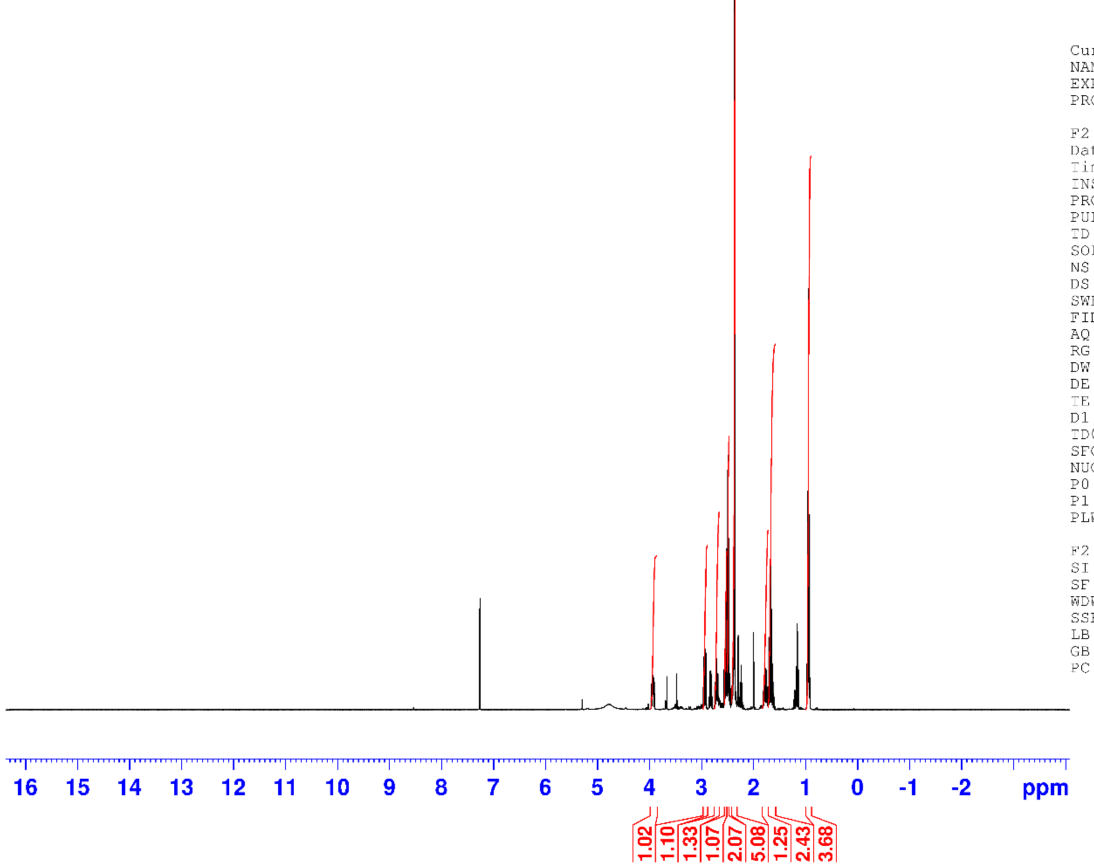

Current Data Parameters

NAME	MD-2-95
EXPNO	1
PROCNO	1

F2 - Acquisition Parameters

Date_	20220929
Time	9.59 h
INSTRUM	AVN 400
PROBHD	Z163739_0038 (
PULPROG	zg30
TD	65536
SOLVENT	CDCl3
NS	16
DS	2
SWH	8196.722 Hz
FIDRES	0.250144 Hz
AQ	3.9976959 sec
RG	101
DW	61.000 usec
DE	13.55 usec
TE	298.0 K
D1	1.00000000 sec
TD0	1
SFO1	400.3024719 MHz
NUC1	1H
P0	3.33 usec
P1	9.98 usec
PLW1	14.13000011 W

F2 - Processing parameters

SI	65536
SF	400.3000101 MHz
WDW	EM
SSB	0
LB	0.30 Hz
GB	0
PC	1.00

(19) ((*3S)*-1-Methylpyrrolidin-3-yl) Butanethioate ^{13}C NMR 100 MHz ($CDCl_3$)

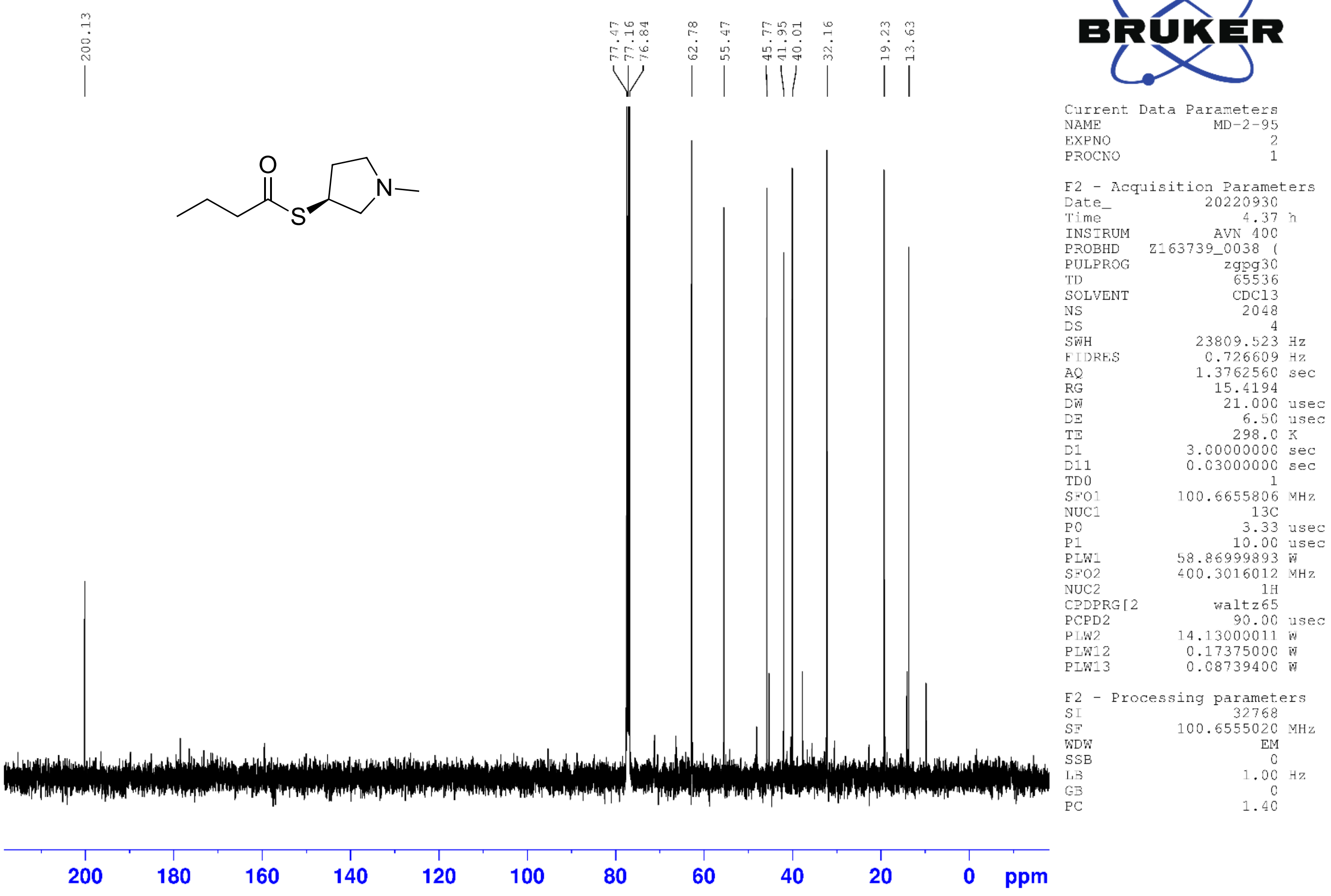

(19) ((*3S*)-1-Methylpyrrolidin-3-yl) Butanethioate High Resolution Mass Spectrum (ESI, positive mode)

Mass Spectrum SmartFormula Report

Analysis Info

Analysis Name	D:\Data\Xiao\Oct 14 2022000006.d	Acquisition Date	2022-10-14 11:10:14 AM
Method	Xiao all 1.m	Operator	x
Sample Name	TRV 6023	Instrument	compact 8255754.20059
Comment			

Acquisition Parameter

Source Type	ESI	Ion Polarity	Positive	Set Nebulizer	0.5 Bar
Focus	Not active	Set Capillary	3500 V	Set Dry Heater	181 °C
Scan Begin	50 m/z	Set End Plate Offset	-500 V	Set Dry Gas	4.0 l/min
Scan End	1500 m/z	Set Charging Voltage	2000 V	Set Divert Valve	Source
		Set Corona	0 nA	Set APCI Heater	0 °C

Meas. m/z	Ion Formula	m/z	err [ppm]
188.1100	C9H18NOS	188.1104	1.8

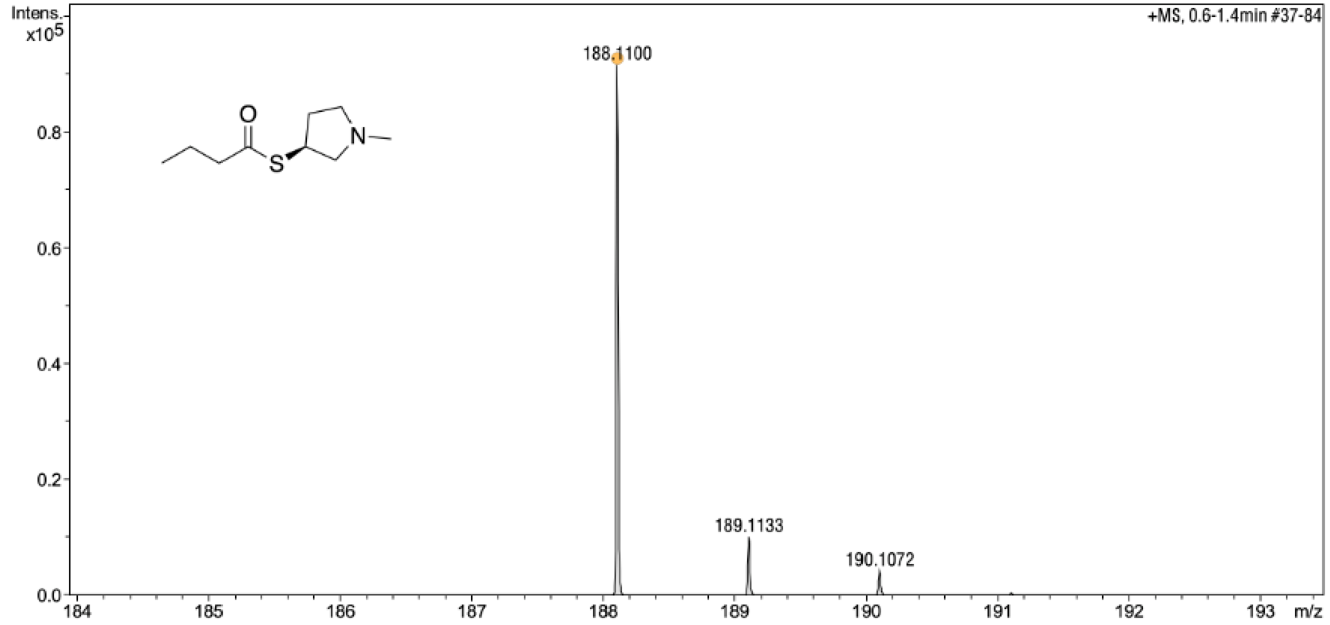

Oct 14 2022000006.d

Bruker Compass DataAnalysis 4.3 printed: 2022-10-14 11:13:15 AM by: x Page 1 of 1

(19) ((*3S*)-1-Methylpyrrolidin-3-yl) Butanethioate HPLC

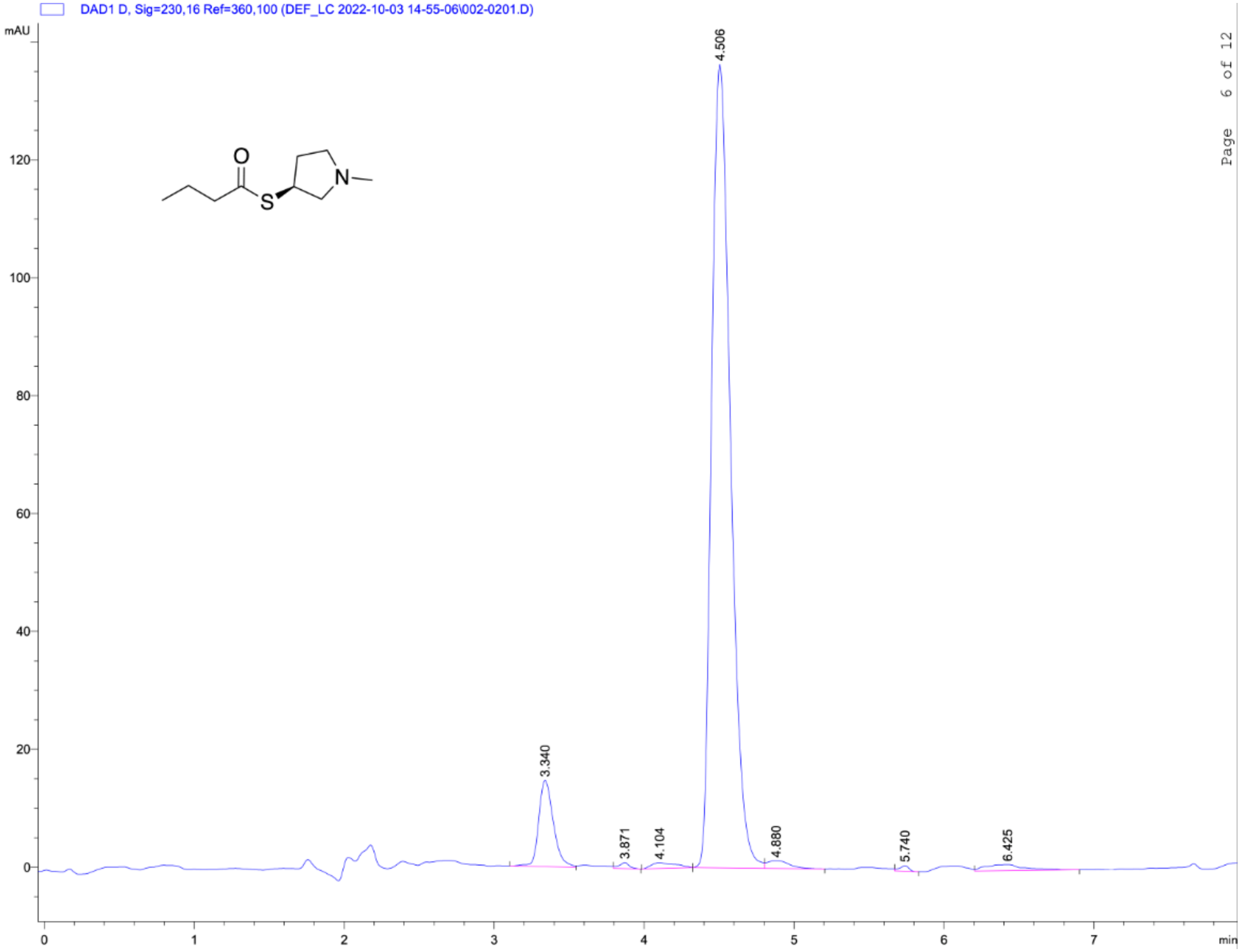

(20) *(3S)*-3-Butyroxy-1-methyl-1-pyrrolidinium Chloride

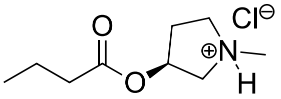

Synthesis: **(*3S*)-1-Methyl-3-pyrrolidinyl butyrate**: (*3S*)-(+)-1-Methyl-3-pyrrolidinol (0.329 mL, 3 mmol) and triethylamine (1.05 mL, 7.5 mmol) were dissolved in anhydrous dichloromethane (10 mL) and cooled to 0°C with an ice bath. Butyryl chloride (0.312 mL, 3.00 mmol) was added dropwise at 0°C. The ice bath was removed after 5 min, and the reaction was stirred for 3hrs. The reaction was quenched with brine (10 mL), extracted with dichloromethane (3 × 15 mL) and dried over Na_2SO_3. The solvent was removed *in vacuo* to produce a yellow oil. The crude mixture was purified using flash chromatography through silica gel column (5% MeOH/DCM) to afford (*3S*)-1-methyl-3-pyrrolidinyl butyrate as a yellow viscous liquid (0.367 g, 71%). The analytical data was similar to previously published data.[4]

***(3S)*-3-Butyroxy-1-methyl-1- pyrrolidinium chloride**: To a flask charged with (*3S*)-1-methyl-3-pyrrolidinyl butyrate (0.367 g, 1.77 mmol) anhydrous diethyl ether was added until complete dissolution (35 mL) under an argon atmosphere. HCl in diethyl ether 1 N (2.65 mL, 2.65 mmol) was added dropwise and stirred for 1hr. The solvent was removed in vacuo resulting in a white solid. The compound was triturated three times with diethyl ether (20 mL), solvent was removed with a pipet. This process was repeated two more times. The resulting white solid was dried *in vacuo* to produce *(3S)*-3-butyroxy-1-methyl-1- pyrrolidinium chloride (0.303 g, 82%).

Spectroscopic data: IR(ATR) 3400, 2964, 2877, 2663, 2606, 2576, 2473, 1731, 1167, 1078 cm^{-1}; ^{1}H NMR (400 MHz, DMSO-*d6*) δ 11.45 (br s, 1H), 5.27-5.23 (m, 1H), 3.85-3.03 (br app m, 4H), 2.79 (s, 3H), 2.38-2.34 (m, 1H), 2.30 (t, J = 7.4 Hz, 2H), 2.03-2.00 (m, 1H), 1.55 (sext, J = 7.4

Hz, 2H), 0.88 (t, J = 7.4 Hz, 3H); ^{13}C NMR (100.7 MHz, DMSO-*d6*) δ 172.4 (0), 72.1 (1), 59.0 (2), 53.1 (2), 40.6 (3), 35.2 (2), 30.3 (2), 17.7 (2), 13.4 (3); LRMS (ESI$^+$): 158.1 (M$^+$); HRMS (ESI$^+$): calculated for $C_9H_{18}NO_2^+$: 172.1332 amu; found for $C_9H_{18}NO_2^+$: 172.1336 amu; HPLC purity at 210 nm (75% CH_3CN : 10% CH_3OH : 15% aqueous triethylamine [0.1% triethylamine in H_2O], retention time 3.786 mins): >99%.

(20) *(3S)*-3-Butyroxy-1-methyl-1-pyrrolidinium Chloride IR(ATR)

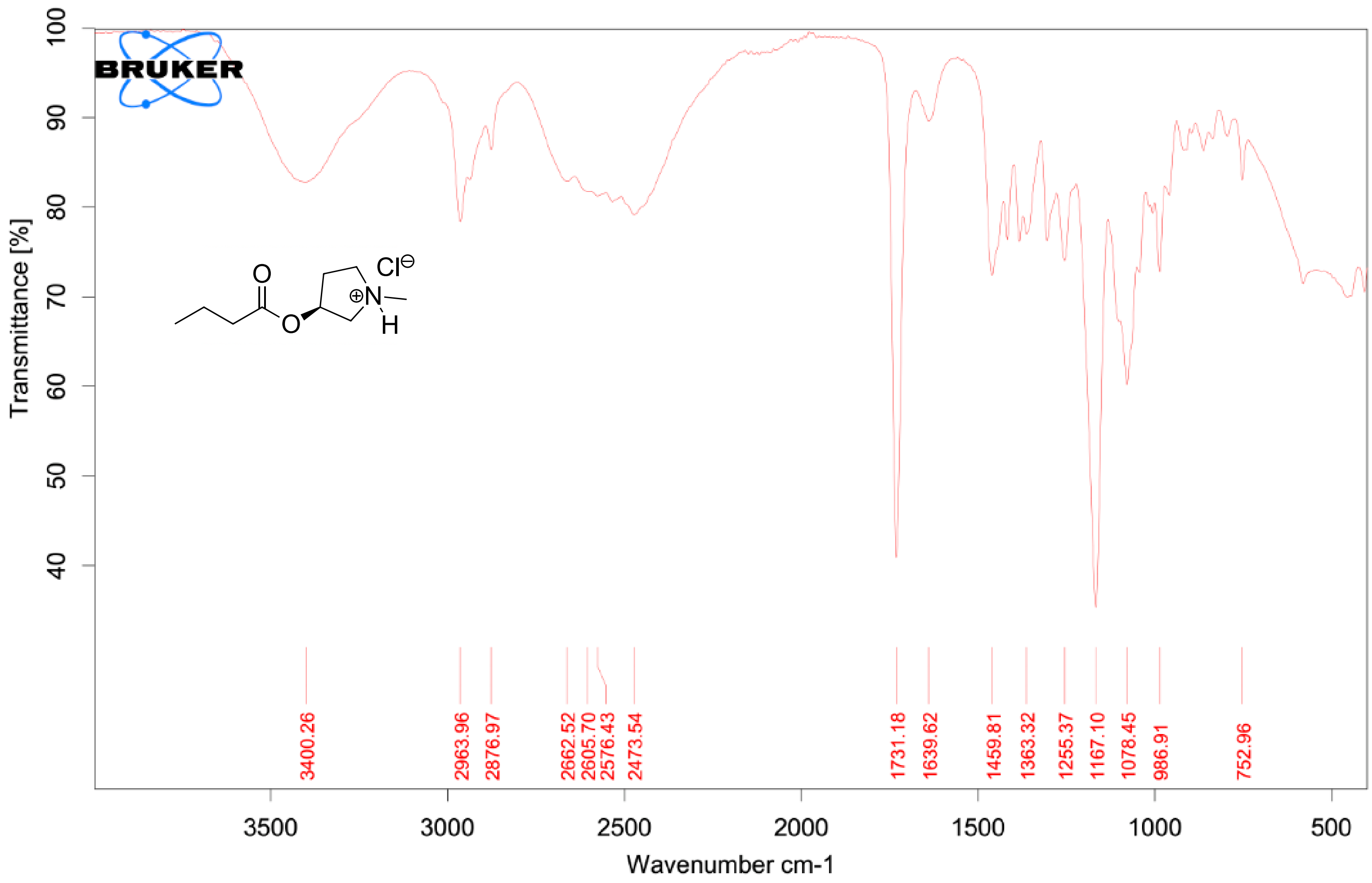

(20) *(3S)*-3-Butyroxy-1-methyl-1-pyrrolidinium Chloride ^{1}H NMR 400 MHz (DMSO-*d6*)

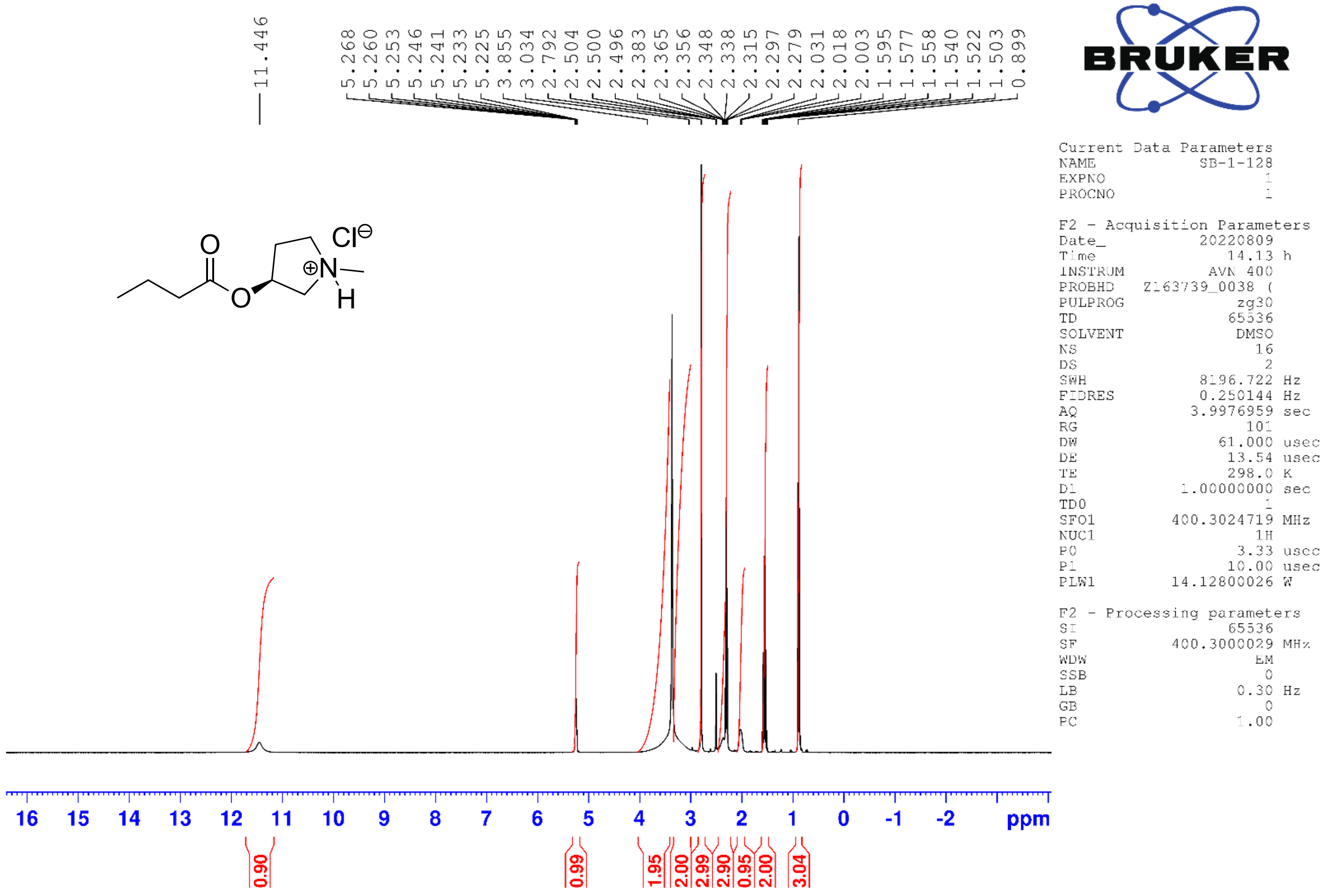

(20) *(3S)*-3-Butyroxy-1-methyl-1-pyrrolidinium Chloride ^{13}C NMR 100 MHz (DMSO-*d6*)

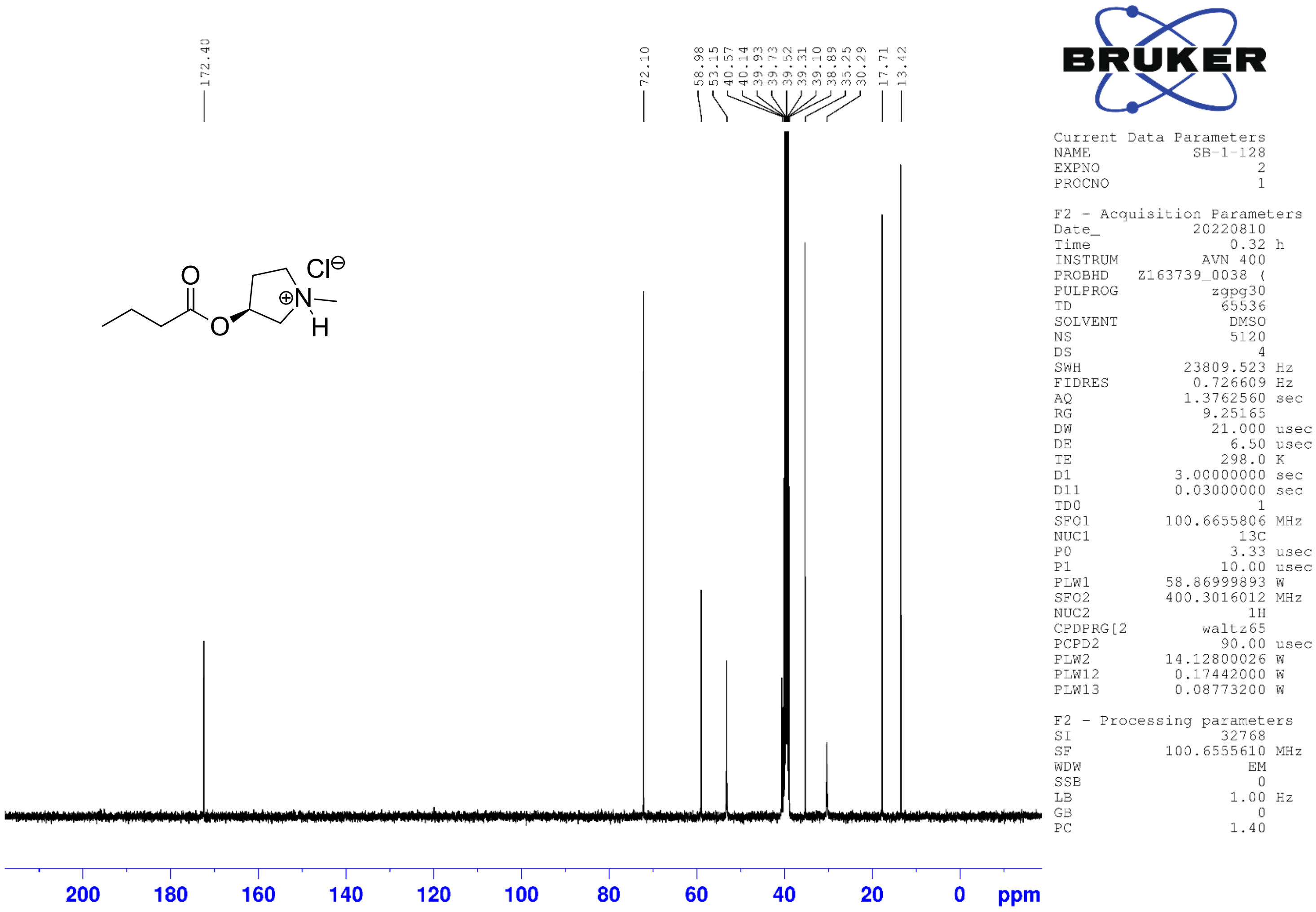

(20) *(3S)*-3-Butyroxy-1-methyl-1-pyrrolidinium Chloride High Resolution Mass Spectrum (ESI, positive mode)

Mass Spectrum SmartFormula Report

Analysis Info

Analysis Name	D:\Data\Xiao\July 07 2022000010.d	Acquisition Date	7/7/2022 9:46:34 AM	
Method	Xiao all 1.m	Operator	x	
Sample Name	TRV 6015 HCl	Instrument	compact	8255754.20059
Comment				

Acquisition Parameter

Source Type	ESI	Ion Polarity	Positive	Set Nebulizer	0.5 Bar
Focus	Not active	Set Capillary	3500 V	Set Dry Heater	180 °C
Scan Begin	50 m/z	Set End Plate Offset	-500 V	Set Dry Gas	4.0 l/min
Scan End	1500 m/z	Set Charging Voltage	2000 V	Set Divert Valve	Source
		Set Corona	0 nA	Set APCI Heater	0 °C

Meas. m/z	Ion Formula	m/z	err [ppm]
172.1336	C9H18NO2	172.1332	-2.2

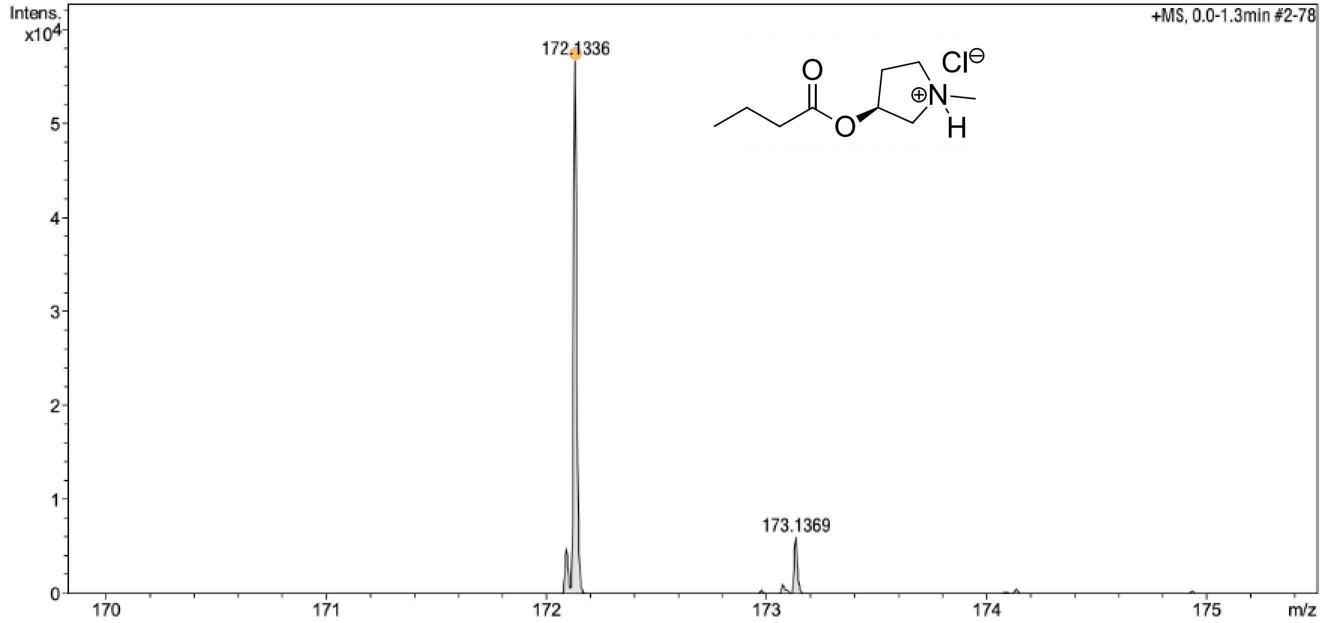

July 07 2022000010.d

Bruker Compass DataAnalysis 4.3 printed: 7/7/2022 9:49:58 AM by: x Page 1 of 1

(20) *(3S)*-3-Butyroxy-1-methyl-1-pyrrolidinium Chloride HPLC. Note: Noise from the injection has a retention time from 1.6 min until 2.5 min. The compound of interest has a retention time of 3.786 mins.

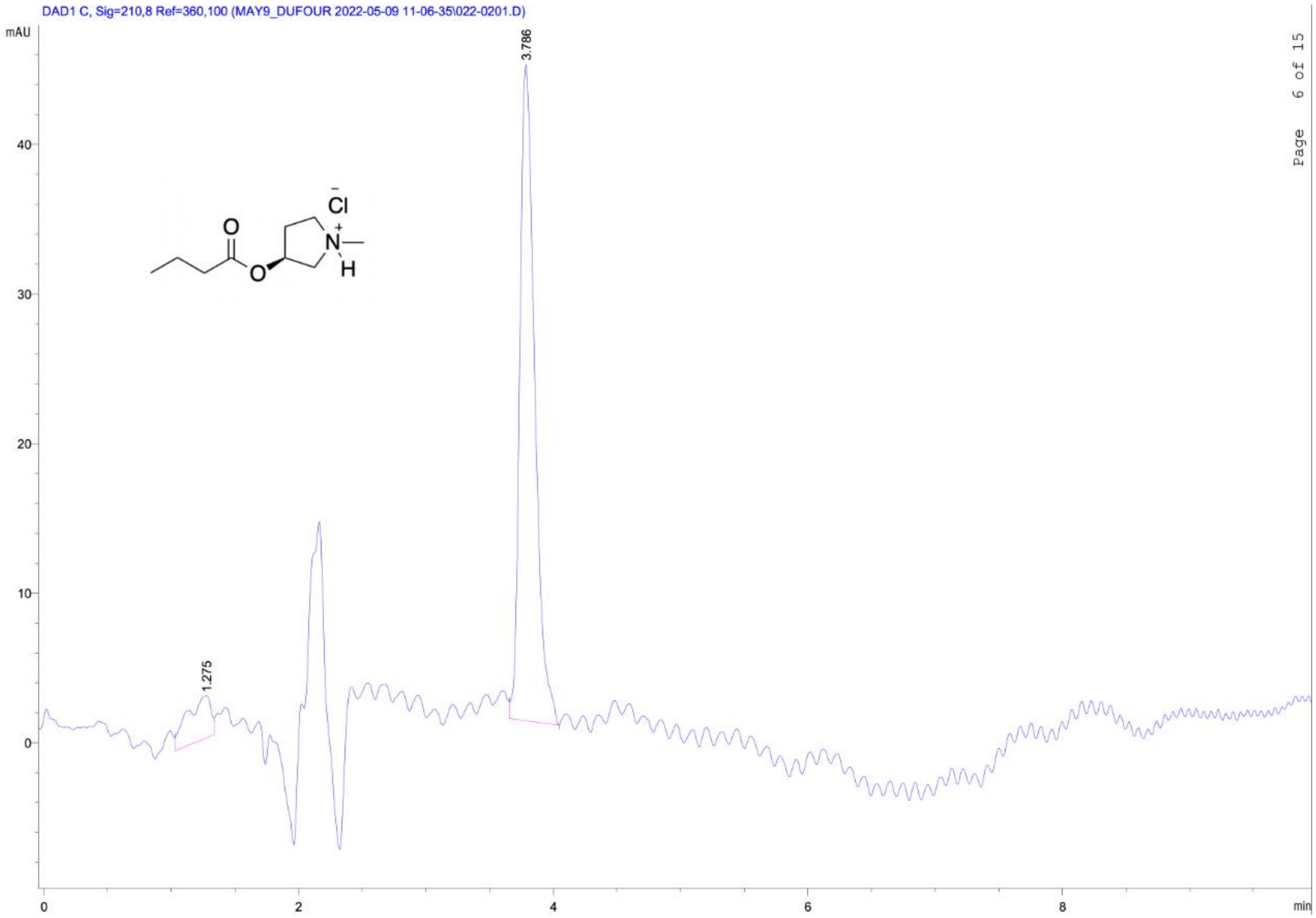

(21) ((*3R*)-1-Methylpyrrolidin-3-yl) Butanethioate

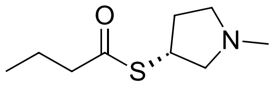

Synthesis: **((*3R*)-1-Methylpyrrolidin-3-yl) butanethioate**: (*3R*)-*S*-(1-Methylpyrrolidin-3-yl) ethanethioate (1.08 g, 6.76 mmol) was dissolved in MeOH (30 mL) and cooled in an ice bath. Sodium methoxide (0.438 g, 8.11 mmol) was added in one portion and the reaction was stirred for 3 hrs. The reaction was concentrated *in vacuo* to ensure all methanol was removed. Anhydrous THF (25 mL) and Et_3N (2.80 mL, 20.3 mmol) was added to this mixture. The stirring solution was cooled to 0^oC using an ice bath. Butyryl chloride (1.05 mL, 10.1 mmol) was added dropwise, and a white precipitate formed immediately. The reaction was warmed to room temperature and stirred for 1.5 hrs. The reaction was diluted with diethyl ether (30 mL) washed with water (2 x 30 mL) and brine (30 mL). The organic layer was dried over Na_2SO_4, gravity filtered, and the filtrate was concentrated *in vacuo*. The crude material was subjected to flash chromatography through silica gel (9% methanol in dichloromethane) to afford ((*3R*)-1-methylpyrrolidin-3-yl) butanethioate (69%, 0.861 g, 4.59 mmol) of as a clear light red liquid.

Spectroscopic data: IR(ATR) 2963, 2938, 2773, 1683, 1449, 1116, 985, 884 cm^{-1}; ^{1}H NMR (400 MHz, $CDCl_3$) δ 3.96-3.89 (m, 1H), 2.89 (dd, J = 10.1, 7.2 Hz, 1H), 2.72-2.67 (m, 1H), 2.53-2.47 (m, 1H), 2.49 (t, J = 7.4 Hz, 2H), 2.45-2.37 (m, 2H), 2.36 (s, 3H), 1.79-1.72 (m, 1H), 1.68 (sext, J = 7.4 Hz, 2H), 0.94 (t, J = 7.4 Hz, 3H); ^{13}C NMR (100.7 MHz, $CDCl_3$) δ 200.2 (0), 63.1 (2), 55.6 (2), 45.8 (2), 42.1 (3), 40.1 (1), 32.2 (2), 19.2 (2), 13.6 (3); LRMS (ESI^+): XXX (M^+); HRMS (ESI^+): calculated for $C_9H_{18}NOS^+$: 188.1104 amu; found for $C_9H_{18}NOS^+$: 188.1106 amu; HPLC purity at 230 nm (25% CH_3CN : 75% CH_3OH, retention time: 3.323 mins): 98%.

(21) ((*3R*)-1-Methylpyrrolidin-3-yl) Butanethioate IR(ATR)

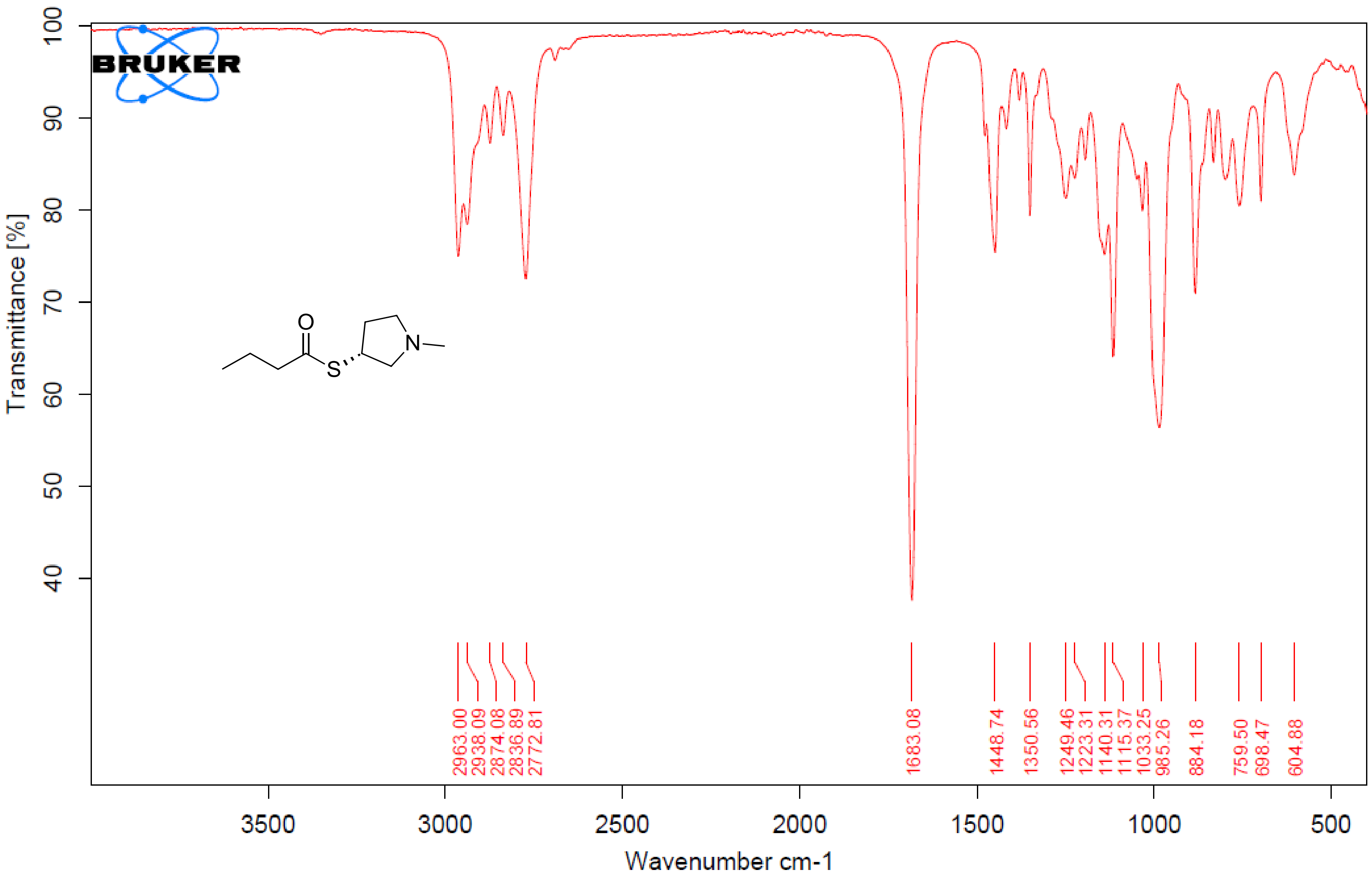

(21) ((*3R*)-1-Methylpyrrolidin-3-yl) Butanethioate ^{1}H NMR 400 MHz ($CDCl_3$)

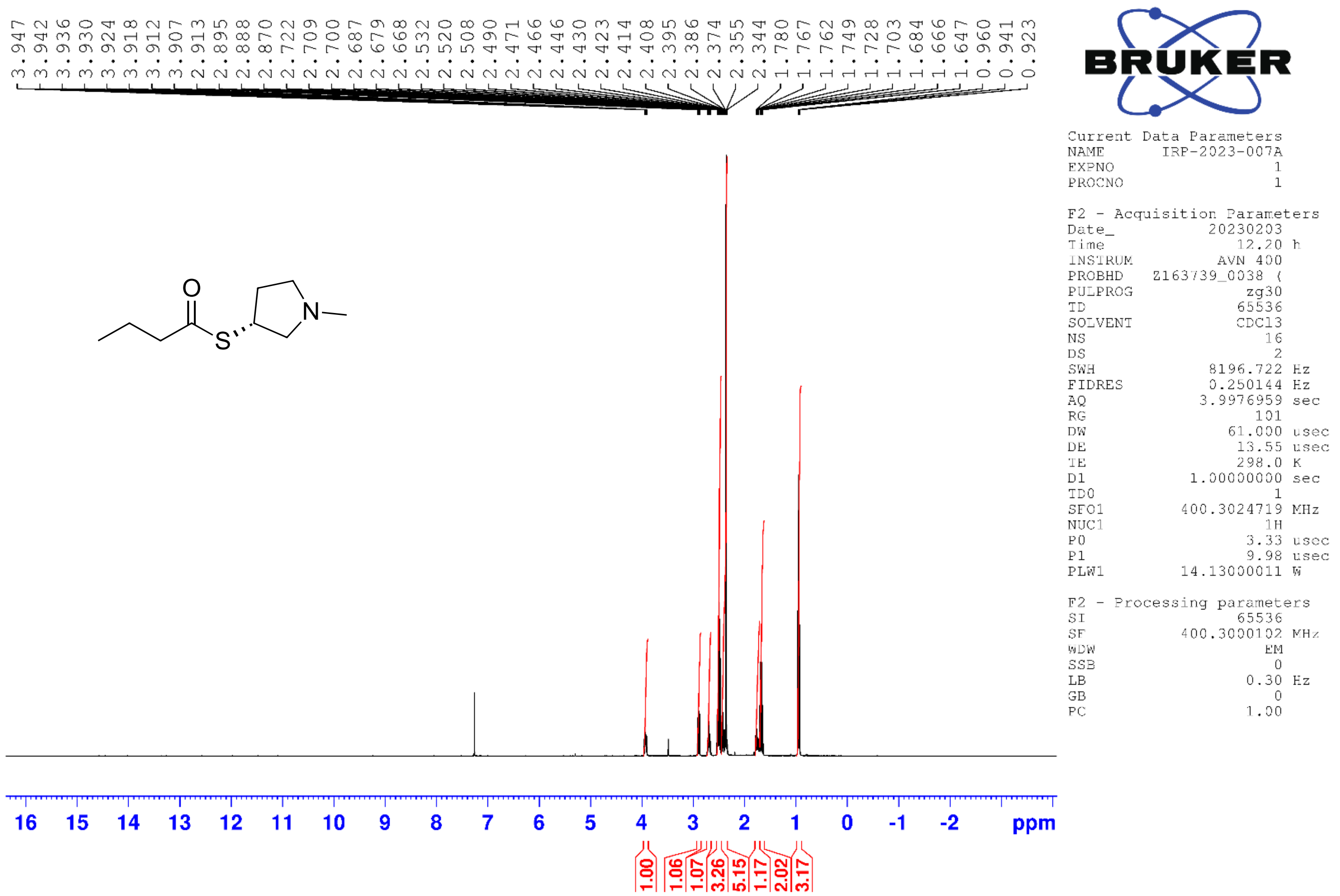

(21) ((*3R*)-1-Methylpyrrolidin-3-yl) Butanethioate ^{13}C NMR 100MHz ($CDCl_3$)

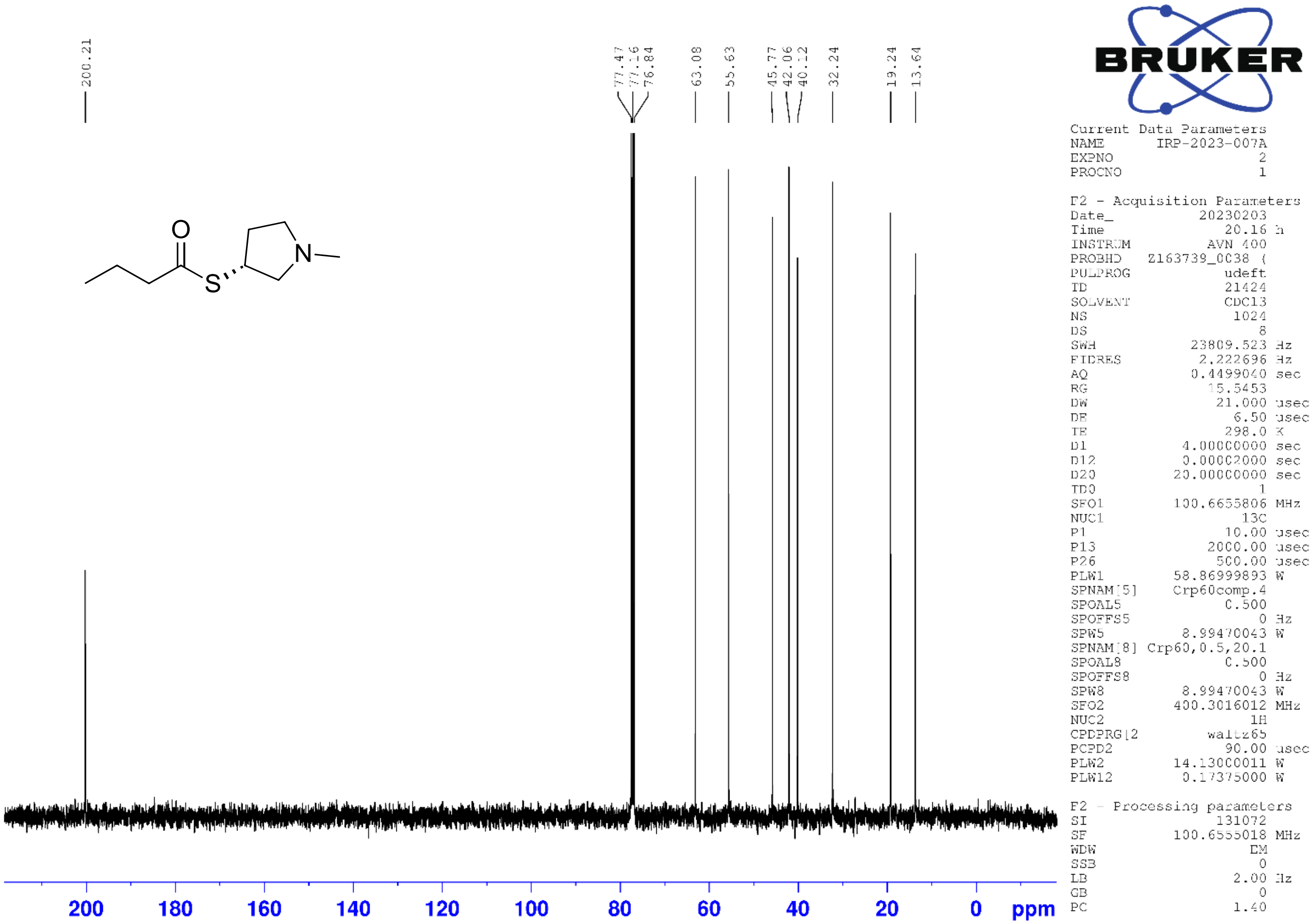

(21) ((*3R*)-1-Methylpyrrolidin-3-yl) Butanethioate High Resolution Mass Spectrum (ESI, positive mode)

Mass Spectrum SmartFormula Report

Analysis Info

Analysis Name	D:\Data\Xiao\Feb 17 2023000007.d	Acquisition Date	2023-02-17 9:28:19 AM
Method	Xiao all 1.m	Operator	x
Sample Name	TRV6024	Instrument	compact 8255754.20059
Comment			

Acquisition Parameter

Source Type	ESI	Ion Polarity	Positive	Set Nebulizer	0.5 Bar
Focus	Not active	Set Capillary	3500 V	Set Dry Heater	180 °C
Scan Begin	50 m/z	Set End Plate Offset	-500 V	Set Dry Gas	4.0 l/min
Scan End	1500 m/z	Set Charging Voltage	2000 V	Set Divert Valve	Source
		Set Corona	0 nA	Set APCI Heater	0 °C

Meas. m/z	Ion Formula	m/z	err [ppm]
188.1106	C9H18NOS	188.1104	-1.3

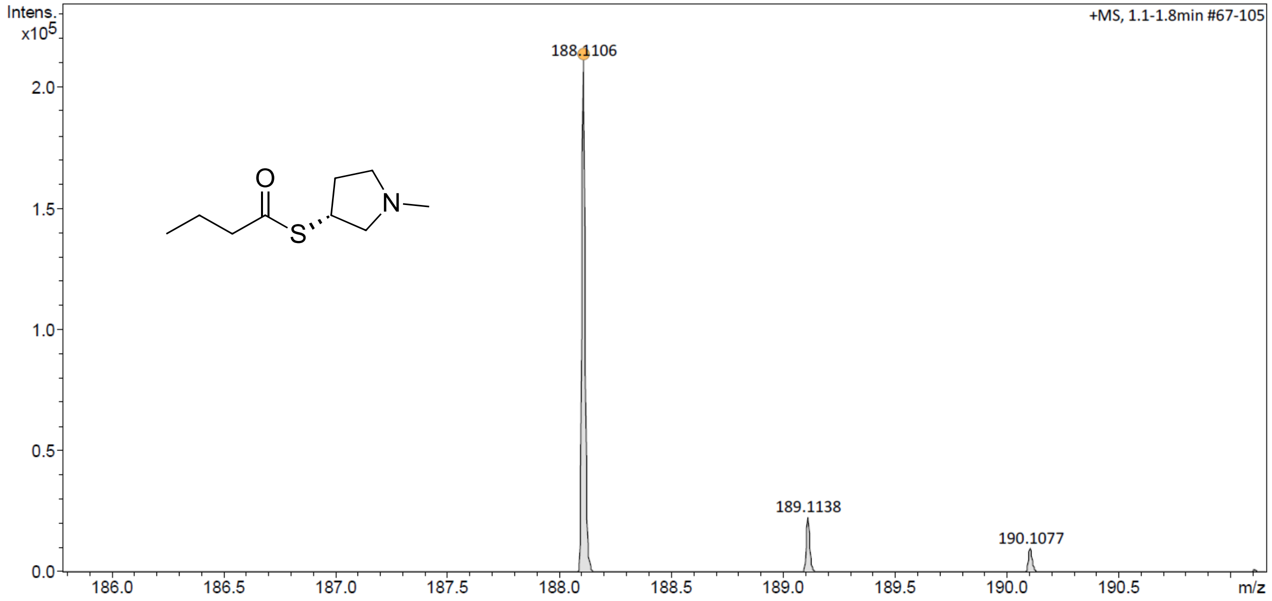

Feb 17 2023000007.d

Bruker Compass DataAnalysis 4.3 printed: 2023-02-17 9:46:43 AM by: x Page 1 of 1

(21) ((*3R*)-1-Methylpyrrolidin-3-yl) Butanethioate HPLC

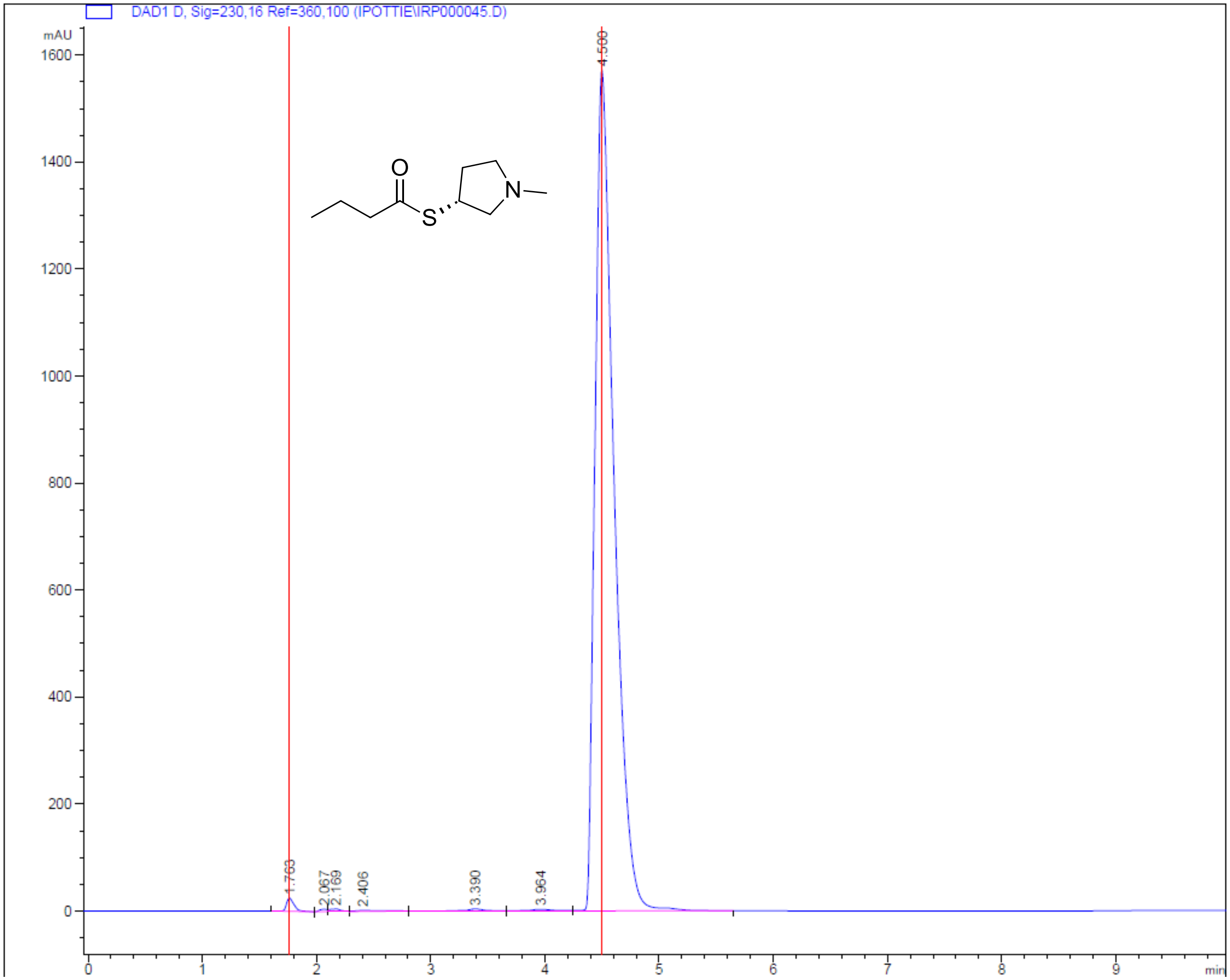

(22) *(3R)*-3-Butyroxy-1-methyl-1-pyrrolidinium Chloride

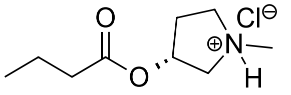

Synthesis: **(*3R*)-1-methyl-3-pyrrolidinyl butyrate**: (*3R*)-(-)-1-Methyl-3-pyrrolidinol (0.549 mL, 5.00 mmol) and triethylamine (1.74 mL, 12.5 mmol) were dissolved in anhydrous dichloromethane (17 mL) and cooled to 0°C with an ice bath. Butyryl chloride (0.519 mL, 5.00 mmol) was added dropwise at 0 °C. The ice bath was removed after 5 mins, and the reaction was stirred for 3hrs. The reaction was quenched with brine (20 mL), extracted with dichloromethane (3 × 15 mL) and dried over Na_2SO_3. The drying agent was removed was removed *via* gravity filtration and the solvent was removed *in vacuo* to produce a yellow oil. The crude mixture was purified by silica gel column chromatography (5% MeOH/DCM) to produce (*3R*)-1-methyl-3-pyrrolidinyl butyrate (0.729 g, 85%) as a yellow viscous liquid.

Spectroscopic data: IR(ATR) 2964, 2939, 2777, 1730, 1251, 1175 cm^{-1}; ^{1}H NMR (400 MHz, $CDCl_3$) δ 5.20-5.15 (m, 1H), 2.84-2.79 (m, 1H), 2.69 (dd, J = 10.9, 2.2 Hz, 1H), 2.61 (dd, J = 10.9, 5.9 Hz, 1H), 2.35 (s, 3H), 2.28-2.25 (m, 4H), 1.88-1.79 (m, 1H), 1.63 (sext, J = 7.4 Hz, 2H), 0.93 (t, J = 7.4 Hz, 3H); ^{13}C NMR (100.7 MHz, CD_3OD-*d4*) δ 173.8 (0), 74.5 (1), 62.4 (2), 55.1 (2), 42.32 (3), 36.4 (2), 32.8 (2), 18.6 (2), 13.8 (3); LRMS (ESI^+): 172.1 (M^+); HRMS (ESI^+): calculated for $C_9H_{18}NO_2^+$: 172.1332 amu; found for $C_9H_{18}NO_2^+$: 172.1334 amu; HPLC purity at 210 nm (75% CH_3CN : 10% CH_3OH, 15 % aqueous triethylamine (0.1 % triethylamine in water) retention time: 3.803 mins): 99%.

***(3R)*-3-Butyroxy-1-methyl-1-pyrrolidinium chloride**: To a flask charged with (*3R*)-1-methyl-3-pyrrolidinyl butyrate (0.364 g, 1.75 mmol) anhydrous diethyl ether was added until complete dissolution (35 mL) under an argon atmosphere. HCl in diethyl ether (1 N - 2.63 mL, 2.63 mmol) was added dropwise and stirred for 1 hr. The solvent was removed *in vacuo* resulting in a white solid. The compound was triturated with diethyl ether (20 mL), solvent was removed with a pipet. The process was repeated two more times. The material was dried *in vacuo* to produce *(3R)*-3-butyroxy-1-methyl-1- pyrrolidinium chloride (0.350 g, 96%) as a white solid. *Spectroscopic data:* IR(ATR) 3407, 2962, 2879, 2663, 2442, 1732, 1164, 1078 cm^{-1}; ^{1}H NMR (400 MHz, D_2O) δ 5.47 (app br s, 1H), 3.62 (app br s, 3H), 3.44 (app br s, 1H), 3.00 (s, 3H), 2.59-2.49 (m, 1H), 2.40 (t, J = 7.4 Hz, 2H), 2.28-2.22 (m, 1H), 1.63 (sext, J = 7.4 Hz, 2H), 0.92 (t, J = 7.4 Hz, 3H); ^{13}C NMR (100.7 MHz, D_2O) δ 175.8 (0), 72.9 (1), 60.4 (2), 54.3 (2), 41.2 (3), 35.6 (2), 30.4 (2), 17.7 (2), 12.8 (3); LRMS (ESI^+): 172.1 (M^+); $^+$); HRMS (ESI^+): calculated for $C_9H_{18}NO_2^+$: 172.1332 amu; found for $C_9H_{18}NO_2^+$: 172.1331 amu; HPLC purity at 210 nm (75% CH_3CN : 10% CH_3OH, 15 % aqueous triethylamine (0.1 % triethylamine in water) retention time: 3.805 mins): >99%.

(22) *(3R)*-3-Butyroxy-1-methyl-1-pyrrolidinium Chloride IR(ATR)

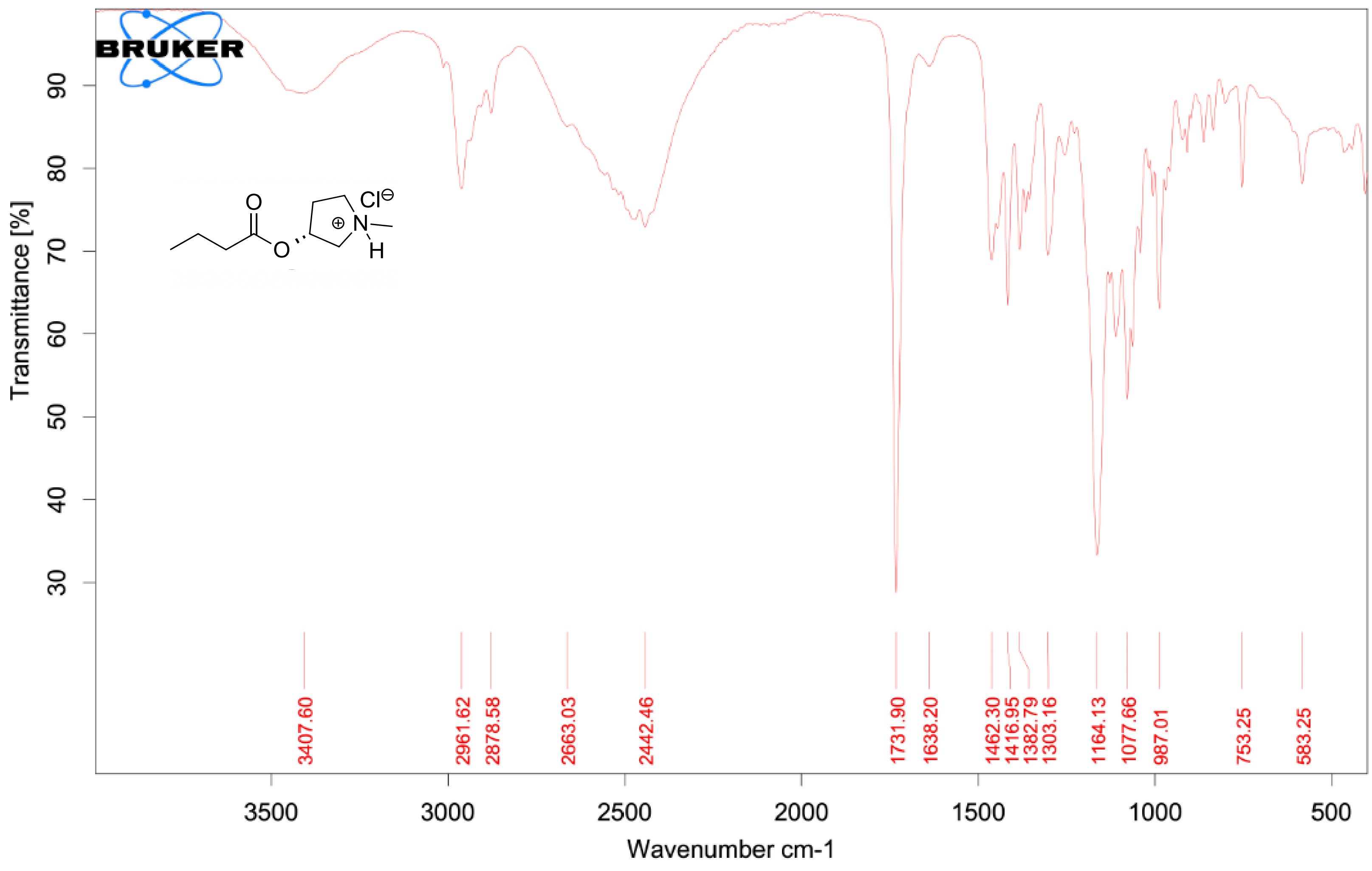

(22) *(3R)*-3-Butyroxy-1-methyl-1-pyrrolidinium Chloride ^{1}H NMR 400 MHz (D_2O)

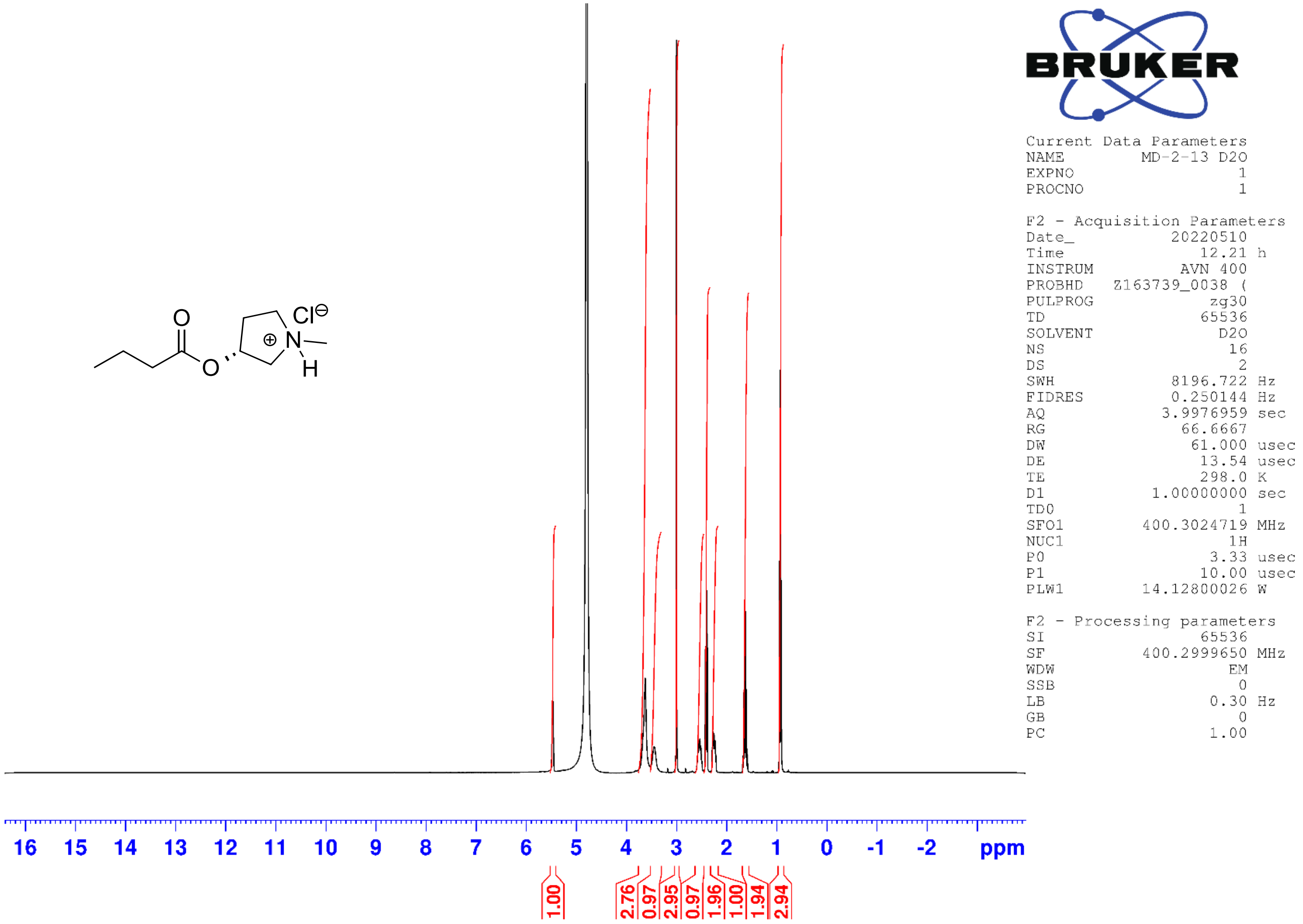

(22) *(3R)*-3-Butyroxy-1-methyl-1-pyrrolidinium Chloride ^{13}C NMR 100 MHz (D_2O)

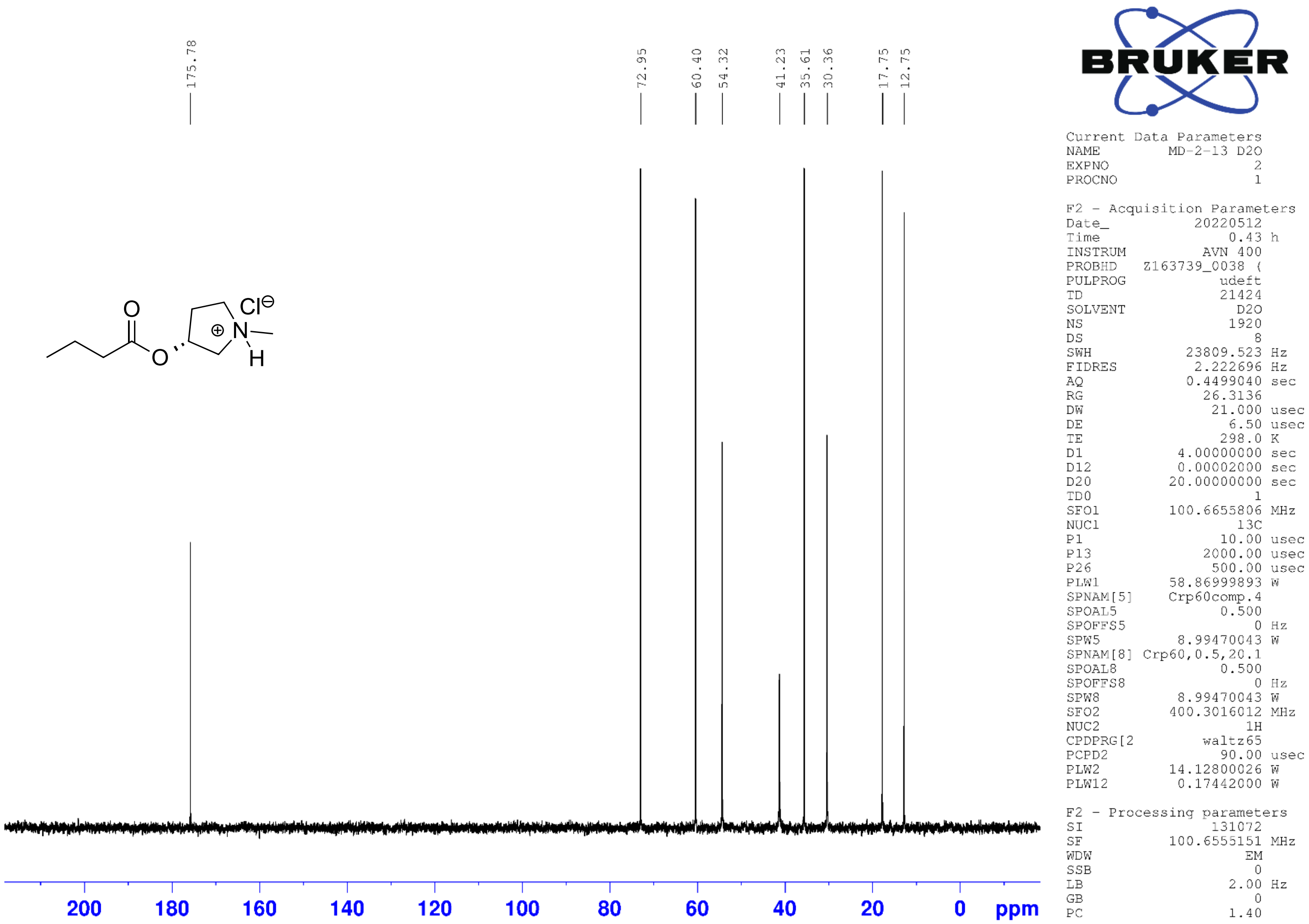

(22) *(3R)*-3-Butyroxy-1-methyl-1-pyrrolidinium Chloride High Resolution Mass Spectrum (ESI, positive mode)

Mass Spectrum SmartFormula Report

Analysis Info

Analysis Name	D:\Data\Xiao\July 07 2022000012.d	Acquisition Date	7/7/2022 9:55:17 AM	
Method	Xiao all 1.m	Operator	x	
Sample Name	TRV 6016 HCl	Instrument	compact	8255754.20059
Comment				

Acquisition Parameter

Source Type	ESI	Ion Polarity	Positive	Set Nebulizer	0.5 Bar
Focus	Not active	Set Capillary	3500 V	Set Dry Heater	180 °C
Scan Begin	50 m/z	Set End Plate Offset	-500 V	Set Dry Gas	4.0 l/min
Scan End	1500 m/z	Set Charging Voltage	2000 V	Set Divert Valve	Source
		Set Corona	0 nA	Set APCI Heater	0 °C

Meas. m/z	Ion Formula	m/z	err [ppm]
172.1331	C9H18NO2	172.1332	0.5

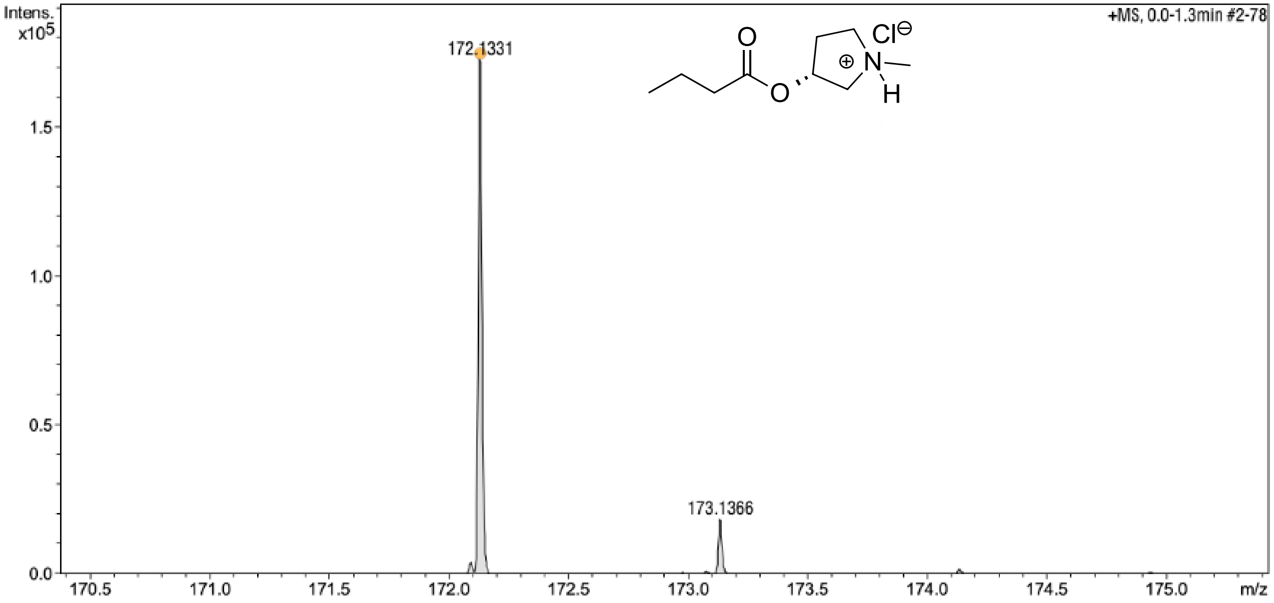

July 07 2022000012.d

Bruker Compass DataAnalysis 4.3 printed: 7/7/2022 9:58:15 AM by: x Page 1 of 1

(22) *(3R)*-3-Butyroxy-1-methyl-1-pyrrolidinium Chloride HPLC. Note: Noise from the injection has a retention time from 1.6 min until 2.5 min. The compound of interest has a retention time of 3.805 min.

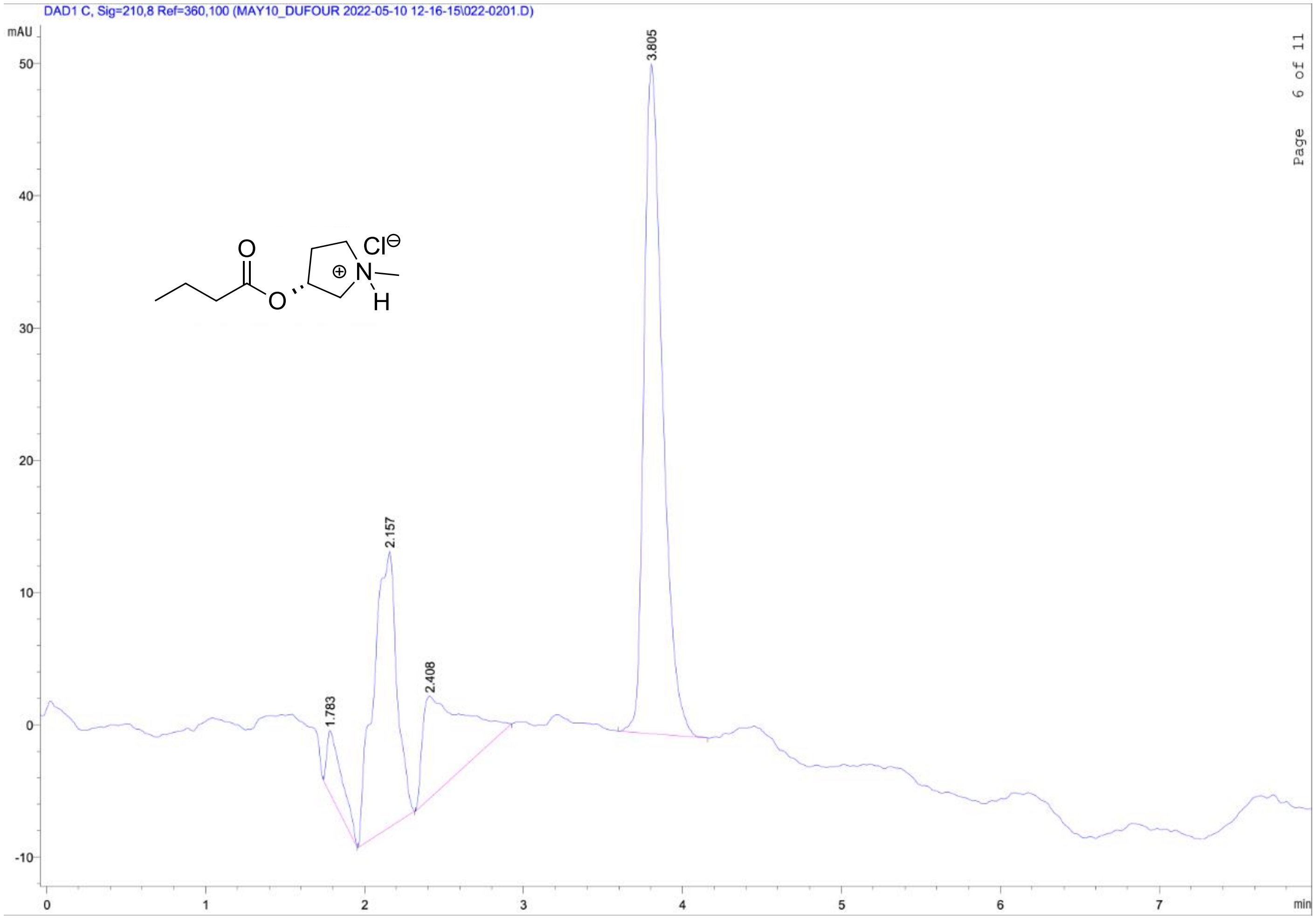

Aryl N-Methylpyrrolidinyl Derivatives

(23) ((*3S*)-1-Methylpyrrolidin-3-yl) *p*-Iodobenzenecarbothioate Oxylate

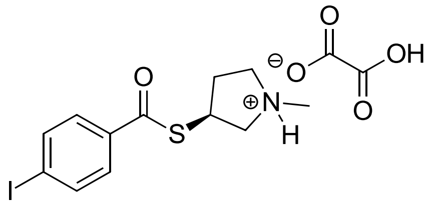

Synthesis: **(*3R*)-3-(Mesyloxy)-1-methylpyrrolidine**: (*R*)-(-)-1-Methyl-3-pyrrolidinol (6.00 mL, 54.6 mmol) was dissolved in anhydrous dichloromethane (180 mL) and cooled to 0°C with an ice bath. Triethylamine (11.4 mL, 81.9 mmol) and methanesulfonyl chloride (4.90 mL, 62.8 mmol) were added dropwise at 0°C. The ice bath was removed after 5 mins, and the reaction was stirred overnight. Brine (50 mL) was added to the reaction and the layers were separated. The aqueous layer was extracted with dichloromethane (3 × 15 mL). The combined organic layers were dried over Na_2SO_3, gravity filtered, and the filtrate was concentrated *in vacuo* to produce a yellow viscous liquid (6.11 g, 54%). Product confirmed by ^{1}H NMR and the crude material was immediately carried through to next step without further purification.

(3*S*)-S-(1-Methylpyrrolidin-3-yl) ethanethioate: (*3R*)-3-(Mesyloxy)-1-methylpyrrolidine (6.11 g, 34.1 mmol), was dissolved in anhydrous THF (114 mL) under an argon atmosphere. 18-crown-6-ether (9.91 g, 37.5 mmol) was added followed by potassium thioacetate (4.29 g, 37.5 mmol), added in 3 portions. Once all potassium thioacetate had gone into solution, the reaction was heated to 30°C and left overnight. The reaction was gravity filtered, the filtrate diluted with water (50 mL) and it was extracted with EtOAc (3 × 15 mL). The combined organic layers were washed with water (3 × 20 mL) and brine (3 × 20 mL), dried over Na_2SO_3, gravity filtered, and concentrated *in vacuo* to produce a red oil. The crude material was then distilled to give (*3S*)-S-(1-methylpyrrolidin-3-yl) ethanethioate as a yellow oil (2.33 g, 37%). The product was confirmed by ^{1}H NMR.

(*S*)-1-Methyl-3-pyrrolidinethiol: (*3S*)-S-(1-Methylpyrrolidin-3-yl) ethanethioate (0.442g, 2.77 mmol) was dissolved in MeOH (10 mL) and sodium methoxide (0.155 g, 2.78 mmol) was added to the stirring solution. After all starting material was shown to be consumed by TLC

experiment, the MeOH was removed *in vacuo* and the resulting material was washed with DCM and concentrated twice. This material was used immediately in the next step.

((*3S*)-1-Methylpyrrolidin-3-yl) *p*-iodobenzenethioate: (*S*)-1-Methyl-3-pyrrolidinethiol from the previous step was dissolved in dichloromethane (10 mL) and cooled to 0°C using an ice bath. 4-Iodobenzoyl chloride (0.740 g, 2.78 mmol) was added followed by triethylamine (0.774 mL, 5.55 mmol). The ice bath was removed after 30 mins and the reaction was left overnight. The next morning the solvent was removed *in vacuo.* The crude material was washed with diethyl ether, gravity filtered to remove all precipitate, and the filtrate was concentrated *in vacuo* to give a white solid. This material recrystallized with methanol and isopropanol to give a white solid (0.727 g).

((*3S*)-1-Methylpyrrolidin-3-yl) *p*-Iodobenzenecarbothioate Oxylate: ((*3S*)-1-Methylpyrrolidin-3-yl) *p*-iodobenzenethioate (0.600 g, 1.73 mmol) was completely dissolved in isopropanol (32.5 mL). With stirring, oxalic acid (0.156 g, 1.73 mmol) was added and stirred for 3 hrs at room temperature. The solution was concentrated *in vacuo* to give a white solid and immediately recrystallized with isopropanol. For further purification, this material was recrystallized from methanol to give ((*3S*)-1-methylpyrrolidin-3-yl) *p*-iodobenzenecarbothioate oxylate as a white solid (0.357 g, 47%).
Spectroscopic Data: IR(ATR): 3041, 2718, 2564, 1714, 1697, 1578, 909 cm^{-1}; ^{1}H NMR (400 MHz, DMSO-*d6*) δ 7.97-7.95 (m, 2H), 7.66-7.64 (m, 2H), 4.51 (br s, 2H, overlapping with H_2O in DMSO), 4.26-4.19 (m, 1H), 3.71 (dd, J = 11.7, 8.1 Hz, 1H), 3.36-3.30 (m, 1H), 3.26-3.21 (m, 2H), 2.77 (s, 3H), 2.58-2.52 (m, 1H), 2.06-1.97 (m, 1H); ^{13}C NMR (100.7 MHz, DMSO-*d6*) δ 190.3 (0), 164.3 (0), 138.9 (1), 135.1 (0), 128.5 (1), 103.0 (0), 59.7 (2), 54.1 (2), 40.4 (3), 38.6 (1), 30.0 (2); HRMS (ESI^+): calculated for $C_{12}H_{15}INOS^+$: 347.9913; found for $C_{12}H_{15}INOS^+$: 347.9912; HPLC purity at 254nm (75% CH_3CN : 10% CH_3OH : 15% aqueous triethylamine [0.1% triethylamine in H_2O], retention time 8.245 mins): 97.2 %.

(23) ((*3S*)-1-Methylpyrrolidin-3-yl) *p*-Iodobenzenecarbothioate Oxylate IR(ATR)

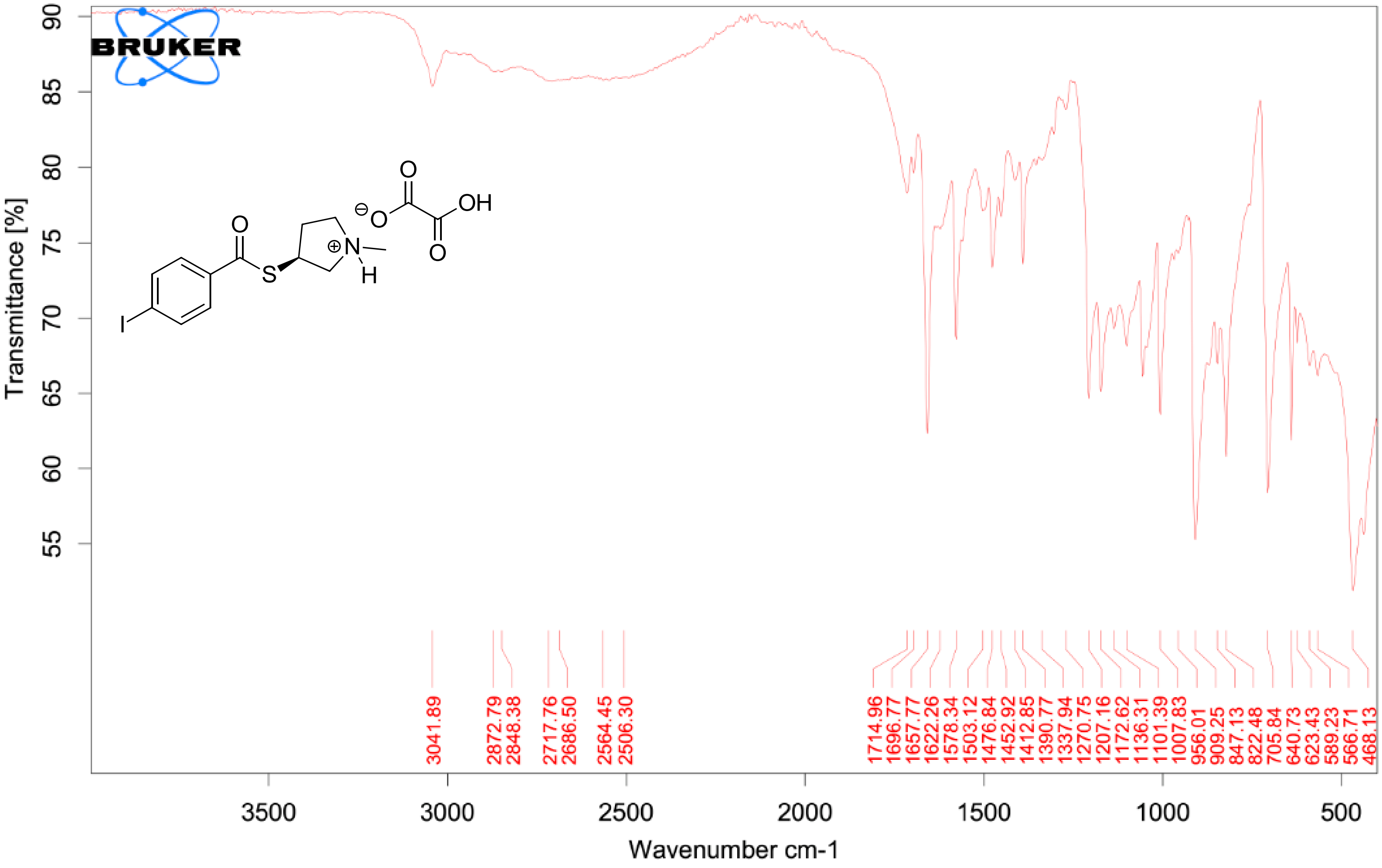

(23) ((*3S*)-1-Methylpyrrolidin-3-yl) *p*-Iodobenzenecarbothioate Oxylate ^{1}H NMR 400 MHz (DMSO-*d6*)

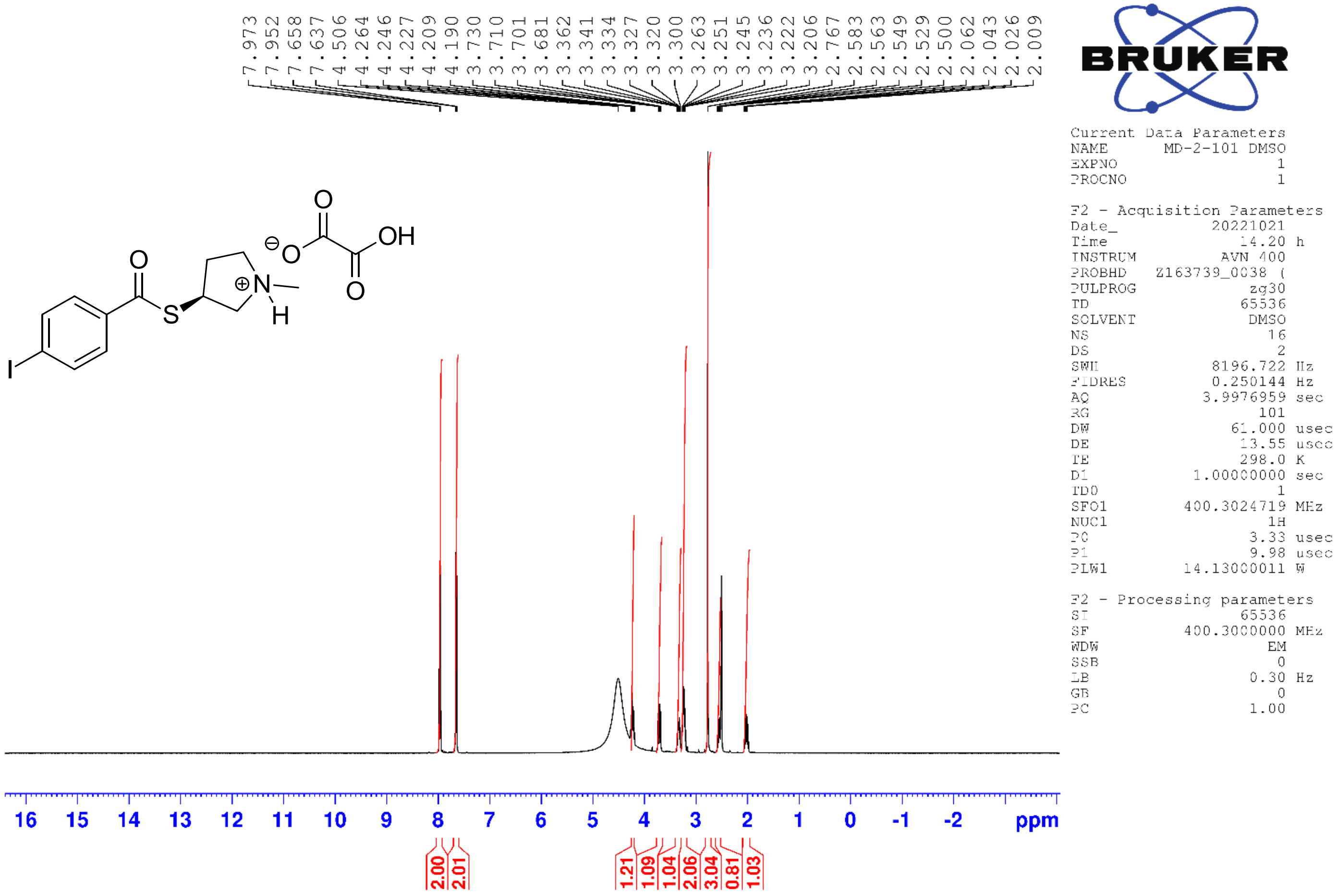

(23) ((*3S*)-1-Methylpyrrolidin-3-yl) *p*-Iodobenzenecarbothioate Oxylate ^{13}C NMR 100 MHz (DMSO-*d6*)

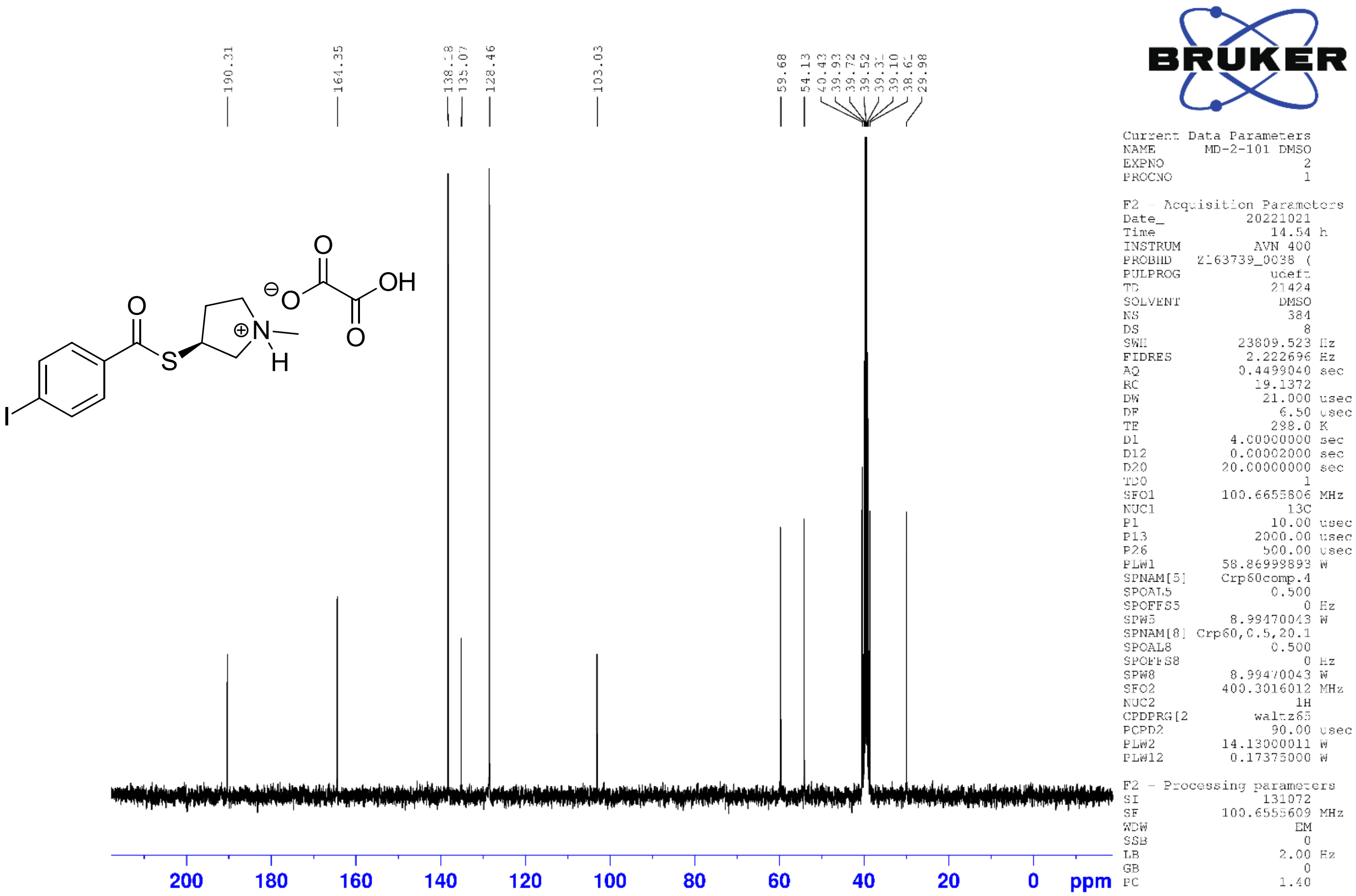

(23) ((*3S*)-1-Methylpyrrolidin-3-yl) *p*-Iodobenzenecarbothioate Oxylate High Resolution Mass Spectrum (ESI, positive mode)

Mass Spectrum SmartFormula Report

Analysis Info

Analysis Name	D:\Data\Xiao\Nov 01 2022000004.d	Acquisition Date	2022-11-01 2:53:21 PM
Method	Xiao all 1.m	Operator	x
Sample Name	TRV 6025	Instrument	compact 8255754.20059
Comment			

Acquisition Parameter

Source Type	ESI	Ion Polarity	Positive	Set Nebulizer	0.5 Bar
Focus	Not active	Set Capillary	3500 V	Set Dry Heater	180 °C
Scan Begin	50 m/z	Set End Plate Offset	-500 V	Set Dry Gas	4.0 l/min
Scan End	1500 m/z	Set Charging Voltage	2000 V	Set Divert Valve	Source
		Set Corona	0 nA	Set APCI Heater	0 °C

Meas. m/z	Ion Formula	m/z	err [ppm]
347.9912	C12H15INOS	347.9914	0.4

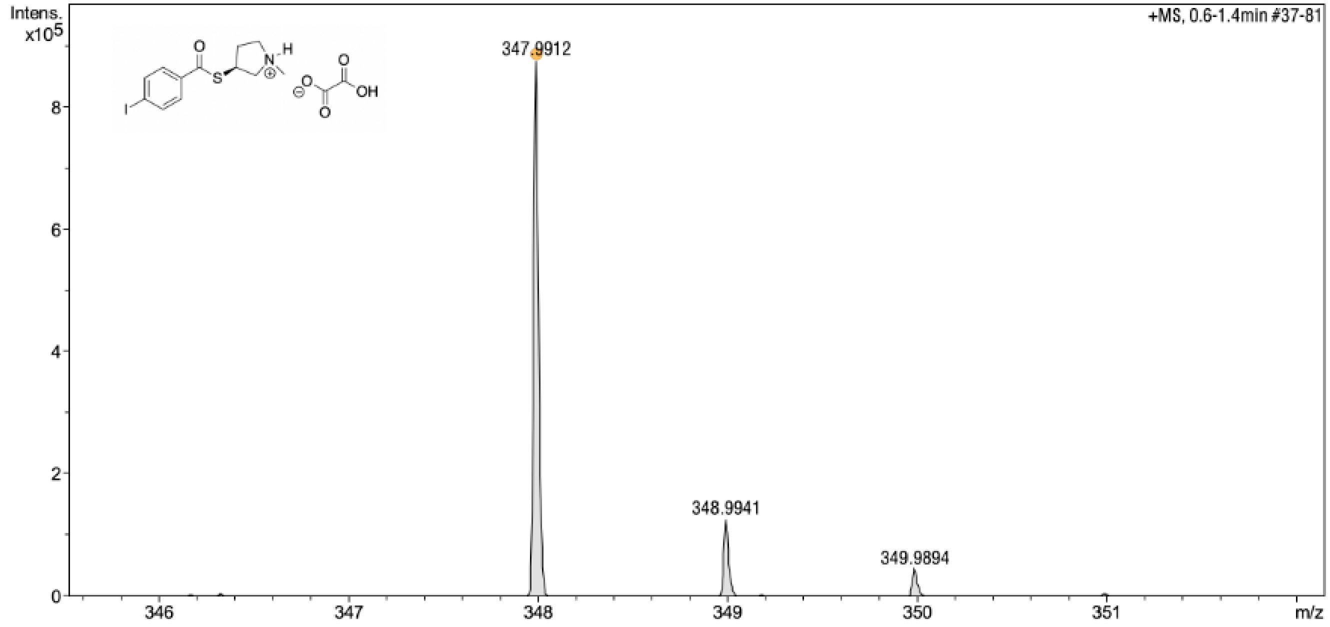

Nov 01 2022000004.d

Bruker Compass DataAnalysis 4.3 printed: 2022-11-08 10:41:42 AM by: x Page 1 of 1

(23) ((*3S*)-1-Methylpyrrolidin-3-yl) *p*-Iodobenzenecarbothioate

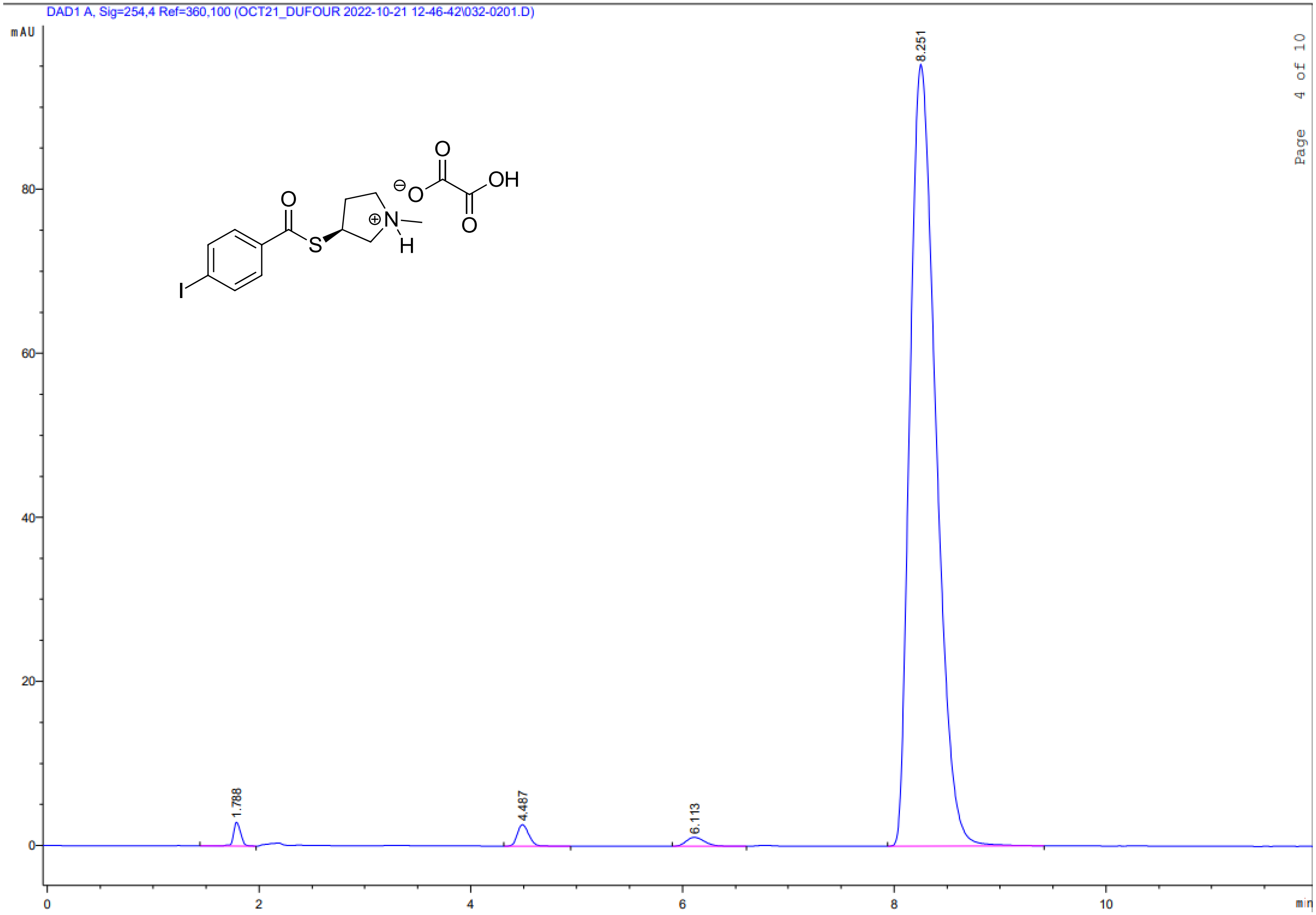

(24) *(3S)*-3-(*p*-Iodobenzoyloxy)-1-methyl-1-pyrrolidinium Chloride

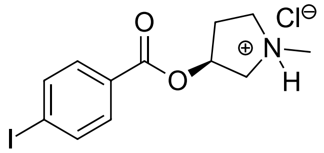

Synthesis: **(3*S*)-1-Methyl-3-pyrrolidinyl *p*-iodobenzoate**: 4-Iodobenzoyl chloride (0.799 g, 3.00 mmol), triethylamine (1.05 mL, 7.50 mmol) and 4-dimethylaminopyridine (DMAP) (0.0659 g, 0.54 mmol) were dissolved in anhydrous dichloromethane (20 mL) under an argon atmosphere. (*S*)-(+)-1-Methyl-3-pyrrolidinol (0.329 mL, 3.00 mmol) was added at 0°C. The ice bath was removed after 5 min, and the reaction was stirred for 3h at room temperature. The reaction was quenched with brine (20 mL), extracted with dichloromethane (3 x 15 mL) and the combined organic layers were dried over Na_2SO_3. After the drying agent was removed *via* filtration the solvent was removed *in vacuo* to produce a white solid. The crude mixture was purified by silica gel column chromatography (5% MeOH/DCM) to afford (3*S*)-1-methyl-3-pyrrolidinyl *p*-iodobenzoate (0.274 g, 28%) as a white solid. The analytical data was similar to previously published data.[7]

***(3S)*-3-(*p*-Iodobenzoyloxy)-1-methyl-1-pyrrolidinium chloride**: (3*S*)-1-Methyl-3-pyrrolidinyl *p*-iodobenzoate (0.136 g, 0.410 mmol) was dissolved in anhydrous diethyl ether (9 mL) under an argon atmosphere. HCl in diethyl ether (1 N - 0.62 mL, 0.620 mmol) was added dropwise resulting in the immediate formation of a white precipitate. The reaction was stirred for an additional 60 mins. The precipitate was collected *via* suction filtration, washed with Et_2O (2 x 10 mL), to afford *(3S)*-3-(*p*-Iodobenzoyloxy)-1-methyl-1-pyrrolidinium chloride, (0.100 mg, 66% yield) as a white solid.

Spectroscopic data: $MP_{(diethyl\ ether)}$: 243-245°C; IR (ATR) 3005, 2931, 2897, 2574, 2472, 1714, 1586, 1269, 1102, 753; ^1H NMR (400 MHz, D_2O) δ 7.93-7.91 (m, 2H), 7.75-7.72 (m, 2H), 5.68 (br app s, 1H), 3.80-3.70 (m, 3H), 3.50 (br app s, 1H), 3.03 (s, 3H), 2.69-2.60 (m, 1H), 2.45-2.39 (m, 1H); ^{13}C NMR (100.7 MHz, D_2O) δ 167.7 (0), 138.7 (1), 131.5 (1), 128.9 (0), 102.3 (0), 74.7 (1), 61.1 (2), 55.1 (2), 42.0 (2), 31.1 (2); LRMS (ESI^+): 332.0 (M+); HRMS (ESI^+): calculated for $C_{12}H_{15}INO_2^+$: 332.0142; found for $C_{12}H_{15}INO_2^+$: 332.0133; HPLC purity at 254 nm (75%

CH_3CN : 10% CH_3OH, 15 % aqueous triethylamine (0.1 % triethylamine in water) retention time: 5.723 mins): >99%.

(24) *(3S)*-3-(*p*-Iodobenzoyloxy)-1-methyl-1-pyrrolidinium Chloride IR(ATR)

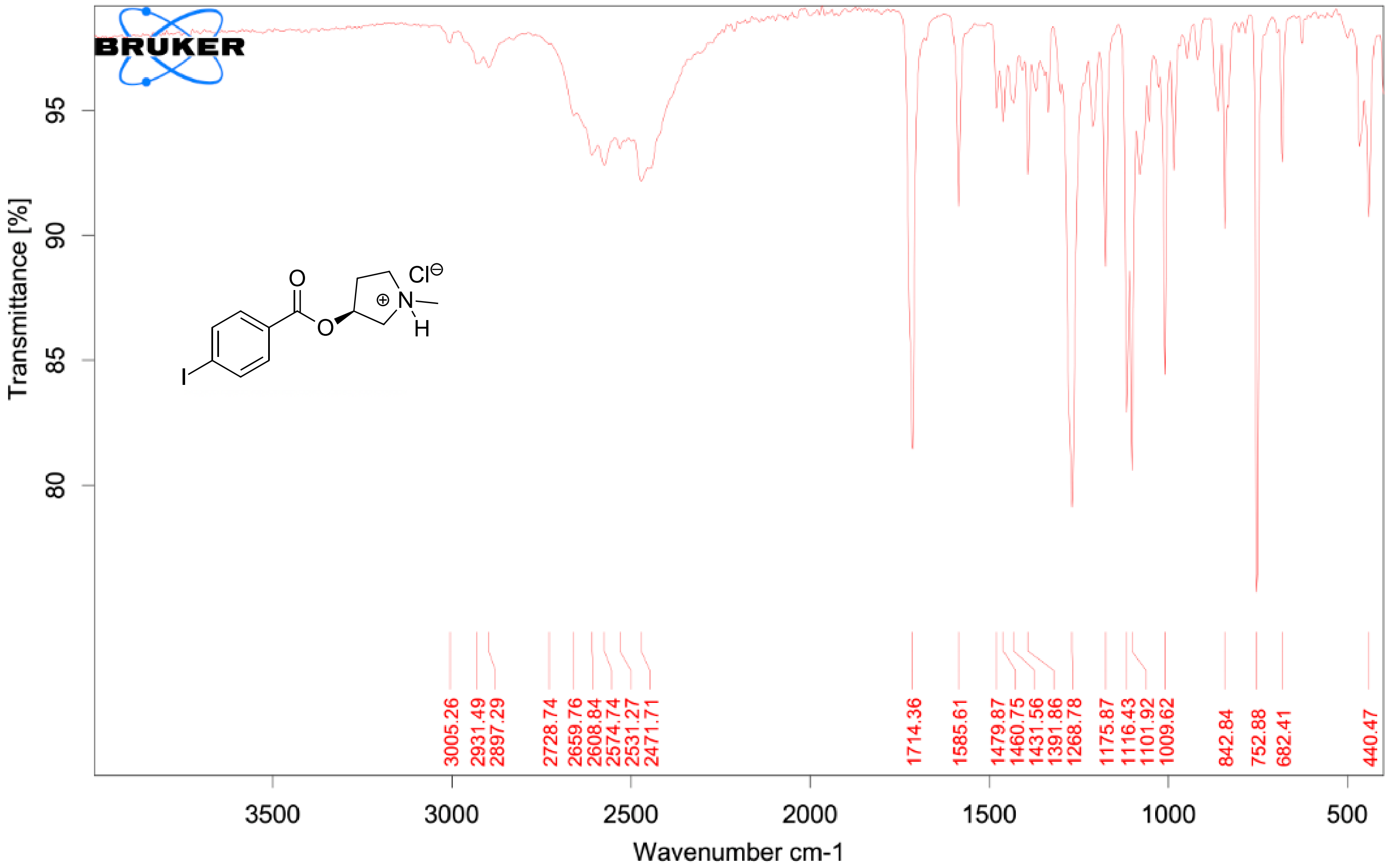

(24) *(3S)*-3-(*p*-Iodobenzoyloxy)-1-methyl-1-pyrrolidinium Chloride ^{1}H NMR 400 MHz (D_2O)

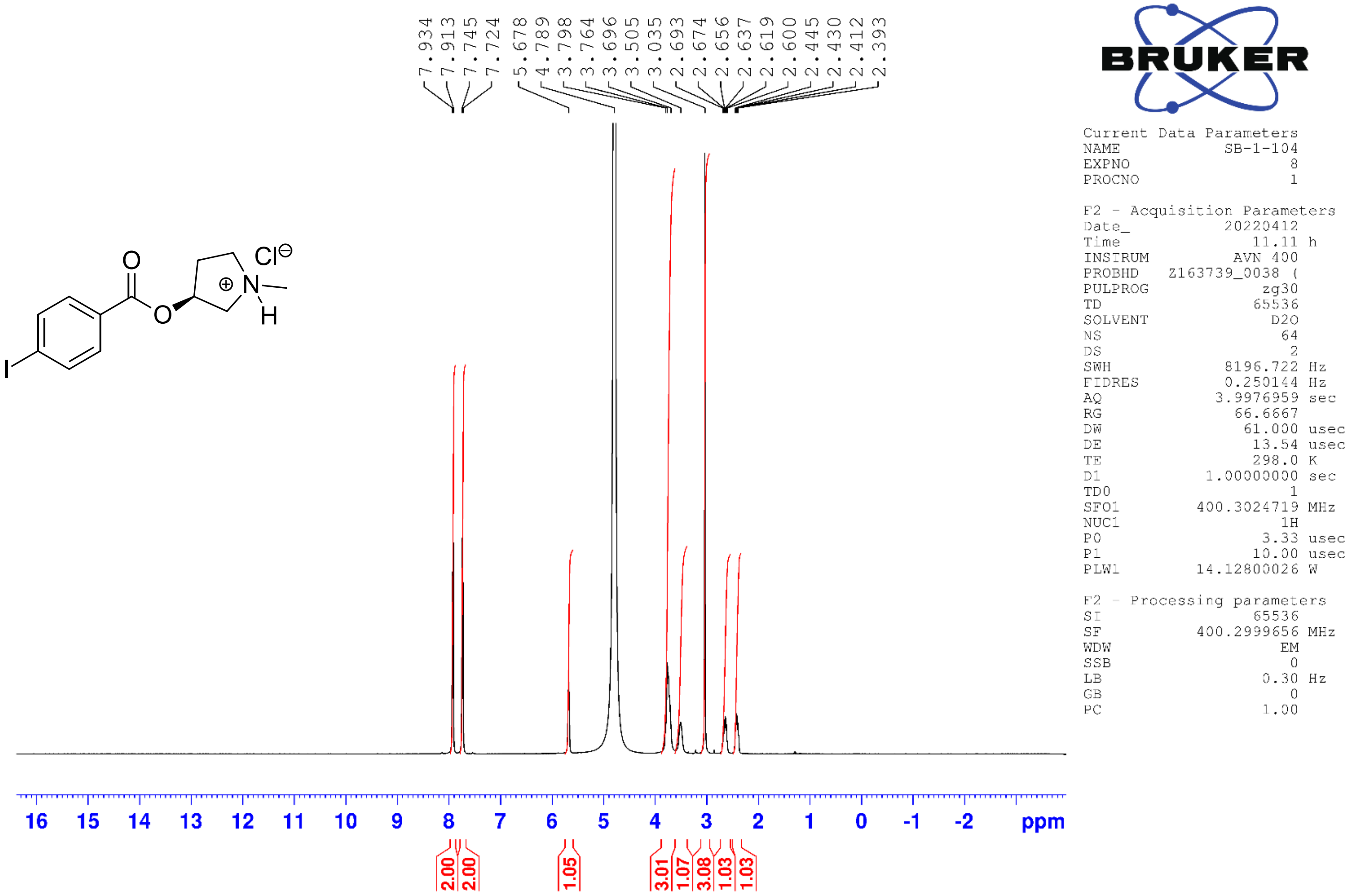

(24) *(3S)*-3-(*p*-Iodobenzoyloxy)-1-methyl-1-pyrrolidinium Chloride ^{13}C UDEFT NMR 100 MHz (D_2O)

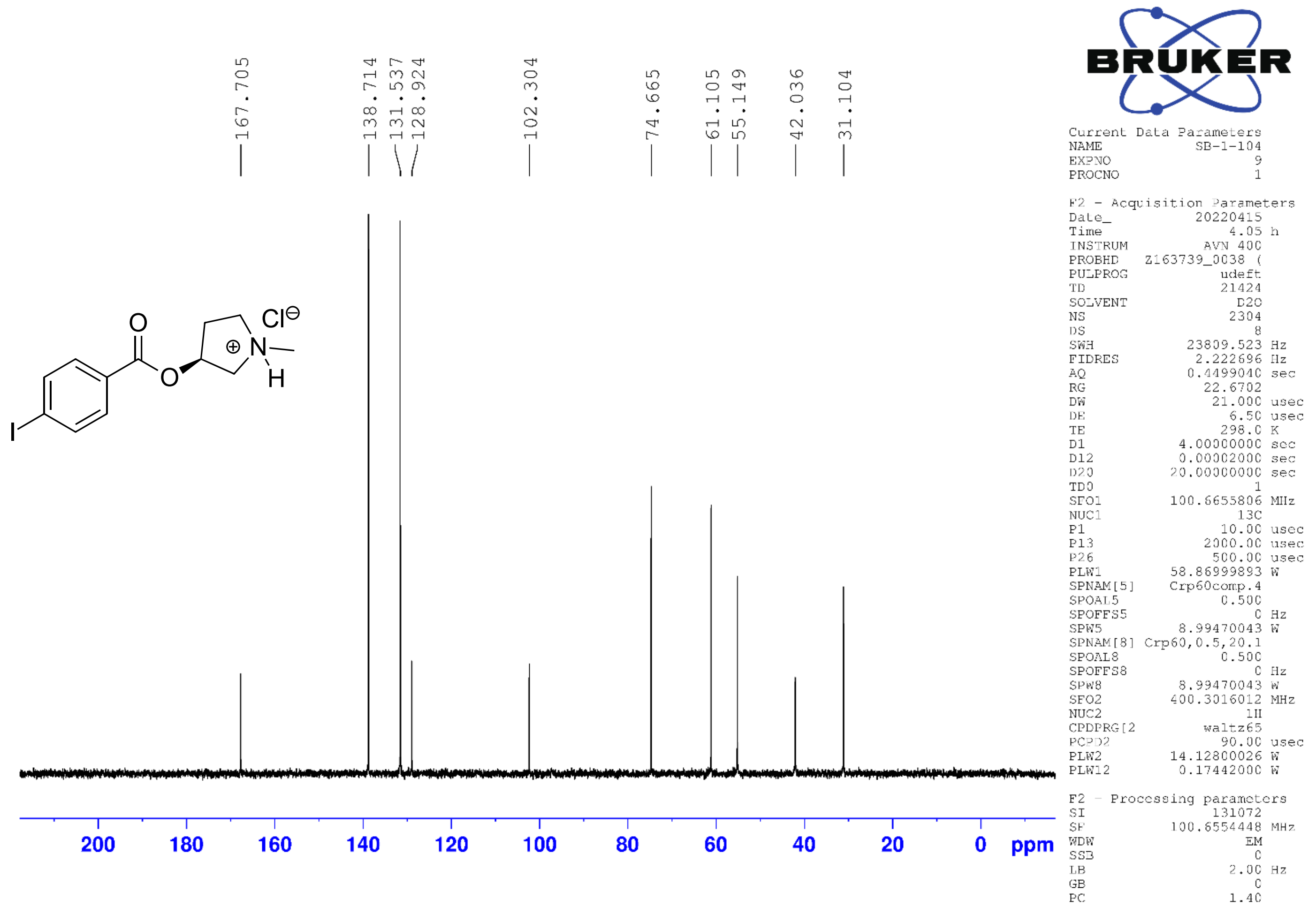

(24) *(3S)*-3-(*p*-Iodobenzoyloxy)-1-methyl-1-pyrrolidinium Chloride High Resolution Mass Spectrum (ESI, positive mode)

Mass Spectrum SmartFormula Report

Analysis Info

Analysis Name	D:\Data\Xiao\July 07 2022000021.d	Acquisition Date	7/7/2022 11:30:06 AM	
Method	Xiao all 1.m	Operator	x	
Sample Name	TRV 6002 HCl	Instrument	compact	8255754.20059
Comment				

Acquisition Parameter

Source Type	ESI	Ion Polarity	Positive	Set Nebulizer	0.5 Bar
Focus	Not active	Set Capillary	3500 V	Set Dry Heater	181 °C
Scan Begin	50 m/z	Set End Plate Offset	-500 V	Set Dry Gas	4.0 l/min
Scan End	1500 m/z	Set Charging Voltage	2000 V	Set Divert Valve	Source
		Set Corona	0 nA	Set APCI Heater	0 °C

Meas. m/z	Ion Formula	m/z	err [ppm]
332.0133	C12H15INO2	332.0142	2.7

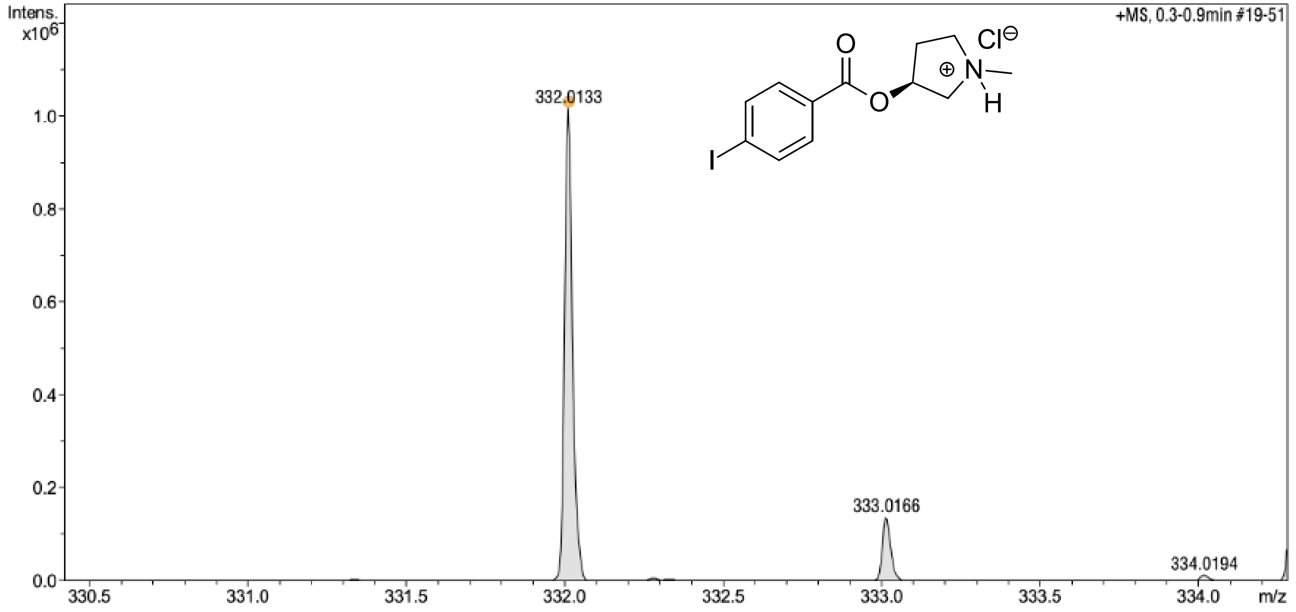

July 07 2022000021.d

Bruker Compass DataAnalysis 4.3 printed: 7/7/2022 11:35:57 AM by: x Page 1 of 1

(24) *(3S)*-3-(*p*-Iodobenzoyloxy)-1-methyl-1-pyrrolidinium Chloride HPLC

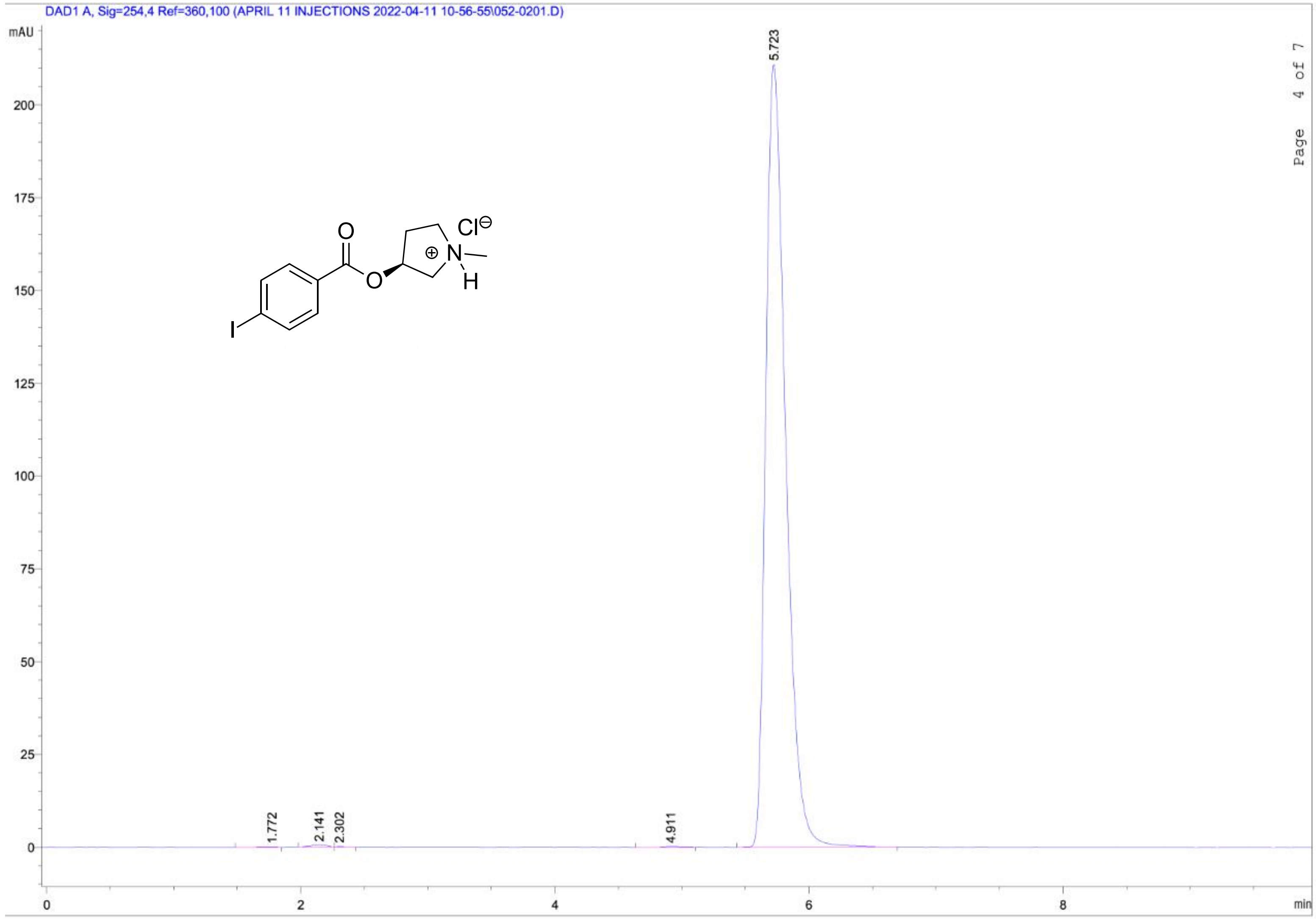

(25) ((*3S*)-1-Methylpyrrolidin-3-yl) *p*-Fluorobenzenecarbothioate Oxylate

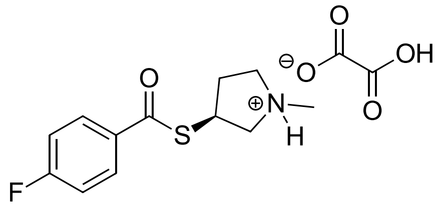

Synthesis: ***(3R*)-3-(Mesyloxy)-1-methylpyrrolidine**: (*R*)-(-)-1-Methyl-3-pyrrolidinol (6.00 mL, 54.6 mmol) was dissolved in anhydrous dichloromethane (180 mL) and cooled to 0°C with an ice bath. Triethylamine (11.4 mL, 81.9 mmol) and methanesulfonyl chloride (4.90 mL, 62.8 mmol) were added dropwise at 0°C. The ice bath was removed after 5 min, and the reaction was stirred overnight. Brine (50 mL) was added to the reaction and the layers were separated. The aqueous layer was extracted with dichloromethane (3 x 15 mL). The combined organic layers were dried over Na_2SO_3, gravity filtered, and the filtrate was concentrated *in vacuo* to produce a yellow viscous liquid (6.11 g, 54%). Product was confirmed by ^{1}H NMR and the crude material was immediately carried through to next step without further purification.

(*3S*)-S-(1-Methylpyrrolidin-3-yl) ethanethioate: (*3R*)-3-(Mesyloxy)-1-methylpyrrolidine (6.11 g, 34.1 mmol) was dissolved in anhydrous THF (114 mL) under an argon atmosphere. 18-crown-6-ether (9.91 g, 37.5 mmol) was added followed by potassium thioacetate (4.29 g, 37.5 mmol), added in 3 portions. Once all potassium thioacetate had gone into solution, the reaction was heated to 30°C and left overnight. The reaction was gravity filtered, the filtrate diluted with water (50 mL) and it was extracted with EtOAc (3 × 15 mL). The combined organic layers were washed with water (3 × 20 mL) and brine (3 × 20 mL), dried over Na_2SO_3, gravity filtered, and concentrated *in vacuo* to produce a red oil. The crude material was then distilled to give (*3S*)-S-(1-methylpyrrolidin-3-yl) ethanethioate as a yellow oil (2.33 g, 37 %). The product was confirmed by ^{1}H NMR and the material was immediately carried through to next step without further purification.

(*S*)-1-Methyl-3-pyrrolidinethiol: (*3S*)-S-(1-Methylpyrrolidin-3-yl) ethanethioate (0.344 g, 2.16 mmol) was dissolved in MeOH (11 mL) and sodium methoxide (0.120 g, 2.16 mmol) was added to the stirring solution. After all starting material was shown to be consumed by TLC

experiment, the MeOH was removed *in vacuo*. The resulting material was placed dichloromethane (20 mL) and concentrated *in vacuo*. This process was repeated, and the material was immediately used in the next step.

((*3S*)-1-Methylpyrrolidin-3-yl) *p*-fluorobenzenethioate: (*S*)-1-Methyl-3-pyrrolidinethiol was dissolved in dichloromethane (7 mL) and cooled to 0°C using an ice bath. 4-Fluorobenzoyl chloride (0.602 g, 4.32 mmol) was added followed by triethylamine (0.774 mL, 5.55 mmol). The ice bath was removed after 30 mins and warmed to room temperature. After all starting material was shown to be consumed by TLC experiment (developed using $KMnO_4$ stain), the reaction was concentrated *in vacuo.* The crude material was washed with diethyl ether, gravity filtered to remove all precipitate, and the filtrate was concentrated *in vacuo* to give a yellow oil (0.406 g).

((*3S*)-1-Methylpyrrolidin-3-yl) *p*-fluorobenzenecarbothioate oxylate: A portion of ((*3S*)-1-methylpyrrolidin-3-yl) *p*-fluorobenzenethioate (0.0.324 g, 1.36 mmol) was completely dissolved in isopropanol (14 mL). With stirring, oxalic acid (0.220 g, 1.36 mmol) was added and stirred for 3 hrs at room temperature. The solution was concentrated *in vacuo* to give a white solid and immediately recrystallized from isopropanol to give ((*3S*)-1-methylpyrrolidin-3-yl) *p*-fluorobenzenecarbothioate oxylate as a white solid (0.289 g, 64%).
Spectroscopic Data: $MP_{(isopropanol)}$: 130-134°C; IR(ATR): 3036, 2961, 2713, 2591, 1717, 1698, 1598, 913 cm^{-1}; ^{1}H NMR (400 MHz, DMSO-*d6*) δ 8.00-7.96 (m, 2H), 7.44-7.38 (m, 2H), 4.52 (br s, 2H, overlapping with H_2O in DMSO), 4.27-4.20 (m, 1H), 3.72 (dd, J = 11.8, 8.1 Hz, 1H), 3.37-3.31 (m, 1H), 3.28-3.22 (m, 2H), 2.78 (s, 3H), 2.59-2.52 (m, 1H), 2.07-1.98 (m, 1H); ^{13}C NMR (100.7 MHz, DMSO-*d6*) δ 189.4 (0), 166.8 (d,$^1J_{C,F}$ = 252.7 Hz, 0), 164.3 (1), 132.5 (d, $^4J_{C,F}$ = 2.8 Hz, 0), 129.9 (d,$^3J_{C,F}$ = 9.6 Hz, 1), 116.4 (d,$^2J_{C,F}$ = 22.4 Hz, 1), 59.7 (2), 54.1 (2), 40.4 (3), 38.6 (1), 30.0(2); ^{19}F NMR (376.6 MHz, DMSO-*d6*) δ -104.1; HRMS (ESI^+): calculated for $C_{12}H_{15}FNOS^+$: 240.0853; found for $C_{12}H_{15}FNOS^+$: 240.0853; HPLC purity at 254nm (75% CH_3CN : 10% CH_3OH : 15% aqueous triethylamine [0.1% triethylamine in H_2O], retention time 4.817 mins): 98.2 %.

(25) ((*3S*)-1-Methylpyrrolidin-3-yl) *p*-Fluorobenzenecarbothioate Oxylate IR(ATR)

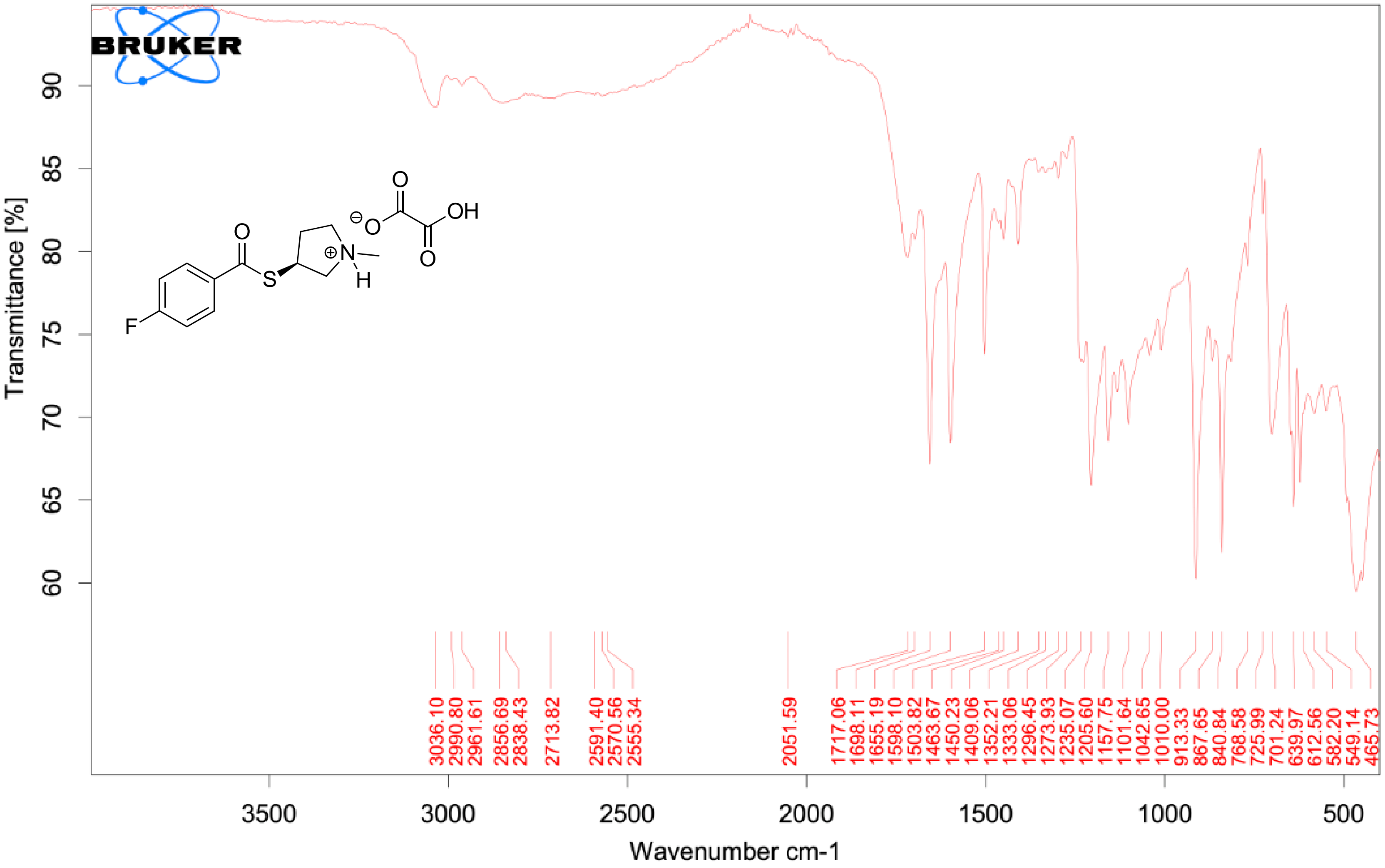

(25) ((*3S*)-1-Methylpyrrolidin-3-yl) *p*-Fluorobenzenecarbothioate Oxylate ^{1}H NMR 400 MHz (DMSO-*d6*)

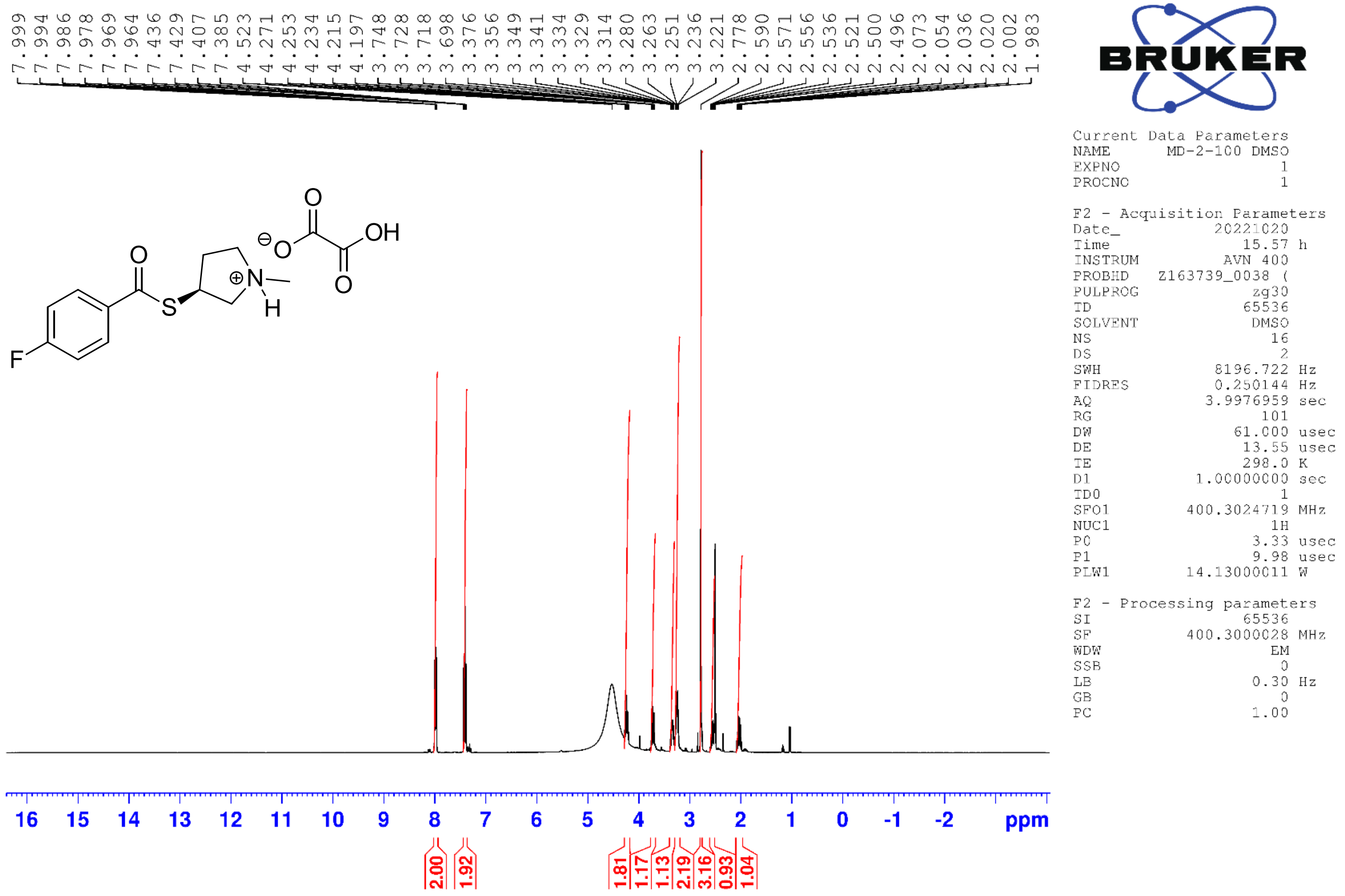

(25) ((*3S*)-1-Methylpyrrolidin-3-yl) *p*-Fluorobenzenecarbothioate Oxylate ^{13}C NMR 100 MHz (DMSO-*d6*)

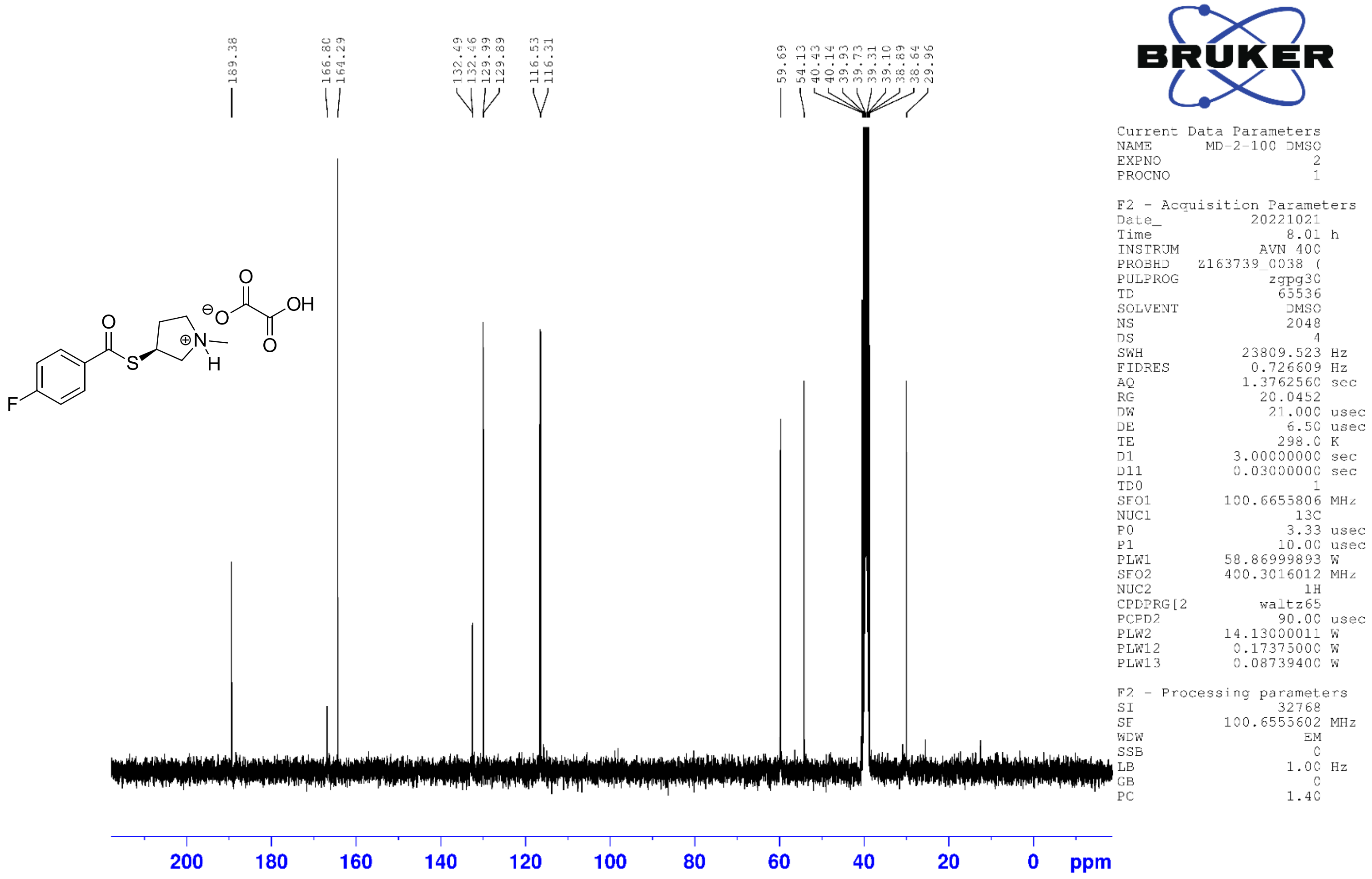

(25) ((3*S*)-1-Methylpyrrolidin-3-yl) *p*-Fluorobenzenecarbothioate Oxylate High Resolution Mass Spectrum (ESI, positive mode)

Mass Spectrum SmartFormula Report

Analysis Info

Analysis Name	D:\Data\Xiao\Nov 01 2022000002.d	Acquisition Date	2022-11-01 2:43:55 PM	
Method	Xiao all 1.m	Operator	x	
Sample Name	TRV 6026	Instrument	compact	8255754.20059
Comment				

Acquisition Parameter

Source Type	ESI	Ion Polarity	Positive	Set Nebulizer	0.5 Bar
Focus	Not active	Set Capillary	3500 V	Set Dry Heater	180 °C
Scan Begin	50 m/z	Set End Plate Offset	-500 V	Set Dry Gas	4.0 l/min
Scan End	1500 m/z	Set Charging Voltage	2000 V	Set Divert Valve	Source
		Set Corona	0 nA	Set APCI Heater	0 °C

Meas. m/z	Ion Formula	m/z	err [ppm]
240.0853	C12H15FNOS	240.0853	0.2

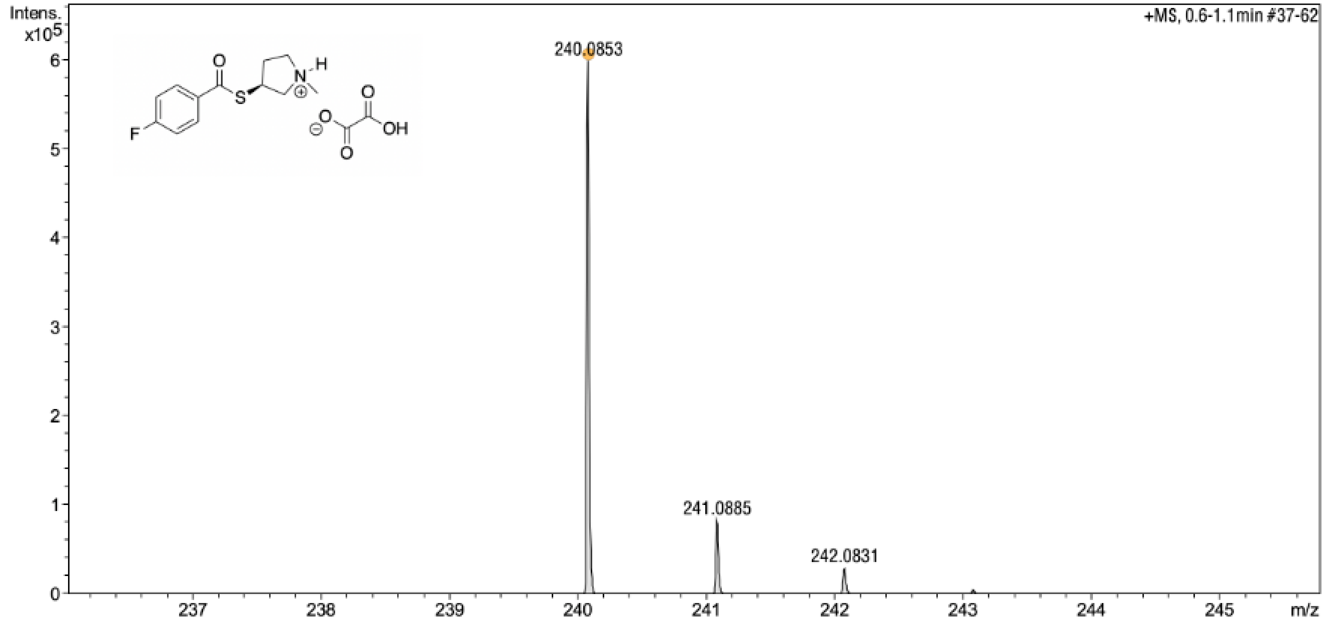

Nov 01 2022000002.d

Bruker Compass DataAnalysis 4.3 printed: 2022-11-08 10:39:33 AM by: x Page 1 of 1

(25) ((*3S*)-1-Methylpyrrolidin-3-yl) *p*-Fluorobenzenecarbothioate Oxylate HPLC

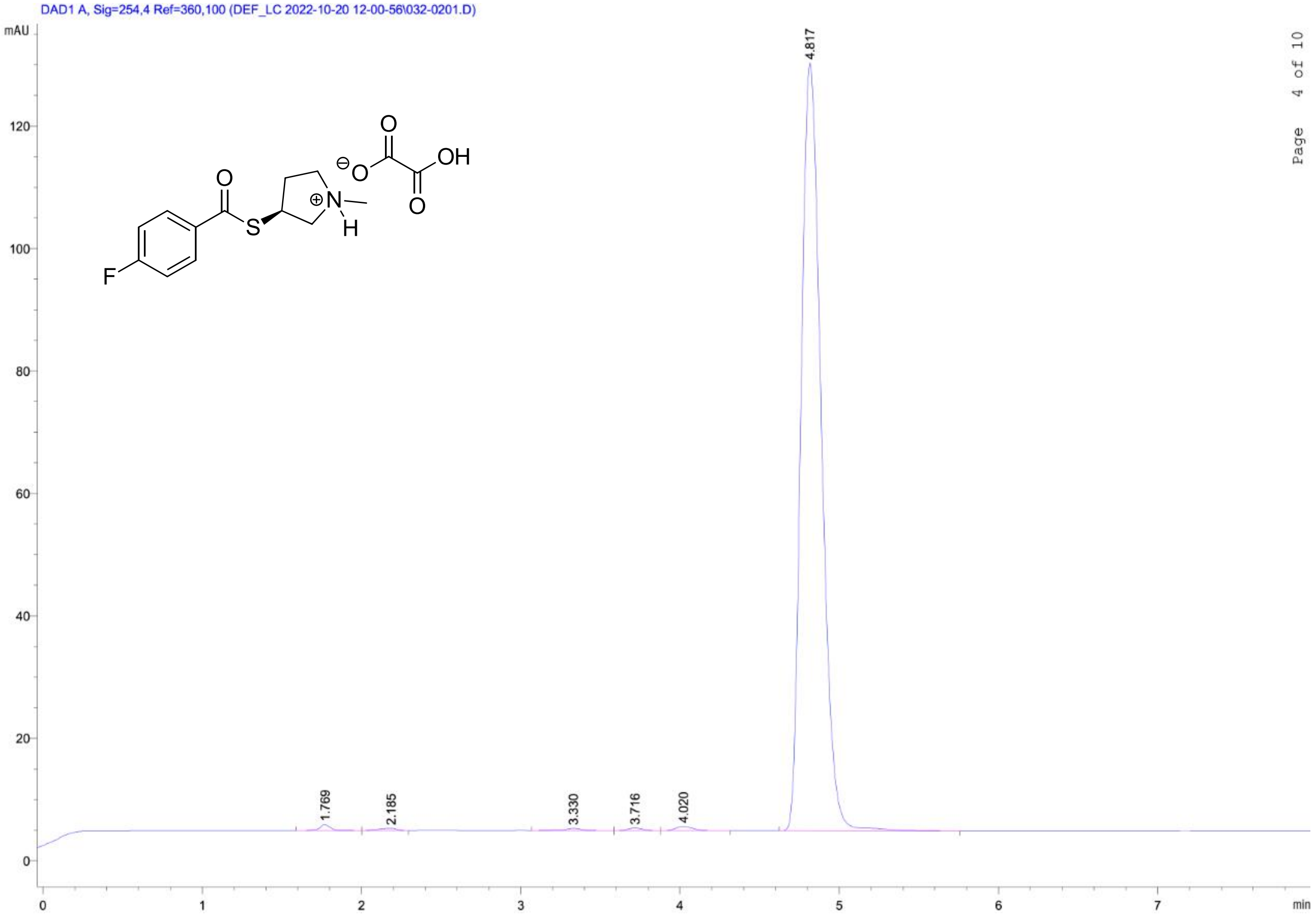

(26) (*3S*)-3-(*p*-Fluorobenzoyloxy)-1-methyl-1-pyrrolidinium Chloride

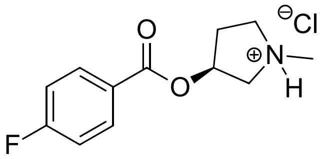

Synthesis: **(*3S*)-1-Methyl-3-pyrrolidinyl *p*-fluorobenzoate**: (*3S*)-(+)-1-Methyl-3-pyrrolidinol (0.20 mL, 1.82 mmol) was dissolved in anhydrous dichloromethane (6.00 mL) under an argon atmosphere and cooled to 0°C. To this solution, triethylamine (0.501 mL, 3.64 mmol) and 4-fluorobenzoyl chloride (0.218 g, 1.82 mmol). The ice bath was removed after 5 min, and the reaction was stirred for 24 hrs. At this time water (20 mL) was added, layers were separated, and the aqueous layer was extracted with dichloromethane (3 × 15 mL). The combined organic layers were dried over Na_2SO_3, gravity filtered, and the filtrate was concentrated *in vacuo* to produce an orange oil. This crude material was purified by flash chromatography through silica gel (5% MeOH/DCM/1%TEA) to afford (*3S*)-1-methyl-3-pyrrolidinyl *p*-fluorobenzoate as an orange oil (0.338 g, 83%).

Spectroscopic data: IR(ATR): 2965, 2942, 2777, 1712, 1603, 1507, 1268, 1113, 688 cm^{-1}; ^{1}H NMR (400 MHz, DMSO-*d6*) δ 8.03-7.99 (m, 2H), 7.37-7.33 (m, 2H), 5.31-5.27 (m, 1H), 2.75-2.69 (m, 3H), 2.33-2.25 (m, 2H), 2.26 (s, 3H), 1.90-1.81 (m, 1H); ^{13}C NMR (100.7 MHz, DMSO-*d6*) δ 165.1 (d, $^{1}J_{C,F}$ = 251.3 Hz, 0), 164.7 (0), 132.1 (d, , $^{3}J_{C,F}$ = 9.7 Hz, 1), 126.4 (d, $^{4}J_{C,F}$ = 2.7 Hz, 0), 115.9 (d, $^{2}J_{C,F}$ = 22.1 Hz, 1), 75.3 (1), 61.6 (2), 54.4 (2), 41.6 (3), 32.2 (2); HPLC purity at 254 nm (75% CH_3CN : 10% CH_3OH : 15% aqueous triethylamine [0.1% triethylamine in H_2O], retention time 4.106 mins): 98.7 %.

(*3S*)-3-(*p*-Fluorobenzoyloxy)-1-methyl-1-pyrrolidinium Chloride: S-((*3S*)1-Methylpyrolidin-3-yl) *p*-fluorobenzenecarbothioate (0.2484 g, 1.11 mmol) was dissolved in anhydrous diethyl ether (22mL) under an argon atmosphere. HCl in diethyl ether 1 N (1.7 mL, 1.67 mmol) was added dropwise and stirred for 3 hrs. The solvent was removed *in vacuo* resulting in a white solid. The compound was triturated three times with diethyl ether (20mL), solvent was removed with a pipet and compound was dried to produce (*3S*)-3-(*p*-fluorobenzoyloxy)-1-methyl-1-pyrrolidinium chloride (0.148 g, 51%) as a mixture of isomers.

Spectroscopic data: $MP_{(diethyl\ ether)}$: 143-145 °C; IR(ATR): 3051, 2953, 2927, 2647, 2566, 2530, 1719, 1603, 1269, 1113, 766 cm^{-1}; ^{1}H NMR (400 MHz, DMSO-*d6*) δ 11.17 (br s, 1H), 8.17-8.11 (m, 2H), 7.40-7.35 (m, 2H), 5.55-5.49 (m, 1H), 3.93-3.63 (m, 2H), 3.30-3.24 (m, 1H), 3.14-3.02 (m, 1H), 2.85 (s, 3H), 2.58-2.14 (m, 2H); ^{13}C NMR (100.7 MHz, DMSO-*d6*) δ 165.4 (d, $^{1}J_{C,F}$ = 251.8 Hz, 0), 164.2 (0), 132.6 (1), 125.9 (0), 115.8 (d, $^{2}J_{C,F}$ = 22.5 Hz, 1), 73.3 (1), 59.5 (2), 53.2 (2), 40.4 (3), 30.3 (2); HRMS (ESI^{+}): calculated for $C_{12}H_{15}FNO_2^{+}$: 224.1081 amu; found for $C_{12}H_{15}FNO_2^{+}$: 224.1083 amu; HPLC purity at 254nm (75% CH_3CN : 10% CH_3OH : 15% aqueous triethylamine [0.1% triethylamine in H_2O], retention time 4.118 mins): 98.8 %.

(26) (3*S*)-3-(*p*-Fluorobenzoyloxy)-1-methyl-1-pyrrolidinium Chloride IR(ATR)

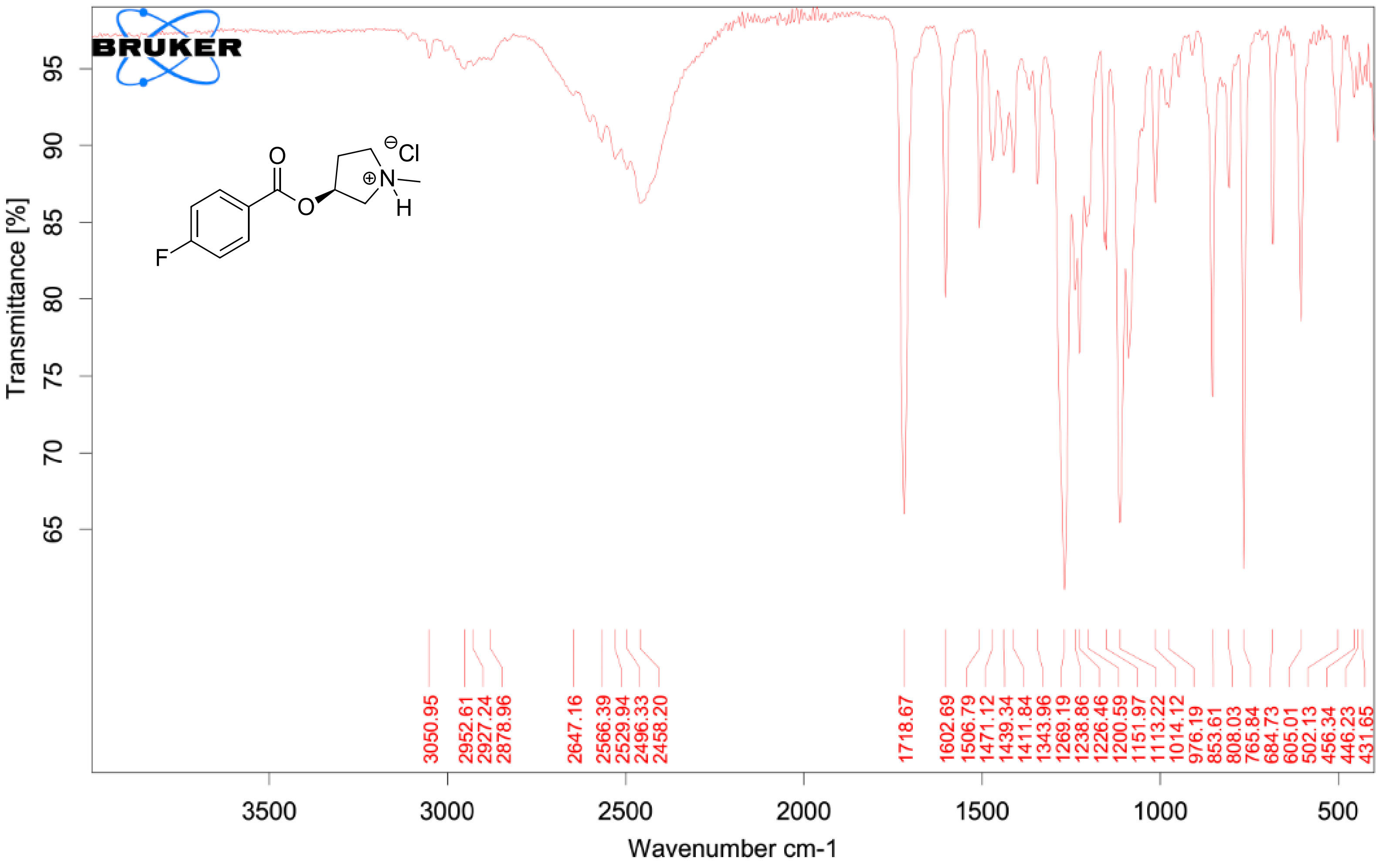

(26) (*3S*)-3-(*p*-Fluorobenzoyloxy)-1-methyl-1-pyrrolidinium Chloride ^{1}HNMR 400 MHz (DMSO-*d6*)

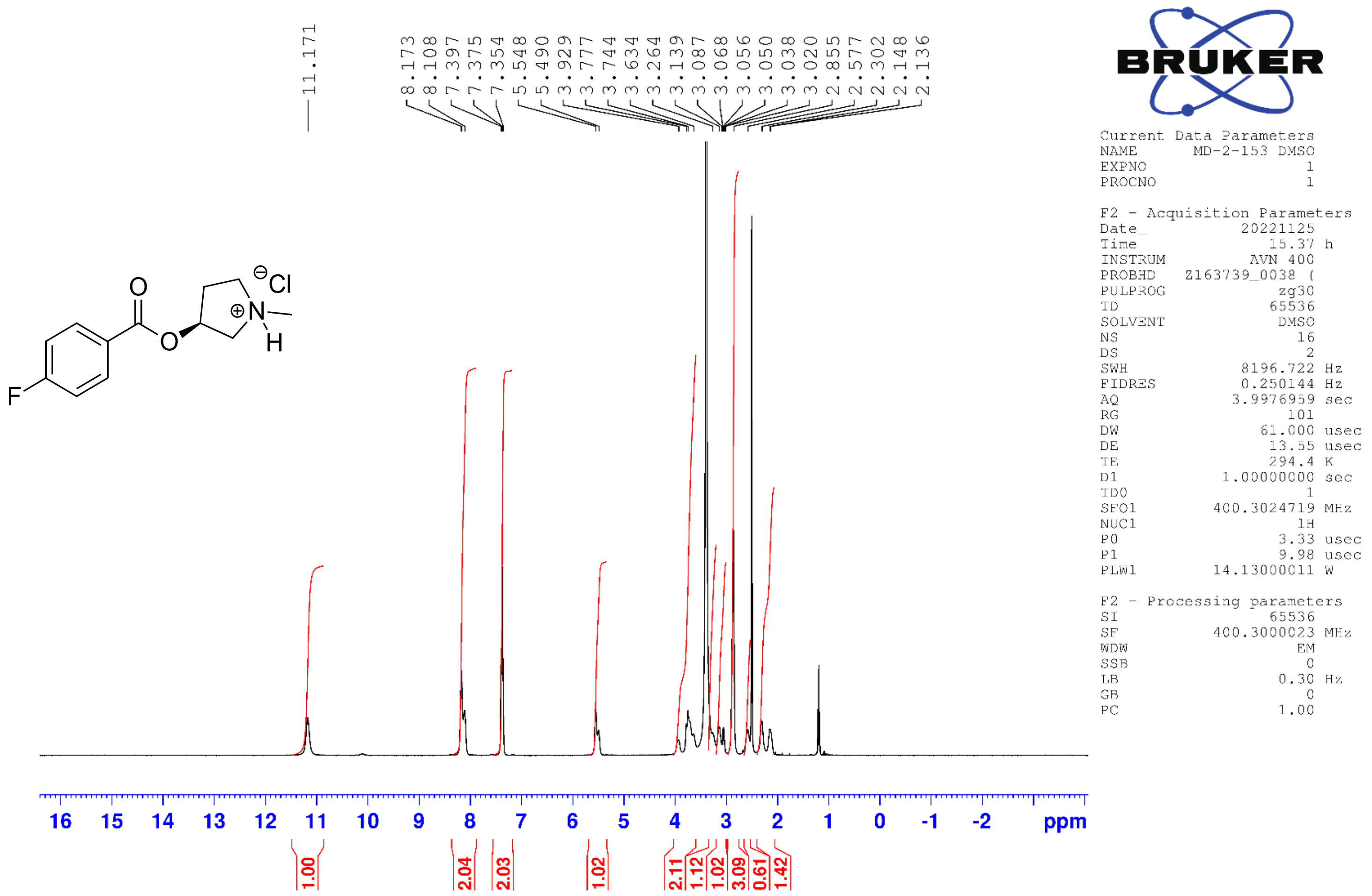

(26) (*3S*)-3-(*p*-Fluorobenzoyloxy)-1-methyl-1-pyrrolidinium Chloride ^{13}CNMR 100 MHz (DMSO-*d6*)

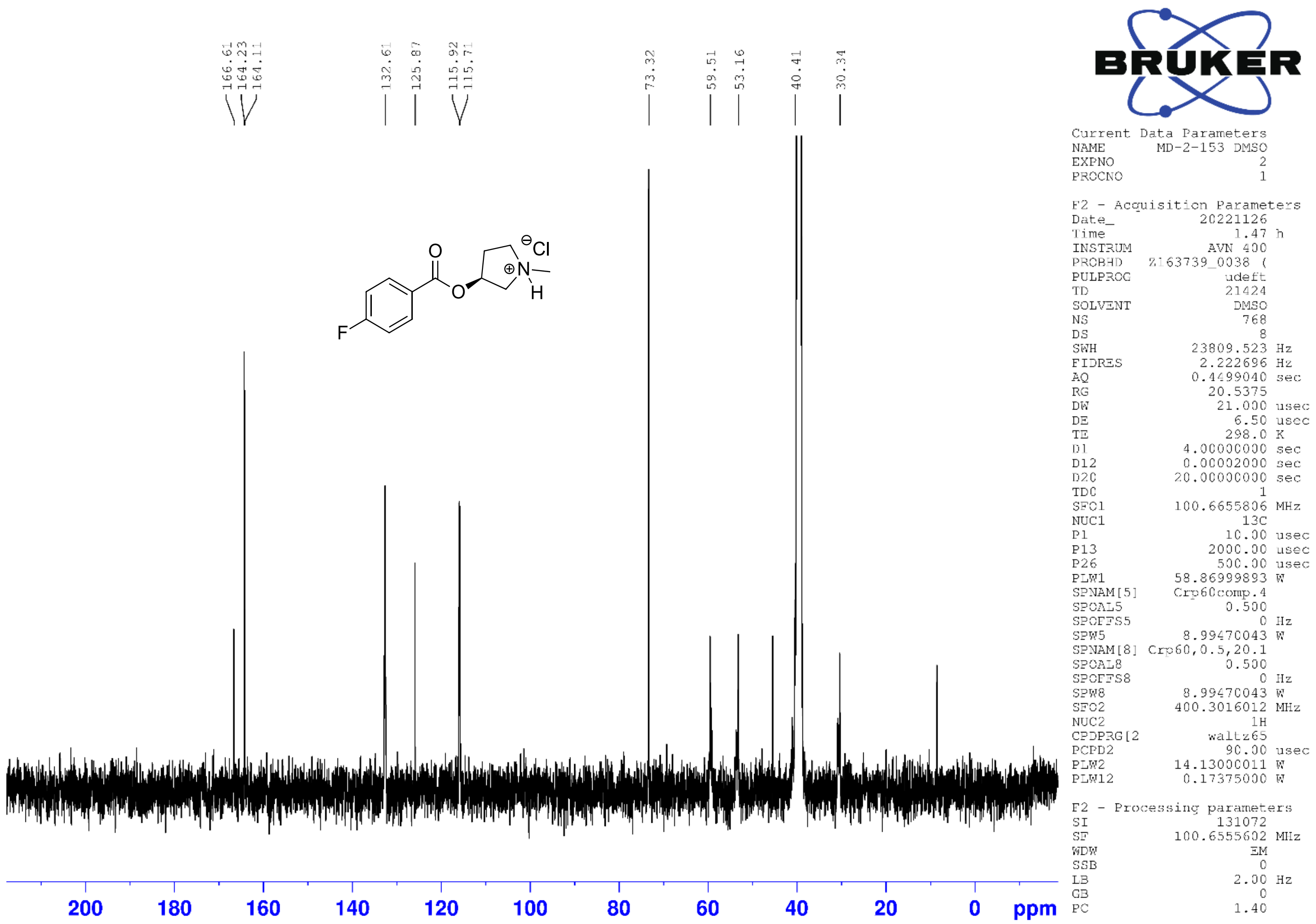

(26) (*3S*)-3-(*p*-Fluorobenzoyloxy)-1-methyl-1-pyrrolidinium Chloride High Resolution Mass Spectrum (ESI, positive mode)

Mass Spectrum SmartFormula Report

Analysis Info

Analysis Name	D:\Data\Xiao\Dec 13 2022000004.d	Acquisition Date	2022-12-13 2:18:41 PM
Method	Xiao all 1.m	Operator	x
Sample Name	TRV6027 HCl	Instrument	compact 8255754.20059
Comment			

Acquisition Parameter

Source Type	ESI	Ion Polarity	Positive	Set Nebulizer	0.5 Bar
Focus	Not active	Set Capillary	3500 V	Set Dry Heater	180 °C
Scan Begin	50 m/z	Set End Plate Offset	-500 V	Set Dry Gas	4.0 l/min
Scan End	1500 m/z	Set Charging Voltage	2000 V	Set Divert Valve	Source
		Set Corona	0 nA	Set APCI Heater	0 °C

Meas. m/z	Ion Formula	m/z	err [ppm]
224.1083	C12H15FNO2	224.1081	-0.6

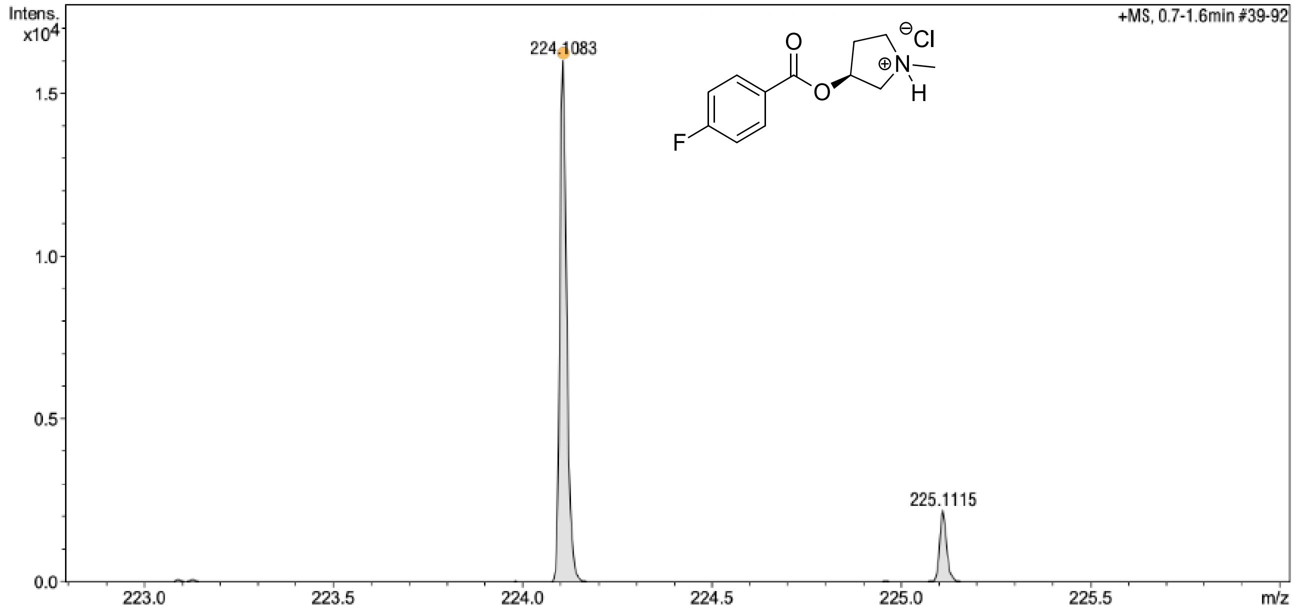

(26) (*3S*)-3-(*p*-Fluorobenzoyloxy)-1-methyl-1-pyrrolidinium Chloride HPLC. Note: Noise from the injection has a retention time from 1.6 min until 2.5 min. The compound of interest has a retention time of 4.118 min.

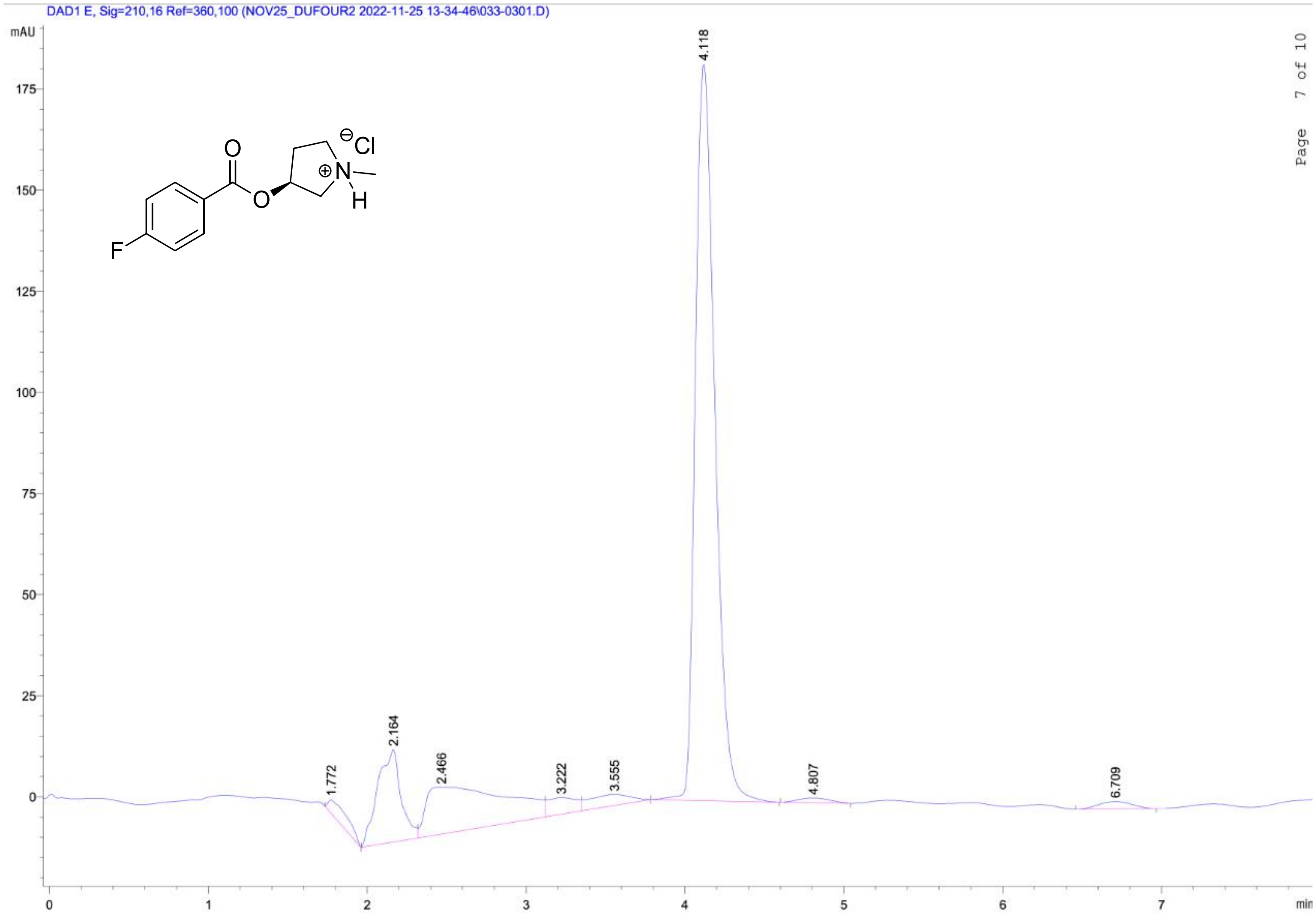

(27) ((*3R*)-1-Methylpyrrolidin-3-yl) *p*-Iodobenzenecarbothioate Oxylate

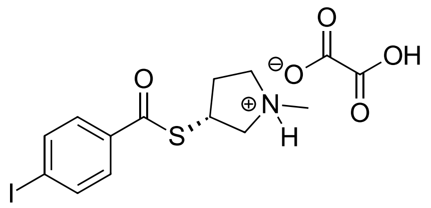

Synthesis: **(*3S*)-3-(Mesyloxy)-1-methylpyrrolidine**: (*S*)-(-)-1-Methyl-3-pyrrolidinol (6.00 mL, 54.6 mmol) was dissolved in anhydrous DCM (130 mL) and cooled to 0°C with an ice bath. Triethylamine (11.4 mL, 81.9 mmol) and methanesulfonyl chloride (4.90 mL, 62.8 mmol) were added dropwise at 0°C. The ice bath was removed after 5 mins, and the reaction was stirred overnight. Brine (50 mL) was added to the reaction and the layers were separated. The aqueous layer was extracted with dichloromethane (3 × 15 mL). The combined organic layers were dried over Na_2SO_3. Gravity filtered, and the filtrate was concentrated in vacuo to produce a yellow oil (8.87 g, 91%). Product confirmed by ^{1}H NMR and the crude material was immediately carried through to next step without further purification.

(*3R*)-S-(1-Methylpyrrolidin-3-yl) ethanethioate: (*3S*)-3-(Mesyloxy)-1-methylpyrrolidine (6.99 g, 39.0 mmol), was dissolved in anhydrous THF (130 mL) under an argon atmosphere. 18-crown-6-ether (15.5 g, 58.5 mmol) was added followed by potassium thioacetate (6.68 g, 58.5 mmol), added in 3 portions. Once all potassium thioacetate had gone into solution, the reaction was heated to 30°C and left overnight. The reaction was filtered then diluted with water (50 mL) and extracted with EtOAc (3 × 15 mL). The organic layer was washed with water (3 × 20 mL) and brine (3 × 20 mL), dried over Na_2SO_3 and concentrated *in vacuo* to produce a red oil. The crude material was then distilled under vacuum (1 mm Hg) at 100 °C to give a yellow oil (4.45 g, 72%). Product confirmed by ^{1}H NMR and the crude material was immediately carried through to next step without further purification.

(*R*)-1-Methyl-3-pyrrolidinethiol: (*3R*)-S-(1-Methylpyrrolidin-3-yl) ethanethioate (0.951 g, 5.97 mmol) was dissolved in MeOH (30 mL) and sodium methoxide (0.333 g, 5.97 mmol) was added to the stirring solution. After all starting material was consumed by TLC using a $KMnO_4$ stain, MeOH was removed *in vacuo* and a solvent swap with DCM was done and used in the next step immediately.

((*3R*)-1-Methylpyrrolidin-3-yl) *p*-iodobenzenethioate: (*R*)-1-Methyl-3-pyrrolidinethiol was dissolved in DCM (20mL) and cooled to 0 °C in an ice bath. 4-Iodobenzoyl chloride (1.59 g, 5.97 mmol) was added to the reaction flask followed by triethylamine (1.66 mL, 11.9 mmol). The ice bath was removed after 30 mins, reaction was warmed to room temperature and stirred for 16 hrs. Once the reaction was complete, the solvent was removed *in vacuo.* The crude material was washed with diethyl ether, filtered to remove all precipitate, and the filtrate was concentrated *in vacuo* to give an orange solid, (1.63 g, 79%). This material was used carried through immediately without further purification.

((*3R*)-1-Methylpyrrolidin-3-yl) *p*-Iodobenzenecarbothioate Oxylate: ((*3R*)-1-Methylpyrrolidin-3-yl) *p*-iodobenzenethioate (1.14 g, 3.28 mmol) was completely dissolved in isopropanol (63 mL). With stirring, oxalic acid (0.296 g, 3.28 mmol) was added and left to react for 3 hrs. The solution was concentrated *in vacuo* to give a white solid and immediately recrystallized with isopropanol. For further purification, this material was recrystallized with methanol twice to give ((*3R*)-1-methylpyrrolidin-3-yl) *p*-iodobenzenecarbothioate Oxylate as a white solid (0.610 g, 42%)

Spectroscopic Data: $MP_{(isopropanol)}$: 197–200 °C; IR(ATR): 3042, 2838, 2705, 2574, 1658, 1561, 909, 706 cm^{-1}; ^{1}H NMR (400 MHz, DMSO-*d6*) δ 7.98-7.96 (m, 2H), 7.67-7.65 (m, 2H), 4.26-4.19 (m, 1H), 4.06 (br s, 2H, overlapping with H_2O in DMSO); 3.69 (dd, J = 11.7, 8.0 Hz, 1H), 3.35-3.29 (m, 1H), 3.24-3.18 (m, 2H), 2.76 (s, 3H), 2.59-2.52 (m, 1H), 2.06-1.97 (m, 1H); ^{13}C NMR (100.7 MHz, DMSO-*d6*) δ 190.3 (0), 164.2 (0), 138.2 (1), 135.1 (0), 128.5 (1), 103.0 (0), 59.8 (2), 54.2 (2), 40.5 (3), 38.6 (1), 30.0 (0); HRMS (ESI^{+}): calculated for $C_{12}H_{15}INOS^{+}$: 347.9913 amu; found for $C_{12}H_{15}INOS^{+}$: 347.9910 amu; HPLC purity at 230nm (75% CH_3CN : 10% CH_3OH : 15% aqueous triethylamine [0.1% triethylamine in H_2O], retention time 8.331 mins): 99.5 %.

(27) ((*3R*)-1-Methylpyrrolidin-3-yl) *p*-Iodobenzenecarbothioate Oxylate IR(ATR)

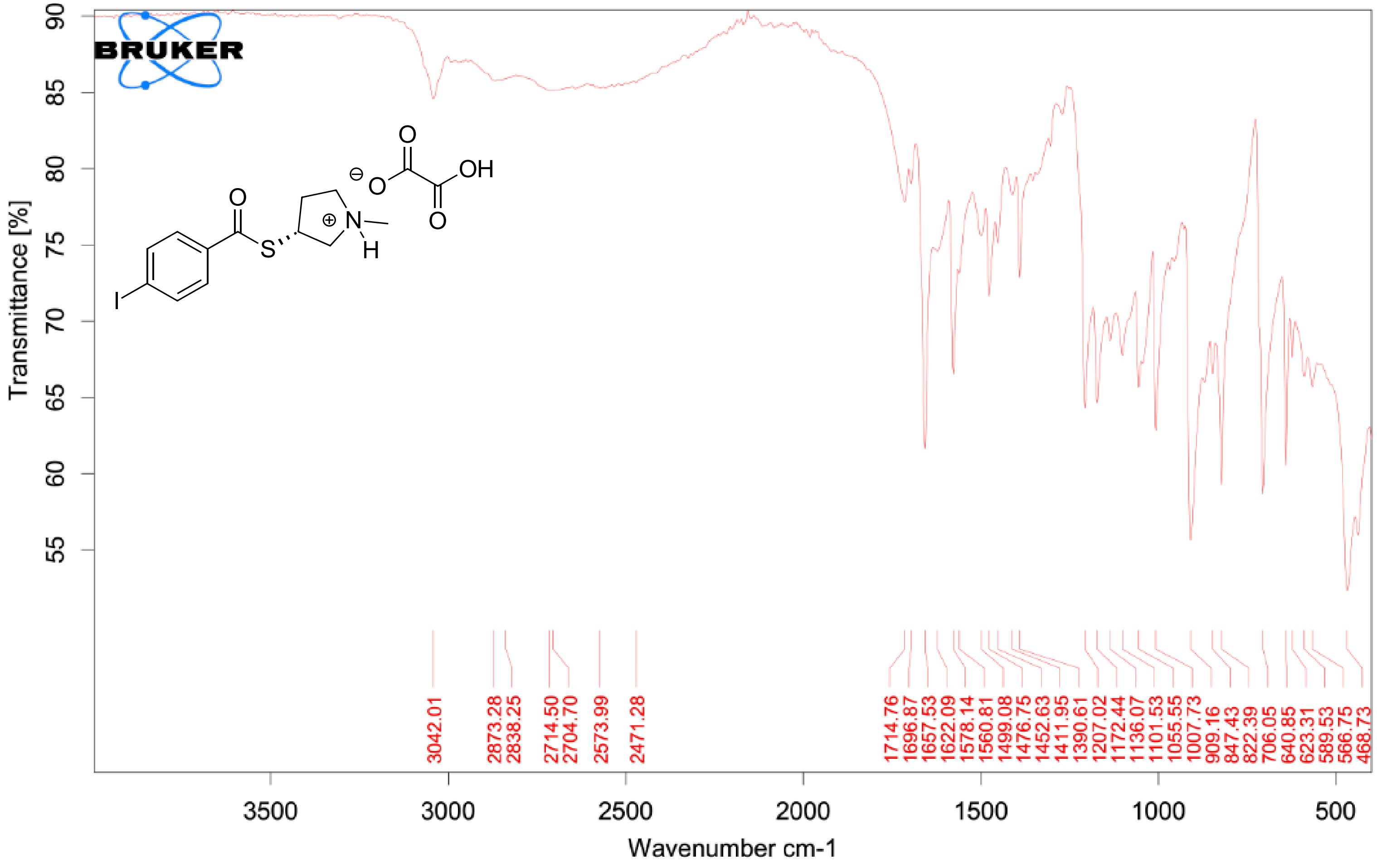

(27) ((*3R*)-1-Methylpyrrolidin-3-yl) *p*-Iodobenzenecarbothioate Oxylate ^{1}HNMR 400 MHz (DMSO-*d6*)

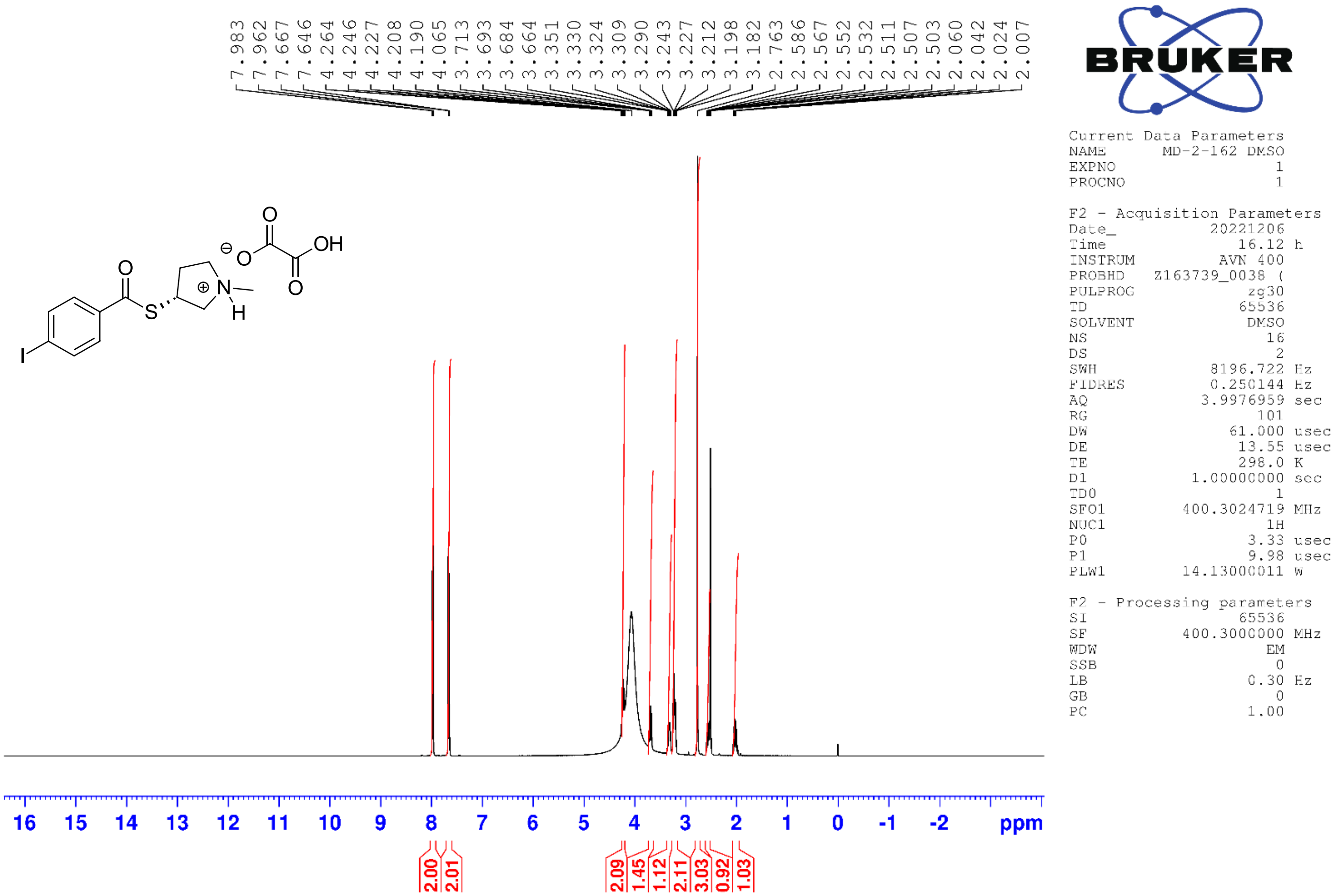

(27) ((*3R*)-1-Methylpyrrolidin-3-yl) *p*-Iodobenzenecarbothioate Oxylate ^{13}CNMR 100 MHz (DMSO-*d6*)

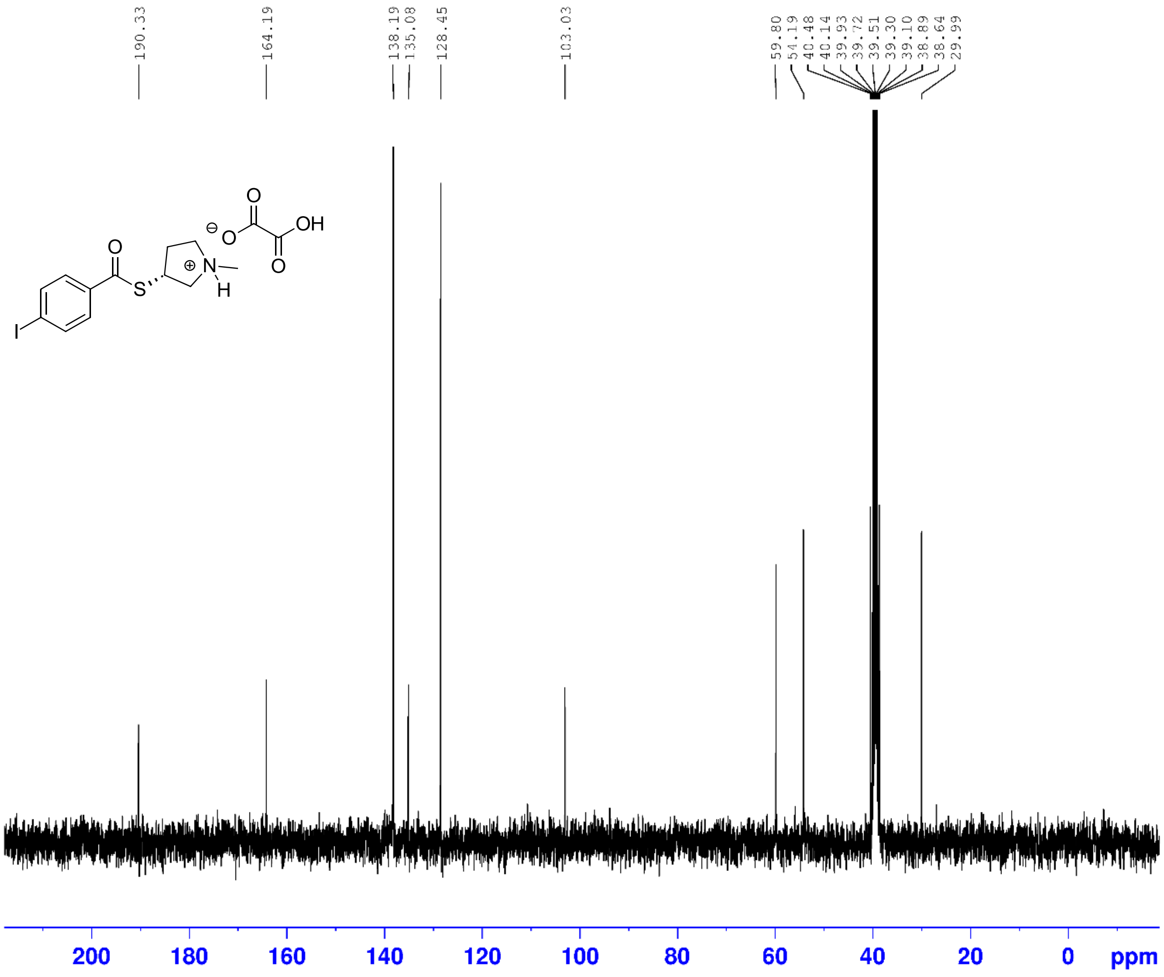

Current Data Parameters
NAME MD-2-162 DMSO
EXPNO 2
PROCNO 1

F2 - Acquisition Parameters
Date_ 20221207
Time 3.48 h
INSTRUM AVN 400
PROBHD Z163739_0038 (
PULPROG udeft
TD 21424
SOLVENT DMSO
NS 840
DS 8
SWH 23809.523 Hz
FIDRES 2.222696 Hz
AQ 0.4499040 sec
RG 8.37054
DW 21.000 usec
DE 6.50 usec
TE 298.0 K
D1 4.00000000 sec
D12 0.00002000 sec
D20 20.00000000 sec
TD0 1
SFO1 100.6655806 MHz
NUC1 13C
P1 10.00 usec
P13 2000.00 usec
P26 500.00 usec
PLW1 58.86999893 W
SPNAM[5] Crp60comp.4
SPOAL5 0.500
SPOFFS5 0 Hz
SPW5 8.99470043 W
SPNAM[8] Crp60,0.5,20.1
SPOAL8 0.500
SPOFFS8 0 Hz
SPW8 8.99470043 W
SFO2 400.3016012 MHz
NUC2 1H
CPDPRG[2 waltz65
PCPD2 90.00 usec
PLW2 14.13000011 W
PLW12 0.17375000 W

F2 - Processing parameters
SI 131072
SF 100.6555612 MHz
WDW EM
SSB 0
LB 2.00 Hz
GB 0
PC 1.40

(27) ((*3R*)-1-Methylpyrrolidin-3-yl) *p*-Iodobenzenecarbothioate Oxylate High Resolution Mass Spectrum (ESI, positive mode)

Mass Spectrum SmartFormula Report

Analysis Info

Analysis Name	D:\Data\Xiao\Dec 13 2022000002.d	Acquisition Date	2022-12-13 2:01:53 PM
Method	Xiao all 1.m	Operator	x
Sample Name	TRV6029 Oxalate	Instrument	compact 8255754.20059
Comment			

Acquisition Parameter

Source Type	ESI	Ion Polarity	Positive	Set Nebulizer	0.5 Bar
Focus	Not active	Set Capillary	3500 V	Set Dry Heater	180 °C
Scan Begin	50 m/z	Set End Plate Offset	-500 V	Set Dry Gas	4.0 l/min
Scan End	1500 m/z	Set Charging Voltage	2000 V	Set Divert Valve	Source
		Set Corona	0 nA	Set APCI Heater	0 °C

Meas. m/z	Ion Formula	m/z	err [ppm]
347.9910	C12H15INOS	347.9914	0.9

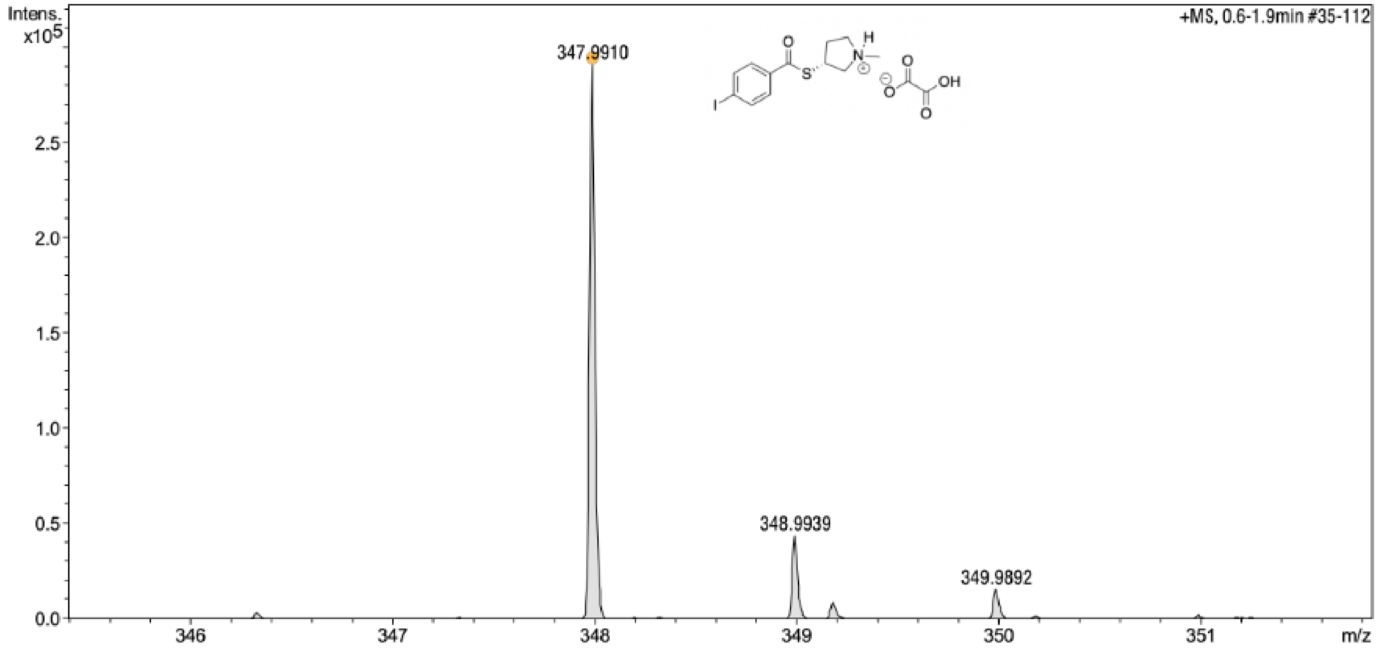

(27) ((*3R*)-1-Methylpyrrolidin-3-yl) *p*-Iodobenzenecarbothioate Oxylate HPLC. Note: Noise from the injection has a retention time from 1.6 min until 2.5 min. The compound of interest has a retention time of 8.331 min.

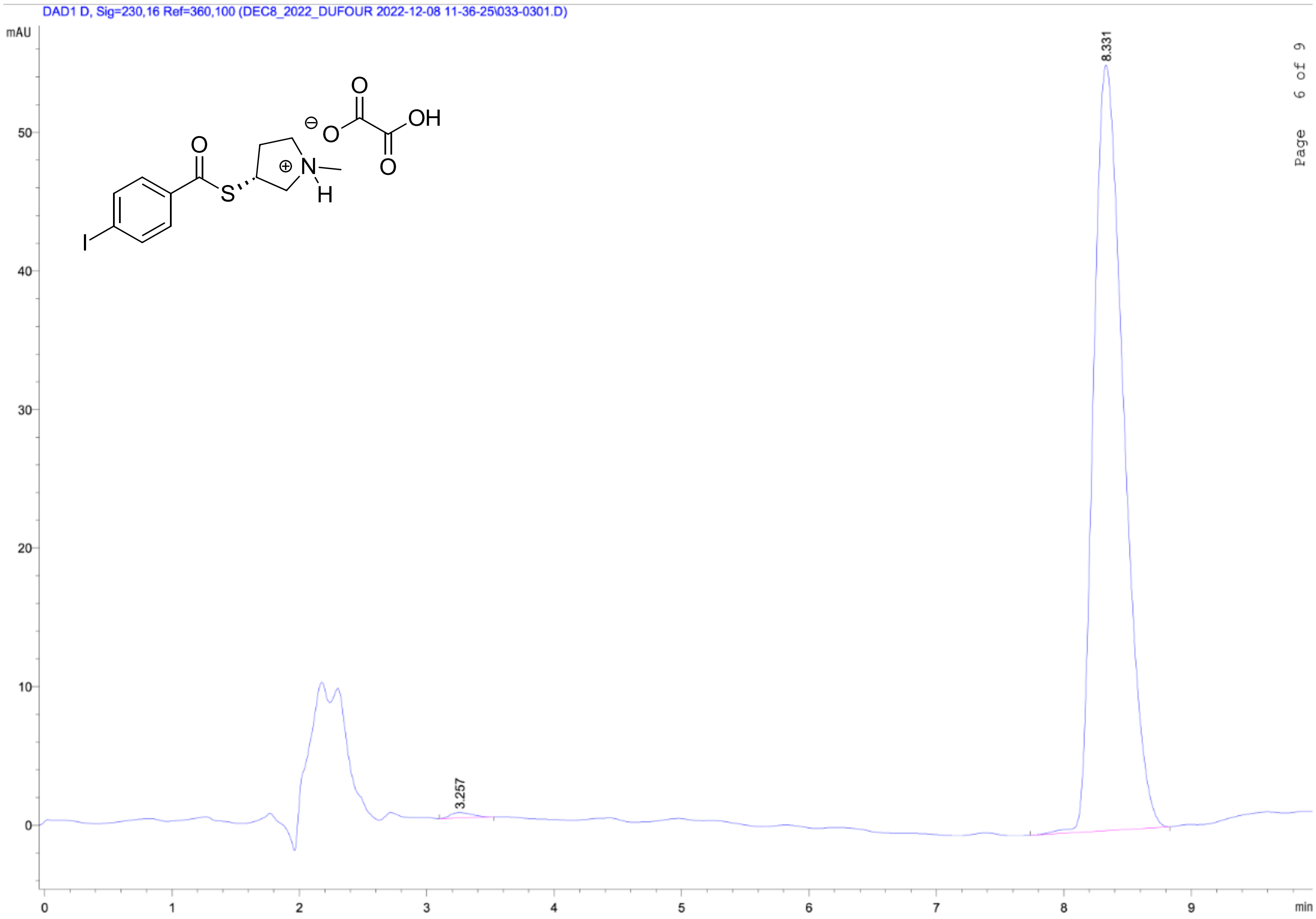

(28) *(3R)*-3-(*p*-Iodobenzoyloxy)-1-methyl-1-pyrrolidinium chloride

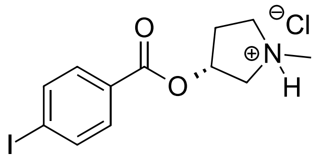

Synthesis: **(*3R*)-1-Methyl-3-pyrrolidinyl *p*-iodobenzoate**: 4-Iodobenzoyl chloride (0.799 g, 3.00 mmol), triethylamine (1.05 mL, 7.50 mmol) and 4-dimethylaminopyridine (DMAP) (0.0659 g, 0.540 mmol) were dissolved in anhydrous dichloromethane (20.0 mL) under an argon atmosphere. (R)-(-)-1-Methyl-3-pyrrolidinol (0.329 mL, 3.00 mmol) was added at 0°C. The ice bath was removed after 5 mins, and the reaction was stirred for 3hrs. The reaction was quenched with brine (20mL), extracted with DCM (3×15 mL) and the combined organic layers were dried over Na_2SO_3. The solvent was removed was *in vacuo* to produce a pink solid. The crude mixture was purified by silica gel column chromatography (5% MeOH/DCM) to produce a pink solid, (*3R*)-1-methyl-3-pyrrolidinyl *p*-iodobenzoate (0.409 g, 41%). The analytical data was similar to previously published data.[7]

***(3R)*-3-(*p*-Iodobenzoyloxy)-1-methyl-1-pyrrolidinium chloride**: (*3R*)-1-Methyl-3-pyrrolidinyl *p*-iodobenzoate (0.257 g, 0.776 mmol) was dissolved in anhydrous diethyl ether (16 mL) under an argon atmosphere. HCl in diethyl ether (1 N - 1.2 mL, 1.16 mmol) was added dropwise resulting in the immediate formation of a white precipitate. The reaction was stirred for an additional 60 mins at room temperature. The precipitate was collected via suction filtration, washing with Et_2O (2 x 10 mL), to afford *(3R)*-3-(*p*-iodobenzoyloxy)-1-methyl-1-pyrrolidinium chloride (0.150 mg, 53 % yield) as a white solid. Spectroscopic data: $MP_{(diethyl\ ether)}$: 243-245 ^oC; IR (ATR) 3012, 3003, 2922, 2894, 2574, 2474, 1714, 1585, 1269, 1102, 753; ^1H NMR (400 MHz, D_2O) δ 7.92-7.90 (m, 2H), 7.73-7.71 (m, 2H), 5.68-5.66 (m, 1H), 3.80-3.70 (m, 3H), 3.51 (br app s, 1H), 3.04 (s, 3H), 2.70-2.60 (m, 1H), 2.44-2.38 (m, 1H); ^{13}C NMR (100.7 MHz, D_2O) δ 167.7, 138.7, 131.5, 128.9, 102.3, 74.7, 61.1, 55.2, 42.0, 31.1; LRMS (ESI^+): 332.0 (M^+); HRMS (ESI^+): calculated for $C_{12}H_{15}INO_2^+$: 332.0142; found for $C_{12}H_{15}INO_2^+$: 332.0133; HPLC purity at 254 nm (75% CH_3CN : 10% CH_3OH : 15% aqueous triethylamine [0.1% triethylamine in H_2O], retention time 5.755 mins): >99%.

(28) *(3R)*-3-(*p*-Iodobenzoyloxy)-1-methyl-1-pyrrolidinium Chloride IR(ATR)

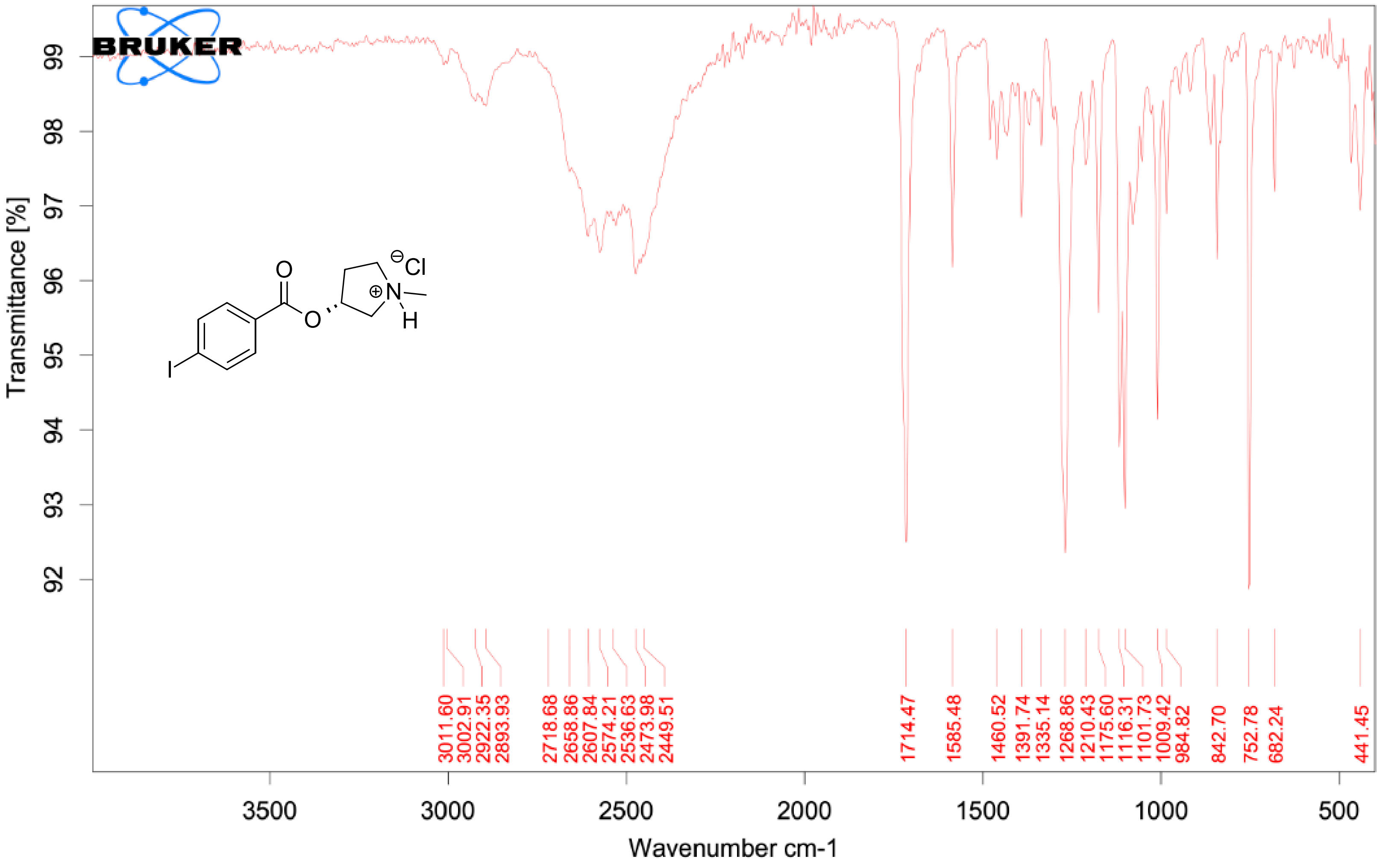

(28) *(3R)*-3-(*p*-Iodobenzoyloxy)-1-methyl-1-pyrrolidinium Chloride ^{1}H NMR 400 MHz (D_2O)

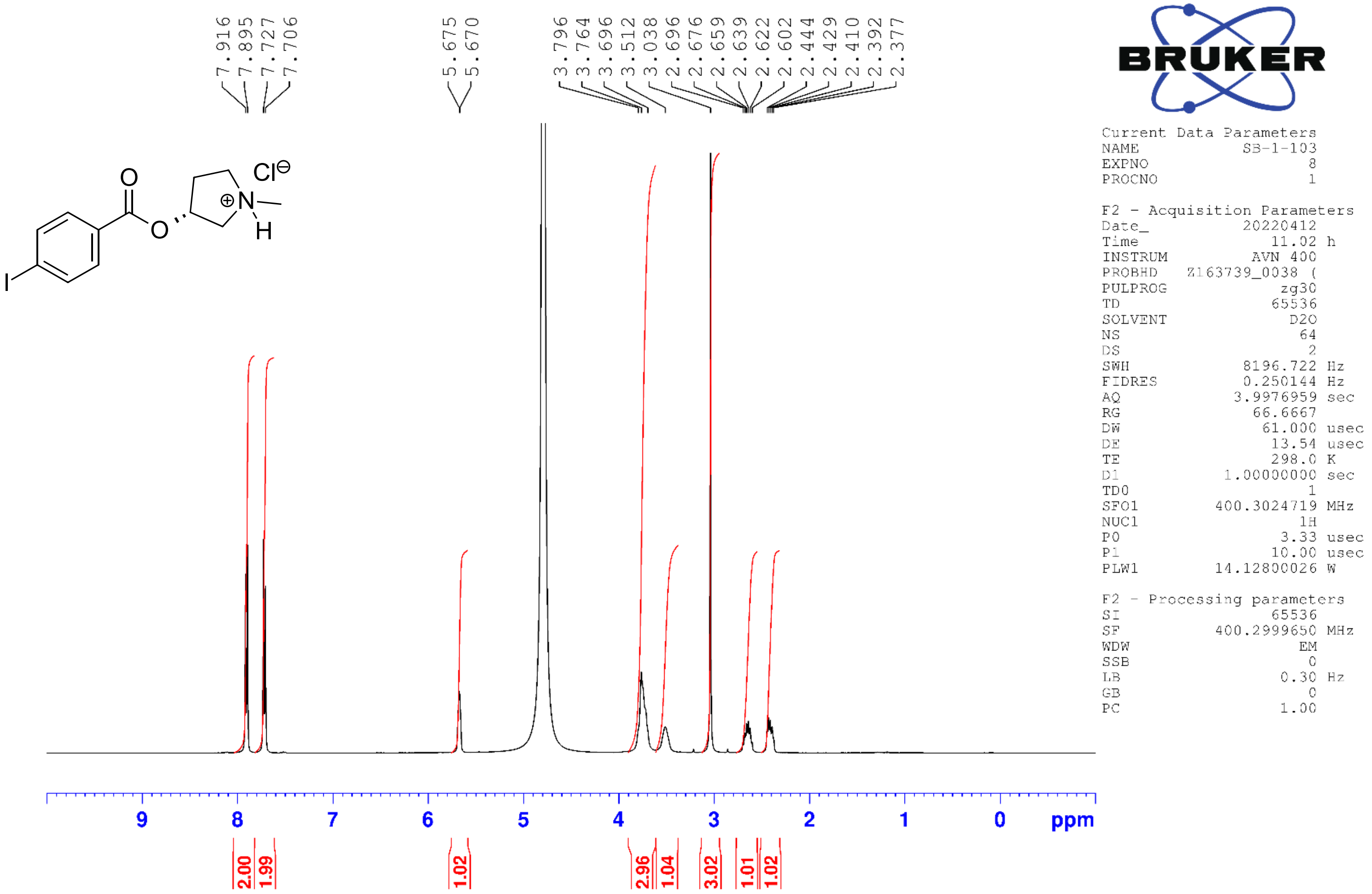

(28) *(3R)*-3-(*p*-Iodobenzoyloxy)-1-methyl-1-pyrrolidinium Chloride ^{13}C NMR 100 MHz (D_2O)

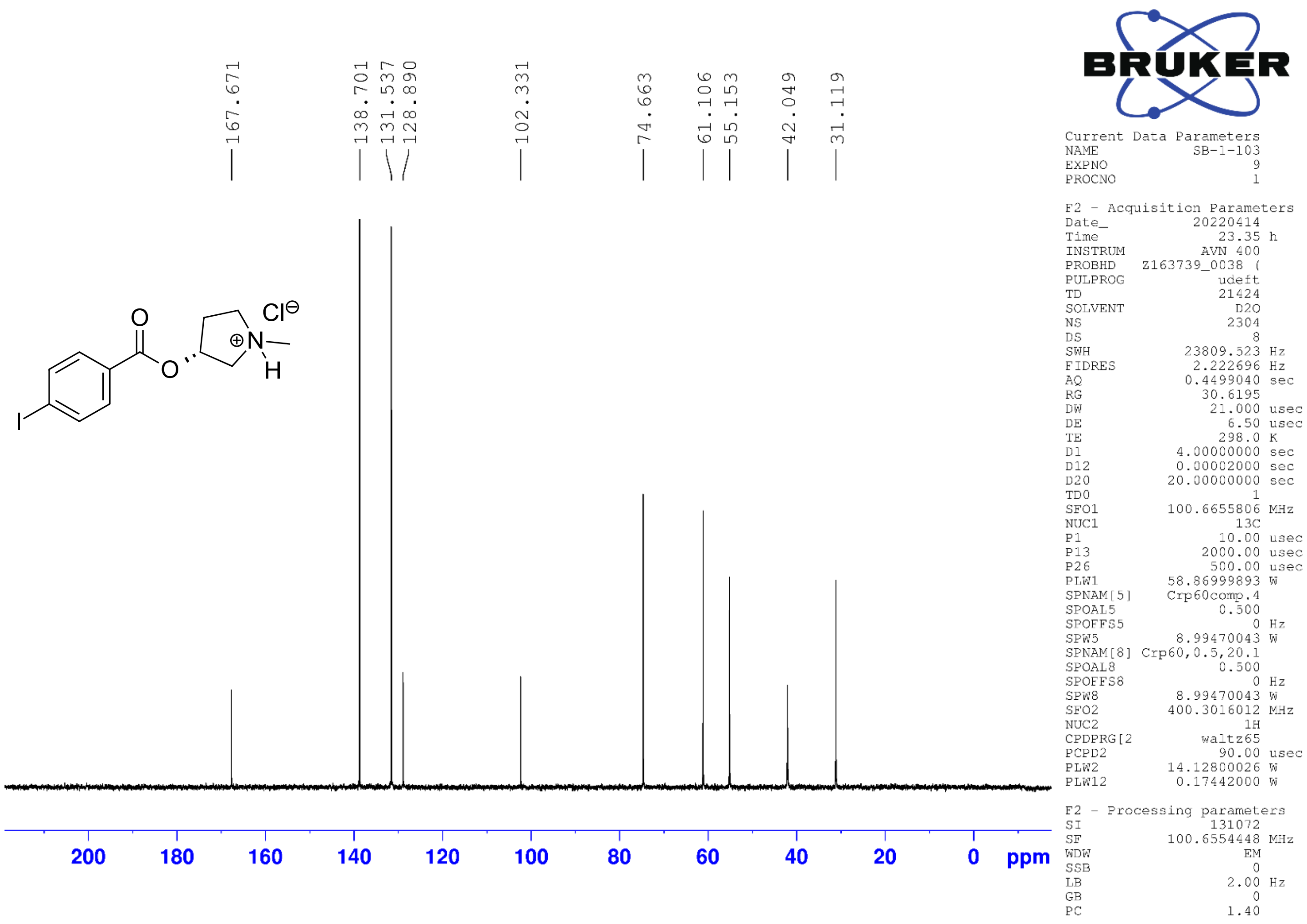

(28) *(3R)*-3-(*p*-Iodobenzoyloxy)-1-methyl-1-pyrrolidinium Chloride High Resolution Mass Spectrum (ESI, positive mode)

Mass Spectrum SmartFormula Report

Analysis Info

Analysis Name	D:\Data\Xiao\July 06 2022000014.d	Acquisition Date	7/6/2022 3:12:40 PM
Method	Xiao all 1.m	Operator	x
Sample Name	TRV 6003 HCl	Instrument	compact 8255754.20059
Comment			

Acquisition Parameter

Source Type	ESI	Ion Polarity	Positive	Set Nebulizer	0.5 Bar
Focus	Not active	Set Capillary	3500 V	Set Dry Heater	181 °C
Scan Begin	50 m/z	Set End Plate Offset	-500 V	Set Dry Gas	4.0 l/min
Scan End	1500 m/z	Set Charging Voltage	2000 V	Set Divert Valve	Source
		Set Corona	0 nA	Set APCI Heater	0 °C

Meas. m/z	Ion Formula	m/z	err [ppm]
332.0133	C12H15INO2	332.0142	2.7

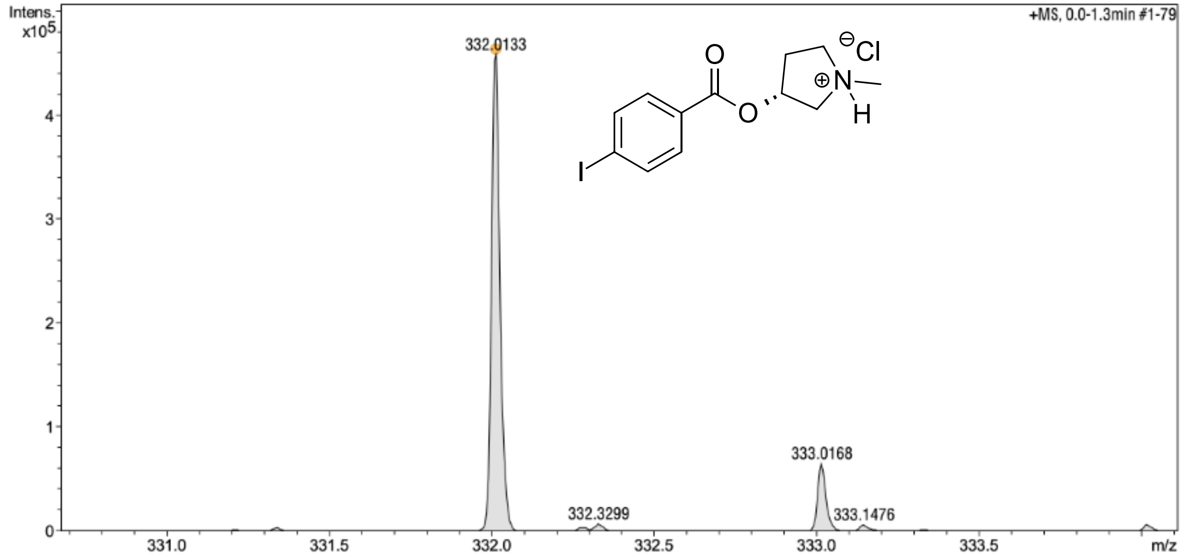

July 06 2022000014.d

Bruker Compass DataAnalysis 4.3 printed: 7/6/2022 3:16:03 PM by: x Page 1 of 1

(28) *(3R)*-3-(*p*-Iodobenzoyloxy)-1-methyl-1-pyrrolidinium Chloride HPLC

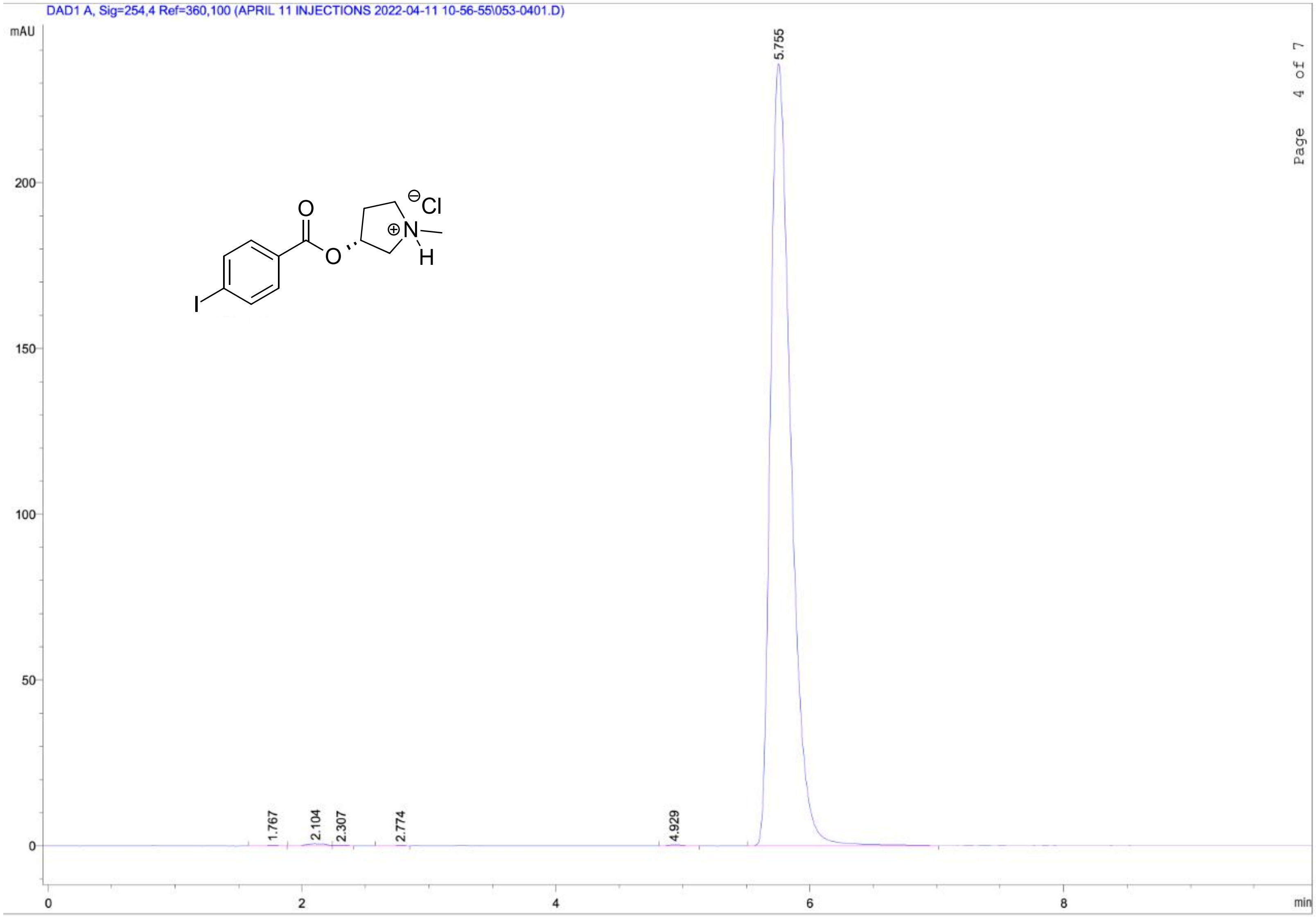

(29) ((*3R*)-1-Methylpyrrolidin-3-yl) *p*-Fluorobenzenecarbothioate Oxylate

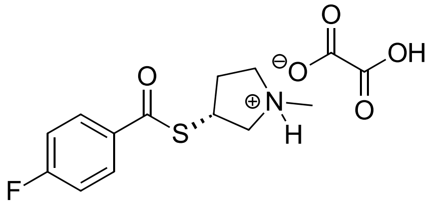

Synthesis: **(*3S*)-3-(Mesyloxy)-1-methylpyrrolidine:** (*3S*)-(-)-1-Methyl-3-pyrrolidinol (6.0 mL, 54.6 mmol) was dissolved in anhydrous dichloromethane (130 mL) and cooled to 0°C with an ice bath. Triethylamine (11.4 mL, 81.9 mmol) and methanesulfonyl chloride (4.9 mL, 62.8 mmol) were added dropwise at 0°C. The ice bath was removed after 5 mins, and the reaction was stirred overnight. Brine (50 mL) was added to the reaction and the layers were separated. The aqueous layer was extracted with dichloromethane (3 × 15 mL). The combined organic layers were dried over Na_2SO_3, gravity filtered, and the filtrate was concentrated *in vacuo* to produce (*3S*)-3-(mesyloxy)-1-methylpyrrolidine as a yellow oil (8.87 g, 91%). Product was confirmed by ^{1}H NMR and the crude material was immediately carried through to next step without further purification.

(*3R*)-S-(1-Methylpyrrolidin-3-yl) ethanethioate: (*3S*)-3-(Mesyloxy)-1-methylpyrrolidine (6.99 g, 39.0 mmol) was dissolved in anhydrous THF (130 mL) under an argon atmosphere. 18-crown-6-ether (15.5 g, 58.5 mmol) was added followed by potassium thioacetate (6.68 g, 58.5 mmol), added in 3 portions. Once all potassium thioacetate had gone into solution, the reaction was heated to 30°C and left overnight. The reaction was filtered, the filtrate diluted with water (50 mL) and extracted with EtOAc (3 × 15 mL). The combined organic layers were washed with water (3 × 20 mL) and brine (3 × 20 mL), dried over Na_2SO_3, gravity filtered, and concentrated *in vacuo* to produce (*3R*)-S-(1-methylpyrrolidin-3-yl) ethanethioate as a red oil. The crude material was then distilled under vacuum (1 mm Hg) at 100-101°C to give a yellow oil (4.45 g, 72%). Product was confirmed by ^{1}H NMR and the material was immediately carried through to next step without further purification.

(*S*)-1-Methyl-3-pyrrolidinethiol: (*3R*)-S-(1-Methylpyrrolidin-3-yl) ethanethioate (0.950 g, 5.97 mmol) was dissolved in MeOH (30 mL) and sodium methoxide (0.332 g, 5.97 mmol) was added to the stirring solution. After all starting material was shown to be consumed by TLC experiment

the reaction was concentrated *in vacuo*. The resulting material was placed dichloromethane (20 mL) and concentrated *in vacuo*. This process was repeated and the material was immediately used in the next step.

((*3R*)-1-Methylpyrrolidin-3-yl) *p*-fluorobenzenecarbothioate oxylate: The (*S*)-1-methyl-3-pyrrolidinethiol was dissolved in dichloromethane (20 mL) and cooled to 0°C using an ice bath. 4-fluorobenzoyl chloride (0.716 mL, 5.97 mmol) was added followed by triethylamine (1.66 mL, 11.9 mmol). The ice bath was removed after 30 mins and warmed to room temperature. After all starting material was shown to be consumed by TLC experiment (developed using $KMnO_4$ stain), the solvent was removed *in vacuo.* The crude material was washed with diethyl ether, filtered to remove all precipitate, and the filtrate was concentrated *in vacuo* to give an orange oil (1.52 g). A portion of this orange oil (1.03 g, 4.30 mmol) was completely dissolved in isopropanol (43 mL). With stirring, oxalic acid (0.387 g, 4.30 mmol) was added and the mixture stirred for 3 hrs. The solution was concentrated *in vacuo* to give a white solid and immediately recrystallized with isopropanol four times to give ((*3R*)-1-methylpyrrolidin-3-yl) *p*-fluorobenzenecarbothioate oxylate as a white solid (0.539 g, 38%).

Spectroscopic Data: $MP_{(isopropanol)}$: 134–137 °C; IR(ATR): 3035, 2964, 2847, 2716, 1655, 1598, 1206, 913, 841 cm^{-1}; ^{1}H NMR (400 MHz, DMSO-*d6*) δ 8.00-7.97 (m, 2H), 7.43-7.39 (m, 2H), 4.24 (pent, J = 7.5 Hz, 1H), 3.74 (dd, J = 12.0, 8.3 Hz, 1H), 3.39-3.34 (m, 1H), 3.30-3.23 (m, 2H), 2.79 (s, 3H), 2.60-2.53 (m, 1H), 2.08-1.99 (m, 1H); ^{13}C NMR (100.7 MHz, DMSO-*d6*) δ 189.4 (0), 165.5 (d, $^{1}J_{CF}$ = 253.3 Hz, 0), 164.5 (1), 132.47 (d, $^{4}J_{CF}$ = 2.6 Hz, 0), 129.9 (d, $^{3}J_{CF}$ = 9.8 Hz, 1), 116.4 (d, $^{2}J_{CF}$ = 22.4 Hz, 1), 59.6 (2), 54.1 (2), 40.4 (3), 38.6 (1), 30.0 (2); ^{19}F NMR (376.6 MHz, DMSO-*d6*) δ 104.1; HRMS (ESI^{+}): calculated for $C_{12}H_{15}FNOS^{+}$: 240.0853 amu; found for $C_{12}H_{15}FNOS^{+}$: 240.0853 amu; HPLC purity at 254nm (75% CH_3CN : 10% CH_3OH : 15% aqueous triethylamine [0.1% triethylamine in H_2O], retention time 5.031 mins): 98.2%.

(29) ((*3R*)-1-Methylpyrrolidin-3-yl) *p*-Fluorobenzenecarbothioate Oxylate IR(ATR)

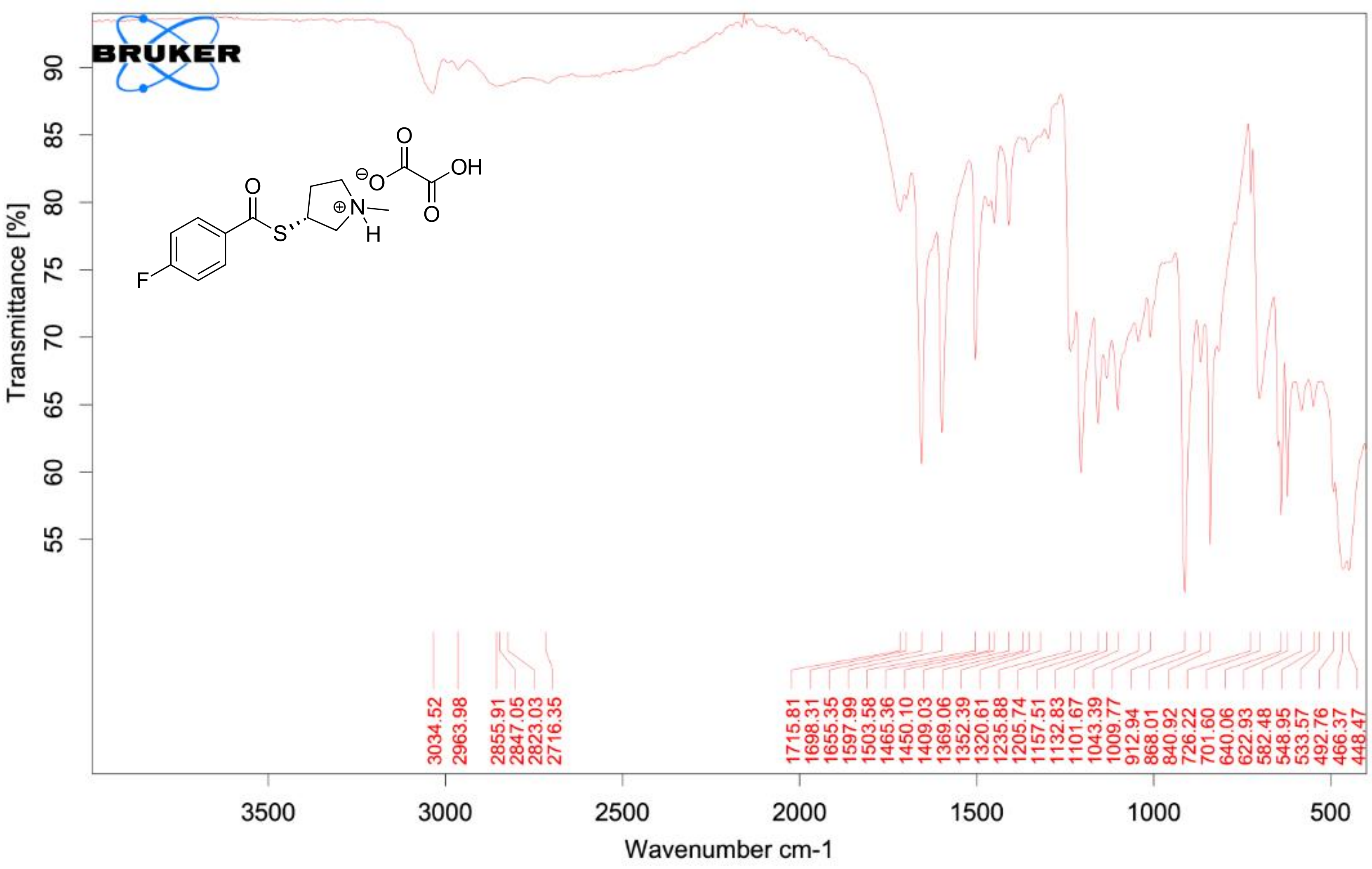

(29) ((*3R*)-1-Methylpyrrolidin-3-yl) *p*-Fluorobenzenecarbothioate Oxylate ^{1}HNMR 400 MHz (DMSO-*d6*)

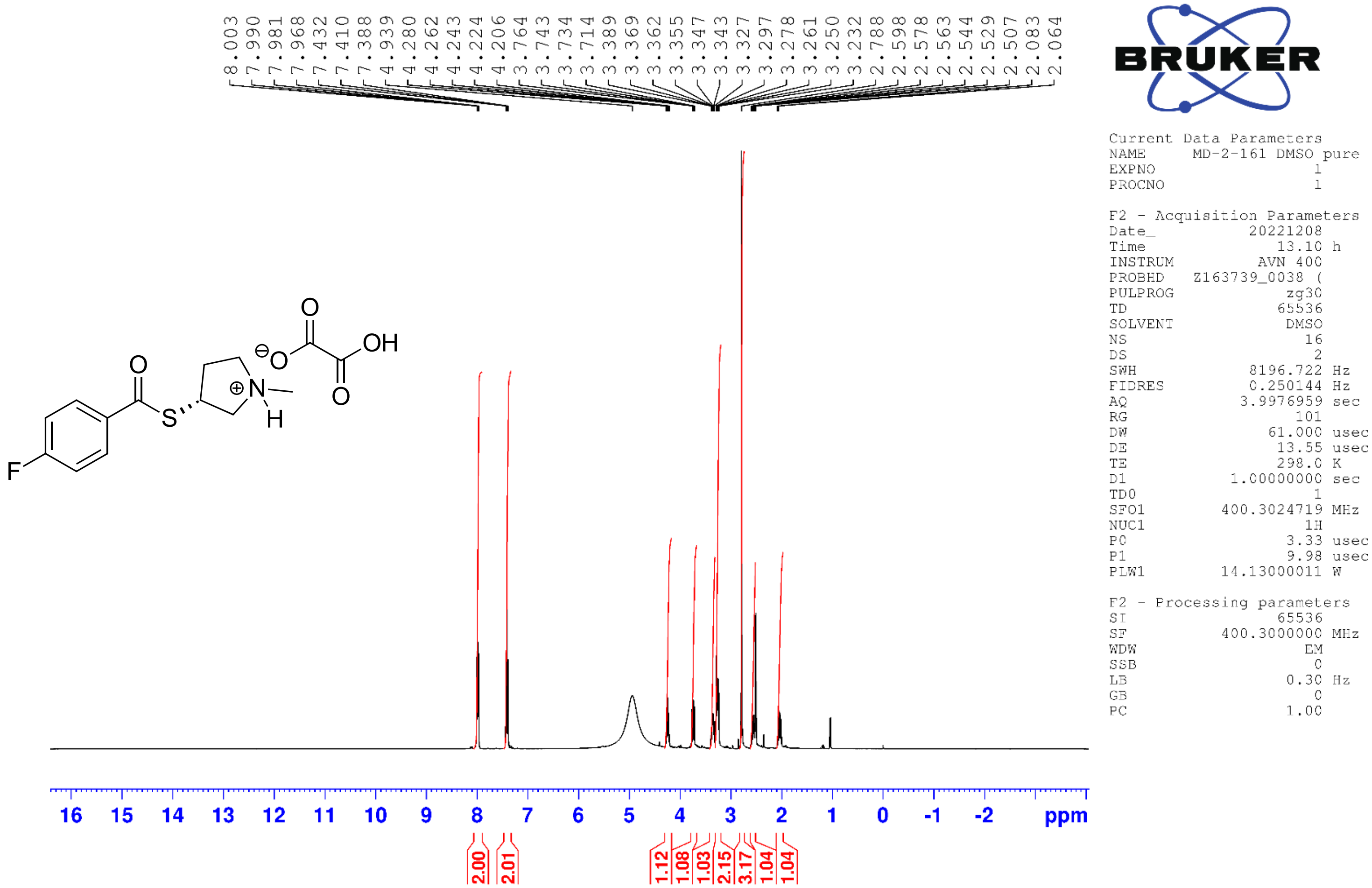

(29) ((*3R*)-1-Methylpyrrolidin-3-yl) *p*-Fluorobenzenecarbothioate Oxylate ^{13}CNMR 100 MHz (DMSO-*d6*)

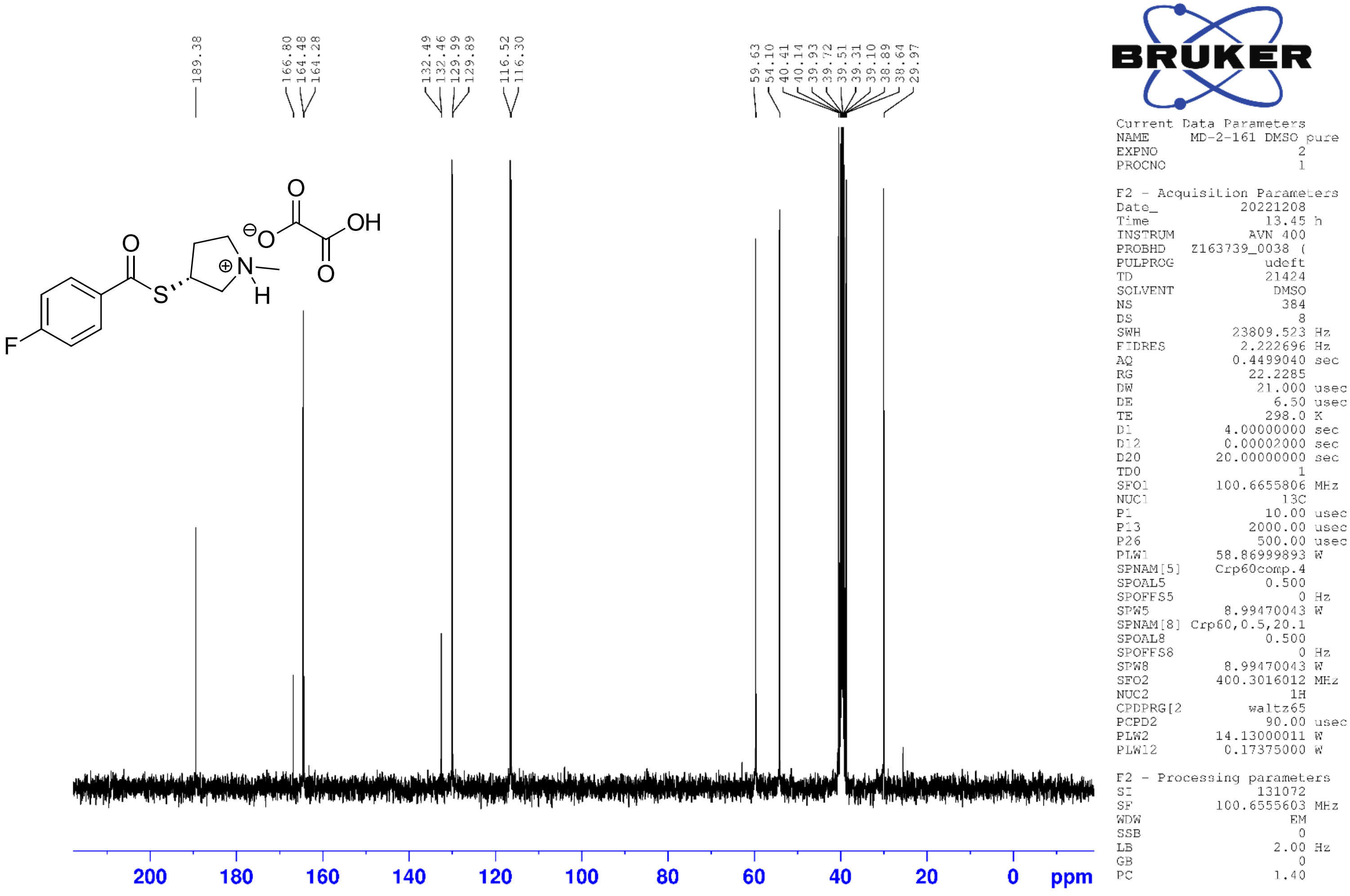

(29) ((*3R*)-1-Methylpyrrolidin-3-yl) *p*-Fluorobenzenecarbothioate Oxylate

Mass Spectrum SmartFormula Report

Analysis Info

Analysis Name	D:\Data\Xiao\Dec 13 2022000001.d	Acquisition Date	2022-12-13 1:52:45 PM	
Method	Xiao all 1.m	Operator	x	
Sample Name	TRV6030 Oxalate	Instrument	compact	8255754.20059
Comment				

Acquisition Parameter

Source Type	ESI	Ion Polarity	Positive	Set Nebulizer	0.5 Bar
Focus	Not active	Set Capillary	3500 V	Set Dry Heater	180 °C
Scan Begin	50 m/z	Set End Plate Offset	-500 V	Set Dry Gas	4.0 l/min
Scan End	1500 m/z	Set Charging Voltage	2000 V	Set Divert Valve	Source
		Set Corona	0 nA	Set APCI Heater	0 °C

Meas. m/z	Ion Formula	m/z	err [ppm]
240.0853	C12H15FNOS	240.0853	0.1

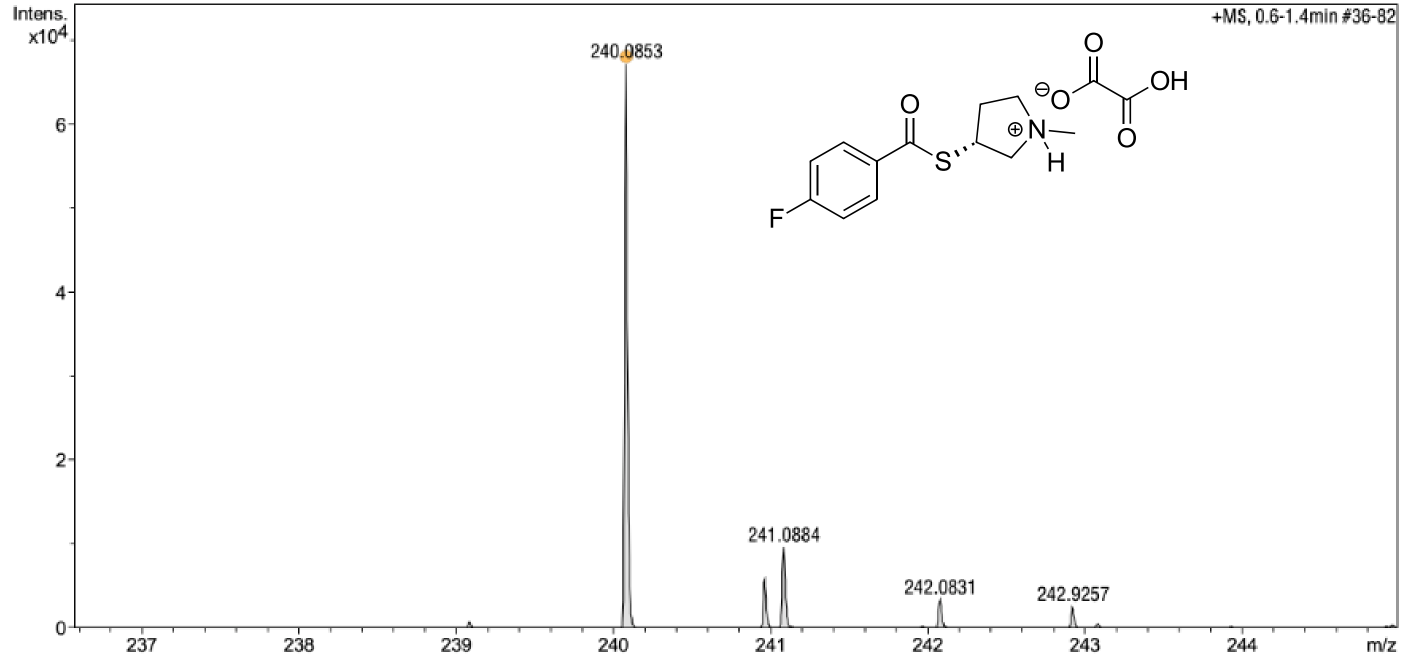

(29) ((*3R*)-1-Methylpyrrolidin-3-yl) *p*-Fluorobenzenecarbothioate Oxylate HPLC

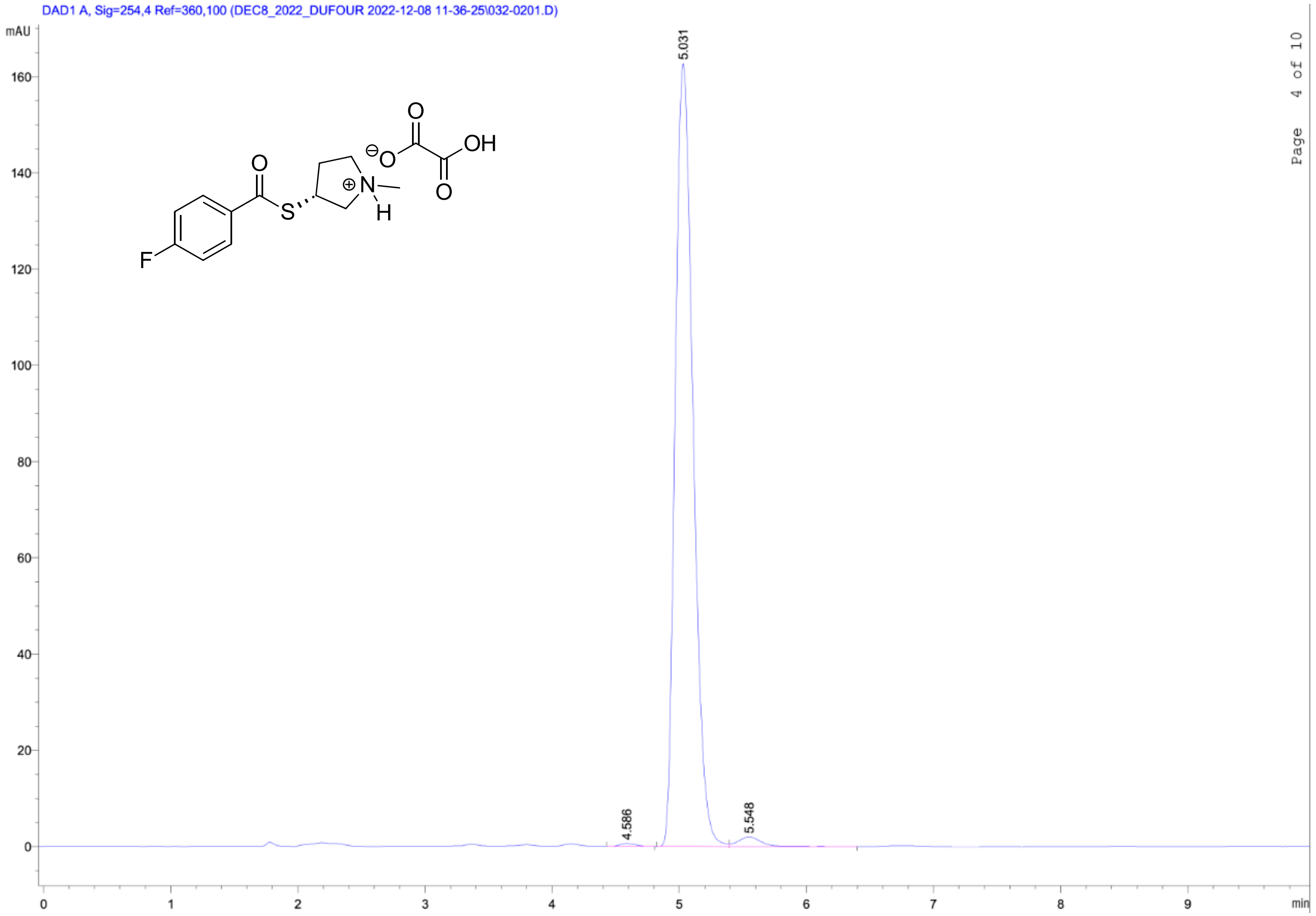

(30) (*3R*)-3-(*p*-Fluorobenzoyloxy)-1-methyl-1-pyrrolidinium Chloride

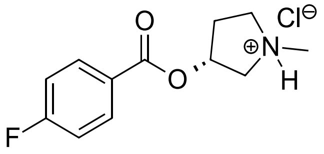

Synthesis: **(*3R*)-1-Methyl-3-pyrrolidinyl *p*-fluorobenzoate**: (*3R*)-(+)-1-Methyl-3-pyrrolidinol (0.659 mL, 6.00 mmol) was dissolved in anhydrous dichloromethane (20 mL) under an argon atmosphere and cooled to 0°C. To this solution, triethylamine (1.67 mL, 12.0 mmol) and 4-fluorobenzoyl chloride (0.720 g, 6.00 mmol). The ice bath was removed after 5 min, and the reaction was stirred for 24hrs. At this time water (20 mL) was added, layers were separated, and the aqueous layer was extracted with dichloromethane (3 × 15 mL). The combined organic layers were dried over Na_2SO_3, gravity filtered, and the filtrate was concentrated *in vacuo* to produce an orange oil. This crude material was purified by flash chromatography through silica gel (5% MeOH/DCM/1%TEA) to afford (*3R*)-1-methyl-3-pyrrolidinyl *p*-fluorobenzoate as an orange oil (1.09 g, 82%).

(*3R*)-3-(*p*-Fluorobenzoyloxy)-1-methyl-1-pyrrolidinium Chloride: (*3R*)-1-Methyl-3-pyrrolidinyl *p*-fluorobenzoate (0.248 g, 1.11 mmol) was dissolved in anhydrous diethyl ether (22 mL) under an argon atmosphere. HCl in diethyl ether (1 N - 1.70 mL, 1.67 mmol) was added dropwise and stirred for 3 hrs. The solvent was removed *in vacuo* resulting in a white solid. The compound was triturated three times with diethyl ether (20 mL), solvent was removed with a pipet and compound was dried to produce (*3R*)-3-(*p*-fluorobenzoyloxy)-1-methyl-1-pyrrolidinium chloride (0.148 g, 51%).

Spectroscopic Data: $MP_{(diethyl\ ether)}$: 144–149 °C; IR(ATR): 3112, 3051, 2955, 2603, 2569, 2455, 1718, 1602, 1270, 1114, 853, 685 cm^{-1}; ^{1}H NMR (400 MHz, DMSO-*d6*) δ 11.36 (br s, 1H),

8.16-8.15 (m, 2H), 7.39-7.35 (m, 2H), 5.54 (br s, 1H), 3.92-3.63 (m, 2H), 3.42 (br s, 1H), 3.14 (br s, 1H), 2.86 (s, 3H), 2.58-2.15 (m, 2H); ^{13}C NMR (100.7 MHz, DMSO-*d6*) δ 165.3 (d, $^{1}J_{C,F}$ = 251.8 Hz, 0), 164.2 (0), 132.6 (1), 125.8 (d, $^{4}J_{C,F}$ = 2.4 Hz, 0), 115.8 (d, $^{2}J_{C,F}$ = 22.2 Hz, 1), 73.3 (1), 59.4 (2), 53.1 (2), 40.4 (3), 30.4 (2); HRMS (ESI$^+$): calculated for $C_{12}H_{15}FNO_2^+$: 224.1081 amu; found for $C_{12}H_{15}FNO_2^+$: 224.1086 amu; HPLC purity at 230nm (75% CH_3CN : 10% CH_3OH : 15% aqueous triethylamine [0.1% triethylamine in H_2O], retention time 4.052 mins): 98.4%.

(30) (*3R*)-3-(*p*-Fluorobenzoyloxy)-1-methyl-1-pyrrolidinium Chloride IR(ATR)

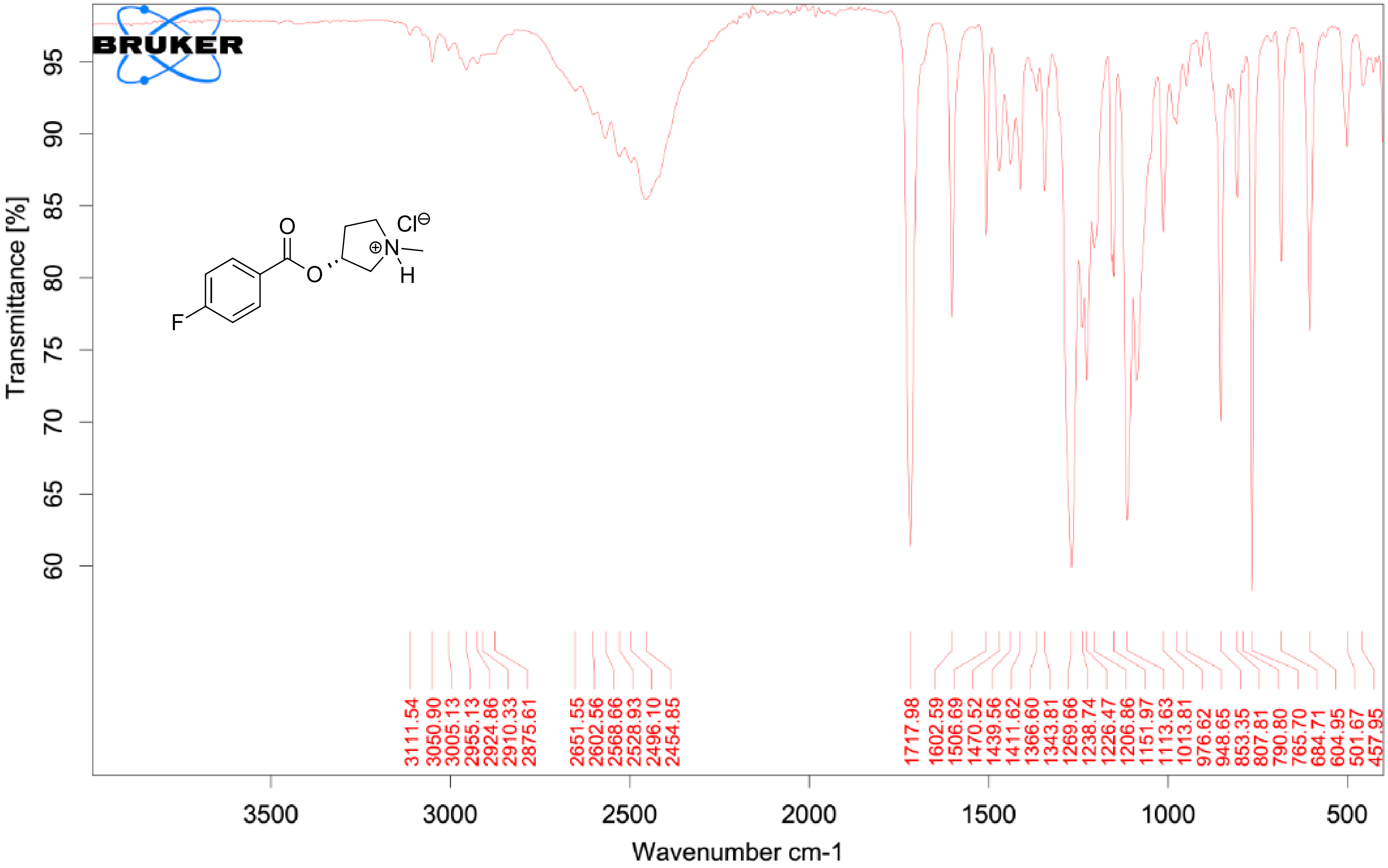

(30) (*3R*)-3-(*p*-Fluorobenzoyloxy)-1-methyl-1-pyrrolidinium Chloride ^{1}HNMR 400 MHz (DMSO-*d6*)

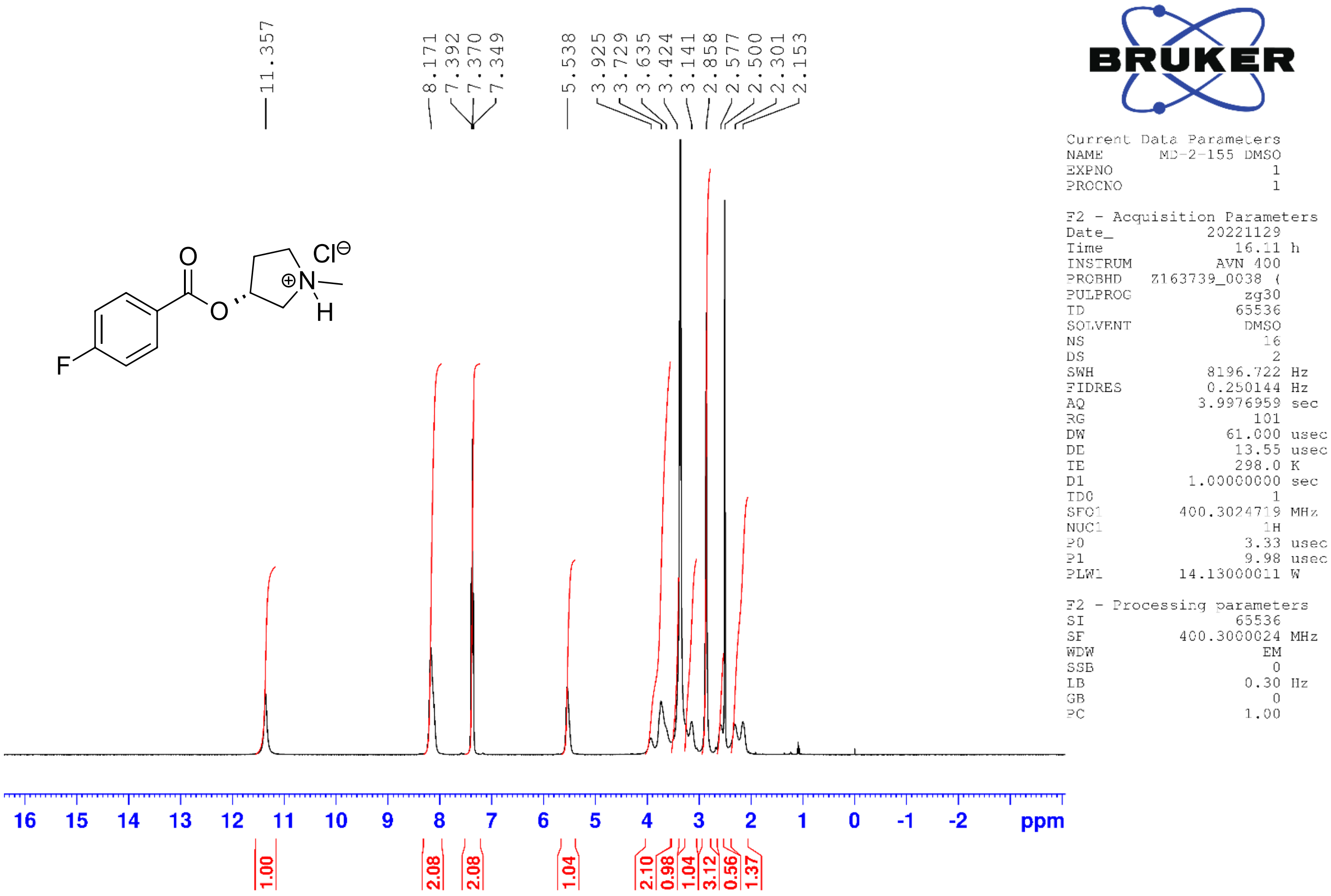

(30) (*3R*)-3-(*p*-Fluorobenzoyloxy)-1-methyl-1-pyrrolidinium Chloride ^{13}CNMR 100 MHz (DMSO-*d6*)

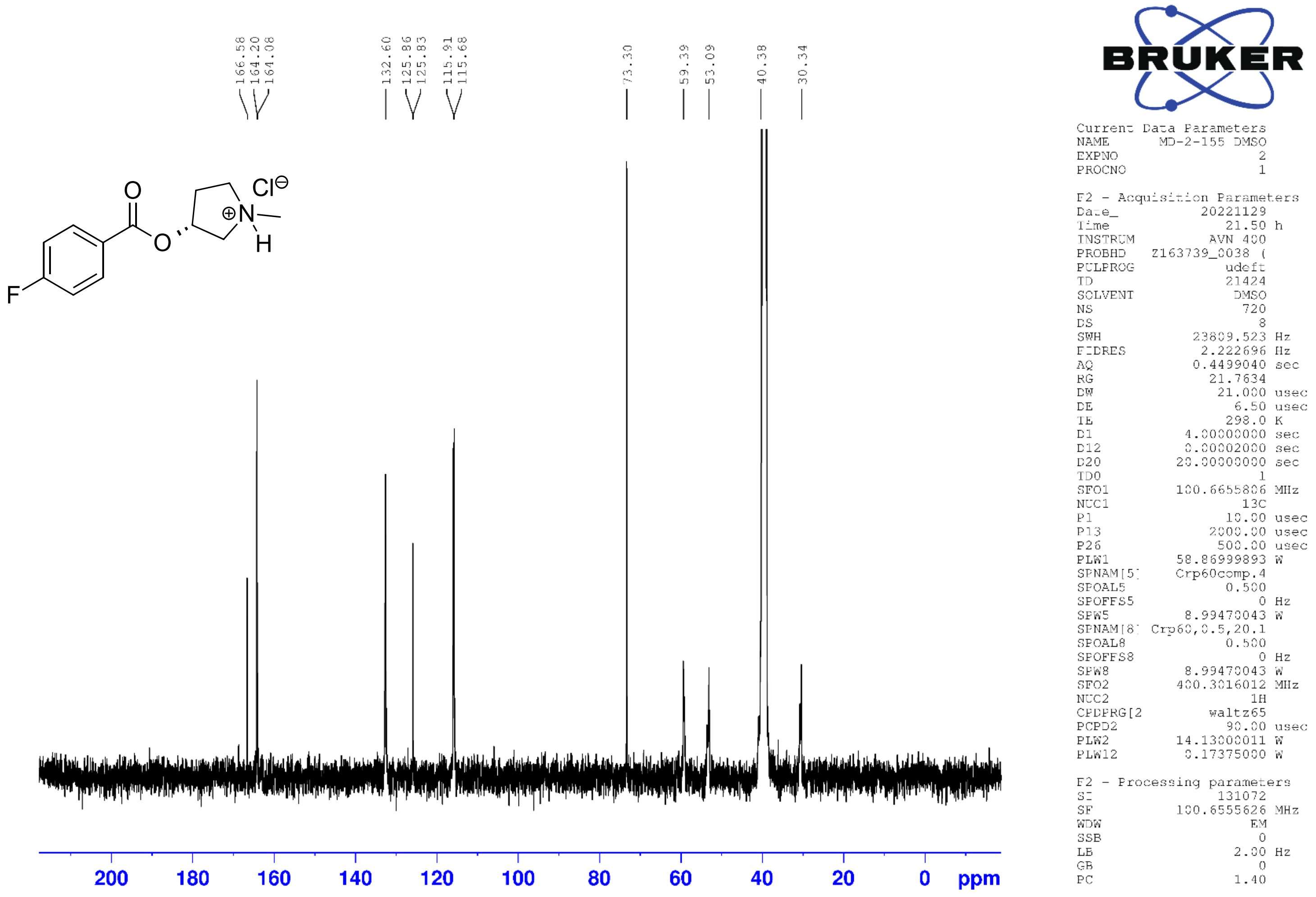

(30) (*3R*)-3-(*p*-Fluorobenzoyloxy)-1-methyl-1-pyrrolidinium Chloride High Resolution Mass Spectrum (ESI, positive mode)

Mass Spectrum SmartFormula Report

Analysis Info

Analysis Name	D:\Data\Xiao\Dec 13 2022000003.d	Acquisition Date	2022-12-13 2:10:47 PM
Method	Xiao all 1.m	Operator	x
Sample Name	TRV6028 HCl	Instrument	compact 8255754.20059
Comment			

Acquisition Parameter

Source Type	ESI	Ion Polarity	Positive	Set Nebulizer	0.5 Bar
Focus	Not active	Set Capillary	3500 V	Set Dry Heater	180 °C
Scan Begin	50 m/z	Set End Plate Offset	-500 V	Set Dry Gas	4.0 l/min
Scan End	1500 m/z	Set Charging Voltage	2000 V	Set Divert Valve	Source
		Set Corona	0 nA	Set APCI Heater	0 °C

Meas. m/z	Ion Formula	m/z	err [ppm]
224.1086	C12H15FNO2	224.1081	-2.1

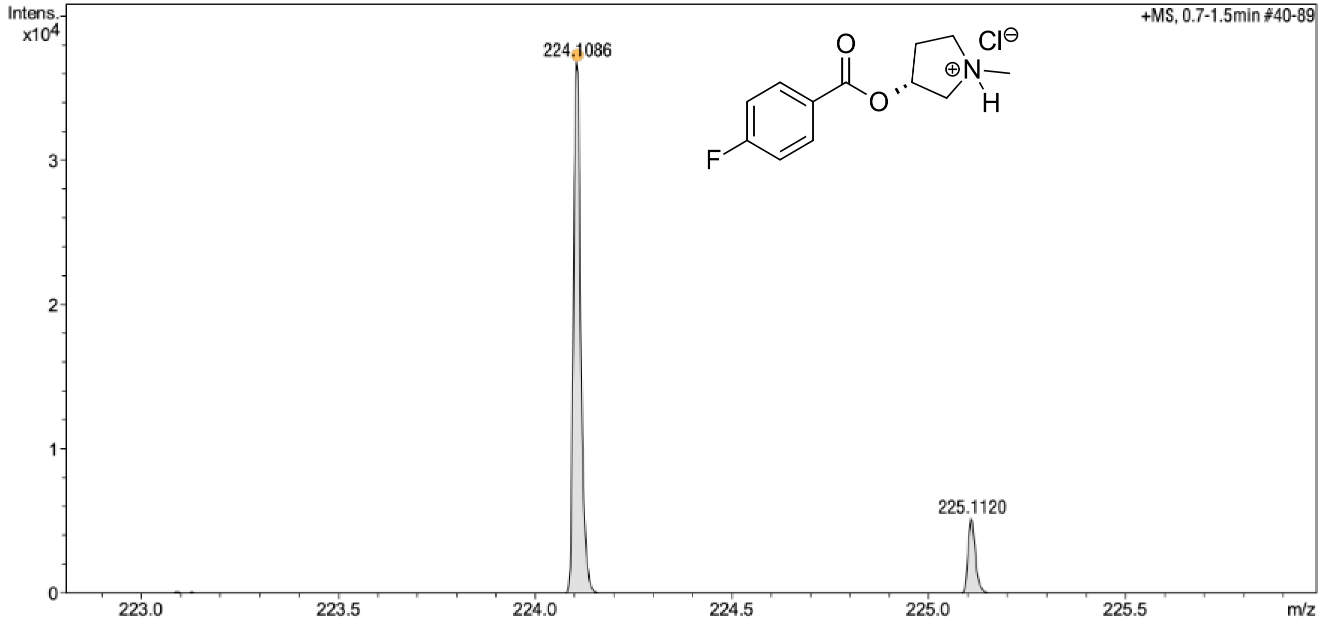

Dec 13 2022000003.d

Bruker Compass DataAnalysis 4.3 printed: 2022-12-13 2:14:03 PM by: x Page 1 of 1

(30) (*3R*)-3-(*p*-Fluorobenzoyloxy)-1-methyl-1-pyrrolidinium Chloride HPLC

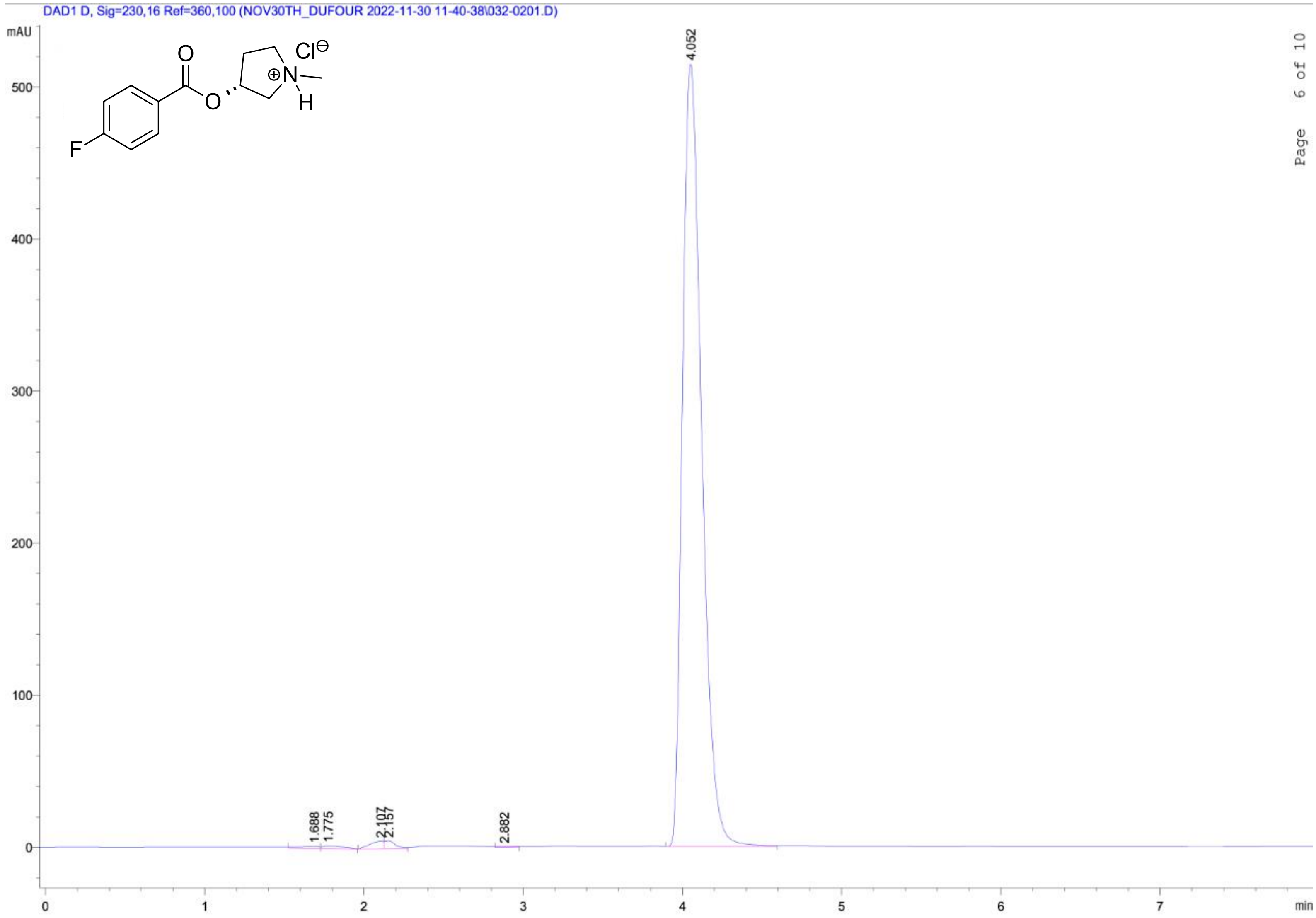

References:

[1] Macdonald IR, Jollymore CT, Reid GA, Pottie IR, Martin E, Darvesh S. Thioesters for the in vitro evaluation of agents to image brain cholinesterases. J Enzyme Inhib Med Chem. 2013;28(3):447-455. DOI: 10.3109/14756366.2011.647008

[2] Bormans G, Sherman P, Snyder SE, Kilbourn MR. Synthesis of carbon-11- and fluorine-18-labeled 1-methyl-4-piperidyl-4'-fluorobenzoate and their biodistribution in mice. Nucl Med Biol. 1996;28(4):513-517. DOI 10.1016/0969-8051(96)00033-9.

[3] Hoeltje HD, Jensen B, Lambrecht G. Cyclic acetyl choline analogs part 4 4 acetoxy piperidines and 4 acetoxythia cyclo hexanes. Eur J Med Chem. 1978;13(5):453-464.

[4] Kikuchi T, Fukushi K, Ikota N, Ueda T, Nagatsuka S-I, Arano Y, Irie T. Synthesis of piperidinyl and pyrrolidinyl butyrates for potential in vivo measurement of cerebral butyrylcholinesterase activity. J Labelled Compd Radiopharm. 2001;44(1):31-41. DOI: 10.1002/jlcr.429.

[5] Cheng CY, Brochmann-Hanssen E, Waters JA. Quantitative structure-activity relationships of aromatic esters of 1-methyl-4-piperidinol as analgesic. J Med Chem. 1982;25(2):145-152. DOI: 10.1021/jm00344a011.

[6] Waters JA. Aromatic esters of nonquaternary carbon-4 piperidinols as analgesics. J Med Chem. 1978;21(7):628-633. DOI: 10.1021/jm00205a007.

[7] Macdonald IR, Reid GA, Joy EE, Pottie IR, Matte G, Burrell S, Mawko G, Martin E, Darvesh S. Synthesis and preliminary evaluation of piperidinyl and pyrrolidinyl iodobenzoates as imaging agents for butyrylcholinesterase. Mol Imaging Biol. 2011;13(6):1250-1261. DOI: 10.1007/s11307-010-0448-0.

B. Figures for histochemistry

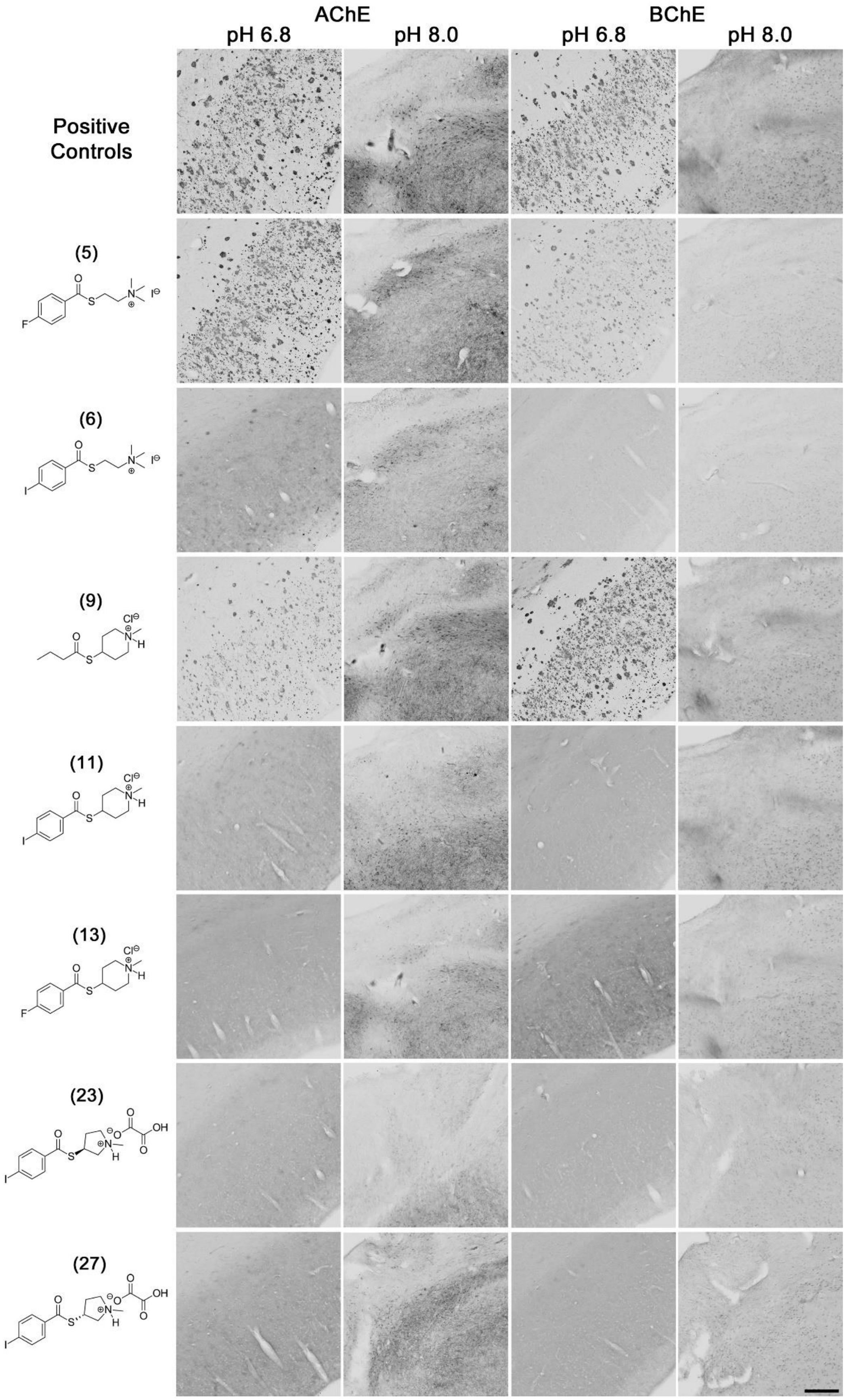
AChE
BChE
pH 6.8
pH 8.0
pH 6.8
pH 8.0
Positive Controls
(5)
(6)
(9)
(11)
(13)
(23)
(27)

Figure S1. Representative photomicrographs of histochemical staining of AChE (columns 1 and 2) and BChE (columns 3 and 4) at pH 6.8 and 8.0 in human brain tissues demonstrating that, when used as inhibitors, some compounds engaged with cholinesterases. Optimal staining for plaques at pH 6.8 is shown in the orbitofrontal cortex, optimal staining of normal neural elements at pH 8.0 is shown in the thalamus. Scale bar =400 µm.

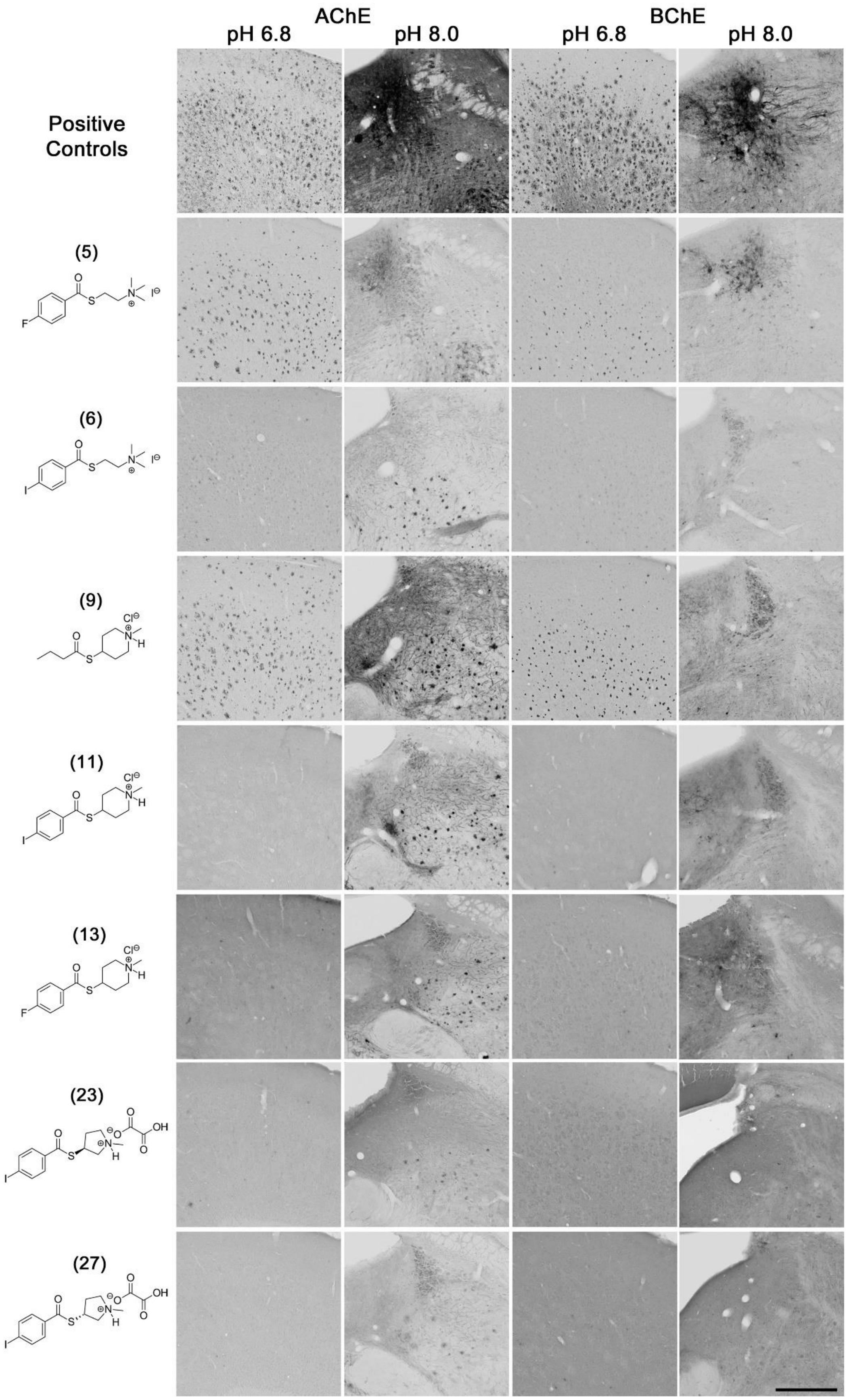
AChE
BChE
pH 6.8
pH 8.0
pH 6.8
pH 8.0
Positive Controls
(5)
(6)
(9)
(11)
(13)
(23)
(27)

Figure S2. Representative photomicrographs of histochemical staining of AChE (columns 1 and 2) and BChE (columns 3 and 4) at pH 6.8 and 8.0 in mouse brain tissues demonstrating that, when used as inhibitors, some compounds engaged with cholinesterases. Optimal staining for plaques at pH 6.8 is shown in the orbitofrontal cortex, optimal staining of normal neural elements at pH 8.0 is shown in the thalamus. Scale bar = 200 µm.
